# Development of a multi-targeted chemotherapeutic approach based on G-quadruplex stabilisation and carbonic anhydrase inhibition

**DOI:** 10.1080/14756366.2024.2366236

**Published:** 2024-06-18

**Authors:** Alessio Nocentini, Anna Di Porzio, Alessandro Bonardi, Carla Bazzicalupi, Andrea Petreni, Tarita Biver, Silvia Bua, Simona Marzano, Jussara Amato, Bruno Pagano, Nunzia Iaccarino, Stefano De Tito, Stefano Amente, Claudiu T. Supuran, Antonio Randazzo, Paola Gratteri

**Affiliations:** a NEUROFARBA Department, Pharmaceutical and Nutraceutical Section and Laboratory of Molecular Modeling Cheminformatics & QSAR, University of Florence, Sesto Fiorentino, Florence, Italy; b Department of Pharmacy, University of Naples Federico II, Naples, Italy; c Department of Chemistry “Ugo Schiff”, University of Florence, Sesto Fiorentino, Florence, Italy; d Department of Chemistry and Industrial Chemistry, University of Pisa, Pisa, Italy; e Research Institute of the University of Bucharest (ICUB), Bucharest, Romania; f Molecular Cell Biology of Autophagy, The Francis Crick Institute, London, UK; g Department of Molecular Medicine and Medical Biotechnologies, University of Naples Federico II, Naples, Italy

**Keywords:** G-Quadruplex, carbonic anhydrase, multitarget-directed ligands, tumours

## Abstract

A novel class of compounds designed to hit two anti-tumour targets, G-quadruplex structures and human carbonic anhydrases (hCAs) IX and XII is proposed. The induction/stabilisation of G-quadruplex structures by small molecules has emerged as an anticancer strategy, disrupting telomere maintenance and reducing oncogene expression. hCAs IX and XII are well-established anti-tumour targets, upregulated in many hypoxic tumours and contributing to metastasis. The ligands reported feature a berberine G-quadruplex stabiliser scaffold connected to a moiety inhibiting hCAs IX and XII. *In vitro* experiments showed that our compounds selectively stabilise G-quadruplex structures and inhibit hCAs IX and XII. The crystal structure of a telomeric G-quadruplex in complex with one of these ligands was obtained, shedding light on the ligand/target interaction mode. The most promising ligands showed significant cytotoxicity against CA IX-positive HeLa cancer cells in hypoxia, and the ability to stabilise G-quadruplexes within tumour cells.

## GRAPHICAL ABSTRACT

**Figure:**
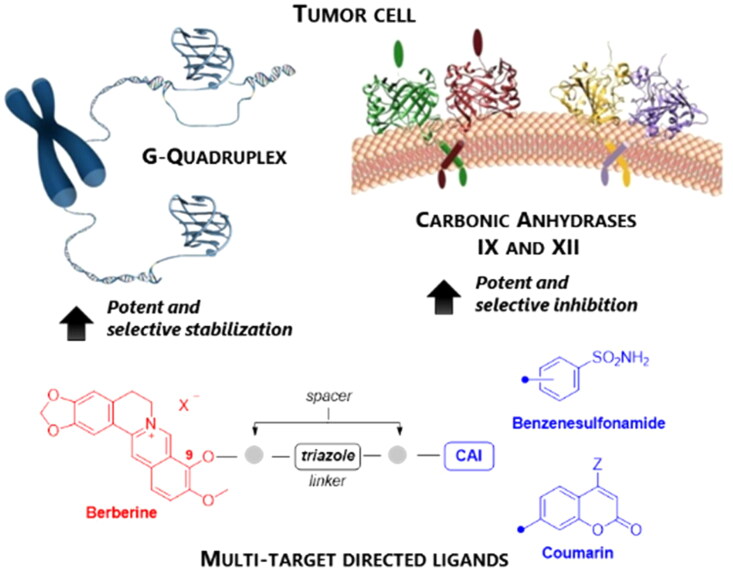

## Introduction

G-Quadruplexes (GQs) are noncanonical nucleic acid secondary structures recognised to play major roles in carcinogenesis^1^. GQs consist of four-stranded nucleic acid helical structures formed by the stacking of two or more guanine tetrads and stabilised by monovalent cations^2^. Guanine-rich sequences able to form GQs are widely distributed in the human genome and are mostly present in regulatory genomic regions responsible for uncontrolled cell proliferation and/or oncogenic transformation^3^. GQ motifs are in fact over-represented at the ends of chromosomes, in telomeres, being involved in maintaining chromosome stability^4^. About 85% of human cancers show overexpression of telomerase which contributes to cancer cell immortality by preventing telomere shortening, leading to uncontrolled proliferation^5^. GQ motifs are often able to inhibit telomerase action and obstruct DNA replication and repair, leading to activation of the DNA damage response pathway that results in apoptosis.

Nonetheless, the majority of GQs are present outside telomeres, such as in the promoter regions of several oncogenes, including c-MYC, c-KIT, KRAS, PDGF-A, hTERT, Rb, RET and BCL-2^4–7^. Stabilisation of such GQ structures was shown to produce the downstream silencing of oncogene expression. Being GQ structures over-represented in telomeres, oncogenes and regulatory genes, and under-represented in housekeeping and tumour suppressor genes, compounds able to selectively bind GQ structures over duplex DNA are promising and innovative chemotherapeutics, potentially interfering with cancer onset and progression without impairing healthy cells^8–10^. To date, a number of small molecules have been identified which can bind and stabilise GQs such as acridine, bis-triazolyl-pyridine, quindoline, phenanthroline, fluorenone, psoralene and berberine derivatives^9–15^. Furthermore, pieces of evidence have shown that GQ structures may be relevant also in the RNA world, being found in lncRNAs, 5′- and 3′-untranslated regions (UTRs) of mRNAs^16–18^. Also in this case, compounds able to interact with RNA GQs have displayed encouraging antitumoral effects^19^.

Inhibition of human carbonic anhydrases (hCAs) IX and XII has been consolidated over the last two decades as another innovative chemotherapeutic strategy against solid (and hypoxic) tumours^20^^,^^21^. hCAs IX and XII play a crucial role in the process of tumorigenesis, cancer cell signalling, tumour progression, acidification, and metastasis. A bunch of targeted inhibitors and biologics have been studied in the preclinical setting showing that inhibition of hCAs IX and XII catalytic activity decreases the growth, proliferation, and metastatic potential of several aggressive cancers both *in vitro* and *in vivo*^21^. **SLC-0111** is the first in class hCAs IX and XII inhibitor entering clinical trials and currently facing Phase Ib/II for the treatment of advanced hypoxic tumours^22^.

Resistance to chemotherapy is a major problem for current cancer research, being responsible for most tumour relapses^23^. Hypoxia, occurring in many solid tumours is a main factor triggering multiple mechanisms of drug resistance^24^. Drug combination therapies have been a successful strategy to enhance efficacy, combat resistance, reduce toxicity and address tumour heterogeneity by concomitant action on more biological targets^25^. Lately, the rational design of multi-target-directed ligands (MTDLs) has risen over drug combination therapies, mainly for the treatment of multi-factorial diseases such as tumours, which depend on a variety of receptors and signalling pathways^26–29^. In comparison to combined therapies, MTDLs (also referred to as molecular hybrids) show improved pharmacokinetics, reaching the target tissue as a unique drug entity, and the absence of drug-drug interactions, producing a greater synergistic effect^28^. MTDLs may thus require lower doses, potentially reducing the side effects and overcoming tolerance to single drugs.

Relevantly, some of us showed that carbonic anhydrase inhibitors (CAIs) such as **SLC-0111** are markedly suitable to cooperate and potentiate the cytotoxic effects of conventional chemotherapeutic drugs that are gemtabicine, dacarbazine, temozolomide, doxorubicin, and 5-fluorouracil^30^^,^^31^. More importantly, our previous studies demonstrated that hCA modulators are well suited to multi-targeting drug design strategies for the treatment of multi-factorial diseases such as ocular pathologies, cancer, inflammation, and neurodegenerative disorders^32–34^. In this context, an early-stage *in vivo* profiling was carried out for some such MTDLs, showing synergistic effects beyond the single-target agents and their equimolar combination.

**Figure 1.**
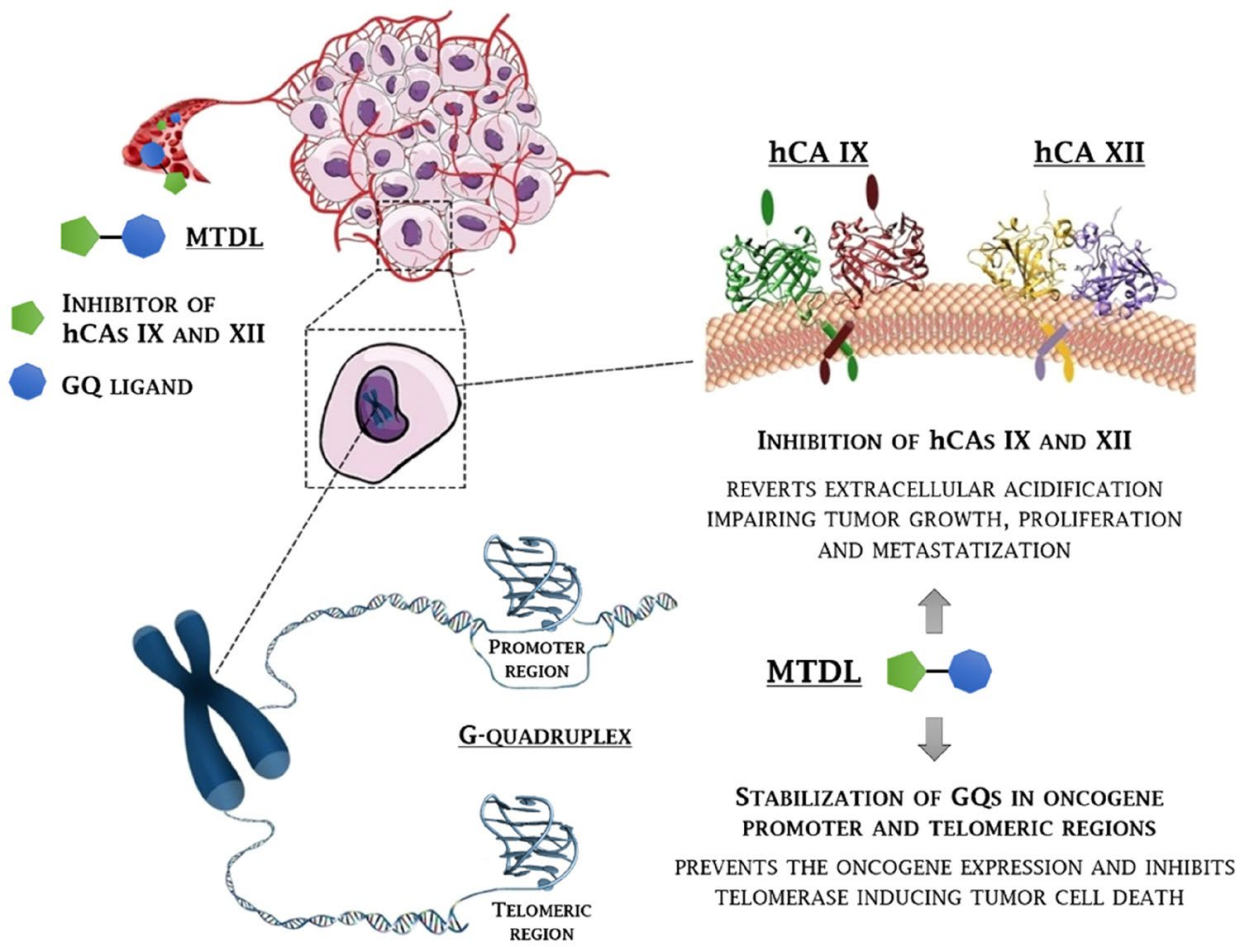
Rationale for the development of GQ ligand–CAI derivatives as innovative chemotherapeutics to disrupt tumour cell growth at multiple levels.

The present work describes the first multi-targeting derivatives capable of stabilising GQ structures and inhibiting hCA IX and XII isoforms as antitumor agents to disrupt tumour cell growth at multiple levels and win drug resistance (Figure 1). Particularly, berberine derivatives including CAI scaffolds of benzenesulfonamide and coumarin types were designed and synthesised, showing potency and selectivity in inhibiting hCAs IX and XII over the off target hCAs I and II, and inducing an effective and selective stabilisation of GQs with respect to a hairpin DNA duplex. The crystal structure of the telomeric GQ in complex with one such MTDL was outstandingly achieved by synchrotron X-ray diffraction, revealing two possible stacking ways over the G-tetrads. Finally, a thorough biological characterisation was conducted on cancer cells which highlighted the therapeutic value of the novel multi-targeting antitumor drug design strategy proposed here.

## Results and discussion

### Drug design

**Figure 2.**
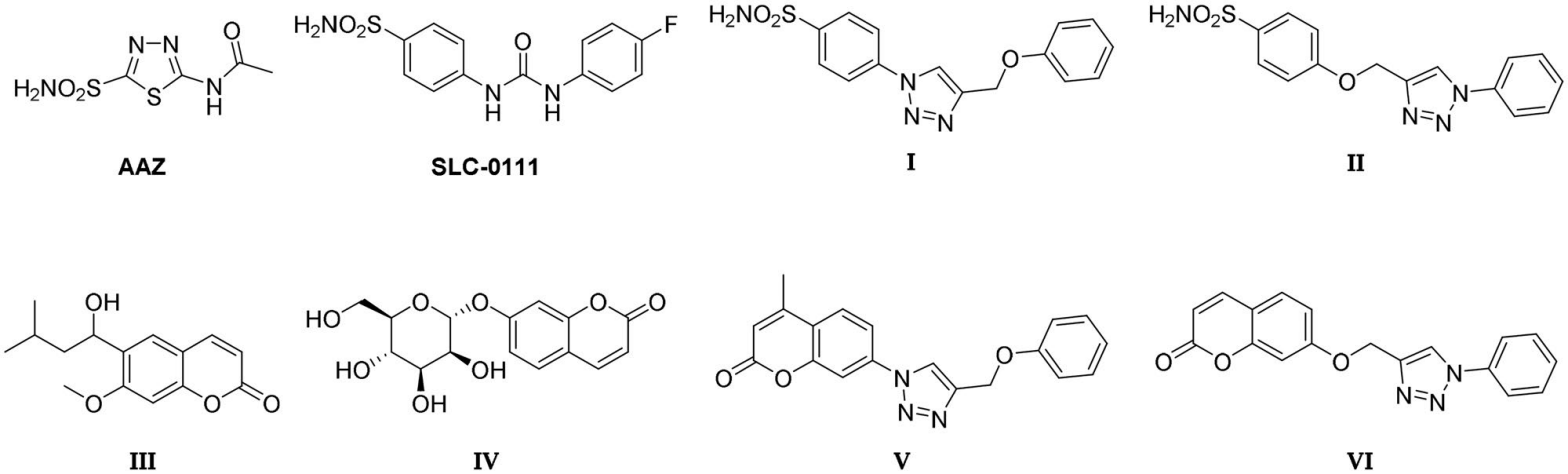
Chemical structure of the carbonic anhydrase inhibitors discussed here.

Selective inhibition of the cancer-associated hCAs is needed to escape the side effects resulting from the modulation of the most abundant and ubiquitous hCA I and II isoforms^35^. The latter might also sequester most inhibitors available in the body, requiring a dose increase. The production of hCAs IX and XII specific inhibitors is the main challenge in the field, being in part successfully overcome by a variety of drug design strategies^21^^,^^35^. Compounds possessing a zinc-binding group (ZBG), such as primary sulphonamides or bioisosters (sulfamates and sulfamides), ensure a potent CA inhibitory action resulting from the displacement of the metal ion-bound nucleophile (water molecule or hydroxide ion). However, first-generation CAIs, such as acetazolamide (**AAZ**, Figure 2) do not show isoform-selective action, leading to side effects resulting from systemic promiscuous CA inhibition^35^. Specific structural optimisation to address isoform selectivity has been pursued over the last two decades, which has been chiefly based on the application of the “tail approach”. The latter has led to a real breakthrough in the field of anticancer CAIs due to improved compound selectivity of action, solubility and physico-chemical properties. As a matter of fact, **SLC-0111** (Figure 2) is a phenylureido-tailed benzene sulphonamide resulting from an extensive application of the tail approach and exhibiting a markedly selective action against hCAs IX and XII over I and II isoforms^24^. Another widely pursued drug design strategy to obtain hCAs IX and XII selective inhibitors addresses alternative mechanisms of action such as the occlusion of the binding site area documented by kinetic, spectroscopic, and crystallographic studies for coumarin derivatives, such as the natural lead compound **III** in Figure 2 ^35^. Coumarin derivatives, after CA-mediated lactone ring cleavage (act as suicide inhibitors), bind further from the zinc-coordination pattern and increase the interaction with residues which differ most within the collection of human CAs. One such compound (*i.e.* **IV** in Figure 2) showed striking antitumor and antimetastatic effects in an animal model of breast cancer, being further evaluated in preclinical models of this disease^36^.

**Figure 3.**
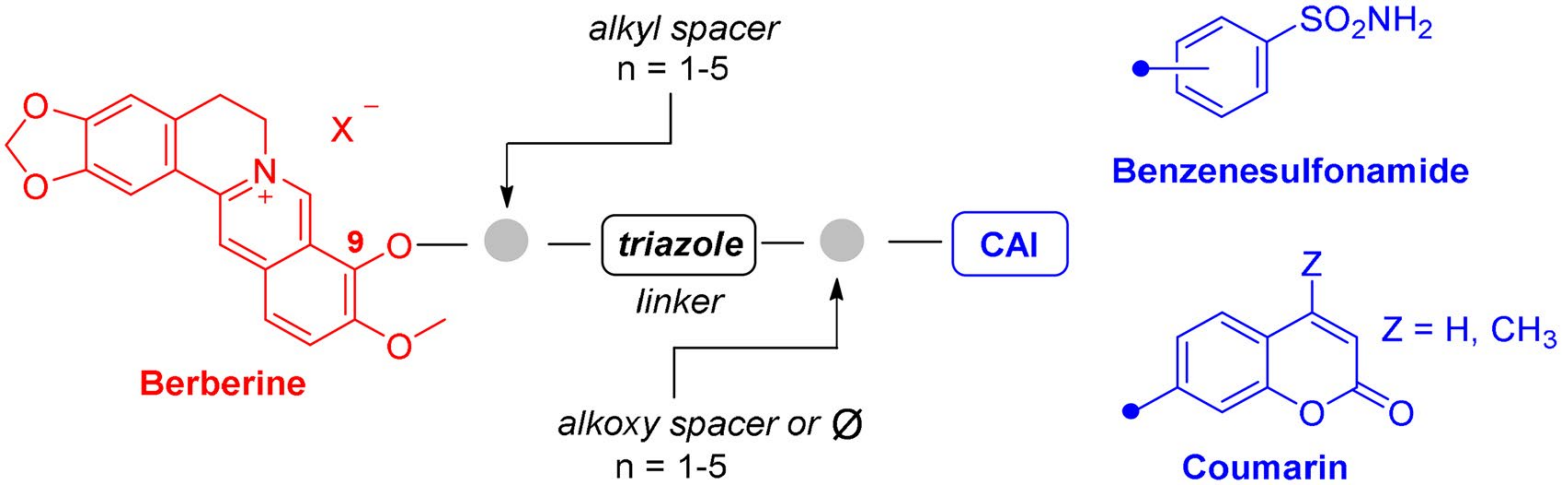
Drug design and general structure of the berberine-CAI derivatives proposed here.

To develop a chemical tool capable of targeting hCAs IX and XII and GQ structures, benzenesulfonamide and coumarin CAI scaffolds were attached to berberine (**1**), a GQ stabilising nucleus, by different 1,2,3-triazole based spacers (Figure 3).

**Figure 4.**
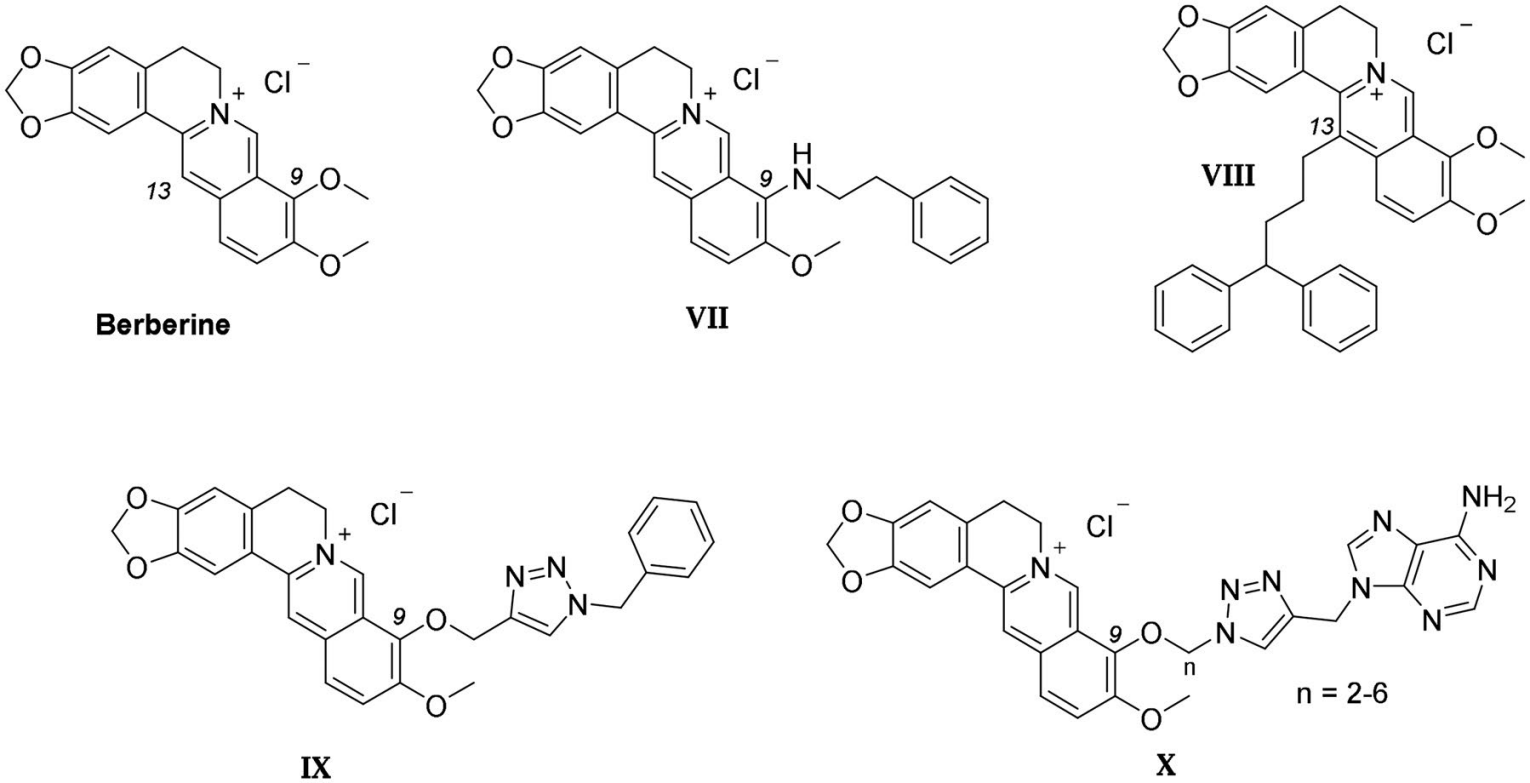
Chemical structure of the berberine derivatives as GQ ligands discussed here.

Berberine (Figure 4) is a natural alkaloid exerting anticancer activities both *in vitro* and *in vivo* through multiple mechanisms, including telomerase inhibition^37^. Berberine proved to bind various GQ structures, including those located at the c-MYC and c-KIT oncogene promoters, with high selectivity with respect to the duplex DNA^38–40^. In fact, the positively charged berberine scaffold appears to be suitable for π–π stacking interactions with the G-tetrads. In 2013, the first X-ray crystal structure of the human telomeric DNA in complex with berberine was reported by some of us, validating a binding stoichiometry higher than the 1:1 previously detected by NMR studies^39^.

Most structural modifications of berberine to produce new anticancer derivatives include mono-derivatization at C9 (substituent of ether, amine, ester or sulphonate types) or C13 (alkylamine or alkylaryl substituents) position of the heterocycle (**VII–X** in Figure 4), both being suitable to improve the GQ binding performance with respect to the lead^41^^,^^42^. Another study appended lipophilic substituents at both C9 and C13 positions, also detecting an increase in the compound antitumor activity^43^. Nonetheless, searching for the additional CA inhibitory action, derivatization at C9 was here preferred over C13 not to induce a “parachute-like” effect (noticeable with compound **VIII** in Figure 4) at the CA active site due to the steric effect expected by CAI attachment at the inner portion of the polycyclic scaffold.

To maintain a 9-ether substituent as in the lead berberine, a 1,4-disubstituted-1,2,3-triazole linker (preferred over 1,5-disubstituted such moieties) was planned to connect the alkaloid to the CAI scaffolds, and different length and type spacers (*i.e.* aliphatic, alkoxy, direct linkage) were explored at the 1 and 4 positions of the triazole. The latter is an amide bioisoster endowed with a moderate dipole character, hydrogen bonding capability, *in vivo* stability, and an aromatic character allowing π-π stacking interactions with the biological target^44^. In previous studies by some of us, the 1,4-disubstituted-1,2,3-triazole linker was used as an amide or urea bioisoster as a novel application/lead optimisation of the tail approach with benzenesulfonamide (**I** and **II** in Figure 2) and coumarin (**V** and **VI** in Figure 2) CAIs^45^^,^^46^. The new triazole derivatives demonstrated markedly enhanced CA inhibition potency and, in some cases, isoform selectivity. X-ray crystallography and *in silico* studies with hCAs pointed out a reinforced binding to the target resulting from interactions of the triazole linker within the enzyme active site.

Importantly, the Cu(I) catalysed azide − alkyne cycloaddition (CuAAC) used to generate a 1,4-disubstituted-1,2,3-triazole also demonstrated the ease and versatility necessary for producing 9-ether-berberine-base derivatives with acceptable purity and yields. Interestingly, small series of berberine derivatives including triazole-based substituents at the 9-position of the scaffold (**IX** and **X** in Figure 4), have been already reported, showing improved binding to GQs and greater in-cell anticancer activity than the lead berberine^47^^,^^48^.

### Chemistry

**Scheme 1.**
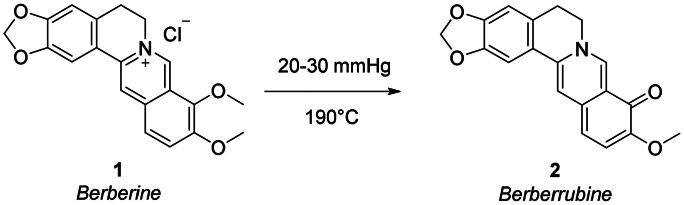
Synthetic path to yield berberrubine **2** from berberine hydrochloride **1**.

**Scheme 2.**
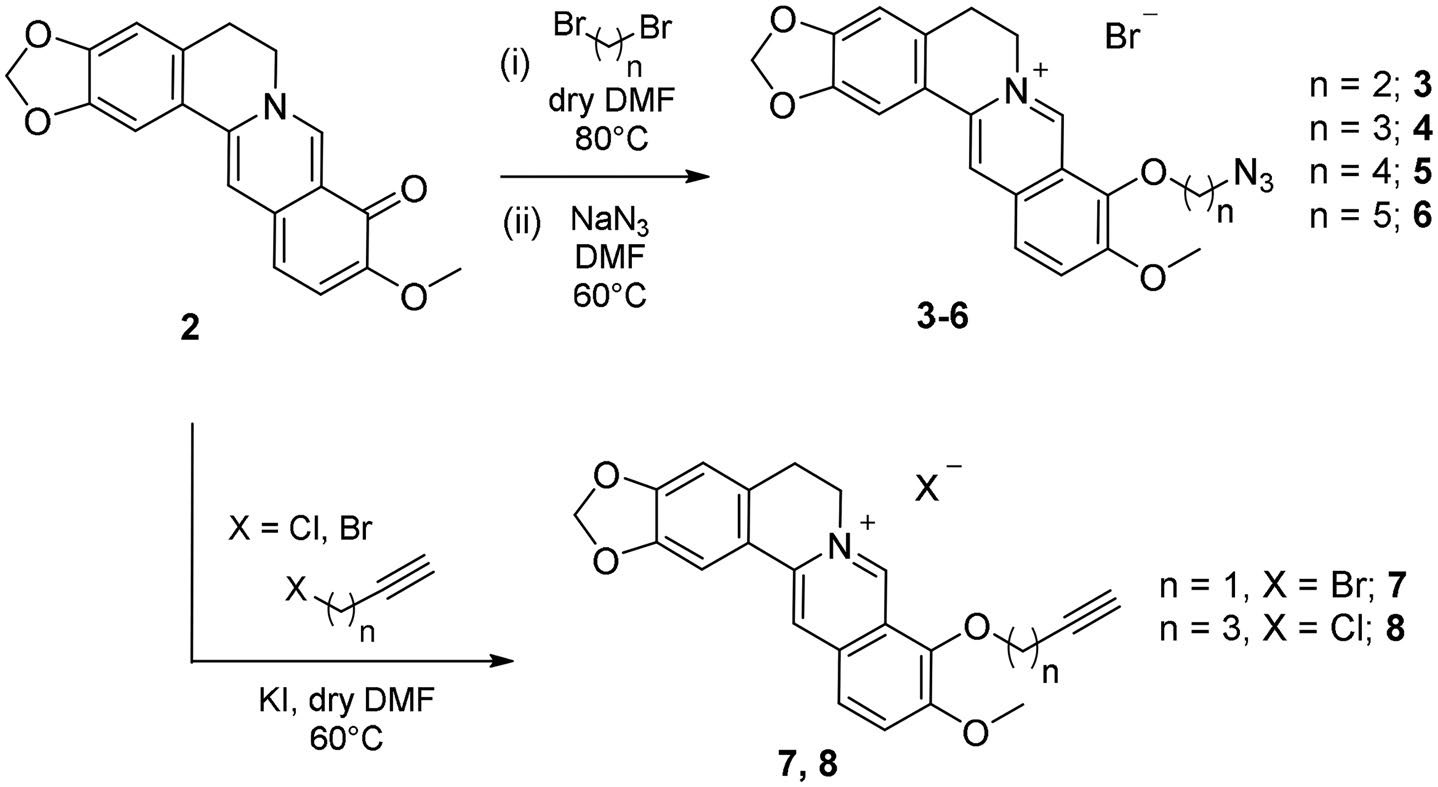
Synthetic pathway to yield the berberine azide (**3–6**) and alkyne (**7**, **8**) intermediates.

To append substituents at the 9-position of berberine, the natural alkaloid **1** was primarily demethylated by under vacuum treatment (20–30 mmHg) at 190 °C for 2 h to give berberrubine **2** (Scheme 1). The demethylation elicits the loss of the permanent positive charge of the nucleus via electron rearrangement with the compound colour switching from yellow to red. Therefore, nucleophilic substitution reactions with haloalkyl reagents were used to convert berberrubine back to the positively charged berberine structure including 9-substituents other than the methyl group and a chloride or bromide counterion. In detail, berberrubine **2** was treated with 3 to 5 carbon atom length dibromoalkanes in nucleophilic substitution conditions in anhydrous DMF at 80 °C, followed by treatment with sodium azide in DMF at 60 °C to yield azide derivatives **3–6** (Scheme 2).

As well, berberrubine **2** was reacted with alkynyl halides (chloride or bromide) of varying lengths, in presence of potassium iodide in DMF to give the terminal alkyne intermediates **7** and **8** (carbonic anhydrases). The 3-butynyl such derivative was not obtained despite a variety of attempted reaction conditions (including microwave-assisted synthesis).

**Scheme 3.**
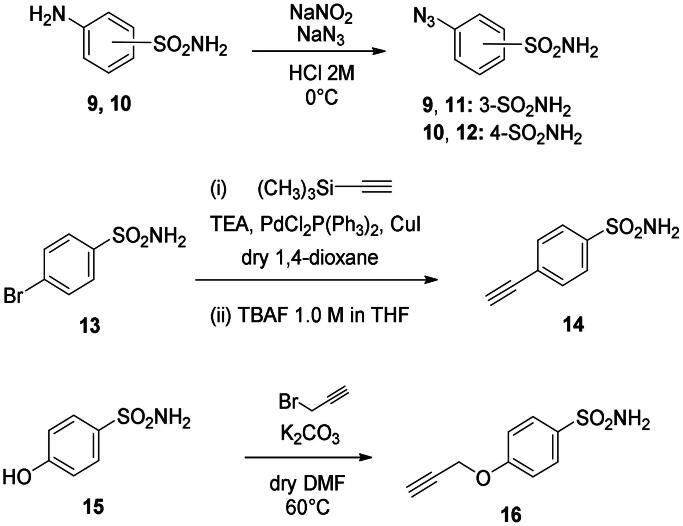
Synthesis of benzenesulfonamide azide (**11**, **12**) and alkyne (**14, 16**) intermediates.

Metanilamide **9** and sulphanilamide **10** were converted to the corresponding aromatic azides **11** and **12** *via* Sand-Meyer reaction with sodium nitrite and sodium azide in aqueous HCl 2 M (carbonic anhydrases). To include a terminal alkyne group with benzenesulfonamide derivatives, two different synthetic pathways were adopted: (i) 4-bromobenzenesulfonamide **13** was reacted with trimethylsilylacetylene by a Sonogashira reaction performed at rt in dry dioxane in presence of PdCl_2_(PPh_3_)_2_, CuI and TEA and therefore treated with TBAF in dry THF to achieve the alkyne derivative **14**; (ii) 4-hydroxybenzenesulfonamide **15** was treated with propargyl bromide in presence of potassium carbonate in anhydrous DMF at 60 °C to afford alkyne **16** (Scheme 3).

**Scheme 4.**
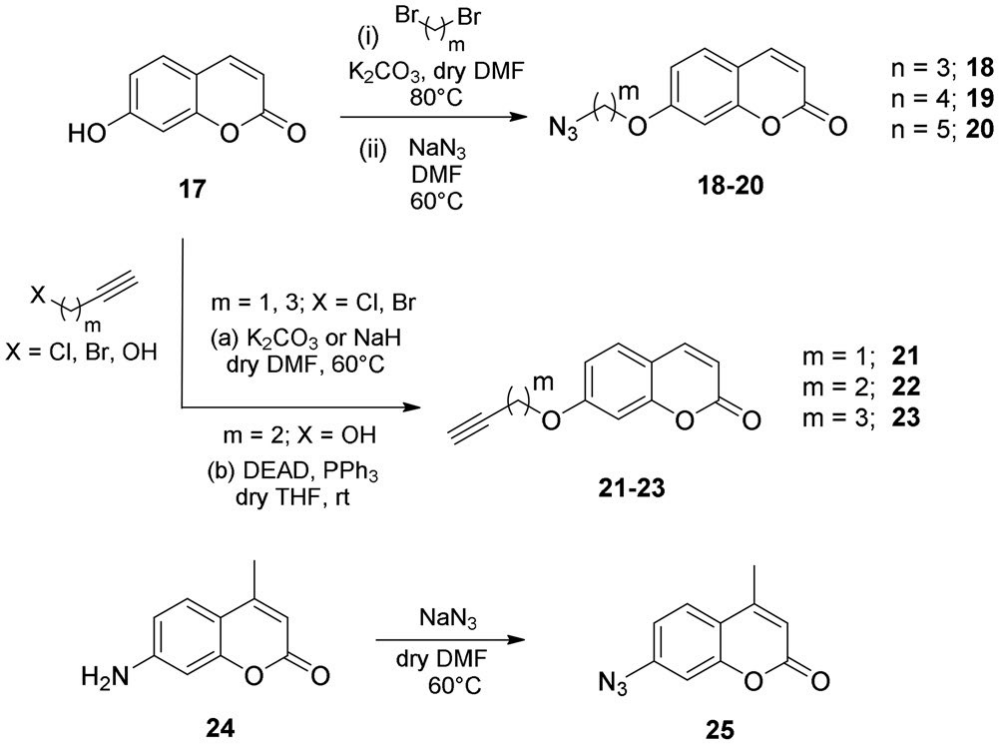
Synthesis of coumarin azide (**18–20** and **25**) and alkyne (**21–23**) derivatives.

The coumarin reagents for the CuAAC reaction were prepared comparably with the berberine intermediate derivatives shown in *Scheme 2*. Umbelliferon **17** was treated with 3 to 5 carbon atom length dibromoalkanes in presence of potassium carbonate in anhydrous DMF at 80 °C, and successively with sodium azide in DMF at 60 °C to yield the azide derivatives **18–20** (*Scheme 4*). Different reaction types and conditions were used to append terminal alkynyl chains at the 7-position of the coumarin scaffold. Umbelliferon **17** was in fact treated with: (i) propargyl bromide in presence of potassium carbonate in anhydrous DMF at rt to give intermediate **21**; (ii) 3-butyn-1-ol via a Mitsunobu reaction in dry THF in presence of diethyl azodicarboxylate and triphenylphosphine to afford compound **22**; (iii) 5-chloro-1-pentyne in presence of sodium hydride in anhydrous DMF at rt to give alkyne **23**. Finally, the coumarin aromatic azide derivative **26** was obtained from 7-amino-4-methyl-coumarin **24** by a Sand-Meyer reaction with sodium nitrite and sodium azide in aqueous HCl 2 M (Scheme 4).

**Scheme 5.**
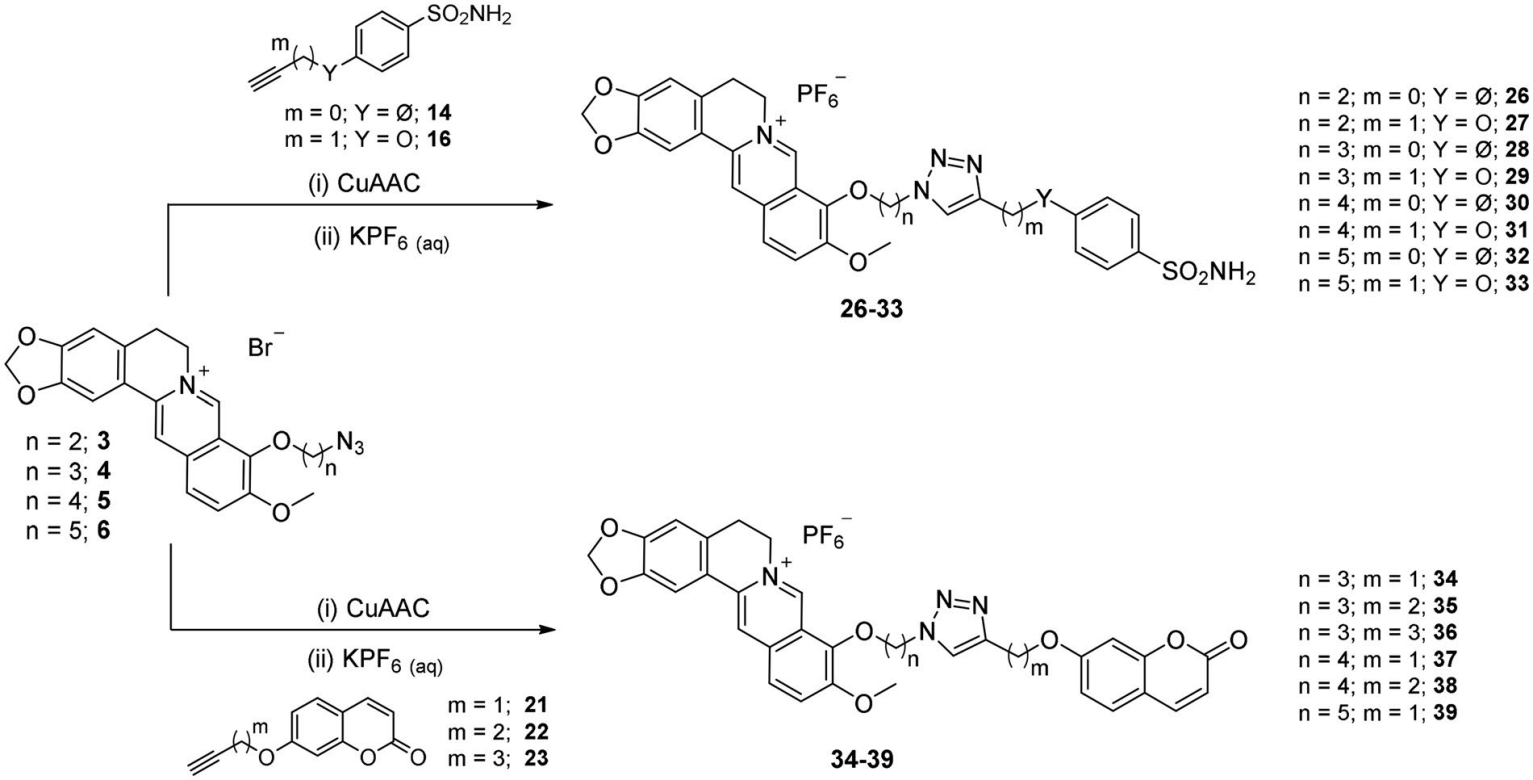
Synthesis of derivatives **26–39** of type ***1*** which include the triazole N^1^ berberine oriented. CuAAC: (i) CuSO_4_ and sodium ascorbate or (ii) Cu nanosized and tetramethylammonium chloride in *t*BuOH/H_2_O at 40 °C.

**Scheme 6.**
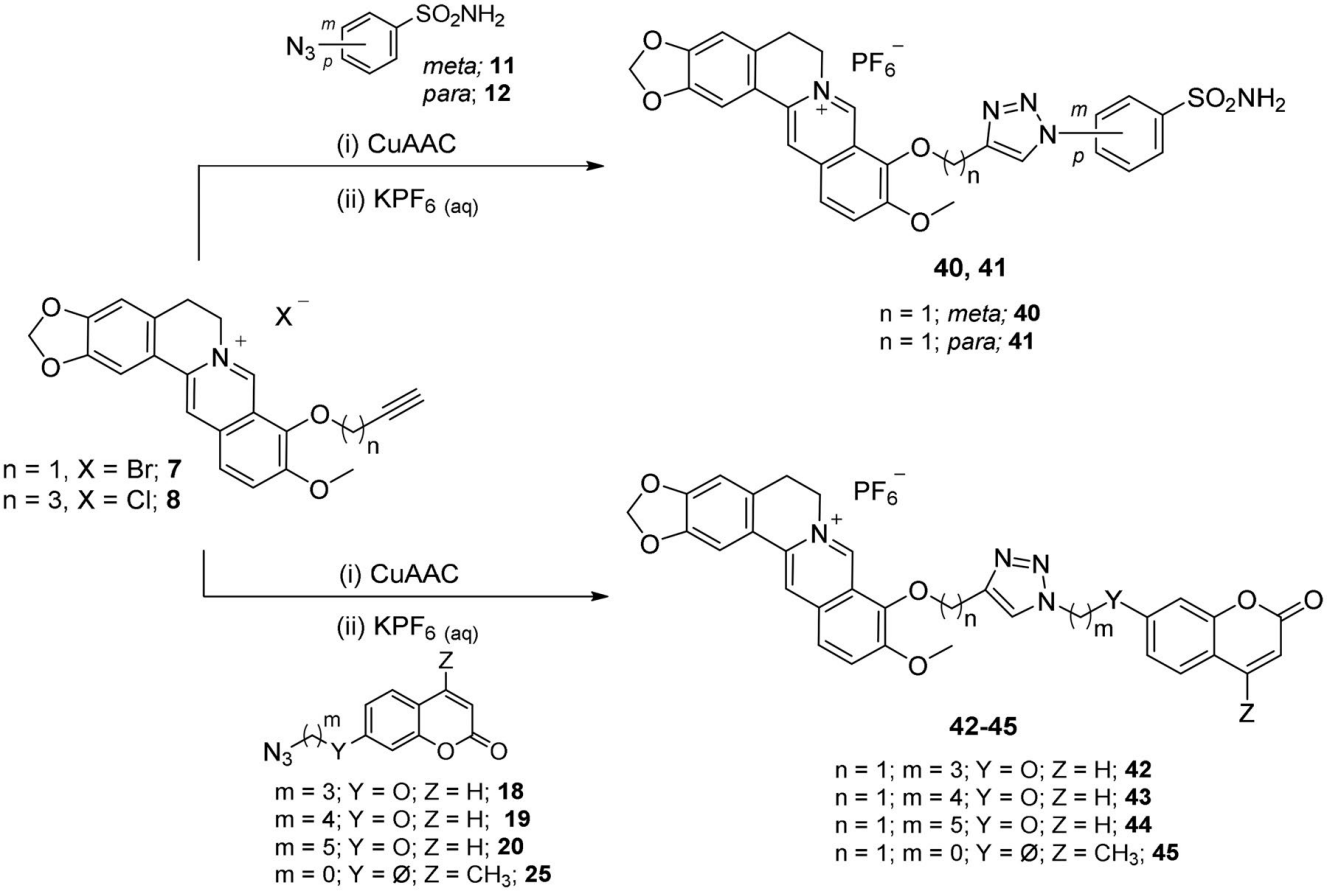
Synthesis of hybrid derivatives **40–45** of type ***2*** which include the triazole N^1^ CAI oriented. CuAAC: (i) CuSO4 and sodium ascorbate or Cu nanosized and tetramethylammonium chloride in *t*BuOH/H_2_O at 40 °C.

The CuAAC reactions between the prepared azide ad alkyne intermediates to form 1,2,3-triazole stably linked berberine-CAI MTDLs were conducted in a water-*t*BuOH mixture as solvent, adopting two different reagent pairs to generate the Cu(I) catalyst: (i) copper(II) sulphate and sodium ascorbate and (ii) copper nanosized and tetramethylammonium chloride. Thus, berberine azide derivatives (**3–6**) were clicked with sulphonamide (**14**, **16**) or coumarin (**21–23**) terminal alkyne compounds to generate derivatives of type ***1*** that include the triazole N^1^ atom berberine oriented (Scheme 5). As well, berberine alkyne derivatives (**7, 8**) were clicked with sulphonamide (**11**, **12**) or coumarin (**18–20, 25**) azide intermediates to generate type ***2*** derivatives **40–45** that incorporate the triazole N^1^ atom CAI oriented (Scheme 6). The 4-pentynyl derivative **8** inexplicably did not click with any of the azide intermediates in several attempted CuAAC conditions.

To have all final compounds with a unique and common counterion and ease the trivial purification process due to the halide salt compound character, the CuAAC reactions were quenched with an aqueous solution of potassium hexafluorophosphate (KPF_6_). Such a lipophilic and inert berberine counterion, devoid of CA modulatory effects, markedly increases the solubility of compounds **26–45** in organic solvents, such as dichloromethane, allowing their extraction from the aqueous reaction medium and separation from inorganic impurities.

**Scheme 7.**
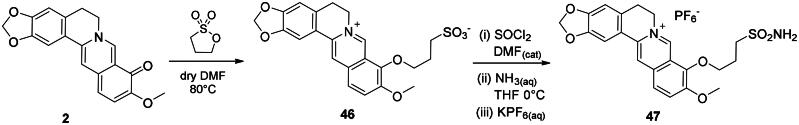
Synthesis of berberine derivative **49** bearing an aliphatic primary sulphonamide moiety.

A unique non-triazole berberine derivative was synthesised, which bears an aliphatic sulphonamide moiety to induce CA inhibition (Scheme 7). Berberrubine **2** was treated with 1,3-propanesulfone in anhydrous DMF at 60 °C to give the zwitterion berberine sulphonate **46**. The latter was treated in refluxing thionyl chloride in presence of catalytic DMF to form the corresponding sulphonyl chloride and therefore with a 30% ammonia solution in THF at 0 °C to give the aliphatic sulphonamide **47**, also isolated as a hexafluorophosphate salt.

Overall, the halide to PF_6_^–^ counterion swap allowed to purify all compounds by column chromatography using silica gel and MeOH/DCM gradients. Compounds **26–45** and **47** were fully characterised by ^1^H-NMR,^13^C-NMR, and HRMS (Supporting Information).

### Carbonic anhydrase inhibition

Primary sulphonamides **26–33**, **40**, **41**, **47** and coumarin derivatives **34–39**, **42–45** were tested for their inhibitory action against the target hCAs IX and XII and hCAs I and II as main off target isoforms by a stopped-flow CO_2_ hydrase assay (Table 1)^49^, using **AAZ** as reference inhibitor and in comparison to **SLC-0111** (Figure 2). Most sulphonamide berberine derivatives showed remarkable inhibition profiles in terms of potency and selectivity, whilst the inhibition constant (K_I_) patterns of the coumarin derivatives were in line with previous findings. The structure-activity relationship is discussed separately for the two compound subsets. Another code pattern is included for the MTDLs in Table 1, which indicates the derivative CAI type (sulphonamide, **S** or coumarin, **C**) and the number of atoms in the spacers connecting either berberine and the triazole (carbon atoms) or the triazole and the benzenesulfonamide/coumarin scaffolds (carbon or oxygen atoms).

**Table 1.** Inhibition data of human CA I, II, IX, and XII isoforms with sulphonamides **26–33**, **40**, **41**, **47** and coumarins **34–39**, **42–45** derivatives and the standard sulphonamide inhibitor acetazolamide (**AAZ**) by a stopped-flow CO_2_ hydrase assay^49^.

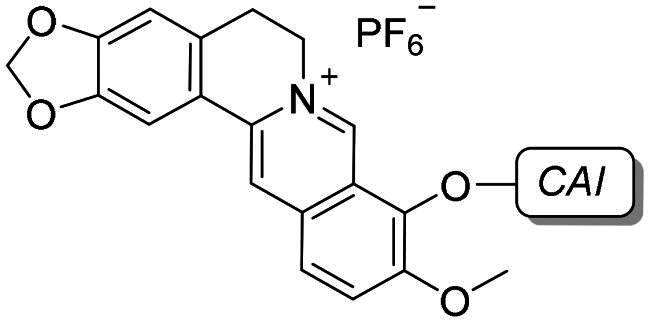						
Compound		*CAI*	K_I_ (nM)^a^^,b^			
			hCA I	hCA II	hCA IX	hCA XII
**26**	**S-2,0**	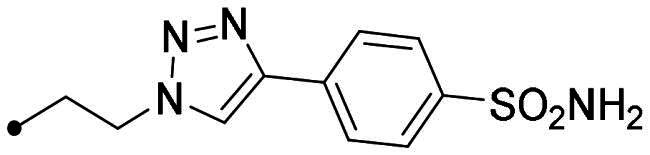	580.5 ± 32.5	59.4 ± 3.8	1.4 ± 0.1	7.3 ± 0.5
**27**	**S-2,2**	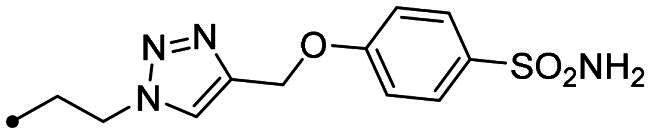	940.2 ± 52.1	6.0 ± 0.4	0.65 ± 0.05	4.4 ± 0.3
**28**	**S-3,0**	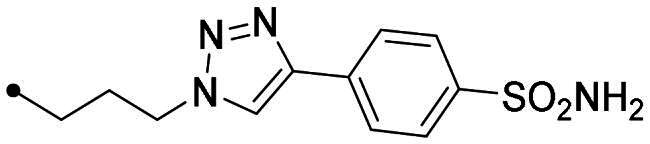	872.1 ± 55.9	66.8 ± 3.9	0.73 ± 0.04	14.0 ± 1.0
**29**	**S-3,2**	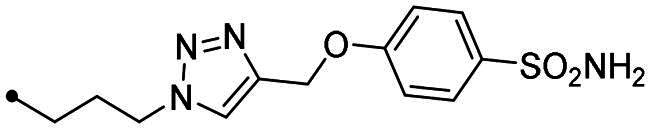	805.0 ± 60.8	62.3 ± 4.7	0.56 ± 0.03	31.2 ± 2.5
**30**	**S-4,0**	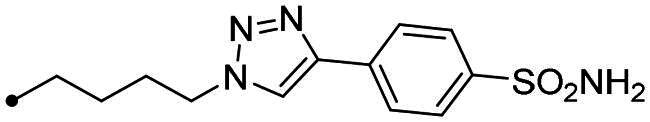	592.4 ± 36.3	55.1 ± 3.8	0.49 ± 0.03	25.5 ± 1.4
**31**	**S-4,2**	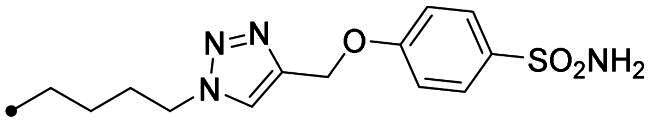	915.6 ± 57.9	71.5 ± 5.3	0.71 ± 0.04	7.2 ± 0.5
**32**	**S-5,0**	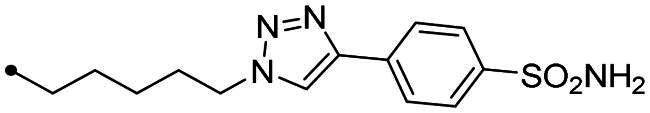	573.6 ± 35.8	15.8 ± 1.2	0.24 ± 0.02	18.3 ± 1.0
**33**	**S-5,2**	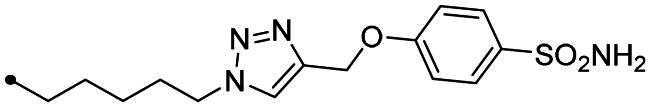	219.5 ± 16.2	41.1 ± 2.7	0.54 ± 0.03	2.9 ± 0.1
**40**	**S-1,0*m***	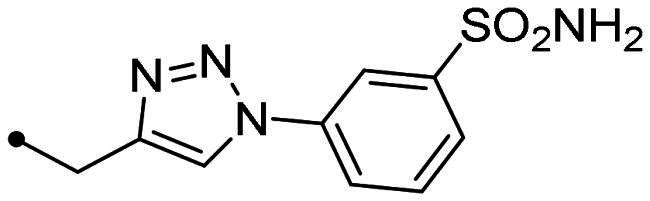	3670 ± 22	90.1 ± 5.6	3.9 ± 0.2	24.8 ± 1.3
**41**	**S-1,0*p***	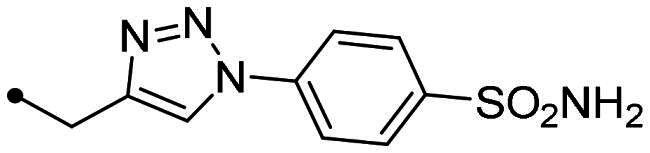	829.1 ± 50.3	22.9 ± 1.4	2.7 ± 0.2	6.1 ± 0.3
**47**	**S-3,a**	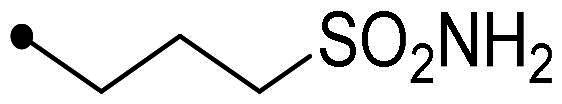	75.1 ± 5.4	21.5 ± 1.6	17.8 ± 0.9	35.9 ± 2.1
**34**	**C-3,2**	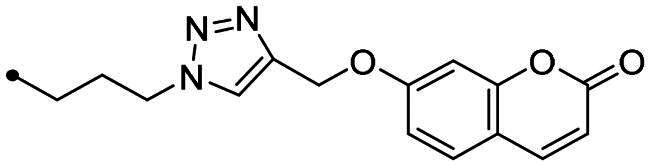	>100 µM	>100 µM	66.0 ± 4.3	30.1 ± 1.5
**35**	**C-3,3**	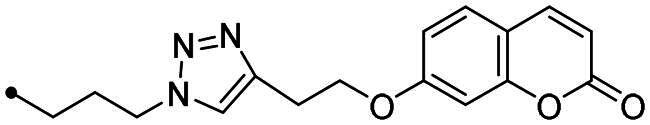	>100 µM	>100 µM	34.5 ± 2.4	18.9 ± 0.9
**36**	**C-3,4**	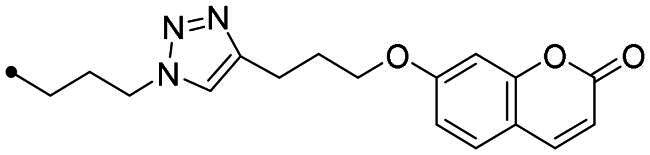	>100 µM	>100 µM	58.9 ± 3.9	28.4 ± 1.7
**37**	**C-4,2**	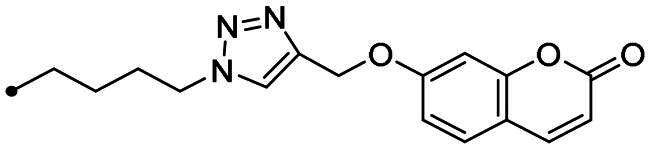	>100 µM	>100 µM	17.3 ± 1.2	8.4 ± 0.5
**38**	**C-4,3**	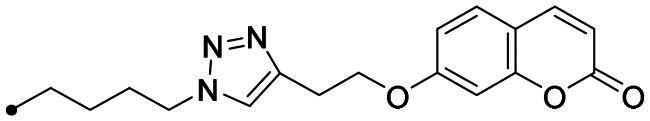	>100 µM	>100 µM	30.1 ± 1.8	16.7 ± 1.2
**39**	**C-5,2**	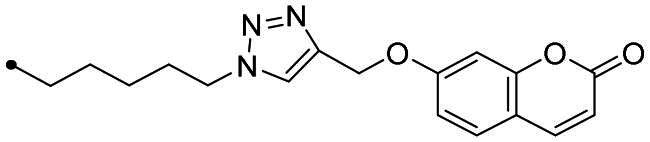	>100 µM	>100 µM	48.6 ± 2.6	20.1 ± 1.3
**42**	**C-1,4**	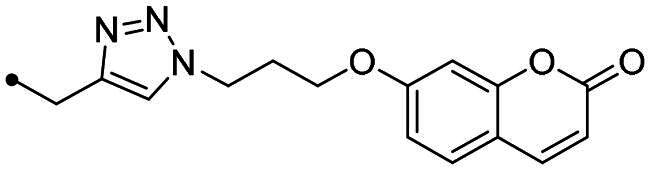	>100 µM	>100 µM	35.1 ± 2.8	10.2 ± 0.8
**43**	**C-1,5**	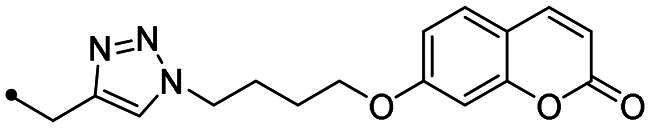	>100 µM	>100 µM	25.5 ± 1.5	4.2 ± 0.3
**44**	**C-1,6**	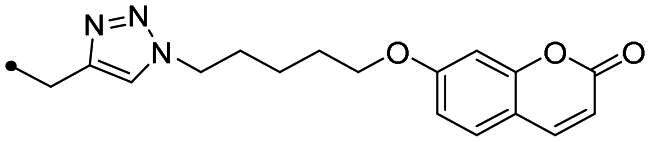	>100 µM	>100 µM	9.6 ± 0.5	14.5 ± 0.9
**45**	**C-1,0c**	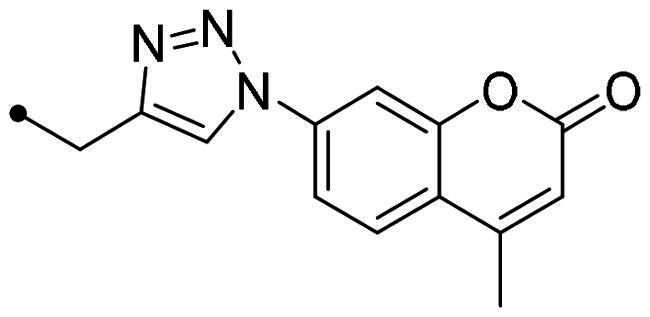	>100 µM	>100 µM	51.5 ± 3.6	5.4 ± 0.3
**AAZ^c^**		–	250	12.5	25.0	5.7
**SLC-0111^c^**		–	5080	960	45	4.5

^a^ Inhibition data are expressed as means ± SEM of three different assays. ^b^inhibitor-enzyme incubation time: 15 min for sulphonamides (S); 6 h for coumarins (C); ^c^from ref. 16. The off target hCA I was the least inhibited isoform by all sulphonamide derivatives **26–33**, **40**, **41**, **47** with K_I_ values in the range 75.1–3670 nM. The *p*-substituted benzenesulfonamides **26–33, 41** showed a rather flat SAR, based on K_I_ values in the narrow range 580.5–940.2 nM, except for compound **33** (**S-5,2**) that reported a K_I_ value of 219.5 nM. The latter is endowed with the longest spacers at the triazole sides, presumably promoting the interaction within the narrow hCA I active site. As a matter of fact, the unhindered aliphatic sulphonamide **47** stood out as the most effective hCA I inhibitor, exhibiting a K_I_ of 75.1 nM. In contrast, the most hindered *m*-substituted benzenesulfonamide **40** inhibited hCA I only in the micromolar range (K_I_ of 3670 nM). The most physiologically relevant hCA II was effectively inhibited by most sulphonamide compounds with K_I_s in a low-medium nanomolar range, *i.e.* 6.0–90.1 nM. Again, the SAR of the *p*-substituted benzenesulfonamides **26–33, 41** was rather flat. Of this subset, compounds **27** (**S-2,2**) and **32** (**S-5,0**) stood out as the most effective hCA II inhibitors with K_I_s of 6.0 and 15.8 nM, respectively. In the case of hCA II, the aliphatic sulphonamide derivative **47** reported a medium inhibitory trend with a K_I_ of 22.1 nM. The *m*-substituted benzenesulfonamide **40** was also the weaker inhibitor against hCA II (K_I_ of 90.1 nM). With the only exception of derivative **47** (K_I_ of 17.8 nM), all sulphonamide compounds **26–33**, **40**, **41** were single-digit to subnanomolar inhibitors of the main target hCA IX. In detail, derivatives **26**, **40**, and **41** bearing the shortest spacers at the triazole sides showed the highest K_I_ values, which are 1.1, 3.9, and 2.7 nM, respectively. The other medium-length benzenesulfonamides unexpectedly exhibited 0.2 to 0.7 nM inhibition constants against hCA IX. Compound **32** (**S-5,0**) stood out as the most potent hCA IX inhibitor of the set (K_I_ of 0.24 nM). Such a general trend of high efficacy in the sulphonamide subset despite differences in the spacer length suggests that the berberine nucleus, as a molecular tail, positively impacts the interaction of benzenesulfonamide derivatives with the hCA IX active site. Indeed, structural studies showed that the binding cleft of the tumour-associated hCA IX is roomier and shows more accessory pockets than the ubiquitous hCAs I and II, and even the related hCA XII, making it possible to better accommodate the polycyclic berberine core. Notably, 10- to more than 100-fold improved K_I_ values were measured for the sulphonamide compounds here reported with respect to **SLC-0111** (K_I_ of 45.0 nM against hCA IX). The other target hCA XII was the second most inhibited isoform by most sulphonamide compounds **26–33**, **40**, **41** with K_I_s in a low-medium nanomolar range, *i.e.* 2.9 – 31.2 nM. In fact, only **32** and **47** comparably or more effectively targeted hCA II *vs* hCA XII. In the subset of single-digit nanomolar hCA XII inhibitors, **27** (**S-2,2**) and **33** (**S-5,2**) demonstrated the most effective inhibitory properties with K_I_s of 4.4 and 2.9 nM, respectively, that are comparable or better than those measured for **SLC-0111** (K_I_ of 4.5 nM). It can be speculated that **27** and **33** have proper spacer lengths to promote the interaction of the positively charged berberine core with specific accessory pockets present in the hCA XII active site, more hydrophilic than hCAs I, II, and IX. The aliphatic derivative **47** showed the worst, though effective, hCA XII inhibition with a K_I_ of 35.9 nM. The inhibition profiles of the coumarin derivatives **34–39**, **42–45** were in line with previous evidence of this class of CAIs^35^. In detail, inhibition of hCAs I and II was detected below 100 µM for none of the lactone compounds, whilst all of them were low-medium nanomolar inhibitors of the target hCAs IX and XII, with K_I_s in the ranges 9.6–66.0 nM and 4.2–30.1 nM, respectively. The effect of spacer length at the triazole sides over the K_I_ values is clear for the prodrug coumarin subset, but a neat SAR cannot be ruled out. In fact, elongation of the spacer between the triazole and coumarin scaffold appears to increase the inhibitory action more than the extension of the berberine-triazole spacer, as observed with compounds **43** (**C-1,5**) with hCA XII (K_I_ of 4.2 nM) and **44** (**C-1,6**) with hCA IX (K_I_ of 9.6 nM). Nonetheless, the shortest coumarin derivative **45** (**C-1,0c**) produced a hCA XII inhibition comparable with **43** (**C-1,5**) (K_I_ of 5.4 nM). With the only exception of derivative **47**, all derivatives showed marked target/off target isoform selectivity (Table S1, Supporting Information), unlike the reference drug **AAZ** and prevalently above the lead **SLC-0111**. Whilst the selectivity indexes (*SI)* of the coumarin derivatives for the target hCAs over hCA I were comparable or slightly greater in the case of hCA XII (151.5–1041 vs 332.2–2381), the sulphonamide derivatives selectively targeted hCA IX over hCA I at a greater extent (307.1–2390) and hCA II (8.5–112.4), with the subset **29–31** being of great interest since displaying *SI* > 1000 and *SI* > 100, respectively. Instead, these zinc binder compounds generally showed a great hCA XII/hCA I selectivity (25.8–213.7), while only sulphonamides **26**, **31** and **33** reported notable *SI* for hCA XII over hCA II (8.1, 9.9, 14.2). Hence, as a general tendency, coumarin derivatives stood out for isoform selectivity, whilst the sulphonamides here reported demonstrated almost unprecedented inhibition potency associated with medium to high selectivity of action.

### Circular dichroism (CD) experiments

The 21 synthesised MTDLs were screened for their ability to bind to and stabilise DNA GQ-forming sequences, compared to berberine, by CD experiments^50^. Indeed, circular dichroism has shown to be outstandingly useful and informative for noncanonical nucleic acid secondary structures^51^. For this purpose, a G-rich 23-nucleotide truncation (23-nt) of the human telomeric sequence (*Tel_23_*) and two G-rich motifs from the promoter regions of c-KIT (*c-Kit1*) and c-MYC (*c-Myc*) oncogenes were employed. First, the overall topology adopted by each DNA sequence in K^+^-containing buffer was verified by CD spectra. *Tel_23_* GQ exhibited a CD spectrum with two positive bands at 290 and 270 nm, and a weak negative band at 240 nm, consistent with a hybrid [3 + 1] GQ conformation (Figure S1, Supporting Information). On the other hand, *c-Kit1 and c-Myc* sequences adopted a parallel GQ conformation, showing a distinctive positive band at around 260 nm and a negative one at ∼240 nm in their CD spectra (Figures S2 and S3, Supporting Information). Additional CD spectra of the investigated DNAs in the presence of the 21 MTDLs and berberine revealed no relevant changes in the DNA chiroptical properties, thus suggesting an overall retention of GQ structures in their presence (Figures S1–S3, Supporting Information).

Afterwards, the GQ-stabilising properties of the molecules were assessed by CD melting experiments, measuring the differences in the apparent melting temperatures of the DNA in the presence and absence of compounds (Δ*T*_1/2_) (Figures S4–S6 and Table S2, Supporting Information). Results showed that the MTDLs were generally more effective than berberine in stabilising the parallel GQ structures adopted by *c-Kit1* and *c-Myc* over the hybrid *Tel_23_* (Table 2), suggesting that hybridisation of berberine with CAI portions enhances the interaction with parallel GQs. This could be explained by considering the higher accessibility of the external G-tetrads in the case of parallel GQs compared to the hybrid one^52^, which would allow the additional CAI moiety of conjugated compounds to be better accommodated in the binding site. Both sulphonamide and coumarin derivatives effectively stabilised *c-Kit1* GQ, with the greatest increases over berberine shown by **S-2,0**, **S-4,0**, and **S-5,0** (ΔΔ*T*_1/2_ ≥ 6.0 °C), belonging to the sulphonamide series, and by the coumarin derivatives **C-3,2** and **C-5,2** (ΔΔ*T*_1/2_ = 7.0 °C in both cases). As far as *c-Myc* GQ is concerned, it was strongly stabilised by coumarin derivatives **C-1,0c**, **C-1,6**, and **C-5,2** (ΔΔ*T*_1/2_ > 13.0 °C compared to berberine), while the most effective compounds among the sulphonamide derivatives were **S-1,0*m***, **S-2,0**, **S-3,0**, and **S-4,0** (ΔΔ*T*_1/2_ > 13.0 °C compared to berberine). On the other hand, the stabilising properties of the MTDLs on *Tel_23_*GQ were only in some cases better than berberine, *i.e.* **C-3,4** among the coumarin derivatives (ΔΔ*T*_1/2_ = 3.0 °C for **C-3,4**), and **S-5,0** among the sulphonamide derivatives (ΔΔ*T*_1/2_ = 2.5 °C). In other cases, the conjugates showed equal or minor stabilising effects compared to berberine.

**Table 2.** Changes in the G4-stabilising effect (ΔΔ*T*_1/2_) of the indicated compounds with respect to berberine, as determined by circular dichroism melting experiments.

Compound		ΔΔ*T*_1/2_ (°C)^a^		
		*Tel_23_*	*c-Kit1*	*c-Myc*
**26**	**S-2,0**	0.5	9.0	>13.0
**27**	**S-2,2**	−2.0	5.5	6.5
**28**	**S-3,0**	−3.5	3.5	>13.0
**29**	**S-3,2**	−1.5	3.0	4.5
**30**	**S-4,0**	−0.5	7.5	>13.0
**31**	**S-4,2**	−1.5	2.5	1.0
**32**	**S-5,0**	2.5	6.0	1.0
**33**	**S-5,2**	1.0	5.5	0.5
**40**	**S-1,0*m***	−4.0	3.0	>13.0
**41**	**S-1,0*p***	−4.0	2.0	2.5
**47**	**S-3,a**	−3.0	2.5	0.5
**34**	**C-3,2**	1.0	7.0	6.0
**35**	**C-3,3**	−1.5	5.5	6.5
**36**	**C-3,4**	3.0	4.0	3.0
**37**	**C-4,2**	−0.5	4.5	1.0
**38**	**C-4,3**	−2.0	4.5	3.0
**39**	**C-5,2**	−1.0	7.0	>13.0
**42**	**C-1,4**	1.0	4.0	4.0
**43**	**C-1,5**	−2.5	3.5	2.0
**44**	**C-1,6**	−2.0	4.5	>13.0
**45**	**C-1,0c**	−2.5	3.5	>13.0

^a^ ΔΔ*T*_1/2_ values are the difference between Δ*T*_1/2_ induced by the MTDL compounds and those induced by berberine.

To evaluate the possible binding and stabilisation of double-stranded DNA, a 27-nt long hairpin-forming sequence (*Hairpin*) was employed. The CD spectrum of *Hairpin* typically shows a positive band at around 280 nm and a negative one at ∼250 nm (Figure S7, Supporting Information). These bands were not significantly altered after the addition of MTDLs or berberine. Moreover, most compounds did not affect *Hairpin* thermal stability (Δ*T*_1/2_ ≤ 2.5 °C), indicating their preference for GQ over hairpin DNA duplex (Figure S8 and Table S2, Supporting Information).

### Förster resonance energy transfer (FRET) melting experiments

FRET melting analysis was used to further estimate the GQ over duplex selectivity of the selected compounds^53^. The GQ-forming oligonucleotide *c-Kit1* labelled with FAM and TAMRA (*F-c-Kit1-T*) was employed because, among the parallel GQs used in this study (*c-Kit1* and *c-Myc*), it was the only one with a lower *T*_1/2_ than the hairpin DNA duplex competitor (*Hairpin*), thus allowing proper estimation of the GQ-stabilising properties of the ligands in the presence of a large excess of the unlabelled (non-fluorescent) competitor. Therefore, the MTDLs that were found to be the best *c-Kit1* stabilisers by CD experiments (*i.e.* **S-2,0**, **S-4,0**, **S-5,0**, **C-3,2**, and **C-5,2**), as well as the most biologically active derivative (**C-4,2**) were evaluated for their ability to bind and stabilise the GQ even in the presence of an excess of *Hairpin* in solution. The sulphonamide **S-3,2** was also considered since the crystal structure of such MTDL in complex with *Tel_23_* was successfully obtained. Results of these experiments (Figure S9, Supporting Information and Table 3) confirmed that these compounds are efficient GQ stabilisers. Moreover, their stabilising effects were not significantly affected by the presence of the competitor (at 15 and 50 molar equiv with respect to the GQ), thus confirming their selective GQ binding.

**Table 3.** Compound-induced thermal stabilisation of the dual-labelled *F-c-Kit1-T* GQ measured by FRET melting competition experiments.

Compound		Δ*T*_1/2_ (°C)^a^		
		*F-c-Kit1-T*	*F-c-Kit1-T + Hairpin* (1:15)	*F-c-Kit1-T + Hairpin* (1:50)
**26**	**S-2,0**	12.4	10.2	12.4
**29**	**S-3,2**	13.2	12.8	13.2
**30**	**S-4,0**	13.6	13.8	12.6
**32**	**S-5,0**	14.4	15.0	14.6
**34**	**C-3,2**	10.6	11.4	11.2
**37**	**C-4,2**	12.0	12.8	12.6
**39**	**C-5,2**	14.6	13.2	14.0

^a^ Δ*T*_1/2_ values are the difference between the *T*_1/2_ of *F-c-Kit1-T* in the presence (2 molar equiv) and absence of ligands, without or with large excesses of unlabelled *Hairpin* (15 and 50 molar equiv with respect to GQ). The *T*_1/2_ of *F-c-Kit1-T* is 57.5 (±0.2) °C. All experiments were performed at least in duplicate, and the reported values are the average of the measurements. Errors in the Δ*T*_1/2_ values are within ± 0.5 °C.

### Crystallographic study

**Figure 5.**
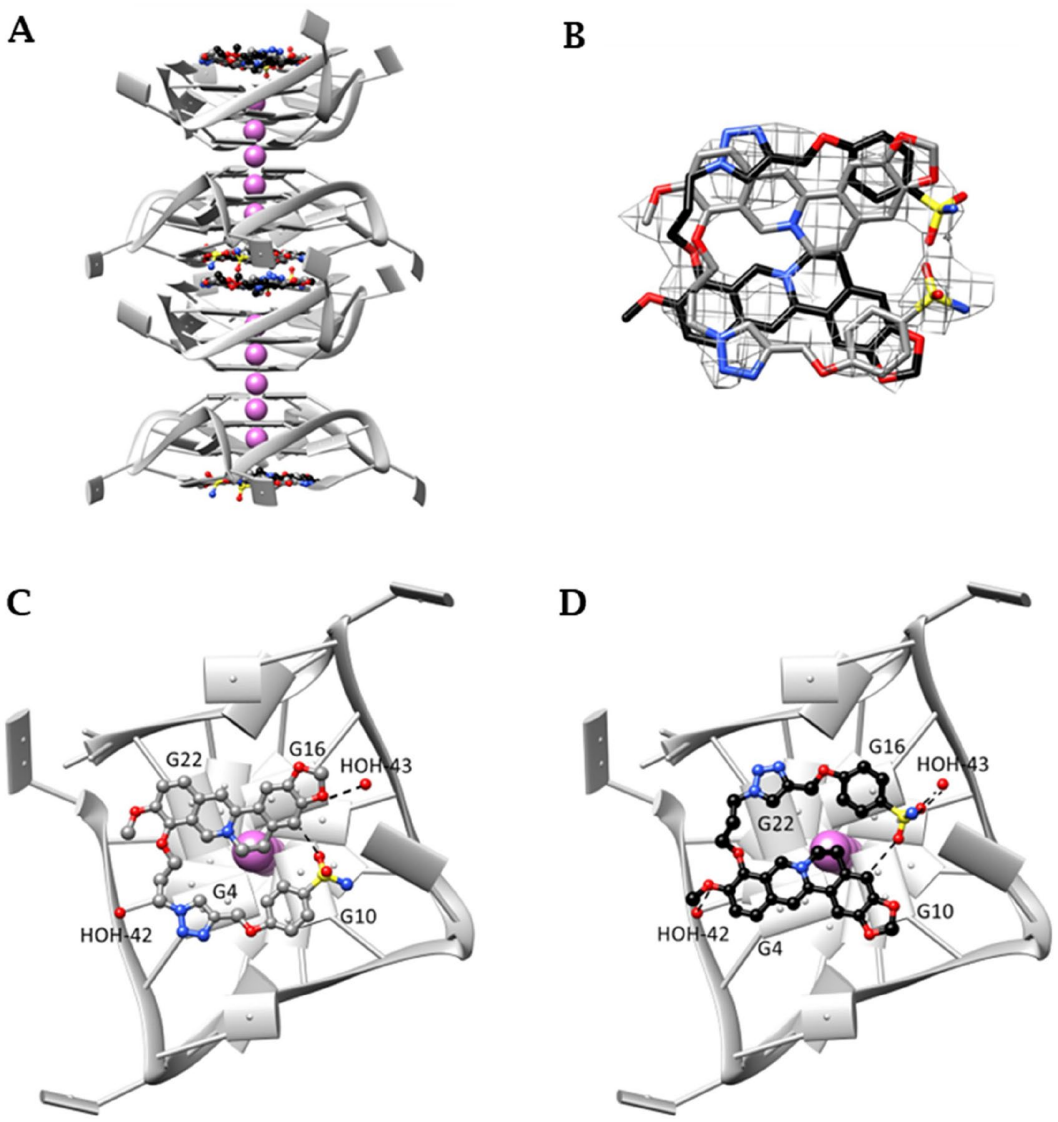
(A) Monomolecular parallel GQs arranged in columns growing along the c axis. (B) OMIT electron density map contoured at 2σ level of **S-3,2** (**29**) and skeleton of the ligand in the two disordered orientations—gray (*Y* orientation) and black (*Z* orientation); (C) and (D) interaction detail with GQ: (C) *Y* orientation; (D) *Z* orientation (T11 and T12 nucleic base atoms not shown). Final coordinates and structure factors (Table S3, Supporting Information) have been deposited with the Protein Data Bank (PDB accession number 7PNL).

Crystals suitable for X-ray diffraction analysis were obtained for the human telomeric DNA sequence *Tel_23_* in complex with the derivative **S-3,2** (**29**). The monomolecular DNA GQ is arranged in columns growing along the c-axis (Figure 5A) and assumes the expected all-parallel conformation. In each column, symmetry-related GQ units are in stacking contact through their 5′-end G-tetrads, while the 3′-end G-tetrads constitute the binding sites. The dimers are stabilised by an additional potassium ion located between the 5′-end G-tetrads, an arrangement previously reported by several authors^54–61^. Contiguous 3′-ends G-tetrads lie about 10 Å apart from one another defining a cavity big enough to host two symmetry-related ligand molecules. Each one is in contact with a 3′-end G-tetrad, and it is found to be disordered and spread over two positions, as shown in Figure 5(A,B). Figure 5(C,D) show the details of the interactions given by **S-3,2** (**29**) in each single orientation adopted in the binding site, in the following indicated as *Y* (Figure 5C) and *Z* structure (Figure 5D). In both orientations the berberine core is almost planar and points the ethylenic chain towards the centre of the G-tetrad. This arrangement has never been previously reported for berberine and berberine derivatized at C13 in complex with the human telomeric GQ^61–63^, and it is most likely induced by the functionalization at the C9 position. Notably, similar placements of berberine have been already found in the NMR solution structures of the PDGFR-β and MYC promoter sequences^62^^,^^63^, where the berberine ethylenic hydrogens interact with a flanking purine residue partially capping the G-tetrad. Instead, in the propeller folding this interaction is lacking, but the three-carbon spacer connecting the oxygen and the triazole nitrogen of **S-3,2** (**29**) has the right length to place the triazole and the benzenesulfonamide rings at stacking distance over the guanines. In the *Y* structure (Figure 5C) the berberine quinoline and benzo groups are at stacking distance from G22 and G16 (interplanar distances about 3.4 Å and 3.5 Å, respectively), while the triazole and the benzenesulfonamide rings are placed over the guanines G4 (3.6 Å) and G10 (3.7 Å). In this way, one of the sulphonamide oxygens gives a strong non-classic CH···O H-bond interaction with the berberine benzene hydrogen atom (C^…^O distance 2.7 Å). In the *Z* structure (Figure 5D), the orientation of the molecule is flipped with respect to the *Y* orientation, and the G4 (interplanar distance 3.3 Å, quinoline groups’ centroids distance 3.6 Å), G10 (interplanar distance 3.3 Å, benzene groups’ centroids distance 3.9 Å) G22 (triazole ring – interplanar distance 3.4 Å) and G16 residues (benzenesulfonamide ring – interplanar distance 3.4 Å) are the most interacting. The intramolecular CH^…^O interaction is still present (C^…^O distance 3.2 Å). As shown in Figure 5, the three-carbon spacer is always placed on the same side of the G-tetrad, in the proximity of guanines G4 and G22. Apparently, no contacts between the nucleic acid and the sulphonamide group are formed, but it cannot be excluded that water-bridged interactions can be established. The amide nitrogen in the *Y* structure is at about 5 Å from a T11 phosphate oxygen. Moreover, two water molecules are in direct contact with **S-3,2** (**29**): HOH-43 is at 2.9 Å with one of the benzodioxole oxygens in *Y* and at 2.7 Å from both the nitrogen and one oxygen of the sulphonamide in *Z*, while HOH-42 gives a H-bond with the methoxy oxygen of *Z*.

As a whole, both length and position of berberine functionalization help to maximise the overall binding strength of **S-3,2** (**29**) towards the human telomeric GQ, even though berberine ethylenic chain, which is used to give contact with capping residues in the PDGFR-β and MYC promoter GQs^63^, is now completely shielded by the benzenesulfonamide arm.

Noticeably, we can suppose that the most effective GQ-stabilising sulphonamide derivatives can bind telomeric GQ similarly to what was observed for **S-3,2** (**29**). If this is true, longer chains on the sulphonamide side would negatively impact the binding more than an analogous elongation on the berberine side. Directly connected triazole and phenyl rings ensure a wide conjugated electron density needed for effective π-π stacking with the G-tetrad.

### Cell experiments in normoxia

#### MTT assay

Combining the results on carbonic anhydrase inhibition and GQ thermal stabilisation, we selected the sulphonamides **S-2,0**, **S-4,0**, **S-5,0** and the coumarins **C-3,2**, **C-4,2**, **C-5,2** as the most promising MTDLs to be further investigated, since able to both efficiently suppress the activity of the hCAs IX/XII and stabilise GQ structures, *in vitro*. Moreover, given that crystals suitable for X-ray diffraction analyses had been successfully obtained for the human telomeric DNA GQ in complex with **S-3,2**, we also included such sulphonamide derivative in the subsequent studies. Thus, we assessed the cytotoxicity of the selected MTDLs on CA IX-positive HeLa cells^38^, using the MTT assay^64^. As shown in Figures S10 and S11, Supporting Information, the viability of HeLa cells treated for 48 h with **S-2,0**, **S-3,2**, **S-4,0** and **C-3,2**, **C-4,2**, **C-5,2** decreased in a concentration-dependent fashion, whereas the cytotoxic effect of **S-5,0** (around 40% reduction in the percentage of cell survival) did not vary while increasing its concentration up to 200 µM.

Overall, conjugates **C-4,2** and **C-5,2** turned out to be the most cytotoxic coumarins, with IC_50_ values of 40.0 and 37.5 µM, respectively (Table 4). Of note, the longer the linker connecting the berberine nucleus and the triazole scaffold, the higher the cytotoxicity of the investigated coumarin MTDLs on HeLa cancer cells, suggesting a chain of at least five carbon atoms as the optimal linker. On the other hand, the four-carbon aliphatic spacer in the structure of **S-4,0** ensured the strongest reduction in tumour cell viability among the tested sulphonamides (IC_50_ = 35.0 µM). Lastly, as for **S-2,0**, **S-3,2**, **S-5,0**, and **C-3,2**, their IC_50_ values fell outside the explored range of concentrations, indicating their lower cytotoxic effect on HeLa cells, under the experimental conditions.

**Table 4.** Concentrations of the investigated MTDLs required to give 50% of cell viability reduction (IC_50_ values), by means of the MTT assay.

Compound		IC_50_ (µM)	95% confidence interval (µM)
**26**	**S-2,0**	>200	–
**29**	**S-3,2**	>200	–
**30**	**S-4,0**	35.0	27.3–45.5
**32**	**S-5,0**	>200	–
**34**	**C-3,2**	>50	–
**37**	**C-4,2**	40.0	38.0–46.0
**39**	**C-5,2**	37.5	28.6–53.0

Afterwards, we dissected whether the most cytotoxic multi-target conjugates (**S-4,0**, **C-4,2**, and **C-5,2**) showed a synergistic cytotoxic effect over the single-target-directed agents (Figure S12, Supporting Information). To this aim, HeLa cells were treated for 48 h with increasing concentrations of **S** (the CAI moiety of **S-4,0**), **C** (the CAI scaffold of **C-4,2** and **C-5,2**), or berberine (the GQ ligand counterpart in each of the three MTDLs). As highlighted in Figure S13, Supporting Information, **S** and **C** only slightly reduced HeLa cell viability, whereas berberine displayed a good dose-dependent cytotoxic effect, in line with previous findings^65^^,^^66^. Nevertheless, the IC_50_ value of berberine (Table S4, Supporting Information) proved to be considerably higher than those of the MTDLs, suggesting that derivatization of berberine at 9-position with some triazole-based substituents could improve its anticancer activity, as for other berberine derivatives reported elsewhere^67^.

#### Immunofluorescence studies

**Figure 6.**
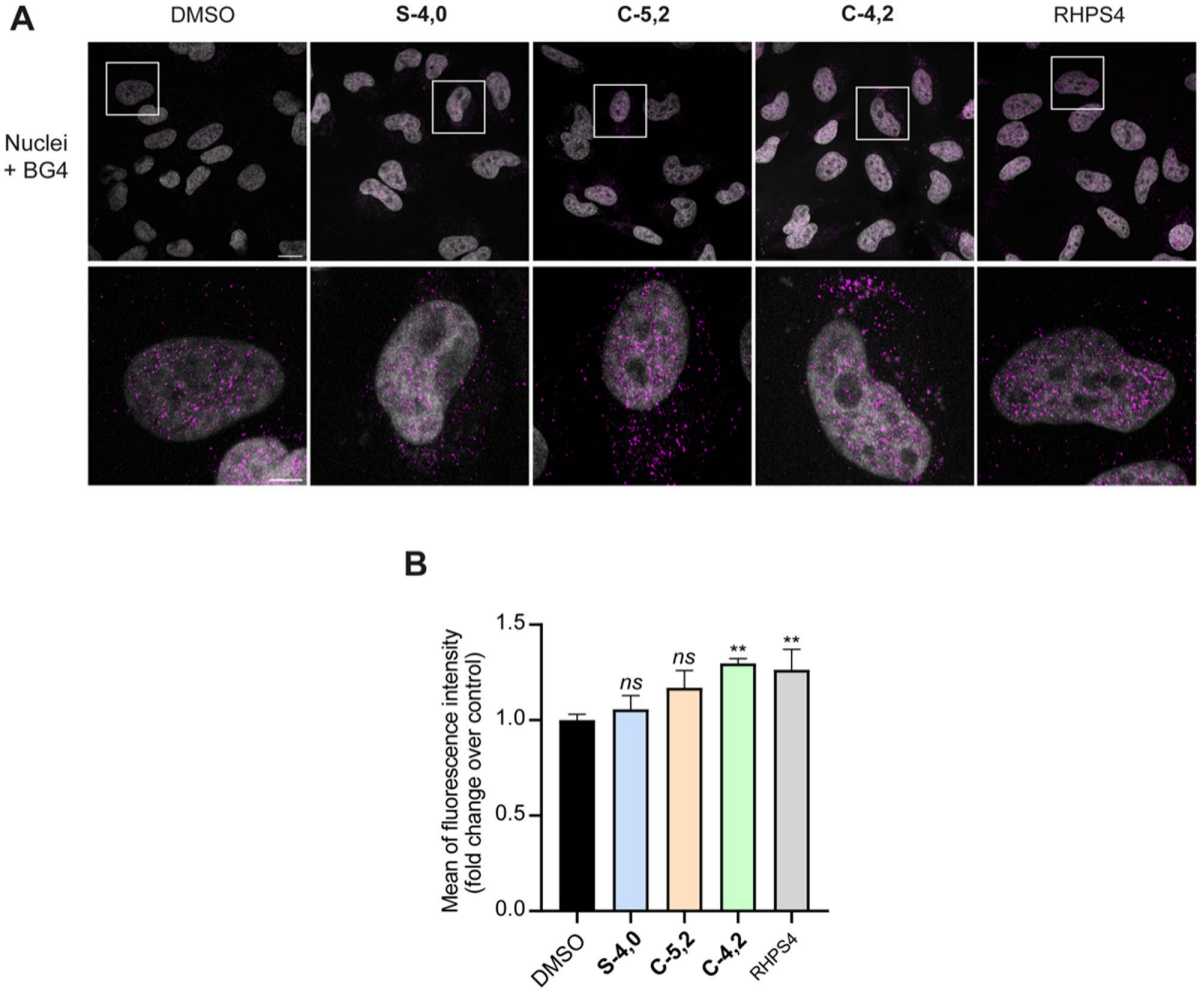
(A) Representative fields showing G4 foci formation detected by immunofluorescence in HeLa cells treated for 24 h with 35.0 µM **S-4,0**, 40.0 µM **C-4,2**, and 37.5 µM **C-5,2** or an equivalent amount of vehicle (0.1% DMSO). As a positive control, cells were treated with 2 µM RHPS4 (a well-established GQ ligand)^68^ for 24 h. Scale bar: 10 µm. Upper panels: the merged channels of BG4-stained GQ structures (magenta) and Hoechst-counterstained nuclei (grey) are reported. Lower panels: enlargements from the pictures in the upper panels. (B) Quantitative analysis of the fluorescence intensity of the total number of GQ foci. An average of 50 cells were screened for each condition and the results are expressed as a fold change of fluorescence intensity (anti-GQ signal) over the negative control (DMSO). Histograms show the mean ± SD of three independent experiments. The statistical significance was calculated using a one-way ANOVA test on GraphPad Prism 8.0.2 (*ns*: not significant, **: *p* < 0.01).

After measuring the viability of HeLa cells upon treatment with the newly synthesised MTDLs mentioned above, we probed the capability of the three most cytotoxic ones (**S-4,0**, **C-4,2**, and **C-5,2**) to affect the formation of GQ structures by immunofluorescence (IF) microscopy, using the BG4 antibody that selectively recognises the GQ structures^47^. As it can be noticed in Figure 6, the fluorescence intensity of the total number of GQ foci increased by around 30% after treatment with both the coumarin derivative **C-4,2** and the reference GQ binder RHPS4, whereas it was not affected by **S-4,0** and **C-5,2**. These results suggest that the multi-target-directed ligand **C-4,2** could bind and stabilise GQ structures also in the cellular context, confirming its GQ-stabilising properties already observed at the biophysical level. On the contrary, the levels of GQ structures were influenced neither by the treatment with the sulphonamide **S-4,0** nor by the coumarin **C-5,2**, despite their ability to thermally stabilise some GQ-forming sequences *in vitro*.

To sum up, while the results of the biophysical studies on **S-4,0** and **C-5,2** could not find confirmation at the biological level, the coumarin **C-4,2** proved to be able to affect the formation of GQ structures also in the complexity of a cellular model, in a way which could be exquisitely ascribed to the characteristics and features of the conjugate itself.

### Cell experiments in hypoxia

#### MTT assay

Taking into consideration the good cytotoxicity of **C-4,2** on cancer cells and its ability to stabilise GQ structures also in the cellular environment, we decided to further evaluate its antitumor potential by testing it under hypoxic conditions, when expression of high amounts of CA IX occurs^69^. To this aim, HeLa cells were pre-treated for 60 h with a non-toxic concentration of cobalt(II) chloride hexahydrate (CoCl_2_, 50 μM, Figure S14, Supporting Information), which was employed as the chemical inducer of HIF-1α to establish hypoxia^70^^,^^71^. After that, HeLa were exposed to increasing concentrations of **C-4,2** or vehicle, for the following 12 h, still in the presence of CoCl_2_. As a comparison, HeLa cells were kept for 60 h in the presence of normal oxygen levels and then treated with the investigated MTDL or vehicle for the following 12 h. According to the results (Figure S15A, Supporting Information), the cytotoxicity of the coumarin **C-4,2** proved to be more pronounced under hypoxic conditions (IC_50_ = 26.5 µM; 20.9–32.8 µM, 95% confidence interval) than in normoxia (IC_50_ = 46.0 µM; 41.9–49.7 µM, 95% confidence interval), in line with the overexpression of the target hCA IX as a result of hypoxia.

Importantly, the cytotoxicity of the multi-target-directed ligand **C-4,2** turned out to be greater than that resulting from the co-exposure to equimolar concentrations of its single structural components (berberine and **C**), both in normoxia and hypoxia (Figure S15B, Supporting Information). Such a stronger cytotoxic effect of the MTDL over the combined treatment might be ascribed to the exploitation of additional cellular uptake systems, ameliorated physicochemical properties, and improved pharmacokinetics of the MTDL itself, which can reach the targets as a unique molecular entity.

#### Immunofluorescence studies

**Figure 7.**
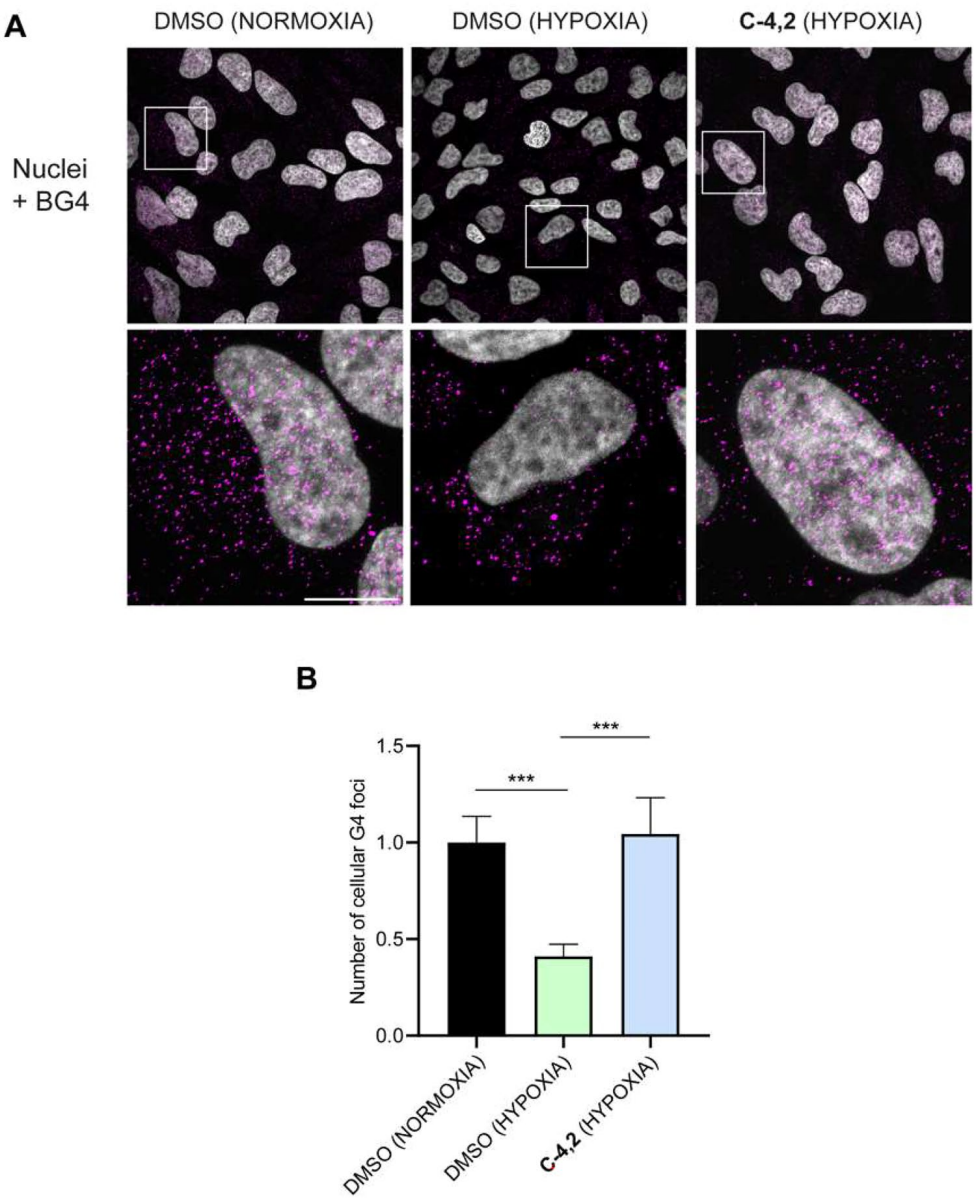
(A) Representative fields showing G4 foci formation detected by immunofluorescence in HeLa cells pre-treated with 50 μM cobalt (II) chloride hexahydrate for 60 h to induce hypoxia and then exposed to 1 µM **C-4,2** or vehicle (0.1% DMSO) for the following 12 h, still in the presence of CoCl_2_. DMSO-treated normoxic HeLa cells are also shown. Scale bar: 10 µm. Upper panels: the merged channels of BG4-stained GQ structures (magenta) and Hoechst-counterstained nuclei (grey) are reported. Lower panels: enlargements from the pictures in the upper panels. (B) Quantification of the cellular BG4 foci in DMSO-treated normoxic HeLa cells, DMSO-treated hypoxic HeLa cells, and **C-4,2**-treated hypoxic HeLa cells. An average of 50 cells were screened for each condition. The number of GQ foci has been normalised to the mean of the corresponding control sample (DMSO). Histograms show the mean ± SD of three independent experiments. The statistical significance was calculated using a one-way ANOVA test on GraphPad Prism 8.0.2 (**: *p* < 0.01, ****: *p* < 0.0001).

Finally, we investigated **C-4,2** capability to impact the formation of GQ structures even in hypoxic HeLa cells. As highlighted in Figure 7, hypoxia per se significantly reduced the number of cellular GQ structures^72^, in line with the hypoxia-associated chromatin compaction^73^^,^^74^. Interestingly, the addition of the MTDL **C-4,2** under hypoxic conditions resulted in a remarkable increase in the number of detectable GQs, supporting the ability of the coumarin derivative to stabilise GQs also in a hypoxic microenvironment, protecting such structures from unfolding in hypoxia.

## Materials and methods

### Chemistry

Solvents and reagents were purchased from Merck, Fluorochem, and TCI. All reactions involving air- or moisture-sensitive compounds were performed under a nitrogen atmosphere, using dried glassware and syringes techniques to transfer solutions. Nuclear magnetic resonance (^1^H-NMR, ^13^C-NMR) spectra were recorded using a Bruker Advance III 400 MHz spectrometer in DMSO-d_6_ or CDCl_3_. Chemical shifts are reported in parts per million (ppm), and the coupling constants (*J*) are expressed in hertz (Hz). Splitting patterns are designated as follows: s, singlet; d, doublet; t, triplet; q, quadruplet; m, multiplet; brs, broad singlet; dd, double of doublets. The assignment of exchangeable protons was confirmed by the addition of D_2_O. Analytical thin-layer chromatography (TLC) was carried out on Merck silica gel F-254 plates. Flash chromatography purifications were performed on Merck silica gel 60 (230–400 mesh ASTM) as the stationary phase and EtOAc/n-hexane or MeOH/DCM was used as eluents. Melting points (mp) were measured in open capillary tubes with a Gallenkamp MPD350.BM3.5 apparatus and are uncorrected. HRMS analysis confirmed the molecular formula of each analyte with a mass error lower than 2 ppm: a mother solution of each analyte was prepared at 1 mg/mL in methanol containing 10% DMSO. A 20 ng/µL solution was then prepared diluting the mother solution in ACN/water containing 0.1% formic acid and was infused in the ESI interface of the high-resolution mass spectrometer. The solution of each analyte was analysed using a Thermo Scientific LTQ Orbitrap mass spectrometer equipped with an IonMax Electrospray interface. The high-resolution mass spectrometer operated in positive ion mode at 60 000 mass resolution (at 400 m/z). Source and interface parameters were optimised on the protonated ion of each analyte: ESI voltage was 4 kV, capillary voltage 13 V, tube lens voltage 60 V; capillary temperature was 290 °C. Sheath, auxiliary and sweep gas was nitrogen, set at 15, 8, and 0 (arbitrary units), respectively. The solution was infused at 7 µL/min. The analyte solutions were also analysed by HPLC-MS using a Thermo Scientific instrument composed of an Ultimate 3000 HPLC coupled to the LTQ Orbitrap mass spectrometer. The column was a Phenomenex Kinetex EVO C18 (100 × 2.1 mm, 5 µm) operating at 250 µL/min and thermostated at 25 °C. The eluents were water (phase A) and ACN (phase B), both containing 0.1% formic acid. The injection volume was 20 µL. The HPLC injector sample plate was thermostated at 10 (±2) °C. The gradient elution program was as follows: time 0 min 7% B, maintained for 1 min, then a linear gradient to 90% B in 9.0 min, maintained for 2 min; thus, initial conditions were restored and left equilibrating for 8 min. The column exit was directly connected to the ESI interface operating with the settings applied for HRMS. Sheath gas, auxiliary gas and sweep gas were at 25, 18, and 5 (arbitrary units), respectively. Low-resolution mass spectra were recorded in positive ion mode using the linear quadrupole ion trap (LTQ), from m/z 160 to m/z 1000. Max ion injection time was set at 50 msec. All hybrid compounds are >95% pure by HPLC analysis. The HPLC traces of compounds tested in cell are reported in the Supporting Information. Intermediates **11**, **12**, **16**, and **25** were obtained as previously described^45^^,^^75^.

#### Synthesis of 10-methoxy-5,6-dihydro-9H-[1,3]dioxolo[4,5-g]isoquinolino[3,2-a]isoquinolin-9-one 2

Berberine hydrochloride **1** (5.0 g) was heated at 190 °C under *vacuum* (20–30 mmHg) for 2h to obtain berberrubine **2** as a dark red solid; 90% yield; m.p. >300 °C; silica gel TLC *R_f_*0.25 (MeOH/DCM 10% *v/v)*; δ_H_ (400 MHz, DMSO-*d*_6_): 9.11 (s, 1H, Ar-*H*), 8.02 (s, 1H, Ar-*H*), 7.65 (s, 1H, Ar-*H*), 7.24 (d, *J* = 7.8 Hz, 1H, Ar-*H*), 7.00 (s, 1H, Ar-*H*), 6.38 (d, *J* = 7.8 Hz, 1H, Ar-*H*), 6.13 (s, 2H, C*H*_2_), 4.51 (t, *J* = 5.9 Hz, 2H, C*H*_2_), 3.76 (s, 3H, C*H*_3_), 3.07 (t, *J* = 5.9 Hz, 2H, C*H*_2_); δ_C_ (100 MHz, DMSO-*d_6_*): 162.70, 149.63, 149.48, 148.02, 145.84, 134.03, 132.25, 129.29, 122.52, 121.42, 120.15, 118.23, 107.75, 106.92, 104.35, 101.84, 55.20, 54.13, 27.57; m/z (ESI positive) 322.1 [M + H]^+^.

### General procedure for the synthesis of berberine azide intermediates 3–6

The dibromoalkane (20.0 eq) was added to a solution of berberrubine **2** (1.0 g, 1.0 eq) in dry DMF (10 mL) and the reaction mixture was heated at 60 °C o.n. The resulting suspension was treated with Et_2_O, and the obtained precipitate was filtered. The crude (bromoalkyl)berberine was dissolved in dry DMF (5 mL) and NaN_3_ (10.0 eq) was added. The mixture was stirred at 60 °C o.n., quenched with water and extracted with DCM (2 × 30 mL). The combined organic layers were dried over anhydrous Na_2_SO_4_, filtered-off, concentrated *in vacuo* and triturated with Et_2_O to give the title compounds as red powders.

#### 9–(2-Azidoethoxy)-10-methoxy-5,6-dihydro-[1,3]dioxolo[4,5-g]isoquinolino[3,2-a]isoquinolin-7-ium bromide 3

72% yield; m.p. 215–217 °C; silica gel TLC *R_f_*0.50 (MeOH/DCM 10% *v/v)*; δ_H_ (400 MHz, DMSO-*d_6_*): 9.85 (s, 1H, Ar-*H*), 8.98 (s, 1H, Ar-*H*), 8.25 (d, *J* = 9.2 Hz, 1H, Ar-*H*), 8.05 (d, *J* = 9.2 Hz, 1H, Ar-*H*), 7.84 (s, 1H, Ar-*H*), 7.13 (s, 1H, Ar-*H*), 6.21 (s, 2H, C*H*_2_), 4.97 (t, *J* = 6.1 Hz, 2H, C*H*_2_), 4.48 (m, 2H, C*H*_2_), 4.11 (s, 3H, C*H*_3_), 3.88 (m, 2H, C*H*_2_), 3.25 (m, 2H, C*H*_2_); δ_C_ (100 MHz, DMSO-*d_6_*): 151.25, 150.79, 148.62, 146.15, 143.03, 138.46, 133.95, 131.58, 127.53, 124.77, 122.42, 121.32, 121.17, 109.34, 106.36, 103.02, 73.48, 58.02, 56.32, 51.50, 27.28; m/z (ESI positive) 391.0 [M]^+^.

#### 9–(3-Azidopropoxy)-10-methoxy-5,6-dihydro-[1,3]dioxolo[4,5-g]isoquinolino[3,2-a]isoquinolin-7-ium bromide 4

79% yield; m.p. 239–242 °C; silica gel TLC *R_f_*0.50 (MeOH/DCM 10% *v/v)*; δ_H_ (400 MHz, DMSO-*d_6_*): 9.84 (s, 1H, Ar-*H*), 8.99 (s, 1H, Ar-*H*), 8.25 (d, *J* = 9.1 Hz, 1H, Ar-*H*), 8.05 (d, *J* = 9.1 Hz, 1H, Ar-*H*), 7.84 (s, 1H, Ar-*H*), 7.14 (s, 1H, Ar-*H*), 6.22 (s, 2H, C*H*_2_), 4.99 (t, *J* = 5.9 Hz, 2H, C*H*_2_), 4.41 (t, *J* = 6.2 Hz, 2H, C*H*_2_), 4.11 (s, 3H, C*H*_2_), 3.71 (t, *J* = 6.7 Hz, 2H, C*H*_2_), 3.26 (t, *J* = 6.0 Hz, 2H, C*H*_2_), 2.19 (m, 2H, C*H*_2_); δ_C_ (100 MHz, DMSO-*d_6_*): 151.25, 150.78, 148.63, 146.20, 143.49, 138.44, 133.98, 131.63, 127.62, 124.47, 122.46, 121.37, 121.16, 109.35, 106.38, 103.03, 72.42, 58.04, 56.24, 48.72, 29.97, 27.27; m/z (ESI positive) 405.1 [M]^+^.

#### 9–(4-Azidobutoxy)-10-methoxy-5,6-dihydro-[1,3]dioxolo[4,5-g]isoquinolino[3,2-a]isoquinolin-7-ium bromide 5

72% yield; m.p. 216–218 °C; silica gel TLC *R_f_* 0.50 (MeOH/DCM 10% *v/v)*; δ_H_ (400 MHz, DMSO-*d_6_*): 9.80 (s, 1H, Ar-*H*), 8.97 (s, 1H, Ar-*H*), 8.23 (d, *J* = 9.2 Hz, 1H, Ar-*H*), 8.03 (d, *J* = 9.2 Hz, 1H, Ar-*H*), 7.83 (s, 1H, Ar-*H*), 7.12 (s, 1H, Ar-*H*), 6.21 (s, 2H, C*H*_2_), 4.98 (t, *J* = 6.1 Hz, 2H, C*H*_2_), 4.34 (t, *J* = 6.4 Hz, 2H, C*H*_2_), 4.09 (s, 3H, C*H*_3_), 3.50 (t, *J* = 6.8 Hz, 2H, C*H*_2_), 3.24 (m, 2H, C*H*_2_), 1.96 (dd, *J* = 14.7, 6.5 Hz, 2H, C*H*_2_), 1.84 (m, 2H, C*H*_2_); δ_C_ (100 MHz, DMSO-*d_6_*): 151.32, 150.77, 148.62, 146.21, 143.70, 138.39, 133.98, 131.60, 127.62, 124.34, 122.55, 121.37, 121.16, 109.34, 106.36, 103.02, 74.68, 58.00, 56.25, 51.45, 27.75, 27.29, 25.87; m/z (ESI positive) 419.1 [M]^+^.

#### 9-((5-Azidopentyl)oxy)-10-methoxy-5,6-dihydro-[1,3]dioxolo[4,5-g]isoquinolino[3,2-a]isoquinolin-7-ium bromide 6

72% yield; m.p. 219–222 °C; silica gel TLC *R_f_*0.35 (MeOH/DCM 10% *v/v)*; δ_H_ (400 MHz, DMSO-*d_6_*): 9.80 (s, 1H, Ar-*H*), 8.98 (s, 1H, Ar-*H*), 8.24 (d, *J* = 9.2 Hz, 1H, Ar-*H*), 8.03 (d, *J* = 9.2 Hz, 1H, Ar-*H*), 7.84 (s, 1H, Ar-*H*), 7.13 (s, 1H, Ar-*H*), 6.21 (s, 2H, C*H*_2_), 4.99 (t, *J* = 6.1 Hz, 2H, C*H*_2_), 4.33 (t, *J* = 6.6 Hz, 2H, C*H*_2_), 4.09 (s, 3H, C*H*_3_), 3.43 (t, *J* = 6.7 Hz, 2H, C*H*_2_), 1.95 (m, 2H, C*H*_2_), 1.69 (m, 4H, 2 × C*H*_2_), 1.60 (m, 2H, C*H*_2_); δ_C_ (100 MHz, DMSO-*d_6_*): 151.33, 150.74, 148.60, 146.18, 143.78, 138.34, 133.97, 131.57, 127.58, 124.28, 122.57, 121.35, 121.14, 109.32, 106.35, 103.01, 74.98, 57.98, 56.25, 51.58, 29.93, 28.97, 27.29, 23.54; m/z (ESI positive) 433.1 [M]^+^.

#### Synthesis of 10-methoxy-9-(prop-2-yn-1-yloxy)-5,6-dihydro-[1,3]dioxolo[4,5-g]isoquinolino[3,2-a]isoquinolin-7-ium bromide 7

Propargyl bromide (0.11 g, 2.0 eq) was added to a solution of berberrubine **2** (0.25 g, 1.0 eq) in dry DMF (2 mL) and the reaction mixture was stirred at 60 °C o.n. and thereafter treated with Et_2_O. The formed precipitate was filtered and purified by silica gel column chromatography eluting with MeOH/DCM 10% to afford **7** as a yellow powder. 49% yield; m.p. >300 °C; silica gel TLC *R_f_*0.52 (MeOH/DCM 10% *v/v)*; δ_H_ (400 MHz, DMSO-*d_6_*): 9.91 (s, 1H, Ar-*H*), 9.00 (s, 1H, Ar-*H*), 8.27 (d, *J* = 9.2 Hz, 1H, Ar-*H*), 8.09 (d, *J* = 9.1 Hz, 1H, Ar-*H*), 7.84 (s, 1H, Ar-*H*), 7.14 (s, 1H, Ar-*H*), 6.22 (s, 2H, C*H*_2_), 5.13 (d, *J* = 2.1 Hz, 2H, C*H*_2_), 5.00 (t, *J* = 6.0 Hz, 2H, C*H*_2_), 4.12 (s, 3H, C*H*_3_), 3.65 (s, 1H, C*H*), 3.25 (m, 2H, C*H*_2_); δ_C_ (100 MHz, DMSO-*d_6_*): 151.20, 150.38, 148.19, 145.82, 141.19, 138.92, 133.42, 131.22, 127.03, 124.76, 122.58, 120.90, 120.79, 108.92, 105.96, 102.60, 80.30, 79.26, 61.44, 57.65, 55.79, 26.82; m/z (ESI positive) 360.0 [M]^+^.

#### Synthesis of 10-methoxy-9-(pent-4-yn-1-yloxy)-5,6-dihydro-[1,3]dioxolo[4,5-g]isoquinolino[3,2-a]isoquinolin-7-ium bromide 8

5-Bromo-1-pentyne (2.0 eq) was added to a solution of berberrubine **2** (1.0 eq, 0.5 g) and KI (1.2 eq) in dry DMF (3 mL). The reaction mixture was stirred and heated up to 60 °C o.n. The suspension was treated with Et_2_O and the obtained precipitate was filtered to afford **8** as a yellow powder. 90% yield; m.p. 250 °C; silica gel TLC *R_f_* 0.36 (MeOH/DCM 5% v/v); δ_H_ (400 MHz, DMSO-*d_6_*): 9.83 (s, 1H, Ar-*H*), 9.01 (s, 1H, Ar-*H*), 8.24 (d, *J* = 9.1 Hz, 1H, Ar-*H*), 8.04 (d, *J* = 9.1 Hz, 1H, Ar-*H*), 7.84 (s, 1H, Ar-*H*), 7.13 (s, 1H, Ar-*H*), 6.21 (d, *J* = 5.9 Hz, 2H, C*H*_2_), 4.99 (m, 2H, C*H*_2_), 4.41 (t, *J* = 6.3 Hz, 2H, C*H*_2_), 4.10 (s, 3H, C*H*_3_), 3.25 (m, 2H, C*H*_2_), 2.91 (m, 1H, C*H*), 2.53 (d, *J* = 6.5 Hz, 2H, C*H*_2_), 2.09 (m, 2H, C*H*_2_); δ_C_ (100 MHz, DMSO-*d_6_*): 151.00, 151.13, 148.75, 146.72, 143.98, 138.91, 134.02, 131.75, 127.78, 124.47, 122.90, 121.72, 121.34, 109.49, 106.50, 103.19, 84.99, 73.96, 72.79, 58.17, 56.40, 29.58, 27.38, 15.65; m/z (ESI positive) 388.1 [M]^+^.

#### Synthesis of 4-ethynylbenzenesulfonamide 14

PdCl_2_(PPh_3_)_2_ (0.1 eq) and CuI (0.1 eq) were added to a solution of 4-bromo benzenesulfonamide **13** (0.5 g, 1.0 eq), (trimethylsilyl)acetylene (1.2 eq) and Et_3_N (10 eq.) in dry dioxane (5.0 mL) at rt under a nitrogen atmosphere. The reaction mixture was stirred at 100 °C o.n., then quenched with slush and extracted with EtOAc (2 × 20 mL). The collected organic layers were dried over anhydrous Na_2_SO_4_, filtered-off and concentrated *in vacuo* to give a residue that was treated with a 1.0 M TBAF solution in THF (2.0 eq). The reaction mixture was stirred at rt for 2 h and thereafter concentrated under *vacuum*. The obtained residue was purified by silica gel chromatography eluting with 5% MeOH in DCM to afford **14** as a white solid. 65% yield; silica gel TLC *R_f_*0.14 (EtOAc/n-Hex 30% v/v); δ_H_ (400 MHz, DMSO-*d_6_*): 7.85 (d, *J* = 7.8, 2H, Ar*-H*), 7.71 (d, *J* = 7.8, 2H, Ar*-H*), 7.49 (s, 2H, exchange with D_2_O, SO_2_N*H*_2_), 4.47 (s, 1H, C*H*); δ_C_ (100 MHz, DMSO-*d_6_*): 145.1, 133.1, 126.8, 125.9, 84.3, 83.2; m/z (ESI negative) 180.0 [M-H]^–^.

### General procedure for the synthesis of coumarin azide intermediates 18–20

The dibromoalkane (5.0 eq) was added to a suspension of 7-hydroxy-2H-chromen-2-one **17** (0.2 g, 1.0 eq) and K_2_CO_3_ (2.0 eq) in dry DMF (5 mL) and the reaction mixture was stirred at 60 °C until the consumption of the starting material (TLC monitoring). NaN_3_ (5.0 eq) was added, and the reaction mixture was stirred at the same temperature for 3h, treated with H_2_O and extracted with EtOAc (2 × 20 mL). The combine organic layers were washed with brine (3 × 15 mL), dried over Na_2_SO_4_, filtered-off, and concentrated *in vacuo* to give **18–20** as white powders.

#### 7–(3-Azidopropoxy)-2H-chromen-2-one 18

54% yield; δ_H_ (400 MHz, CDCl_3_): 7.57 (d, *J* = 9.5 Hz, 1H, Ar*-H*), 7.31 (d, *J* = 8.3 Hz, 1H, Ar*-H*), 6.78 (m, 2H, Ar*-H*), 6.19 (d, *J* = 9.5 Hz, 1H, Ar*-H*), 4.11 (t, *J* = 5.8 Hz, 2H, C*H*_2_), 3.54 (t, *J* = 6.4 Hz, 2H, C*H*_2_), 2.29 (m, 2H, C*H*_2_); δ_C_ (100 MHz, CDCl_3_): 160.84, 160.11, 154.85, 142.32, 127.80, 113.51, 112.29, 111.73, 100.55, 64.86, 30.95, 28.50; m/z (ESI positive) 246.0 [M + H]^+^.

#### 7–(4-Azidobutoxy)-2H-chromen-2-one 19

90% yield; silica gel TLC *R_f_* 0.5 (EtOAc/Hex 30% v/v); δ_H_ (400 MHz, DMSO-*d_6_*): 7.96 (d, *J* = 9.5 Hz, 1H, Ar-*H*), 7.59 (d, *J* = 8.5 Hz, 1H, Ar-*H*), 6.94 (d, *J* = 2.2 Hz, 1H, Ar-*H*), 6.91 (dd, *J* = 8.5, 2.2 Hz, 1H, Ar-*H*), 6.27 (d, *J* = 9.5 Hz, 1H, Ar-*H*), 4.08 (t, *J* = 6.2 Hz, 2H, C*H*_2_), 3.41 (t, *J* = 6.8 Hz, 2H, C*H*_2_), 1.80 (m, 2H, C*H*_2_), 1.69 (m, 2H, C*H*_2_); δ_C_ (100 MHz, DMSO-*d_6_*): 162.76, 161.33, 156.44, 145.28, 130.45, 113.68, 113.44, 113.34, 102.12, 68.78, 51.40, 26.75, 26.07; m/z (ESI positive) 260.0 [M + H]^+^.

#### 7-((5-Azidopentyl)oxy)-2H-chromen-2-one 20

92% yield; silica gel TLC *R_f_* 0.5 (EtOAc/*n*-hexane 30% *v/v*); δ_H_ (400 MHz, DMSO-*d_6_*): 7.98 (d, *J* = 9.5 Hz, 1H, Ar-*H*), 7.61 (d, *J* = 8.5 Hz, 1H, Ar-*H*), 6.96 (s, 1H, Ar-*H*), 6.92 (m, 1H, Ar-*H*), 6.28 (d, *J* = 9.5 Hz, 1H, Ar-*H*), 4.08 (dd, *J* = 11.6, 5.3 Hz, 2H, C*H*_2_), 3.36 (t, *J* = 3.3 Hz, 2H, C*H*_2_), 1.77 (m, 2H, C*H*_2_), 1.60 (m, 2H, C*H*_2_), 1.48 (m, 2H, C*H*_2_); δ_C_ (100 MHz, DMSO-*d_6_*): 162.89, 161.35, 156.48, 145.34, 130.49, 113.73, 113.43, 113.31, 102.14, 69.16, 51.63, 31.70, 29.02 (d, J = 1.8 Hz), 23.80; m/z (ESI positive) 274.1 [M + H]^+^.

### General procedure for the synthesis of coumarin alkyne intermediates 21–23

The alkynyl halide (1.2 eq) was added to a solution of 7-hydroxy-2H-chromen-2-one **17** (1.0 eq, 0,7 g) and K_2_CO_3_ (1.2 eq) in dry DMF (3 mL). The reaction mixture was stirred and heated up to 80 °C o.n. The reaction was quenched with slush and the obtained precipitate was filtered and washed with Et_2_O to give the title compounds.

#### 7-(Prop-2-yn-1-yloxy)-2H-chromen-2-one 21

98% yield; m.p. 118–119 °C; silica gel TLC *R_f_* 0.5 (EtOAc/Hex 30% *v/v*); δ_H_ (400 MHz, DMSO-*d_6_*): 8.01 (t, *J* = 9.4 Hz, 1H, Ar-*H*), 7.68 (d, *J* = 8.6 Hz, 1H, Ar-*H*), 7.07 (d, J = 2.2 Hz, 1H, Ar-*H*), 7.02 (dd, J = 8.6, 2.2 Hz, 1H, Ar-*H*), 6.35 (d, *J* = 9.5 Hz, 1H, Ar-*H*), 4.97 (d, *J* = 2.2 Hz, 2H, C*H*_2_), 3.69 (t, *J* = 2.1 Hz, 1H, C*H*); δ_C_ (100 MHz, DMSO-*d_6_*): 161.25, 161.01, 156.18, 145.26, 130.56, 114.02, 113.95, 113.92, 102.82, 79.96, 79.56, 57.17; m/z (ESI positive) 201.0 [M + H]^+^.

#### Synthesis of 7-(but-3-yn-1-yloxy)-2H-chromen-2-one 22

33% yield; m.p. 93–94 °C; silica gel TLC *R_f_* 0.5 (EtOAc/Hex 40% *v/v*); δ_H_ (400 MHz, DMSO-*d_6_*): 8.03 (d, *J* = 9.5 Hz, 1H, Ar-*H*), 7.66 (d, *J* = 8.6 Hz, 1H, Ar-*H*), 7.03 (t, *J* = 3.9 Hz, 1H, Ar-*H*), 7.00 (dd, *J* = 8.6, 2.3 Hz, 1H, Ar-*H*), 6.33 (d, *J* = 9.5 Hz, 1H, Ar-*H*), 4.21 (t, *J* = 6.4 Hz, 2H, C*H*_2_), 3.65 (t, *J* = 2.1 Hz, 1H, C*H*), 2.71 (m, 2H, C*H*_2_); δ_C_ (100 MHz, DMSO-*d_6_*): 161.32, 161.01, 156.42, 145.35, 130.60, 113.76, 113.69, 113.60, 102.36, 82.21, 73.61, 67.51, 19.79; m/z (ESI positive) 215.1 [M + H]^+^.

#### 7-(Pent-4-yn-1-yloxy)-2H-chromen-2-one 23

86% yield; m.p. 103–104 °C; silica gel TLC R*_f_* 0.5 (EtOAc/Hex 30% *v/v*); δ_H_ (400 MHz, DMSO-*d_6_*): 7.99 (t, *J* = 9.5 Hz, 1H, Ar *H*), 7.64 (d, *J* = 8.5 Hz, 1H, Ar-*H*), 7.00 (d, *J* = 2.0 Hz, 1H, Ar-*H*), 6.97 (dd, *J* = 8.5, 2.0 Hz, 1H, Ar-*H*), 6.31 (d, *J* = 9.5 Hz, 1H; C*H*), 4.17 (t, *J* = 6.1 Hz, 2H, C*H*_2_), 3.50 (t, *J* = 2.2 Hz, 1H, C*H*), 2.38 (m, 2H, C*H*_2_), 1.95 (m, 2H, C*H*_2_); δ_C_ (100 MHz, DMSO-*d_6_*): 161.34, 161.01, 156.44, 145.34, 130.55, 113.68, 113.55, 113.44, 102.22, 84.55, 72.79, 67.81, 28.54, 15.50; m/z (ESI positive) 229.0 [M + H]^+^.

### General procedure for the cu(I) catalysed azide-alkyne huisgen cycloaddition reactions (26–39, 40–45)

The alkyne (0.2 g) and azide (1.0 eq) derivatives to react were suspended in H_2_O/*t*BuOH 1/1 (12 mL) at rt, and the catalyst made of CuSO_4_ · 5 H_2_O (0.2 eq) and sodium ascorbate (1.0 eq) in H_2_O (1 mL) was added. The suspension was stirred at 40 °C for 24 h, then treated with a 1.0 M KPF_6_ aqueous solution (6.0 eq) and extracted with DCM (3 × 40 mL). The organic layers were dried over Na_2_SO_4_, filtered-off trough Celite^®^, and concentrated *in vacuo.* The raw products were purified by silica gel chromatography eluting with 5 to 15% MeOH in DCM to afford the title 1,2,3-triazole derivatives as yellow-brownish powders.

#### 10-Methoxy-9–(2-(4–(4-sulfamoylphenyl)-1H-1,2,3-triazol-1-yl)ethoxy)-5,6-dihydro-[1,3]dioxolo[4,5-g]isoquinolino[3,2-a]isoquinolin-7-ium hexafluorophosphate 26

Compound **26** was obtained according to the general procedure reported above by reacting azide **3** and alkyne **14.** 59% yield; m.p. 212–215 °C; silica gel TLC *R_f_*0.55 (MeOH/DCM 10% *v/v)*; δ_H_ (400 MHz, DMSO-*d_6_*): 9.60 (s, 1H, Ar-*H*), 8.93 (s, 1H, Ar-*H*), 8.90 (s, 1H, Ar-*H*), 8.20 (d, *J* = 9.0 Hz, 1H, Ar-*H*), 8.06 (d, *J* = 8.0 Hz, 2H, Ar-*H*), 8.01 (d, *J* = 9.0 Hz, 1H, Ar-*H*), 7.93 (d, *J* = 8.0 Hz, 2H, Ar-*H*), 7.80 (s, 1H, Ar-*H*), 7.44 (s, 2H, exchange with D_2_O, SO_2_N*H*_2_), 7.12 (s, 1H, Ar-*H*), 6.21 (s, 2H, C*H*_2_), 5.03 (m, 2H, C*H*_2_), 4.83 (m, 4H, 2 × C*H*_2_), 4.04 (s, 3H, C*H*_3_), 3.18 (m, 2H, C*H*_2_); δ_C_ (100 MHz, DMSO-*d_6_*): 151.16, 150.95, 148.78, 146.35, 146.01, 144.23, 142.80, 138.53, 134.77, 133.93, 131.56, 127.53, 127.51, 126.44, 124.97, 124.33, 122.38, 121.33, 121.18, 109.47, 106.45, 103.14, 73.03, 58.06, 56.48, 51.33, 27.31; ESI-HRMS (m/z) [M]^+^: calculated for C_29_H_26_N_5_O_6_S 572.1598; found 572.1593.

#### 10-Methoxy-9–(2-(4-((4-sulfamoylphenoxy)methyl)-1H-1,2,3-triazol-1-yl)ethoxy)-5,6-dihydro-[1,3]dioxolo[4,5-g]isoquinolino[3,2-a]isoquinolin-7-ium hexafluorophosphate 27

Compound **27** was obtained according to the general procedure reported above by reacting azide **3** and alkyne **16.** 63% yield; m.p. 183–185 °C; silica gel TLC *R_f_*0.56 (MeOH/DCM 10% *v/v)*; δ_H_ (400 MHz, DMSO-*d_6_*): 9.50 (s, 1H, Ar-*H*), 8.93 (s, 1H, Ar-*H*), 8.44 (s, 1H, Ar-*H*), 8.20 (d, *J* = 9.0 Hz, 1H, Ar-*H*), 8.01 (d, *J* = 9.0 Hz, 1H, Ar-*H*), 7.82 (s, 1H, Ar-*H*), 7.76 (d, *J* = 8.4 Hz, 2H, Ar-*H*), 7.26 (s, 2H, exchange with D_2_O, SO_2_N*H*_2_), 7.17 (d, *J* = 8.4 Hz, 2H, Ar-*H*), 7.12 (s, 1H, Ar-*H*), 6.22 (s, 2H, C*H*_2_), 5.26 (s, 2H, C*H*_2_), 4.98 (m, 2H, C*H*_2_), 4.89 (m, 2H, C*H*_2_), 4.79 (m, 2H, C*H*_2_), 4.04 (s, 3H, C*H*_3_), 3.24 (m, 2H, C*H*_2_); δ_C_ (100 MHz, DMSO-*d_6_*): 161.24, 151.00, 150.85, 148.68, 145.84, 143.41, 142.77, 138.41, 137.47, 133.79, 131.53, 128.58, 127.47, 126.36, 124.82, 122.19, 121.28, 121.12, 115.64, 109.39, 106.38, 103.06, 73.11, 62.29, 57.95, 56.40, 51.05, 27.26; ESI-HRMS (m/z) [M]^+^: calculated for C_30_H_28_N_5_O_7_S 602.1704; found 602.1709.

#### 10-Methoxy-9–(3-(4–(4-sulfamoylphenyl)-1H-1,2,3-triazol-1-yl)propoxy)-5,6-dihydro-[1,3]dioxolo[4,5-g]isoquinolino[3,2-a]isoquinolin-7-ium hexafluorophosphate 28

Compound **28** was obtained according to the general procedure reported above by reacting azide **4** and alkyne **14.** 63% yield; m.p. 205–208 °C; silica gel TLC *R_f_*0.50 (MeOH/DCM 10% *v/v)*; δ_H_ (400 MHz, DMSO-*d_6_*): 9.89 (s, 1H, Ar-*H*), 8.97 (s, 1H, Ar-*H*), 8.85 (s, 1H, Ar-*H*), 8.23 (d, *J* = 8.8 Hz, 1H, Ar-*H*), 8.06 (m, 3H, Ar-*H*), 7.93 (d, *J* = 8.0 Hz, 2H, Ar-*H*), 7.83 (s, 1H, Ar-*H*), 7.44 (s, 2H, exchange with D_2_O, SO_2_N*H*_2_), 7.14 (s, 1H, Ar-*H*), 6.22 (s, 2H, C*H*_2_), 5.01 (m, 2H, C*H*_2_), 4.82 (m, 2H, C*H*_2_), 4.38 (m, 2H, C*H*_2_), 4.06 (s, 3H, C*H*_3_), 3.34 (m, 2H, C*H*_2_, overlap with water signal), 3.27 (m, 2H, C*H*_2_); δ_C_ (100 MHz, DMSO-*d_6_*): 151.26, 150.78, 148.62, 146.17, 146.12, 144.10, 143.32, 138.40, 134.85, 133.95, 131.61, 127.57, 127.38, 126.30, 124.53, 123.76, 122.42, 121.32, 121.09, 109.34, 106.33, 103.00, 71.92, 57.92, 56.14, 47.72, 31.05, 27.27; ESI-HRMS (m/z) [M]^+^: calculated for C_30_H_28_N_5_O_6_S 586.1755; found 586.1758.

#### 10-Methoxy-9–(3-(4-((4-sulfamoylphenoxy)methyl)-1H-1,2,3-triazol-1-yl)propoxy)-5,6-dihydro-[1,3]dioxolo[4,5-g]isoquinolino[3,2-a]isoquinolin-7-ium hexafluorophosphate 29

Compound **29** was obtained according to the general procedure reported above by reacting azide **4** and alkyne **16.** 42% yield; m.p. 120–122 °C; silica gel TLC *R_f_*0.45 (MeOH/DCM 10% *v/v)*; δ_H_ (400 MHz, DMSO-*d_6_*): 9.87 (s, 1H, Ar-*H*), 8.97 (s, 1H, Ar-*H*), 8.40 (s, 1H, Ar-*H*), 8.22 (d, *J* = 9.0 Hz, 1H, Ar-*H*), 8.03 (d, *J* = 9.0 Hz, 1H, Ar-*H*), 7.84 (s, 1H, Ar-*H*), 7.78 (d, *J* = 8.4 Hz, 2H, Ar-*H*), 7.27 (s, 2H, exchange with D_2_O, SO_2_N*H*_2_), 7.22 (d, *J* = 8.4 Hz, 2H, Ar-*H*), 7.13 (s, 1H, Ar-*H*), 6.22 (s, 2H, C*H*_2_), 5.29 (s, 2H, C*H*_2_), 5.00 (m, 2H, C*H*_2_), 4.77 (m, 2H, C*H*_2_), 4.33 (m, 2H, C*H*_2_), 4.06 (s, 3H, C*H*_3_), 3.25 (m, 2H, C*H*_2_), 2.49 (m, 2H, C*H*_2_); δ_C_ (100 MHz, DMSO-*d_6_*): 161.26, 151.25, 150.79, 148.64, 146.20, 143.30, 143.20, 138.45, 137.45, 133.97, 131.61, 128.57, 127.60, 125.89, 124.55, 122.42, 121.34, 121.16, 115.66, 109.36, 106.36, 103.01, 71.92, 62.35, 57.93, 56.20, 47.53, 31.17, 27.27; ESI-HRMS (m/z) [M]^+^: calculated for C_31_H_30_N_5_O_7_S 616.1860; found 616.1866.

#### 10-Methoxy-9–(4–(4-(4-sulfamoylphenyl)-1H-1,2,3-triazol-1-yl)butoxy)-5,6-dihydro-[1,3]dioxolo[4,5-g]isoquinolino[3,2-a]isoquinolin-7-ium hexafluorophosphate 30

Compound **30** was obtained according to the general procedure reported above by reacting azide **5** and alkyne **14.** 74% yield; m.p. 183–185 °C; silica gel TLC *R_f_*0.33 (MeOH/DCM 10% *v/v)*; δ_H_ (400 MHz, DMSO-*d_6_*): 9.80 (s, 1H, Ar-*H*), 8.95 (s, 1H, Ar-*H*), 8.80 (s, 1H, Ar-*H*), 8.22 (d, *J* = 8.8 Hz, 1H, Ar-*H*), 8.06 (d, *J* = 8.0 Hz, 2H, Ar-*H*), 8.01 (d, *J* = 8.8 Hz, 1H, Ar-*H*), 7.93 (d, *J* = 8.0 Hz, 2H, Ar-*H*), 7.83 (s, 1H, Ar-*H*), 7.42 (s, 2H, exchange with D_2_O, SO_2_N*H*_2_), 7.12 (s, 1H, Ar-*H*), 6.22 (s, 2H, C*H*_2_), 4.99 (m, 2H, C*H*_2_), 4.62 (m, 2H, C*H*_2_), 4.37 (m, 2H, C*H*_2_), 4.09 (s, 3H, C*H*_3_), 3.25 (m, 2H, C*H*_2_), 2.22 (m, 2H, C*H*_2_), 1.94 (m, 2H, C*H*_2_); δ_C_ (100 MHz, DMSO-*d_6_*): 151.26, 150.76, 148.61, 146.13, 146.04, 144.04, 143.60, 138.36, 134.89, 133.94, 131.57, 127.51, 127.34, 126.26, 124.34, 123.60, 122.50, 121.33, 121.11, 109.32, 106.35, 103.01, 74.42, 57.96, 56.24, 50.38, 27.45, 27.27, 27.21; ESI-HRMS (m/z) [M]^+^: calculated for C_31_H_30_N_5_O_6_S 600.1911; found 600.1912.

#### 10-Methoxy-9–(4-(4-((4-sulfamoylphenoxy)methyl)-1H-1,2,3-triazol-1-yl)butoxy)-5,6-dihydro-[1,3]dioxolo[4,5-g]isoquinolino[3,2-a]isoquinolin-7-ium hexafluorophosphate 31

Compound **31** was obtained according to the general procedure reported above by reacting azide **5** and alkyne **16.** 51% yield; m.p. 165–168 °C; silica gel TLC *R_f_*0.31 (MeOH/DCM 10% *v/v)*; δ_H_ (400 MHz, DMSO-*d_6_*): 9.79 (s, 1H, Ar-*H*), 8.97 (s, 1H, Ar-*H*), 8.35 (s, 1H, Ar-*H*), 8.22 (d, *J* = 9.2 Hz, 1H, Ar-*H*), 8.02 (d, *J* = 9.2 Hz, 1H, Ar-*H*), 7.83 (s, 1H, Ar-*H*), 7.79 (d, *J* = 8.4 Hz, 2H, Ar-*H*), 7.27 (s, 2H, exchange with D_2_O, SO_2_N*H*_2_), 7.22 (d, *J* = 8.4 Hz, 2H, Ar-*H*), 7.12 (s, 1H, Ar-*H*), 6.21 (s, 2H, C*H*_2_), 5.27 (s, 2H, C*H*_2_), 4.99 (m, 2H, C*H*_2_), 4.55 (m, 2H, C*H*_2_), 4.34 (m, 2H, C*H*_2_), 4.08 (s, 3H, C*H*_3_), 3.25 (m, 2H, C*H*_2_), 2.14 (m, 2H, C*H*_2_), 1.89 (m, 2H, C*H*_2_); δ_C_ (100 MHz, DMSO-*d_6_*): 161.28, 151.29, 150.76, 148.62, 146.15, 143.61, 143.09, 138.37, 137.45, 133.94, 131.59, 128.59, 127.53, 125.72, 124.36, 122.51, 121.35, 121.13, 115.70, 109.33, 106.36, 103.02, 74.44, 62.38, 57.96, 56.24, 50.13, 27.43, 27.30, 27.28; ESI-HRMS (m/z) [M]^+^: calculated for C_32_H_32_N_5_O_7_S 630.2017; found 630.2014.

#### 10-Methoxy-9-((5–(4-(4-sulfamoylphenyl)-1H-1,2,3-triazol-1-yl)pentyl)oxy)-5,6-dihydro-[1,3]dioxolo[4,5-g]isoquinolino[3,2-a]isoquinolin-7-ium hexafluorophosphate 32

Compound **32** was obtained according to the general procedure reported above by reacting azide **6** and alkyne **14.** 55% yield; m.p. 200–202 °C; silica gel TLC *R_f_*0.44 (MeOH/DCM 10% *v/v)*; δ_H_ (400 MHz, DMSO-*d_6_*): 9.75 (s, 1H, Ar-*H*), 8.94 (s, 1H, Ar-*H*), 8.78 (s, 1H, Ar-*H*), 8.22 (d, *J* = 8.8 Hz, 1H, Ar-*H*), 8.02 (m, 3H, Ar-*H*), 7.92 (d, *J* = 8.0 Hz, 2H, Ar-*H*), 7.83 (s, 1H, Ar-*H*), 7.44 (s, 2H, exchange with D_2_O, SO_2_N*H*_2_), 7.13 (s, 1H, Ar-*H*), 6.21 (s, 2H, C*H*_2_), 4.98 (m, 2H, C*H*_2_), 4.53 (m, 2H, C*H*_2_), 4.34 (m, 2H, C*H*_2_), 4.07 (s, 3H, C*H*_3_), 3.23 (m, 2H, C*H*_2_), 2.04 (m, 2H, 2 × C*H*_2_), 1.95 (m, 2H, 2 × C*H*_2_),1.56 (m, 2H, C*H*_2_); δ_C_ (100 MHz, DMSO-*d_6_*): 151.23, 150.73, 148.60, 146.06, 145.94, 144.00, 143.69, 138.35, 134.85, 133.93, 131.55, 127.57, 127.28, 126.16, 124.18, 123.43, 122.50, 121.33, 121.10, 109.30, 106.32, 102.97, 74.85, 57.92, 56.20, 50.53, 30.19, 29.76, 27.23, 23.31; ESI-HRMS (m/z) [M]^+^: calculated for C_32_H_32_N_5_O_6_S 614.2068; found 614.2073.

#### 10-Methoxy-9-((5–(4-((4-sulfamoylphenoxy)methyl)-1H-1,2,3-triazol-1-yl)pentyl)oxy)-5,6-dihydro-[1,3]dioxolo[4,5-g]isoquinolino[3,2-a]isoquinolin-7-ium hexafluorophosphate 33

Compound **33** was obtained according to the general procedure reported above by reacting azide **6** and alkyne **16.** 20% yield; m.p. 168–171 °C; silica gel TLC *R_f_*0.38 (MeOH/DCM 10% *v/v)*; δ_H_ (400 MHz, DMSO-*d_6_*): 9.77 (s, 1H, Ar-*H*), 8.97 (s, 1H, Ar-*H*), 8.32 (s, 1H, Ar-*H*), 8.22 (d, *J* = 8.8 Hz, 1H, Ar-*H*), 8.03 (d, *J* = 8.8 Hz, 1H, Ar-*H*), 7.84 (s, 1H, Ar-*H*), 7.78 (d, *J* = 8.2 Hz, 2H, Ar-*H*), 7.26 (s, 2H, exchange with D_2_O, SO_2_N*H*_2_), 7.21 (d, *J* = 8.2 Hz, 2H, Ar-*H*), 7.13 (s, 1H, Ar-*H*), 6.21 (s, 2H, C*H*_2_), 5.25 (s, 2H, C*H*_2_), 4.98 (m, 2H, C*H*_2_), 4.47 (m, 2H, C*H*_2_), 4.30 (m, 2H, C*H*_2_), 4.08 (s, 3H, C*H*_3_), 3.24 (m, 2H, C*H*_2_), 1.96 (m, 4H, 2 × C*H*_2_), 1.51 (m, 2H, C*H*_2_); δ_C_ (100 MHz, DMSO-*d_6_*): 161.23, 151.28, 150.74, 148.60, 146.11, 143.71, 143.00, 138.37, 137.42, 133.94, 131.58, 128.53, 127.59, 125.55, 124.22, 122.54, 121.34, 121.12, 115.64, 109.31, 106.33, 102.98, 74.85, 62.35, 57.91, 56.21, 50.29, 30.31, 29.72, 27.25, 23.27; ESI-HRMS (m/z) [M]^+^: calculated for C_33_H_34_N_5_O_7_S 644.2173; found 644.2175.

#### 10-Methoxy-9–(3-(4-(((2-oxo-2H-chromen-7-yl)oxy)methyl)-1H-1,2,3-triazol-1-yl)propoxy)-5,6-dihydro-[1,3]dioxolo[4,5-g]isoquinolino[3,2-a]isoquinolin-7-ium hexafluorophosphate 34

Compound **34** was obtained according to the general procedure reported above by reacting azide **4** and alkyne **21.** 85% yield; m.p. 140 °C; silica gel TLC R_f_ 0.18 (MeOH/DCM 10% *v/v*); δ_H_ (400 MHz, DMSO-*d_6_*): 9.77 (s, 1H, Ar-*H*), 8.94 (s, 1H, Ar-*H*), 8.43 (s, 1H, Ar-*H*), 8.21 (d, *J* = 9.2 Hz, 1H, Ar-*H*), 8.00 (m, 2H, Ar-*H*), 7.82 (s, 1H, Ar-*H*), 7.62 (d, *J* = 8.8 Hz, 1H, Ar-*H*), 7.15 (m, 2H, Ar-*H*), 7.01 (dd, *J* = 8.6, 2.2 Hz, 1H, Ar-*H*), 6.32 (d, *J* = 9.2 Hz, 1H, Ar-*H*), 6.21 (s, 2H, C*H*_2_), 5.32 (s, 2H, C*H*_2_), 4.98 (m, 2H, C*H*_2_), 4.77 (t, *J* = 6.7 Hz, 2H, C*H*_2_), 4.32 (t, *J* = 5.8 Hz, 2H, C*H*_2_), 4.06 (s, 3H, C*H*_3_), 3.25 (m, 2H, C*H*_2_), 2.50 (m, 2H, C*H*_2_); δ_C_ (100 MHz, DMSO-*d_6_*): 161.91, 161.09, 156.11, 151.23, 150.79, 148.63, 146.04, 145.10, 143.25, 143.03, 138.41, 133.90, 131.56, 130.34, 127.57, 126.00, 124.48, 122.33, 121.29, 121.11, 113.77, 113.56, 113.43, 109.33, 106.32, 103.00, 102.42, 72.05, 62.58, 57.90, 56.21, 47.65, 31.10, 27.24; ESI-HRMS (m/z) [M]^+^: calculated for C_34_H_29_N_4_O_7_ 605.2031; found 605.2036.

#### 10-Methoxy-9–(3-(4–(2-((2-oxo-2H-chromen-7-yl)oxy)ethyl)-1H-1,2,3-triazol-1-yl)propoxy)-5,6-dihydro-[1,3]dioxolo[4,5-g]isoquinolino[3,2-a]isoquinolin-7-ium hexafluorophosphate 35

Compound **35** was obtained according to the general procedure reported above by reacting azide **4** and alkyne **22.** 42% yield; m.p. 130–131 °C; silica gel TLC *R_f_* 0.2 (MeOH/DCM 5% *v/v*); δ_H_ (400 MHz, DMSO-*d_6_*): δ 9.80 (s, 1H, Ar-*H*), 8.92 (s, 1H, Ar-*H*), 8.20 (d, *J* = 9.2 Hz, 1H, Ar-*H*), 8.14 (s, 1H, Ar-*H*), 7.99 (m, 2H, Ar-*H*), 7.79 (s, 1H, Ar-*H*), 7.58 (d, *J* = 8.8 Hz, 1H, Ar-*H*), 7.11 (s, 1H, Ar-*H*), 6.97 (d, *J* = 2.1 Hz, 1H, Ar-*H*), 6.89 (dd, *J* = 8.6, 2.2 Hz, 1H, Ar-*H*), 6.31 (d, *J* = 9.2 Hz, 1H, Ar-*H*), 6.21 (s, 2H, C*H*_2_), 4.99 (m, 2H, C*H*_2_), 4.73 (t, *J* = 6.7 Hz, 2H, C*H*_2_), 4.37 (t, *J* = 6.3 Hz, 2H, C*H*_2_), 4.29 (t, *J* = 5.7 Hz, 2H, C*H*_2_), 4.05 (s, 3H, C*H*_3_), 3.25 (m, 2H, C*H*_2_), 3.17 (t, *J* = 6.2 Hz, 2H, C*H*_2_), 2.47 (m, 2H, C*H*_2_); δ_C_ (100 MHz, DMSO-*d_6_*): 162.40, 161.17, 156.21, 151.27, 150.81, 148.65, 146.08, 145.18, 144.47, 143.24, 138.40, 133.90, 131.57, 130.39, 127.54, 124.55, 124.05, 122.39, 121.30, 121.16, 113.47, 113.43, 113.33, 109.36, 106.33, 103.06, 102.16, 72.00, 68.26, 57.92, 56.21, 47.40, 31.19, 27.27, 26.29. ESI-HRMS (m/z) [M]^+^: calculated for C_35_H_31_N_4_O_7_ 619.2187; found 619.2182.

#### 10-Methoxy-9–(3-(4–(3-((2-oxo-2H-chromen-7-yl)oxy)propyl)-1H-1,2,3-triazol-1-yl)propoxy)-5,6-dihydro-[1,3]dioxolo[4,5-g]isoquinolino[3,2-a]isoquinolin-7-ium hexafluorophosphate 36

Compound **36** was obtained according to the general procedure reported above by reacting azide **4** and alkyne **23.** 30% yield; m.p. 145–146 °C; silica gel TLC *R_f_* 0.5 (MeOH/DCM 10% *v/v*); δ_H_ (400 MHz, DMSO-*d_6_*): 9.84 (s, 1H, Ar-*H*), 8.93 (s, 1H, Ar-*H*), 8.20 (d, *J* = 9.2 Hz, 1H, Ar-*H*), 8.06 (s, 1H, Ar-*H*), 8.00 (d, *J* = 9.6 Hz, 2H, Ar-*H*), 7.81 (s, 1H, Ar-*H*), 7.61 (d, *J* = 9.2 Hz, 1H, Ar-*H*), 7.12 (s, 1H, Ar-*H*), 6.93 (m, 2H, Ar-*H*), 6.30 (d, *J* = 9.2 Hz, 1H, Ar-*H*), 6.21 (s, 2H, C*H*_2_), 5.00 (m, 2H, C*H*_2_), 4.70 (t, *J* = 6.7 Hz, 2H, C*H*_2_), 4.28 (t, *J* = 5.8 Hz, 2H, C*H*_2_), 4.13 (t, *J* = 6.3 Hz, 2H, C*H*_2_), 4.06 (s, 3H, C*H*_3_), 3.25 (m, 2H, C*H*_2_), 2.84 (t, *J* = 7.3 Hz, 2H, C*H*_2_), 2.45 (m, 2H, C*H*_2_), 2.11 (m, 2H, C*H*_2_); δ_C_ (100 MHz, DMSO-*d_6_*): 162.59, 161.13, 156.24, 151.25, 150.79, 148.62, 146.07, 145.18, 145.16, 143.26, 138.41, 133.91, 131.55, 130.35, 127.55, 124.51, 123.18, 122.38, 121.28, 121.13, 113.54, 113.33, 113.20, 109.32, 106.31, 103.00, 101.96, 71.97, 68.34, 57.91, 56.19, 47.26, 31.14, 29.10, 27.25, 22.37; ESI-HRMS (m/z) [M]^+^: calculated for C_36_H_33_N_4_O_7_ 633.2344; found 633.2340.

#### 10-Methoxy-9–(4-(4-(((2-oxo-2H-chromen-7-yl)oxy)methyl)-1H-1,2,3-triazol-1-yl)butoxy)-5,6-dihydro-[1,3]dioxolo[4,5-g]isoquinolino[3,2-a]isoquinolin-7-ium hexafluorophosphate 37

Compound **37** was obtained according to the general procedure reported above by reacting azide **5** and alkyne **21.** 45% yield; m.p. 108–110 °C; silica gel TLC R*_f_* 0.32 (MeOH/DCM 10% *v/v*); δ_H_ (400 MHz, DMSO-*d_6_*): 9.78 (s, 1H, Ar-*H*), 8.95 (s, 1H, Ar-*H*), 8.38 (s, 1H, Ar-*H*), 8.22 (d, *J* = 9.0 Hz, 1H, Ar-*H*), 8.02 (d, *J* = 9.2 Hz, 2H, Ar-*H*), 7.81 (s, 1H, Ar-*H*), 7.67 (d, *J* = 9.0 Hz, 1H, Ar-*H*), 7.19 (s, 1H, Ar-*H*), 7.12 (s, 1H, Ar-*H*), 7.04 (m, 1H, Ar-*H*), 6.33 (d, *J* = 9.2 Hz, 1H, Ar-*H*), 6.21 (s, 2H, C*H*_2_), 5.30 (s, 2H, C*H*_2_), 4.98 (m, 2H, C*H*_2_), 4.56 (m, 2H, C*H*_2_), 4.33 (m, 2H, C*H*_2_), 4.08 (s, 3H, C*H*_3_), 3.24 (m, 2H, C*H*_2_), 2.15 (m, 2H, C*H*_2_), 1.88 (m, 2H, C*H*_2_); δ_C_ (100 MHz, DMSO-*d_6_*): 161.99, 161.13, 156.17, 151.22, 150.72, 148.58, 145.81, 145.17, 143.59, 142.90, 138.35, 133.89, 131.56, 130.41, 127.51, 125.80, 124.32, 122.44, 121.36, 121.04, 113.81, 113.55, 113.49, 109.29, 106.33, 102.99, 102.47, 74.38, 62.67, 57.96, 56.18, 50.14, 27.41, 27.28, 27.26; ESI-HRMS (m/z) [M]^+^: calculated for C_35_H_31_N_4_O_7_ 619.2187; found 619.2182.

#### 10-Methoxy-9–(4-(4–(2-((2-oxo-2H-chromen-7-yl)oxy)ethyl)-1H-1,2,3-triazol-1-yl)butoxy)-5,6-dihydro-[1,3]dioxolo[4,5-g]isoquinolino[3,2-a]isoquinolin-7-ium hexafluorophosphate 38

Compound **38** was obtained according to the general procedure reported above by reacting azide **5** and alkyne **22.** 82% yield; m.p. 185–186 °C; silica gel TLC *R_f_* 0.21 (MeOH/DCM 5% *v/v*); δ_H_ (400 MHz, DMSO-*d_6_*): 9.76 (s, 1H, Ar-*H*), 8.91 (s, 1H, Ar-*H*), 8.19 (d, *J* = 9.2 Hz, 1H, Ar-*H*), 8.07 (s, 1H, Ar-*H*), 7.98 (d, *J* = 9.2 Hz, 2H, Ar-*H*), 7.78 (s, 1H, Ar-*H*), 7.61 (d, *J* = 8.4 Hz, 1H, Ar-*H*), 7.10 (s, 1H, Ar-*H*), 6.97 (m, 2H, Ar-*H*), 6.29 (d, *J* = 9.4 Hz, 1H Ar-*H*), 6.21 (s, 2H, C*H*_2_), 4.99 (m, 2H, C*H*_2_), 4.51 (m, 2H, C*H*_2_), 4.34 (m, 4H, 2 × C*H*_2_), 4.07 (s, 3H, C*H*_3_), 3.25 (m, 2H, C*H*_2_), 3.16 (t, *J* = 6.1 Hz, 2H, C*H*_2_), 2.11 (m, 2H, C*H*_2_), 1.88 (m, 2H, C*H*_2_); δ_C_ (100 MHz, DMSO-*d_6_*): 162.43, 161.18, 156.23, 151.25, 150.77, 148.62, 146.10, 145.19, 144.19, 143.54, 138.38, 133.89, 131.58, 130.41, 127.51, 124.32, 123.82, 122.47, 121.33, 121.12, 113.51, 113.45, 113.35, 109.33, 106.32, 103.04, 102.22, 74.39, 68.24, 57.91, 56.24, 49.94, 27.45, 27.31, 27.26, 26.28. ESI-HRMS (m/z) [M]^+^: calculated for C_36_H_33_N_4_O_7_ 633.2344; found 633.2349.

#### 10-Methoxy-9-((5–(4-(((2-oxo-2H-chromen-7-yl)oxy)methyl)-1H-1,2,3-triazol-1-yl)pentyl)oxy)-5,6-dihydro-[1,3]dioxolo[4,5-g]isoquinolino[3,2-a]isoquinolin-7-ium hexafluorophosphate 39

Compound **39** was obtained according to the general procedure reported above by reacting azide **6** and alkyne **21.** 55% yield; m.p. 206–207 °C; silica gel TLC *R_f_* 0.22 (MeOH/DCM 5% *v/v*); δ_H_ (400 MHz, DMSO-*d_6_*): 9.76 (s, 1H, Ar-*H*), 8.96 (s, 1H, Ar-*H*), 8.35 (s, 1H, Ar-*H*), 8.22 (d, *J* = 9.2 Hz, 1H, Ar-*H*), 8.01 (d, *J* = 9.2 Hz, 2H, Ar-*H*), 7.83 (s, 1H, Ar-*H*), 7.65 (d, *J* = 8.6 Hz, 1H, Ar-*H*), 7.15 (d, *J* = 2.1 Hz, 1H, Ar-*H*), 7.12 (s, 1H, Ar-*H*), 7.02 (dd, *J* = 8.6, 2.2 Hz, 1H, Ar-*H*), 6.32 (d, *J* = 9.2 Hz, 1H, Ar-*H*), 6.21 (s, 2H, C*H*_2_), 5.28 (s, 2H, C*H*_2_), 4.98 (m, 2H, C*H*_2_), 4.49 (t, *J* = 6.8 Hz, 2H, C*H*_2_), 4.30 (t, *J* = 6.4 Hz, 2H, C*H*_2_), 4.07 (s, 3H, C*H*_3_), 3.25 (m, 2H, C*H*_2_), 1.96 (m, 4H, 2 × C*H*_2_), 1.52 (m, 2H, C*H*_2_); δ_C_ (100 MHz, DMSO-*d_6_*): 161.99, 161.16, 156.19, 151.29, 150.76, 148.62, 146.08, 145.17, 143.66, 142.85, 138.38, 133.93, 131.59, 130.41, 127.56, 125.72, 124.24, 122.52, 121.35, 121.14, 113.80, 113.59, 113.49, 109.34, 106.32, 103.03, 102.45, 74.84, 62.64, 57.91, 56.25, 50.33, 30.35, 29.76, 27.26, 23.31; ESI-HRMS (m/z) [M]^+^: calculated for C_36_H_33_N_4_O_7_ 633.2344; found 633.2348.

#### 10-Methoxy-9-((1–(3-sulfamoylphenyl)-1H-1,2,3-triazol-4-yl)methoxy)-5,6-dihydro-[1,3]dioxolo[4,5-g]isoquinolino[3,2-a]isoquinolin-7-ium hexafluorophosphate 40

Compound **40** was obtained according to the general procedure reported above by reacting azide **11** and alkyne **7.** 34% yield; m.p. 195–198 °C; silica gel TLC *R_f_*0.45 (MeOH/DCM 10% *v/v)*; δ_H_ (400 MHz, DMSO-*d_6_*): 9.81 (s, 1H, Ar-*H*), 9.20 (s, 1H, Ar-*H*), 8.99 (s, 1H, Ar-*H*), 8.41 (s, 1H, Ar-*H*), 8.29 (d, *J* = 9.2 Hz, 1H, Ar-*H*), 8.17 (d, *J* = 7.6 Hz, 1H, Ar-*H*), 8.07 (d, *J* = 9.2 Hz, 1H, Ar-*H*), 7.98 (d, *J* = 7.6 Hz, 1H, Ar-*H*), 7.88 (t, *J* = 7.6 Hz, 1H, Ar-*H*), 7.88 (s, 1H, Ar-*H*), 7.63 (s, 2H, exchange with D_2_O, SO_2_N*H*_2_), 7.12 (s, 1H, Ar-*H*), 6.21 (s, 2H, C*H*_2_), 5.61 (s, 2H, C*H*_2_), 4.97 (m, 2H, C*H*_2_), 4.16 (s, 3H, C*H*_3_), 3.23 (m, 2H, C*H*_2_); δ_C_ (100 MHz, DMSO-*d_6_*): 151.69, 150.85, 148.67, 146.76, 146.11, 145.01, 142.65, 138.56, 137.60, 134.00, 131.97, 131.64, 127.58, 126.64, 125.01, 124.66, 124.18, 122.71, 121.35, 121.23, 118.31, 109.39, 106.39, 103.04, 67.39, 58.10, 56.36, 27.30; ESI-HRMS (m/z) [M]^+^: calculated for C_28_H_24_N_5_O_6_S 558.1442; found 558.1444.

#### 10-Methoxy-9-((1–(4-sulfamoylphenyl)-1H-1,2,3-triazol-4-yl)methoxy)-5,6-dihydro-[1,3]dioxolo[4,5-g]isoquinolino[3,2-a]isoquinolin-7-ium hexafluorophosphate 41

Compound **41** was obtained according to the general procedure reported above by reacting azide **12** and alkyne **7.** 19% yield; m.p. 250–253 °C; silica gel TLC *R_f_*0.26 (MeOH/DCM 10% *v/v)*; δ_H_ (400 MHz, DMSO-*d_6_*): 9.80 (s, 1H, Ar-*H*), 9.21 (s, 1H, Ar-*H*), 8.99 (s, 1H, Ar-*H*), 8.29 (d, *J* = 8.8 Hz, 1H, Ar-*H*), 8.18 (d, *J* = 7.6 Hz, 2H, Ar-*H*), 8.08 (m, 3H, Ar-*H*), 7.83 (s, 1H, Ar-*H*), 7.59 (s, 2H, exchange with D_2_O, SO_2_N*H*_2_), 7.13 (s, 1H, Ar-*H*), 6.21 (s, 2H, C*H*_2_), 5.61 (s, 2H, C*H*_2_), 4.97 (m, 2H, C*H*_2_), 4.16 (s, 3H, C*H*_3_), 3.23 (m, 2H, C*H*_2_); δ_C_ (100 MHz, DMSO-*d_6_*): 151.66, 150.86, 148.68, 146.11, 145.05, 149.99, 142.65, 139.51, 138.57, 133.99, 131.62, 128.53, 127.57, 124.99, 124.59, 122.68, 121.46, 121.34, 121.23, 109.38, 106.37, 103.07, 67.45, 58.08, 56.40, 27.32; ESI-HRMS (m/z) [M]^+^: calculated for C_28_H_24_N_5_O_6_S 558.1442; found 558.1444.

#### 10-Methoxy-9-((1–(3-((2-oxo-2H-chromen-7-yl)oxy)propyl)-1H-1,2,3-triazol-4-yl)methoxy)-5,6-dihydro-[1,3]dioxolo[4,5-g]isoquinolino[3,2-a]isoquinolin-7-ium hexafluorophosphate 42

Compound **42** was obtained according to the general procedure reported above by reacting azide **18** and alkyne **7.** 78% yield; m.p. 117–118 °C; silica gel TLC *R_f_* 0.16 (MeOH/DCM 2.5% *v/v*); δ_H_ (400 MHz, DMSO-*d_6_*): 9.70 (s, 1H, Ar-*H*), 8.90 (s, 1H, Ar-*H*), 8.41 (s, 1H, Ar-*H*), 8.23 (d, *J* = 9.2 Hz, 1H, Ar-*H*), 8.01 (m, 2H, Ar-*H*), 7.75 (s, 1H, Ar-*H*), 7.63 (d, *J* = 8.4 Hz, 1H, Ar-*H*), 7.09 (s, 1H, Ar-*H*), 6.88 (m, 2H, Ar-*H*), 6.32 (d, *J* = 9.2 Hz, 1H, Ar-*H*), 6.20 (s, 2H, C*H*_2_), 5.51 (s, 2H, C*H*_2_), 4.93 (m, 2H, C*H*_2_), 4.57 (m, 2H, C*H*_2_), 4.13 (s, 3H, C*H*_3_), 3.99 (m, 2H, C*H*_2_), 3.20 (m, 2H, C*H*_2_), 2.30 (m, 2H, C*H*_2_); δ_C_ (100 MHz, DMSO-*d_6_*): 162.22, 161.15, 156.17, 151.72, 150.77, 148.59, 146.02, 145.14, 143.40, 142.50, 138.32, 133.79, 131.44, 130.38, 127.44, 126.20, 124.78, 122.79, 121.20, 121.12, 113.49, 113.40, 113.39, 109.26, 106.24, 103.03, 102.13, 67.41, 66.20, 57.94, 56.27, 47.44, 30.13, 27.25; ESI-HRMS (m/z) [M]^+^: calculated for C_34_H_29_N_4_O_7_ 605.2031; found 605.2031.

#### 10-Methoxy-9-((1–(4-((2-oxo-2H-chromen-7-yl)oxy)butyl)-1H-1,2,3-triazol-4-yl)methoxy)-5,6-dihydro-[1,3]dioxolo[4,5-g]isoquinolino[3,2-a]isoquinolin-7-ium hexafluorophosphate 43

Compound **43** was obtained according to the general procedure reported above by reacting azide **19** and alkyne **7.** 51% yield; m.p. 124–125 °C; silica gel TLC *R_f_* 0.3 (MeOH/DCM 5% *v/v*); δ_H_ (400 MHz, DMSO-*d_6_*): 9.70 (s, 1H. Ar-*H*), 8.88 (s, 1H, Ar-*H*), 8.34 (s, 1H, Ar-*H*), 8.23 (d, *J* = 9.2 Hz, 1H, Ar-*H*), 8.01 (d, *J* = 9.6 Hz, 2H, Ar-*H*), 7.70 (s, 1H, Ar-*H*), 7.62 (d, *J* = 8.4 Hz, 1H, Ar-*H*), 7.02 (s, 1H, Ar-*H*), 6.89 (m, 2H, Ar-*H*), 6.31 (d, *J* = 9.4 Hz, 1H, Ar-*H*), 6.17 (s, 2H, C*H*_2_), 5.52 (s, 2H, C*H*_2_), 4.93 (m, 2H, C*H*_2_), 4.45 (t, *J* = 6.6 Hz, 2H, C*H*_2_), 4.14 (s, 3H, C*H*_3_), 3.98 (t, *J* = 6.1 Hz, 2H, C*H*_2_), 3.19 (m, 2H, C*H*_2_), 1.93 (m, 2H, C*H*_2_), 1.49 (m, 2H, C*H*_2_); δ_C_ (100 MHz, DMSO-*d_6_*): 162.61, 161.36, 156.39, 151.97, 150.88, 148.69, 146.17, 145.35, 143.29, 142.41, 138.43, 133.90, 131.54, 130.48, 127.58, 126.29, 124.97, 123.04, 121.29, 121.28, 113.61, 113.50, 113.36, 109.40, 106.35, 103.15, 102.11, 68.56, 67.35, 58.09, 56.41, 50.07; ESI-HRMS (m/z) [M]^+^: calculated for C_35_H_31_N_4_O_7_ 619.2187; found 619.2182.

#### 10-Methoxy-9-((1–(5-((2-oxo-2H-chromen-7-yl)oxy)pentyl)-1H-1,2,3-triazol-4-yl)methoxy)-5,6-dihydro-[1,3]dioxolo[4,5-g]isoquinolino[3,2-a]isoquinolin-7-ium hexafluorophosphate 44

Compound **44** was obtained according to the general procedure reported above by reacting azide **20** and alkyne **7.** 39% yield; m.p. 171–173 °C; silica gel TLC *R_f_* 0.34 (MeOH/DCM 10% *v/v)*; δ_H_ (400 MHz, DMSO-*d_6_*): 9.72 (s, 1H. Ar-*H*), 8.94 (s, 1H, Ar-*H*), 8.34 (s, 1H, Ar-*H*), 8.25 (d, *J* = 9.2 Hz, 1H, Ar-*H*), 8.02 (m, 2H, Ar-*H*), 7.78 (s, 1H, Ar-*H*), 7.63 (d, *J* = 8.5 Hz, 1H, Ar-*H*), 7.08 (s, 1H, Ar-*H*), 6.93 (m, 2H, Ar-*H*), 6.31 (d, *J* = 9.6 Hz, 1H, Ar-*H*), 6.19 (s, 2H, C*H*_2_), 5.50 (s, 2H, C*H*_2_), 4.94 (m, 2H, C*H*_2_), 4.42 (t, *J* = 6.6 Hz, 2H, C*H*_2_), 4.14 (s, 3H, C*H*_3_), 4.02 (t, *J* = 6.1 Hz, 2H, C*H*_2_), 3.21 (m, 2H, C*H*_2_), 1.86 (m, 2H, C*H*_2_), 1.72 (m, 2H, C*H*_2_), 1.28 (m, 2H, C*H*_2_); δ_C_ (100 MHz, DMSO-*d_6_*): 162.79, 161.37, 156.44, 151.91, 150.91, 148.74, 146.25, 145.38, 143.40, 142.59, 138.49, 133.93, 131.63, 130.51, 127.59, 126.13, 124.94, 122.96, 121.38, 121.27, 113.69, 113.47, 113.33, 109.46, 106.41, 103.17, 102.12, 68.92, 67.45, 57.97, 56.31, 50.18, 32.25, 30.28, 28.62, 27.29, 23.21; ESI-HRMS (m/z) [M]^+^: calculated for C_36_H_33_N_4_O_7_ 633.2344; found 633.2340.

#### 10-Methoxy-9-((1–(4-methyl-2-oxo-2H-chromen-7-yl)-1H-1,2,3-triazol-4-yl)methoxy)-5,6-dihydro-[1,3]dioxolo[4,5-g]isoquinolino[3,2-a]isoquinolin-7-ium hexafluorophosphate 45

Compound **45** was obtained according to the general procedure reported above by reacting azide **25** and alkyne **7.** 46% yield; m.p. 280–282 °C; silica gel TLC *R_f_* 0.55 (MeOH/DCM 10% *v/v)*; δ_H_ (400 MHz, DMSO-*d_6_*): 9.79 (s, 1H, Ar-*H*), 9.24 (s, 1H, Ar-*H*), 8.97 (s, 1H, Ar-*H*), 8.28 (d, *J* = 9.2 Hz, 1H, Ar-*H*), 8.05 (m, 4H, Ar-*H*), 7.82 (s, 1H, Ar-*H*), 7.11 (s, 1H, Ar-*H*), 6.54 (s, 1H, Ar-*H*), 6.21 (s, 2H, C*H*_2_), 5.61 (s, 2H, C*H*_2_), 4.96 (m, 2H, C*H*_2_), 4.16 (s, 3H, C*H*_3_), 3.22 (m, 2H, C*H*_2_), 2.53 (s, 3H, C*H*_3_, overlapped with DMSO signal); δ_C_ (100 MHz, DMSO-*d_6_*): 160.22, 154.49, 153.54, 151.55, 150.77, 148.59, 145.99, 144.97, 142.54, 139.26, 138.48, 133.90, 131.52, 128.14, 127.52, 124.88, 124.41, 122.57, 121.24, 121.14, 120.46, 116.44, 115.66, 109.29, 108.43, 106.26, 102.99, 67.35, 58.01, 56.29, 27.22, 18.95; ESI-HRMS (m/z) [M]^+^: calculated for C_32_H_25_N_4_O_6_ 561.1769; found 561.1765.

#### Synthesis of 10-methoxy-9–(3-sulfamoylpropoxy)-5,6-dihydro-[1,3]dioxolo[4,5-g]isoquinolino[3,2-a]isoquinolin-7-ium hexafluorophosphate 47

1,3-Propanesultone (2.0 eq) was added to a solution of berberrubine **2** (0.5 g, 1.0 eq) in dry DMF (2 mL) and the reaction mixture was stirred at 60 °C for 3 h. The crude zwitterion sulphonate **46** obtained upon treatment with Et_2_O (20 mL) was filtered, suspended in SOCl_2_ (10 mL) and DMF (catalytic amount). The mixture was stirred at rt o.n. and concentrated in *vacuo*. The residue was solubilised in THF and treated with NH_4_OH 30% (4.0 eq). The mixture was stirred at rt for 1 h and then treated with a 1.0 M KPF_6_ aqueous solution (6.0 eq). The precipitate was filtered and purified by silica gel chromatography eluting with 5 to 15% MeOH in DCM to afford **47** as a yellow powder. 12% yield; silica gel TLC *R_f_* 0.3 (MeOH/DCM 5%); δ_H_ (400 MHz, DMSO-*d_6_*): 9.84 (s, 1H, Ar-*H*), 8.98 (s, 1H, Ar-*H*), 8.25 (d, *J* = 8.9 Hz, 1H, Ar-*H*), 8.05 (d, *J* = 8.9 Hz, 1H, Ar-*H*), 7.84 (s, 1H, Ar-*H*), 7.14 (s, 1H, Ar-*H*), 6.95 (s, 2H, exchange with D_2_O, SO_2_N*H*_2_), 6.21 (s, 2H, C*H*_2_), 4.98 (m, 2H, C*H*_2_), 4.42 (m, 2H, C*H*_2_), 4.10 (s, 3H, C*H*_3_), 3.34 (m, 2H, C*H*_2_), 3.25 (s, 2H, C*H*_2_), 2.32 (m, 2H, C*H*_2_); δ_C_ (100 MHz, DMSO-*d_6_*): 151.47, 150.95, 148.79, 146.38, 143.49, 138.61, 134.09, 131.79, 127.75, 124.67, 122.66, 121.52, 121.33, 109.52, 106.51, 103.18, 73.58, 58.16, 56.39, 52.49, 27.42, 25.66; ESI-HRMS (m/z) [M]^+^: calculated for C_22_H_23_N_2_O_6_S 443.1271; found 443.1277.

### Carbonic anhydrase inhibition

An Applied Photophysics stopped-flow instrument has been used for assaying the CA-catalysed CO_2_ hydration activity^49^, as reported earlier^76^. The enzyme concentrations were in the range 5–12 nM. All hCA isoforms were recombinant proteins, being obtained in-house as reported earlier^77^.

### Oligonucleotide synthesis and sample preparation

DNA sequences were synthesised at a 1-µmol scale on an ABI 394 DNA/RNA synthesiser (Applied Biosystem, Foster City, CA, USA), by exploiting the standard β-cyanoethylphosphoramidite solid-phase chemistry, as described elsewhere^78^. A treatment with an aqueous solution of concentrated ammonia at 55 °C, for 12 h, was used to subsequently detach the oligonucleotides from the support and remove the semi-permanent protection groups. DNA molecules were then purified by high-performance liquid chromatography (HPLC) equipped with a Nucleogel SAX 1000–8/46 column (Macherey-Nagel, GmbH & Co. KG, Düren, Germany), as previously reported^79^. The purified fractions were desalted by means of Sep-pak cartridges (C-18) and the isolated oligomers proved to be more than 98% pure, by NMR. In particular, the following DNA sequences were synthesised and employed for the experiments: d(AGGGAGGGCGCTGGGAGGAGGG) (*c-Kit1*), d(TGAGGGTGGGTAGGGTGGGTAA) (*c-Myc*), d(TAGGGTTAGGGTTAGGGTTAGGG) (*Tel_23_*), and d(CGCGAATTCGCGTTTCGCGAATTCGCG) (*Hairpin*). Oligonucleotides were dissolved in the proper buffer and their concentration was measured by UV absorption at 90 °C, as previously reported^80^^,^^81^. To achieve the correct folding of the sequences, the DNA solutions were heated in a water bath at 90 °C for 5 min, and then allowed to cool slowly to room temperature overnight. Then, DNA samples were stored at 4 °C for 24 h, before data acquisition.

### Circular dichroism (CD) experiments

CD experiments were carried out on a Jasco J-815 spectropolarimeter (JASCO Inc., Tokyo, Japan) equipped with a PTC-423S/15 Peltier temperature controller, using quartz cuvettes with a path length of 0.1 cm. The samples were prepared by dissolving the oligonucleotides in 5 mM KH_2_PO_4_/K_2_HPO_4_ buffer (pH 7.0) containing 20 mM KCl (or LiCl in the case of *c-Myc* because of its high thermal stability). DNA concentrations of 20 µM for GQs and 30 µM for *Hairpin* were used. For each compound, a 10 mM stock solution in 100% DMSO was prepared. Oligonucleotide/compound mixtures were obtained by adding 2 molar equiv. of each compound (1 molar equiv. in the case of *c-Myc*) to the folded DNA structures. CD spectra were acquired at 20 and 100 °C, in the 230–320 nm wavelength range, averaged over three scans and subtracted of the buffer baseline. The scanning speed was set to 100 nm/min, with a 0.5 s response time, and 1 nm bandwidth. CD melting experiments were carried out in the 20–100 °C temperature range, with 1 °C/min heating rate, by following changes in the CD signal at the wavelengths of the maximum CD intensity (*i.e.* 263 nm for *c-Kit1* and *c-Myc*, and 290 nm for *Tel_23_*). As for *Hairpin*, CD melting curves were recorded by following the changes in the CD signal at 252 nm (the wavelength of the minimum CD intensity). CD melting curves were normalised between 0 and 1. The apparent melting temperatures (*T*_1/2_) were calculated by using the curve fitting function in OriginPro 2021 software (OriginLab Corp., Northampton, MA, USA). The Δ*T*_1/2_ values correspond to the difference in the *T*_1/2_ values of the nucleic acid structures in the presence and absence of the compounds. All experiments were performed in duplicate, and the reported values are the mean of the two measurements.

### Förster resonance energy transfer (FRET) melting experiments

Measurements were carried out on an FP-8300 spectrofluorometer equipped with a Peltier temperature controller system (PCT-818). The dual-labelled GQ-forming FAM-[d(AGGGAGGGCGCTGGGAGGAGGG)]-TAMRA sequence (*F-c-Kit1-T)*, supplied by biomers.net GmbH (Ulm, Germany), was used for the experiments without further purification. The oligonucleotide was dissolved in water at 1 mM concentration and then diluted to 1 μM in 5 mM KH_2_PO_4_ buffer (pH 7.0) containing 20 mM KCl. The resulting DNA solution was annealed (by heating at 90 °C for 5 min, and then slowly cooling to room temperature overnight) and stored at 4 °C for 24 h before data acquisition. The experiments were carried out in sealed quartz cuvettes with a path length of 1 cm, by using 0.2 μM GQ-forming oligonucleotide without or with 0.4 μM of each compound, and the unlabelled competitor (*Hairpin*) at 0, 3, and 10 μM final concentrations. FRET melting experiments were performed in the range 15–90 °C, with 0.2 °C/min heating rate, by monitoring the emission of FAM at 520 nm (upon excitation at 492 nm). Both excitation and emission slits were set to 5 nm. The emission intensity of FAM was then normalised between 0 and 1. Fluorescence emission spectra were acquired before (at 15 °C) and after (at 90 °C) the melting assay, in the range 500–650 nm, with a 100 nm/s scan speed. Data analysis was performed using OriginPro 2021 software.

### X-ray crystallography

The human telomeric DNA sequence d[AG_3_(T_2_AG_3_)_3_T] (*Tel_23_*) was purchased from Metabion as lyophilised material. Analytical grade reagents and ultra-pure water obtained through a Millipore S.A0.670120 Mosheim apparatus were employed. Representative compounds from the two subseries were dissolved in DMSO to a total 10 mM concentration, while the *Tel_23_* sequence was dissolved in 20 mM potassium cacodylate pH 6.5 and 50 mM KCl up to a concentration 1 mM and annealed to allow GQ formation by heating to 90 °C for 15 min and then slowly cooling to rt overnight. Each DMSO stock solution was added to the annealed DNA solution in 1:1 ligand: DNA molar ratio. Crystallisation trials were set up by mixing 1 μL DNA–drug complex solution with 1 μL crystallisation solution. Crystals were obtained for all the tested compounds in several crystallisation conditions and screened by Single Crystal X-ray Diffraction (SC-XRD) analysis. Unfortunately, crystals were generally affected by twinning and/or pseudosymmetry phenomena. Only the **29**/*Tel_23_* adduct gave crystals in suitable quality to proceed with the SC-XD analysis. Thus, good crystals were obtained at 296 K using the sitting drop vapour diffusion method from a solution containing 0.3 M KCl, 0.05 M potassium cacodylate buffer, pH = 6.5, and 10% v/v MPD^82^. Drops were equilibrated against the same solution (100 μL). The **29**/*Tel_23_* complex crystallises in the monoclinic system, space group C2 (*a* = 36.842 Å, *b* = 72.717 Å, *c* = 27.028 Å, β = 90.10°). Data collection was performed using synchrotron light (λ = 1.0000 Å, XRD-2 Beamline, Elettra Trieste). Data was collected at 100 K, using the crystallisation solution added with MPD up to 30% v/v as cryoprotectant. Data was integrated and scaled using the program XDS^83^. The structure was solved by the Molecular Replacement technique using the program MolRep and the coordinates of the Tel_24_ GQ structure PDB 6H5R^60^, without all the heteroatoms, as a search model. The model was refined with the program Refmac5 ^84^ from the CCP4 program suite^85^. Manual rebuilding of the model was performed using the program Coot^86^. For the T23 residue, only the phosphate group was clearly visible in the electron density map, thus only those coordinates were refined. Data collection information and final refinement statistics are reported in Table S3, Supporting Information. Final coordinates and structure factors have been deposited with the Protein Data Bank (PDB accession number 7PNL).

### Biology

HeLa cells were purchased from American Type Culture Collection (ATCC; Manassas, VA, USA). Dulbecco’s Modified Eagle’s Medium (DMEM), Dulbecco’s Phosphate Buffered Saline (PBS), foetal bovine serum (FBS), penicillin, and streptomycin were provided by Euroclone S.p.a. (Milan, Italy). Dimethyl sulfoxide (DMSO), 4% paraformaldehyde, Triton X-100, and bovine serum albumin (BSA) were supplied by HiMedia (Pennsylvania, USA). The following antibodies were used for the immunofluorescence (IF) experiments: Anti-DNA G-quadruplex structures, clone BG4 (Merck-Millipore, Prague, Czech Republic, #MABE917); rabbit anti-FLAG antibody (Merck, St. Louis, MO, USA, #F7425); donkey anti-rabbit IgG (H + L) highly cross-adsorbed secondary antibody (Alexa Fluor 488 Conjugate) (Thermo Fisher Scientific, Waltham, MA, USA, #A-21206). The Hoechst 33 258 solution, Mowiol 4–88, Cell Proliferation Kit (MTT), and RHPS4 were purchased from Merck (St. Louis, MO, USA). The cobalt (II) chloride hexahydrate (CoCl_2_) powder was provided by Merck (St. Louis, MO, USA).

### Cell Culture

HeLa cells were grown in Dulbecco’s Modified Eagle’s Medium supplemented with 10% FBS, 100 U/mL penicillin, and 100 μg/mL streptomycin, at 37 °C, in a 5% CO_2_/95% air atmosphere. Cells were sub-cultured at 90% confluence, in the split ratio 1:8, every 3–5 days. The MTDLs used in this study, as well as the CAIs **S** and **C**, were dissolved in DMSO at 40 mM (for the sulphonamides) or 10 mM (for the coumarins) concentration. As for berberine, a 50 mM stock solution in DMSO was prepared. Each tested compound was diluted in cell culture medium to the required concentration, immediately prior to use. As regards the experiments under hypoxic conditions, an 80 mM stock solution of CoCl_2_ was prepared in sterile distilled water and further diluted in medium to obtain the final desired concentrations.

### MTT assay

For the MTT experiments in the presence of normal oxygen levels, HeLa cells were seeded in 96-well plates at a density of 15,000 cells per well (culture medium volume – 100 μL/well) and incubated at 37 °C, for 24 h. The medium was then removed, and HeLa were incubated, for 48 h, with fresh medium containing increasing concentrations of MTDLs, single-target-directed agents (berberine, **S**, and **C**) or vehicle (0.5% DMSO). The MTDL **C-4,2** was also tested for 12 h. As for the co-treatments, cells were co-treated for 12 h with equimolar concentrations of berberine and **C**. As regards the MTT experiments under hypoxic conditions, HeLa cells were seeded in 96-well plates (8,000 cells per well) and incubated overnight, pre-treated with 50 μM cobalt (II) chloride hexahydrate (CoCl_2_) and then, for the following 12 h, either exposed to increasing concentrations of **C-4,2** or vehicle (0.5% DMSO), or co-treated with equimolar concentrations of berberine and **C**. Each condition was assayed in triplicate. At the end of the treatments, 10 µL of MTT reagent were added to each well, at a final concentration of 0.5 mg/mL, and the plates were incubated at 37 °C for 4 h. To dissolve the resulting purple formazan crystals, 100 μL of the solubilisation solution were added into each well and the plates were allowed to stand overnight in the incubator (37 °C, 5% CO_2_/95% air). Finally, the optical density of the samples (at 570 nm) was measured on an ELx800 Absorbance Microplate Reader (BioTek Instruments, Inc., Winooski, VT, USA). The percentage (%) of cell survival for each condition was calculated by using the following equation:

$$\text{\% cell survival}=\frac{\text{OD}-\;{\text{OD}}_{0}}{{\text{OD}}_{\text{CONTROL}}-\;{\text{OD}}_{0}}\times 100$$

where OD is the optical density of the sample, OD_0_ is the optical density of the background signal, and OD_CONTROL_ is the optical density of the control sample (cells treated with vehicle, 0.5% DMSO). The concentration able to reduce by 50% the cellular viability (IC_50_) was calculated, for each investigated compound, using Prism 8.0.2 (GraphPad, San Diego, CA, USA) and plotting the percentage of cell survival versus the compound concentration (µM). The statistical details of all experiments are reported in the figure legends.

### Immunofluorescence studies

For the IF experiments in the presence of normal oxygen levels, HeLa cells were seeded on sterile coverslips, in a 24-well plate, at a density of 90,000 cells per well, and incubated overnight. Then, cells were treated with a sublethal concentration of each investigated MTDL or an equivalent amount of vehicle (0.1% DMSO), for 24 h. As positive control, cells were exposed for 24 h to 2 µM RHPS4. As regards the IF experiments under hypoxic conditions, HeLa were seeded on sterile coverslips, in a 24-well plate, at a density of 50,000 cells per well, and incubated overnight. After that, cells were pre-treated with 50 μM cobalt (II) chloride hexahydrate for 60 h to induce hypoxia and then exposed to a non-toxic concentration of **C-4,2** or vehicle (0.1% DMSO) for the following 12 h, still in the presence of CoCl_2_. At the end of the treatments, HeLa were fixed in 4% paraformaldehyde, at RT, for 10 min, and permeabilized in 0.1% Triton X-100 in PBS, at RT, for 10 min. Non-specific binding sites were blocked with 5% BSA in PBS, at RT for 30 min. Cells were then incubated with 1:100 BG4 antibody, at rt for 1 h, rinsed three times with PBS, and incubated with 1:1000 rabbit anti-FLAG antibody, at rt for 1 h. Following three rinsing steps with PBS, cells were incubated with 1:500 donkey anti-rabbit IgG (H + L) highly cross-adsorbed secondary antibody (Alexa Fluor 488 Conjugate), at RT for 1 h, and then rinsed again three times with PBS. Finally, nuclei were stained with the Hoechst 33 258 solution and each coverslip was mounted on a microscope slide with Mowiol 4–88. Z-stacks of three planes were acquired by means of confocal microscopy (Zeiss LSM 980, Plan-Apochromat 63×/1.4 NA oil objective). For the image analysis, a maximum intensity projection of each z-stack was generated. From there, the cellular and nuclear foci were segmented, and their number or fluorescence intensity was quantified by using the Image Analysis package of ZEISS ZEN 3.1 software. The statistical details of all experiments are reported in the figure legends.

## Conclusions

We proposed here MTDLs hitting two promising anticancer targets, GQ structures and hCAs IX and XII, to disrupt cancer cell growth at multiple levels. Particularly, we synthesised a series of MTDLs containing both a berberine scaffold, as GQ stabiliser, and a sulphonamide or coumarin hCAs IX and XII inhibitor moiety, connected by 1,2,3-triazolyl linkers. A subset of sulphonamide derivatives exhibited 0.2 to 0.7 nM inhibition constants against hCA IX, K_I_s in a low-medium nanomolar range (2.9–31.2 nM) against hCA XII, and a up to two orders of magnitude selectivity for the target isozymes over the off target hCAs I and II. Low-medium nanomolar inhibition of hCAs IX and XII was instead measured for the coumarin-based MTDLs (K_I_s in the ranges 9.6–66.0 nM and 4.2–30.1 nM, respectively), but with a complete selectivity over hCAs I and II (K_I_s > 100 µM). CD melting experiments revealed that these MTDLs are generally more effective than berberine in stabilising the parallel GQ structures adopted by *c-Kit1* and *c-Myc* (Δ*T*_1/2_ ≥ 19.0 °C) over the hybrid one formed by *Tel_23_*(Δ*T*_1/2_ ≈ 10.0). Interestingly, the thermal stability of *Hairpin* double-stranded DNA was not affected by most of the compounds (Δ*T*_1/2_ ≤ 2.5 °C). FRET melting analyses confirmed that the MTDL-induced stabilising effects were not significantly influenced by the presence of the *Hairpin* competitor in solution, indicating a highly selective GQ-binding behaviour. Moreover, the first crystal structure of a GQ in adduct with a MTDL was reported, that is *Tel_23_* in complex with the sulphonamide **S-3,2**, which adopts two distinct binding conformations over the GQ guanine-tetrad.

Importantly selected MTDLs exhibited a significant anti-proliferative action against CA IX-positive HeLa cancer cells, which is greater than that of the co-exposure to the equimolar single structural components (berberine and CAI) resulting from the dual targeting of both CAs and GQs. Of note, the cytotoxicity of the coumarin **C-4,2** turned out to be highly significant both in normoxia and hypoxia. Immunofluorescence studies showed that **C-4,2** can also impact the formation of GQ structures in the cellular environment, both under normoxic and hypoxic conditions, remarkably increasing the number of detectable GQs.

The present work, collecting drug-design, synthetic approaches, physico-chemical methodologies, X-ray crystallographic and biological studies, demonstrates for the first time that GQ ligands can be successfully modified to obtain MTDL derivatives with relevant biological properties, paving the way to multi-targeting purposes in the field of GQs as drug targets.

## Supplementary information

Figures S1-S16, Table S1-S4, ^1^H- and ^13^C-NMR spectra, HPLC traces. The atomic coordinates of the **29**/*Tel_23_* complex have been deposited in the Protein Data Bank with accession code 7PNL.

## Supplementary Material

Supplemental Material
