# Supplementary material for: Development of a multi-targeted chemotherapeutic approach based on G-quadruplex stabilisation and carbonic anhydrase inhibition
Source: J Enzyme Inhib Med Chem. 2024 Jun 18;39(1):2366236. doi: 10.1080/14756366.2024.2366236 (PMC11195807; doi:10.1080/14756366.2024.2366236)
Supplement: Supplemental Material [file IENZ_A_2366236_SM1097.pdf]

**Development of a multi-targeted chemotherapeutic approach based on G-quadruplex stabilization and carbonic anhydrase inhibition.**

Alessio Nocentini,[1†] Anna Di Porzio,[2†] Alessandro Bonardi,[1] Carla Bazzicalupi,[3,]* Andrea Petreni,[1] Tarita Biver,[4] Silvia Bua,[5] Simona Marzano,[2] Jussara Amato,[2] Bruno Pagano,[2] Nunzia Iaccarino,[2] Stefano De Tito,[6] Stefano Amente,[7] Claudiu T. Supuran,[1,]* Antonio Randazzo,[2,]* Paola Gratteri[1]

[1] NEUROFARBA Department, Pharmaceutical and Nutraceutical Section and Laboratory of Molecular Modeling Cheminformatics & QSAR, University of Florence, Via U. Schiff 6, 50019, Sesto Fiorentino, Florence, Italy
[2] Department of Pharmacy, University of Naples Federico II, Via D. Montesano 49, 80131 Naples, Italy
[3] Department of Chemistry "Ugo Schiff", University of Florence, Via della Lastruccia 3-13, 50019, Sesto Fiorentino, Florence, Italy
[4] Department of Chemistry and Industrial Chemistry, University of Pisa, Via G. Moruzzi 13, 56124 Pisa, Italy
[5] Research Institute of the University of Bucharest (ICUB), Bucharest, Romania
[6] Molecular Cell Biology of Autophagy, The Francis Crick Institute, 1 Midland Road, London NW1 1AT, UK
[7]Department of Molecular Medicine and Medical Biotechnologies, University of Naples Federico II, Via Pansini 5, 80131 Naples, Italy
† These authors contributed equally.

**Table of contents**

| | **Page** |
|---|---|
| **Table S1** | S2 |
| **Figure S1** | S3 |
| **Figure S2** | S4 |
| **Figure S3** | S5 |
| **Figure S4** | S6 |
| **Figure S5** | S7 |
| **Figure S6** | S8 |
| **Figure S7** | S9 |
| **Figure S8** | S10 |
| **Table S2** | S11 |
| **Figure S9** | S12 |
| **Table S3** | S13 |
| **Figure S10** | S14 |
| **Figure S11** | S14 |
| **Figure S12** | S15 |
| **Figure S13** | S15 |
| **Table S4** | S15 |
| **Figure S14** | S16 |
| **Figure S15** | S16 |
| **$^{1}$H- and $^{13}$C-NMR spectra** | S17 |
| **HPLC traces** | S59 |

**Table S1.** Selectivity index (*SI*) of the primary sulfonamides **26–33**, **40**, **41**, **47** and coumarin derivatives **34–39**, **42–45**, the reference **AAZ** and **SLC-0111** against the tumor-associated hCA IX and XII isoforms *vs* the ubiquitous cytosolic hCAs I and II.

| **Compound** | | ***SI* (target hCA/off-target hCA)** | | | |
|---|---|---|---|---|---|
| | | **I/IX** | **I/XII** | **II/IX** | **II/XII** |
| **26** | **S-2,0** | 414.6 | 79.5 | 42.4 | 8.1 |
| **27** | **S-2,2** | 1446 | 213.7 | 9.2 | 1.4 |
| **28** | **S-3,0** | 1194 | 62.3 | 91.5 | 4.8 |
| **29** | **S-3,2** | 1437 | 25.8 | 111.3 | 2.0 |
| **30** | **S-4,0** | 1209 | 23.2 | 112.4 | 2.2 |
| **31** | **S-4,2** | 1308 | 127.2 | 102.1 | 9.9 |
| **32** | **S-5,0** | 2390 | 31.3 | 65.8 | 0.9 |
| **33** | **S-5,2** | 406.5 | 75.7 | 76.1 | 14.2 |
| **40** | **S-1,0*m*** | 941.1 | 148.0 | 23.1 | 3.6 |
| **41** | **S-1,0*p*** | 307.1 | 135.9 | 8.5 | 3.8 |
| **47** | **S-3,a** | 4.2 | 2.1 | 1.2 | 0.6 |
| **34** | **C-3,2** | >151.5 | >332.2 | >151.5 | >332.2 |
| **35** | **C-3,3** | >289.9 | >529.1 | >289.9 | >529.1 |
| **36** | **C-3,4** | >169.8 | >352.1 | >169.8 | >352.1 |
| **37** | **C-4,2** | >578.0 | >1190 | >578.0 | >1190 |
| **38** | **C-4,3** | >332.2 | >598.8 | >332.2 | >598.8 |
| **39** | **C-5,2** | >205.8 | >497.5 | >205.8 | >497.5 |
| **42** | **C-1,4** | >284.9 | >980.4 | >284.9 | >980.4 |
| **43** | **C-1,5** | >392.2 | >2381 | >392.2 | >2381 |
| **44** | **C-1,6** | >1041 | >689.7 | >1041 | >689.7 |
| **45** | **C-1,0c** | >194.2 | >1852 | >194.2 | >1852 |
| **AAZ** | | 10 | 43.9 | 0.5 | 2.2 |
| **SLC-0111** | | 112.9 | 1128.9 | 21.3 | 213.3 |

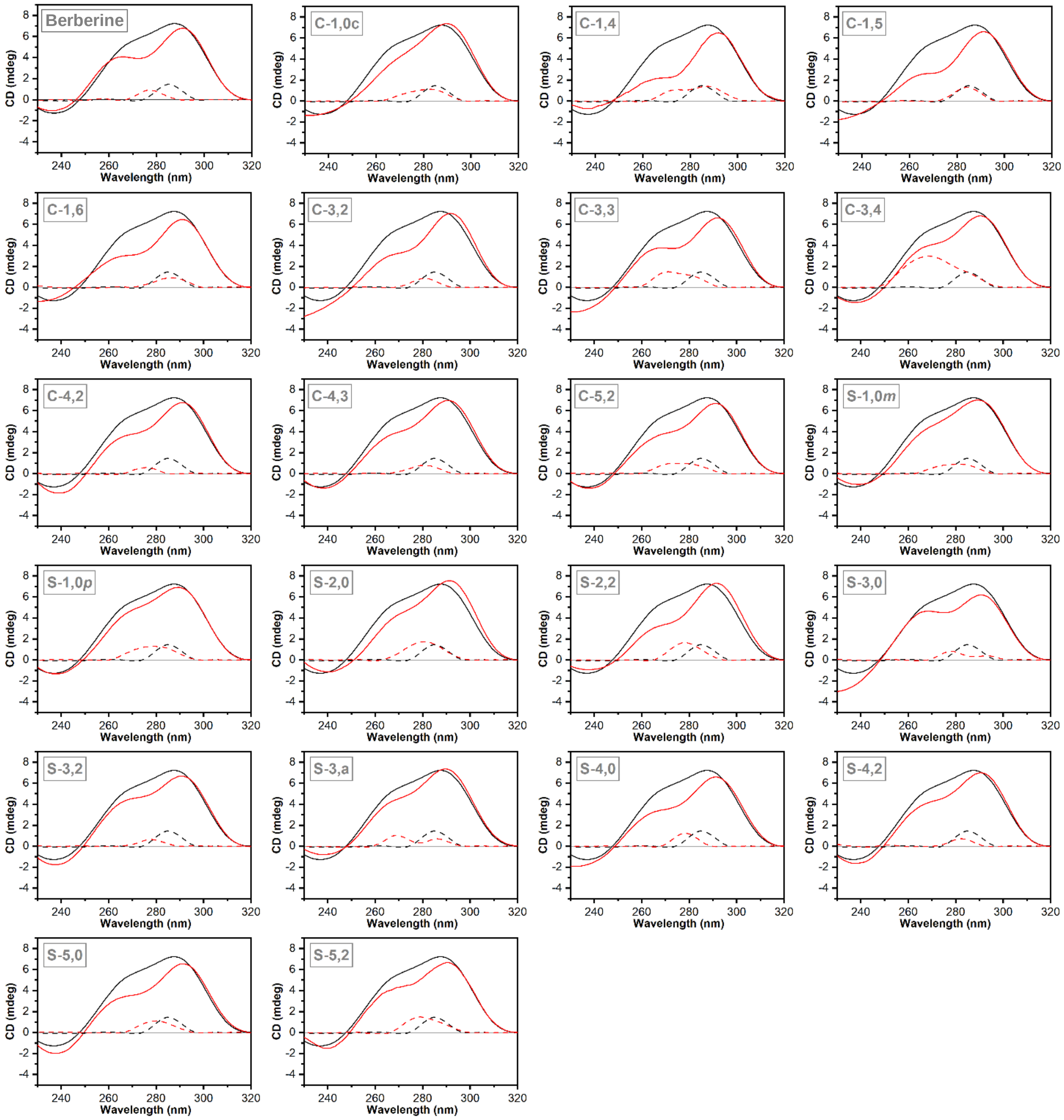


**Figure S1.** CD spectra of *Tel*$_{23}$ G-quadruplex at 20 and 100 °C (solid and dashed lines, respectively) in the absence (black) and presence (red) of 2 molar equiv of the indicated compounds.

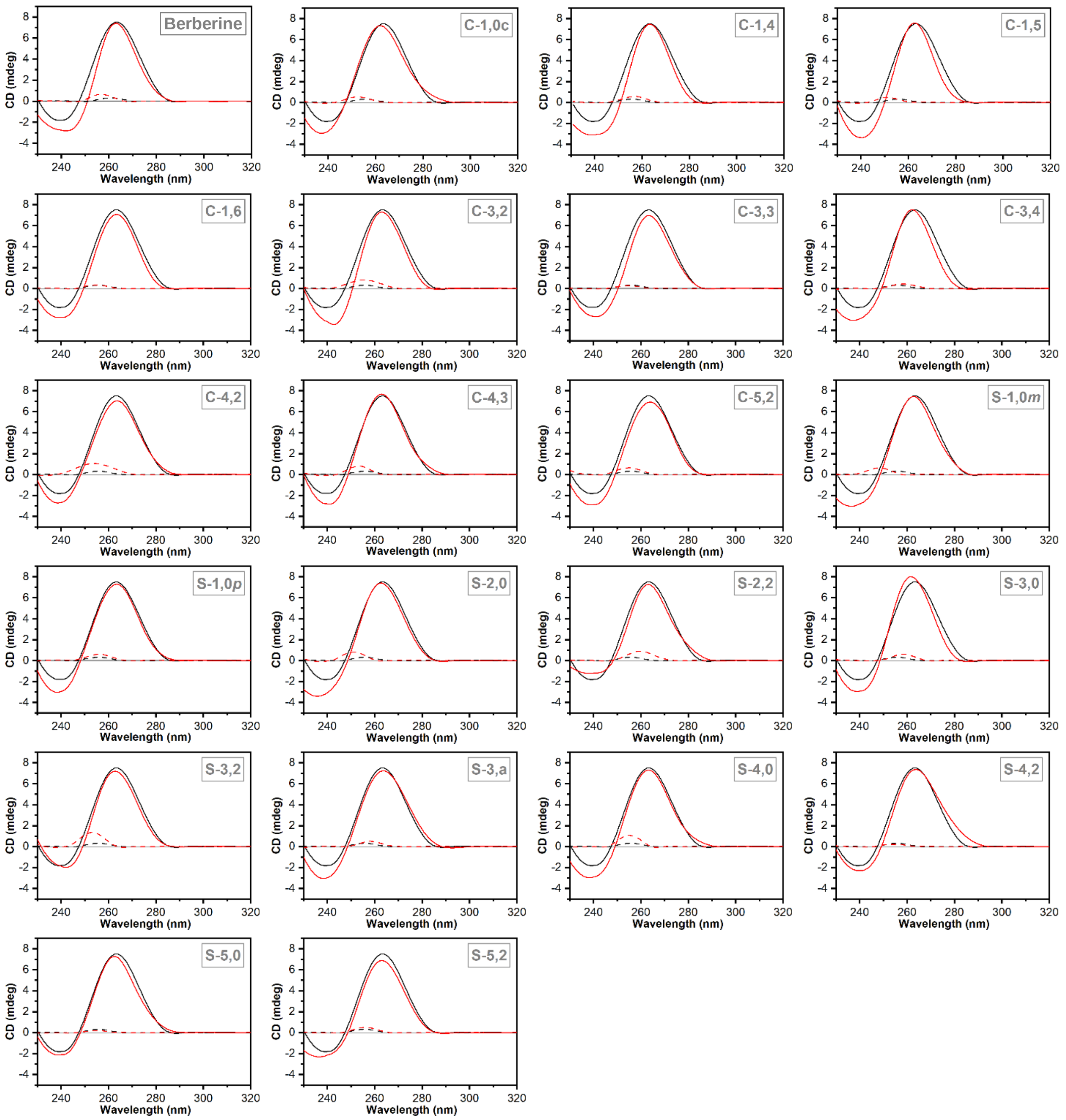


**Figure S2.** CD spectra of *c-Kit1* G-quadruplex at 20 and 100 °C (solid and dashed lines, respectively) in the absence (black) and presence (red) of 2 molar equiv of the indicated compounds.

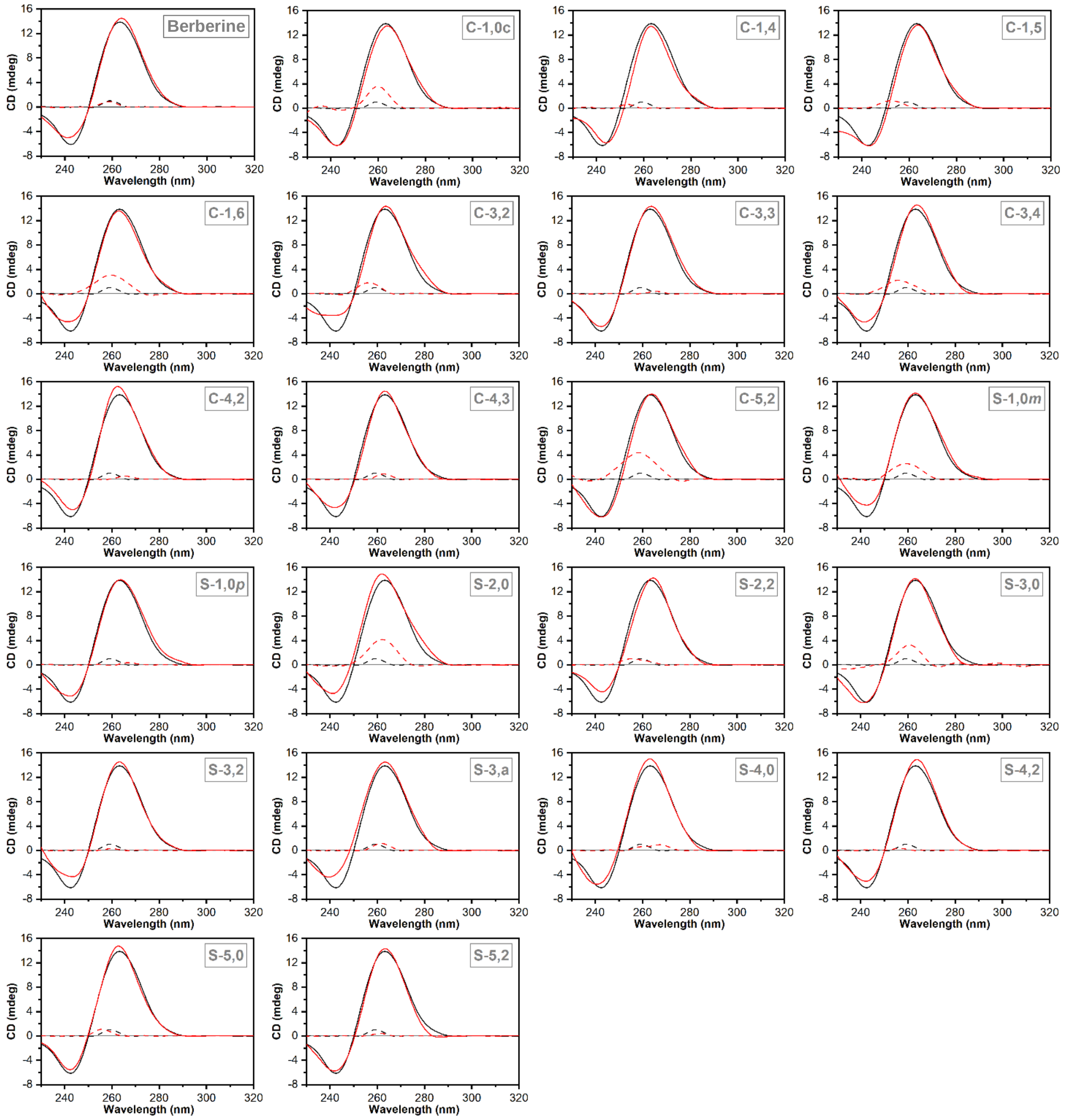


**Figure S3.** CD spectra of *c-Myc* G-quadruplex at 20 and 100 °C (solid and dashed lines, respectively) in the absence (black) and presence (red) of 1 molar equiv of the indicated compounds.

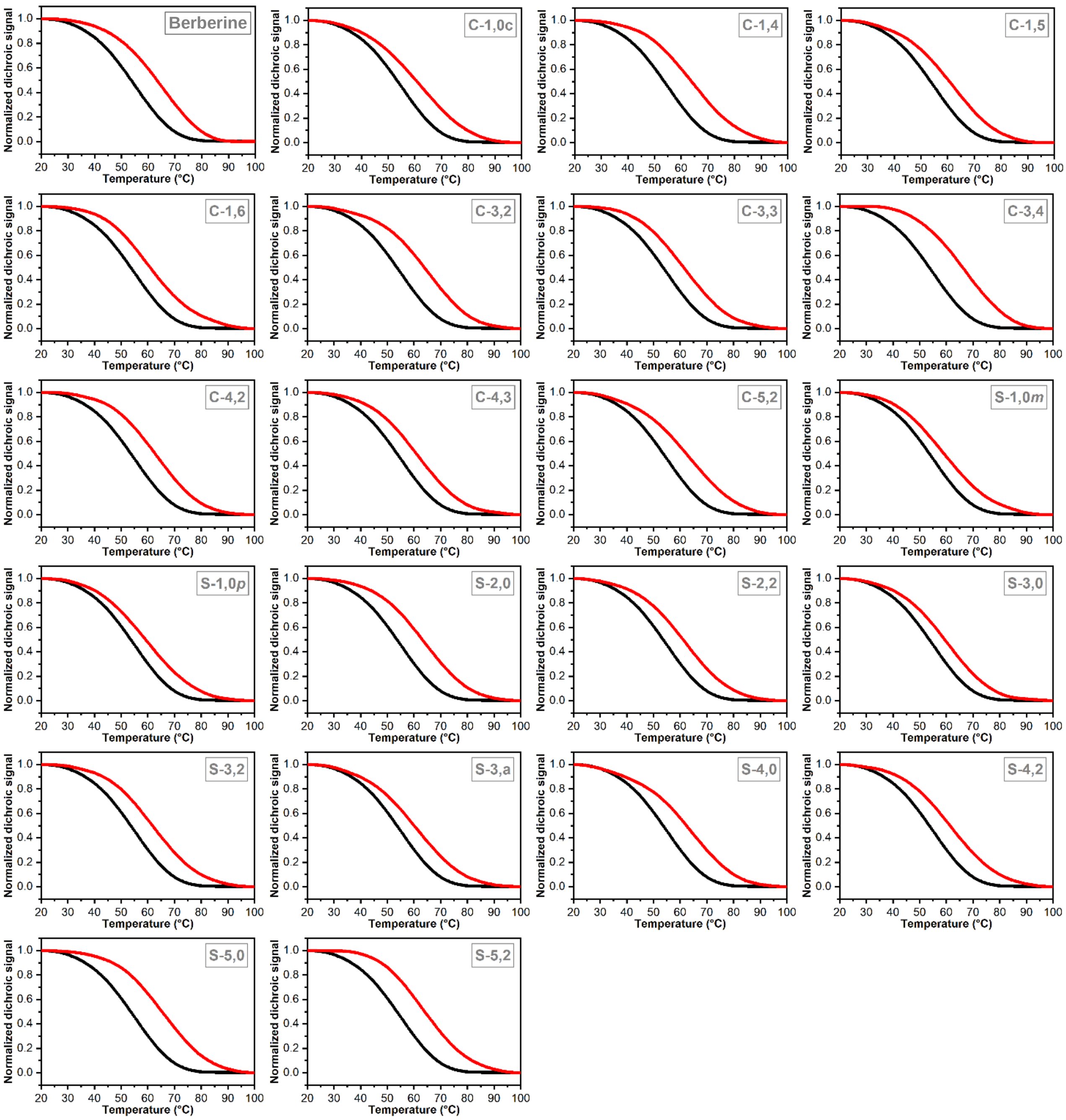


**Figure S4.** Normalized CD melting curves of *Tel*$_{23}$ G-quadruplex in the absence (black lines) and presence (red lines) of 2 molar equiv of the indicated compounds, recorded at 1 °C/min heating rate.

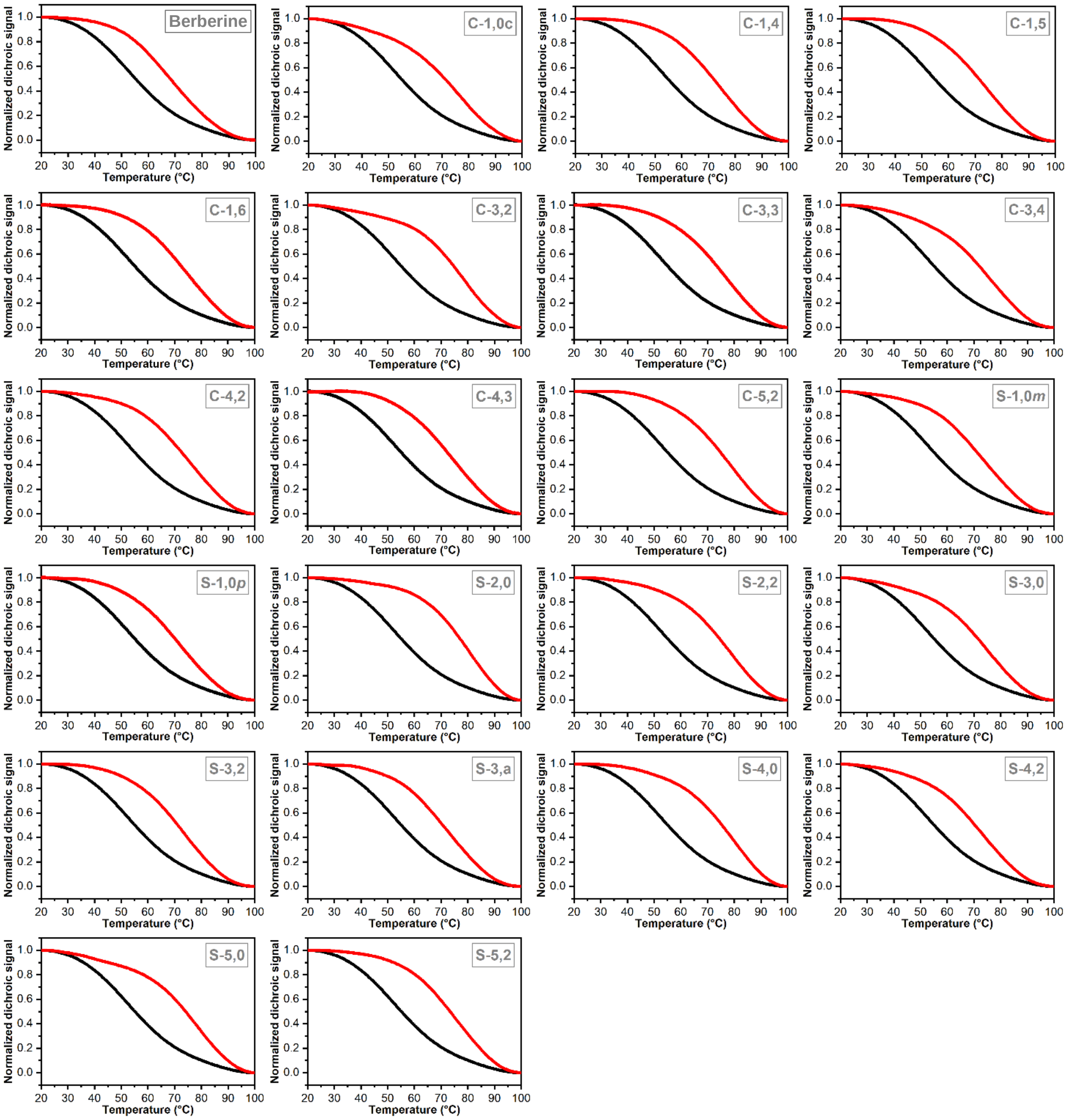


**Figure S5.** Normalized CD melting curves of *c-Kit1* G-quadruplex in the absence (black lines) and presence (red lines) of 2 molar equiv of the indicated compounds, recorded at 1 °C/min heating rate.

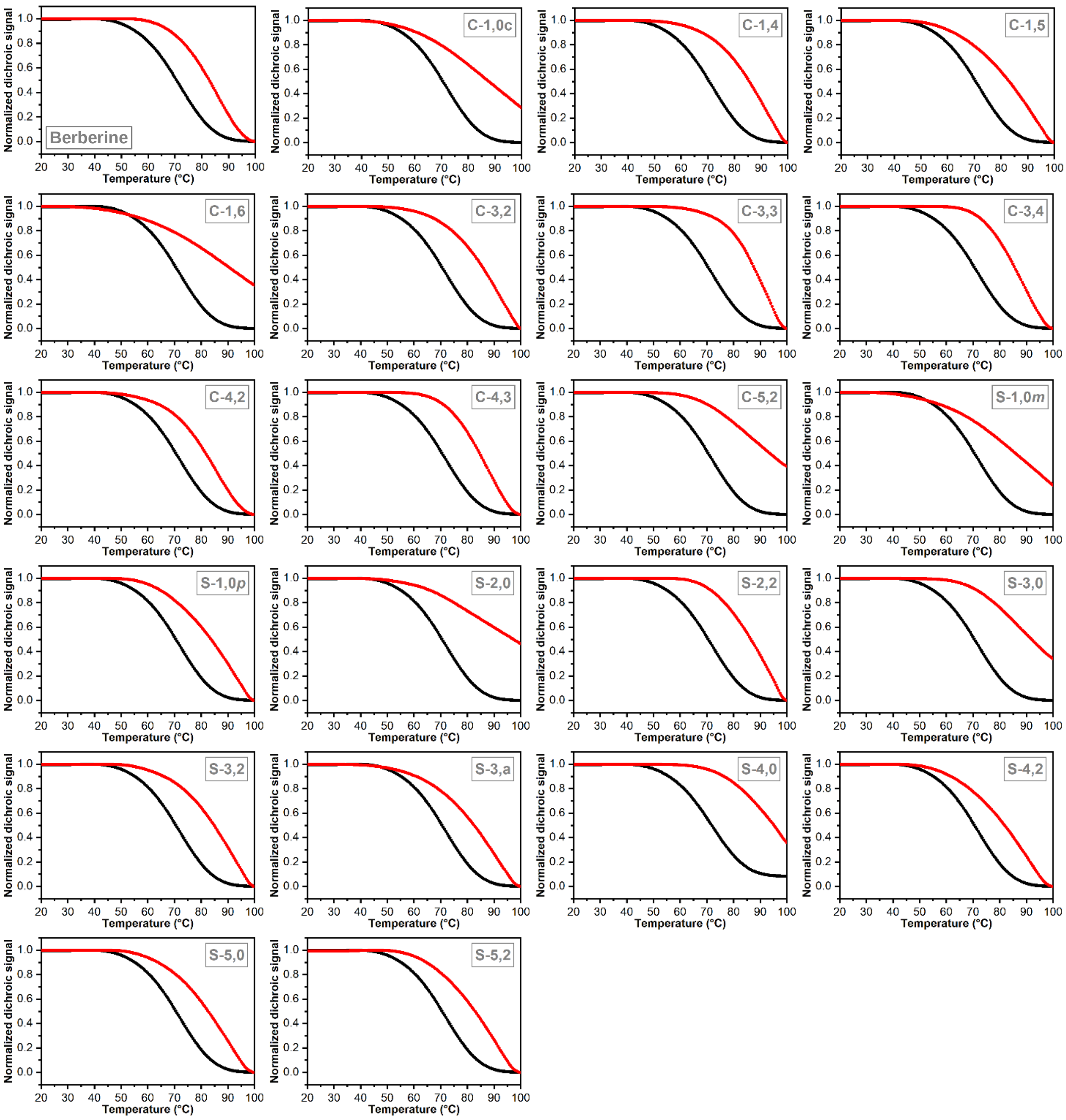


**Figure S6.** Normalized CD melting curves of *c-Myc* G-quadruplex in the absence (black lines) and presence (red lines) of 1 molar equiv of the indicated compounds, recorded at 1 °C/min heating rate.

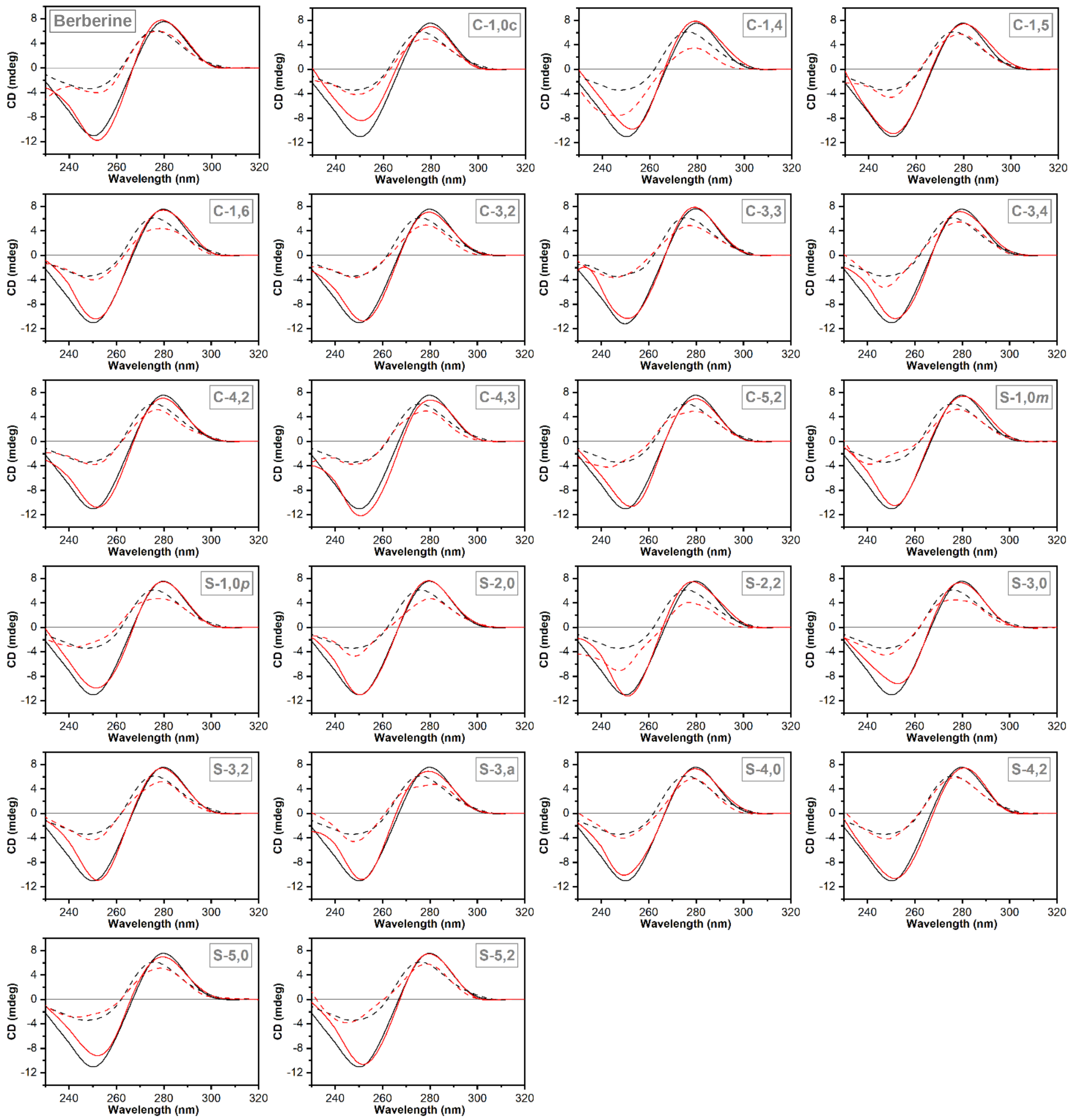


**Figure S7.** CD spectra of *Hairpin* at 20 and 100 °C (solid and dashed lines, respectively) in the absence (black) and presence (red) of 2 molar equiv of the indicated compounds.

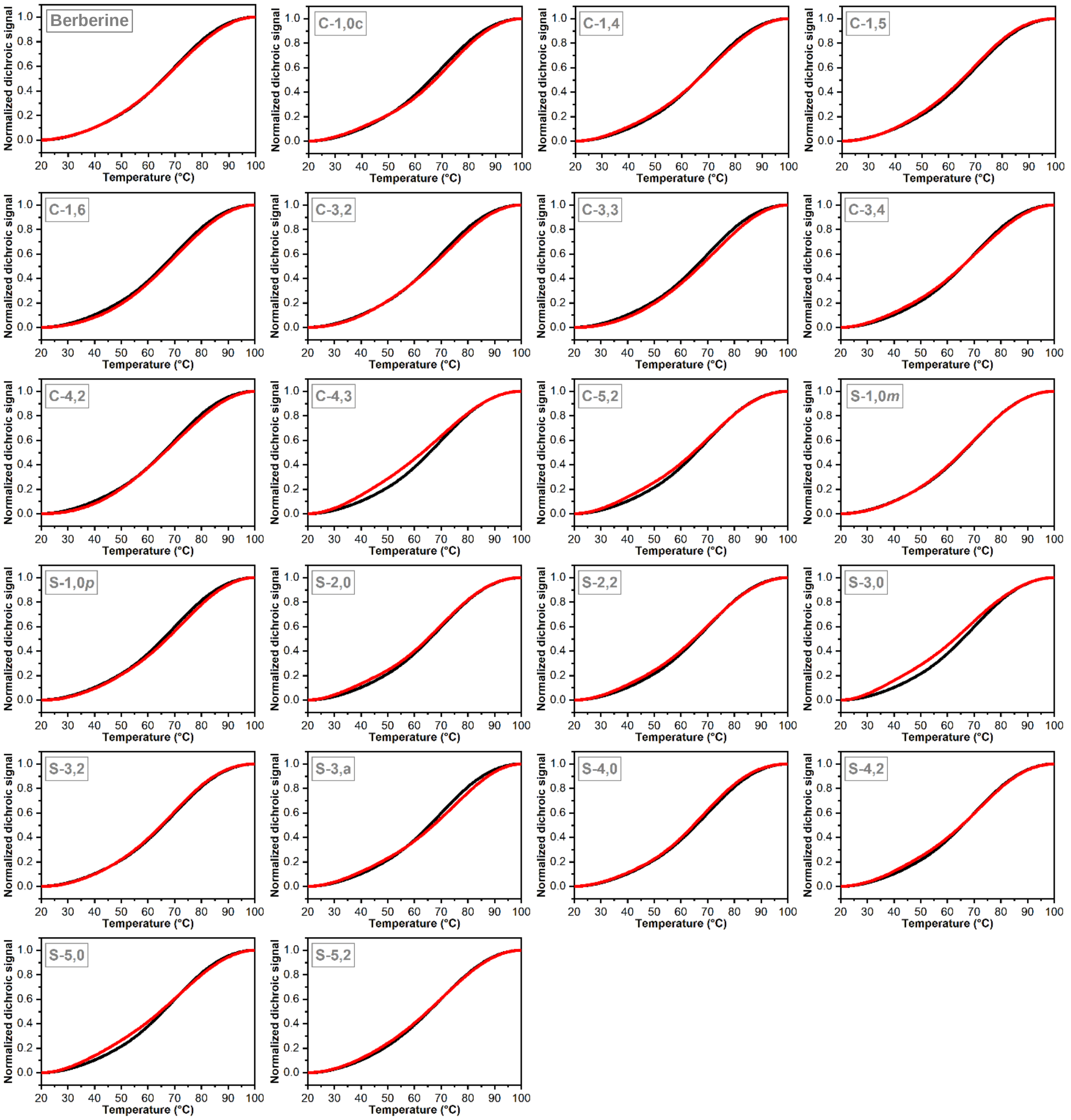


**Figure S8.** Normalized CD melting curves of *Hairpin* in the absence (black lines) and presence (red lines) of 2 molar equiv of the indicated compounds, recorded at 1 °C/min heating rate.

**Table S2.** Compound-induced thermal stabilization of the investigated DNA structures, measured by circular dichroism melting experiments.

| **Compound** | | **Δ$T_{1/2}$ (°C)**[a] | | | |
|---|---|---|---|---|---|
| | | ***Tel23*** | ***c-Kit1*** | ***c-Myc*** | ***Hairpin*** |
| **Berberine** | | 9.5 | 13.0 | 12.0 | 0.2 |
| **26** | **S-2,0** | 10.0 | 22.0 | > 25[b] | -0.5 |
| **27** | **S-2,2** | 7.5 | 18.5 | 18.5 | -0.5 |
| **28** | **S-3,0** | 6.0 | 16.5 | > 25[b] | -2.0 |
| **29** | **S-3,2** | 8.0 | 16.0 | 16.5 | -0.5 |
| **30** | **S-4,0** | 9.0 | 20.5 | > 25[b] | -1.0 |
| **31** | **S-4,2** | 8.0 | 15.5 | 13.0 | 0.2 |
| **32** | **S-5,0** | 12.0 | 19.0 | 13.0 | -1.0 |
| **33** | **S-5,2** | 10.5 | 18.5 | 12.5 | 0.2 |
| **40** | **S-1,0*m*** | 5.5 | 16.0 | > 25[b] | 0.2 |
| **41** | **S-1,0*p*** | 5.5 | 15.0 | 14.5 | 2.5 |
| **47** | **S-3,a** | 6.5 | 15.5 | 12.5 | 2.0 |
| **34** | **C-3,2** | 10.5 | 20.0 | 18.0 | 1.0 |
| **35** | **C-3,3** | 8.0 | 18.5 | 18.5 | 2.0 |
| **36** | **C-3,4** | 12.5 | 17.0 | 15.0 | 0.5 |
| **37** | **C-4,2** | 9.0 | 17.5 | 13.0 | 0.5 |
| **38** | **C-4,3** | 7.5 | 17.5 | 15.0 | -2.0 |
| **39** | **C-5,2** | 8.5 | 20.0 | > 25[b] | -1.0 |
| **42** | **C-1,4** | 10.5 | 17.0 | 16.0 | 0.5 |
| **43** | **C-1,5** | 7.0 | 16.5 | 14.0 | -0.5 |
| **44** | **C-1,6** | 7.5 | 17.5 | > 25[b] | 1.0 |
| **45** | **C-1,0c** | 7.0 | 16.5 | > 25[b] | 2.5 |

[a]Δ$T_{1/2}$ values are the difference in the apparent melting temperature of the DNA in the presence and absence of compounds (2 molar equiv for *Tel23*, *c-Kit1*, and *Hairpin*, 1 molar equiv for *c-Myc*). $T_{1/2}$ values of DNAs alone are: *Tel23* = 53.5 (±0.5) °C; *c-Kit1* = 55.0 (±0.5) °C; *c-Myc* = 70.0 (±0.5) °C; *Hairpin* = 66.0 (±0.5) °C. All experiments were performed in duplicate, and the values reported are the average of two measurements. Errors in Δ$T_{1/2}$ values are within ±1.0 °C.
[b]Δ$T_{1/2}$ could not be accurately determined as the compound hugely increases the thermal stability of GQ.

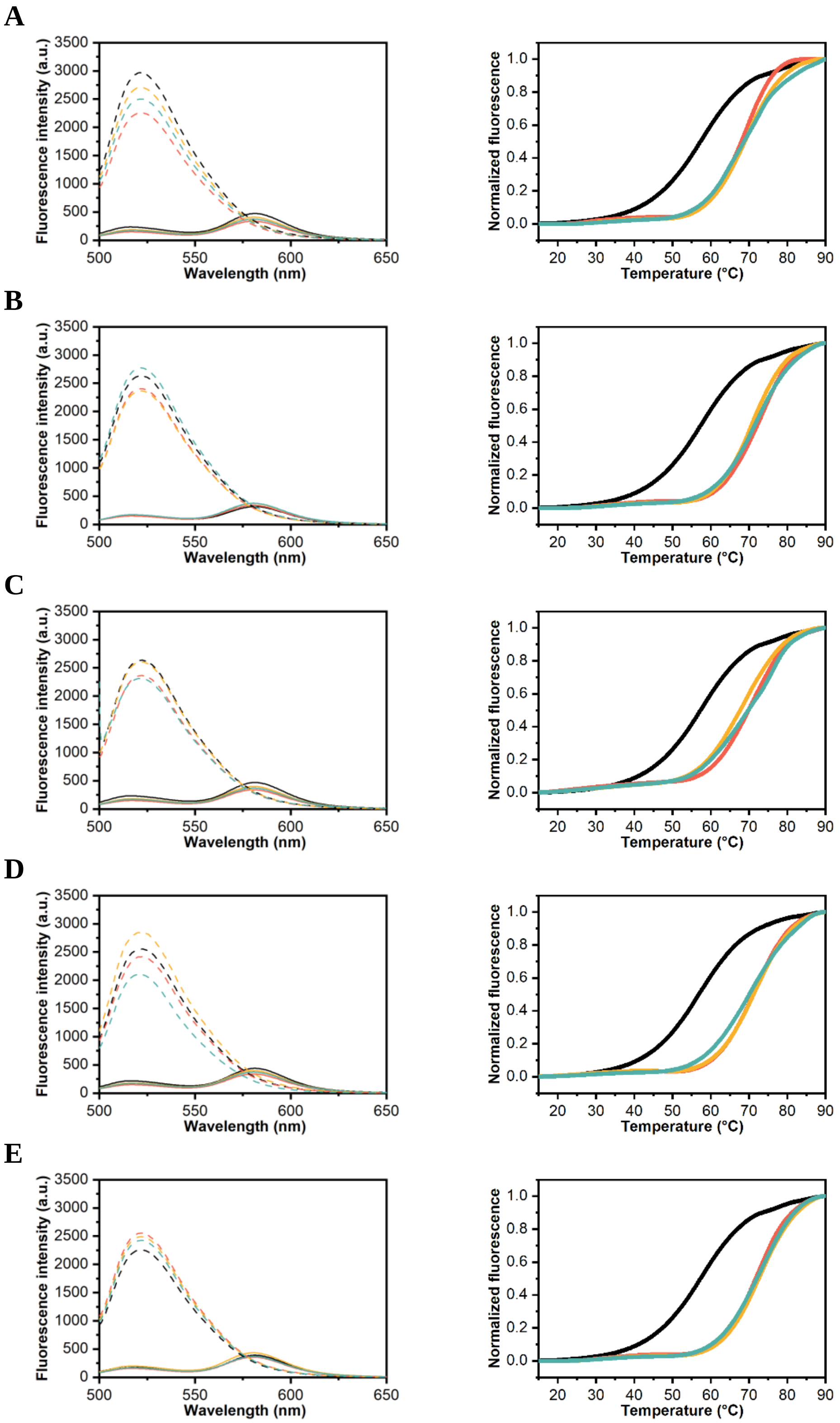


**Figure S9.** Fluorescence emission spectra (left of each panel) recorded at 15 and 90 °C (solid and dashed lines, respectively), and normalized FRET melting curves (right of each panel) for *F-c-Kit1-T* oligonucleotide (0.2 μM) in the absence (black lines) and presence (red lines) of 2 molar equiv of (A) **C-3,2**, (B) **C-5,2**, (C) **S-2,0**, (D) **S-4,0**, and (E) **S-5,0**. Experiments in the presence of compounds were also performed in the presence of large excesses of *Hairpin* duplex competitor (15 and 50 molar equiv with respect to G-quadruplex, yellow and green lines, respectively).

**Table S3.** Summary of data collection and atomic model refinement statistics. Values in parentheses are for the highest resolution shell.

| Parameter | 29 / $Tel_{23}$ |
|---|---|
| Wavelength (Å) | 1.00000 |
| Space group | C2 |
| Cell dimension (Å, deg) | *a* = 36.842 *b* = 72.717 *c* = 27.028<br>α = γ = 90.00 β = 90.10 |
| Limiting resolution (Å) | 36.36 – 1.83 |
| Unique reflections | 6268 |
| $R_{sym}$ (%) | 4.1 |
| Multiplicity | 6.3 |
| Completeness overall (%) | 99.24 |
| <I/σ(I)> | 19.28 |
| CC (1/2) | 99.7 |
| Resolution range (Å) | 36.36 – 1.83 |
| Unique reflections, working/free | 5913 / 336 |
| Rfactor (%) | 20.4 |
| Rfree (%) | 32.1 |
| rmsd bonds(Å) | 0.008 |
| rmsd angles (°) | 2.138 |

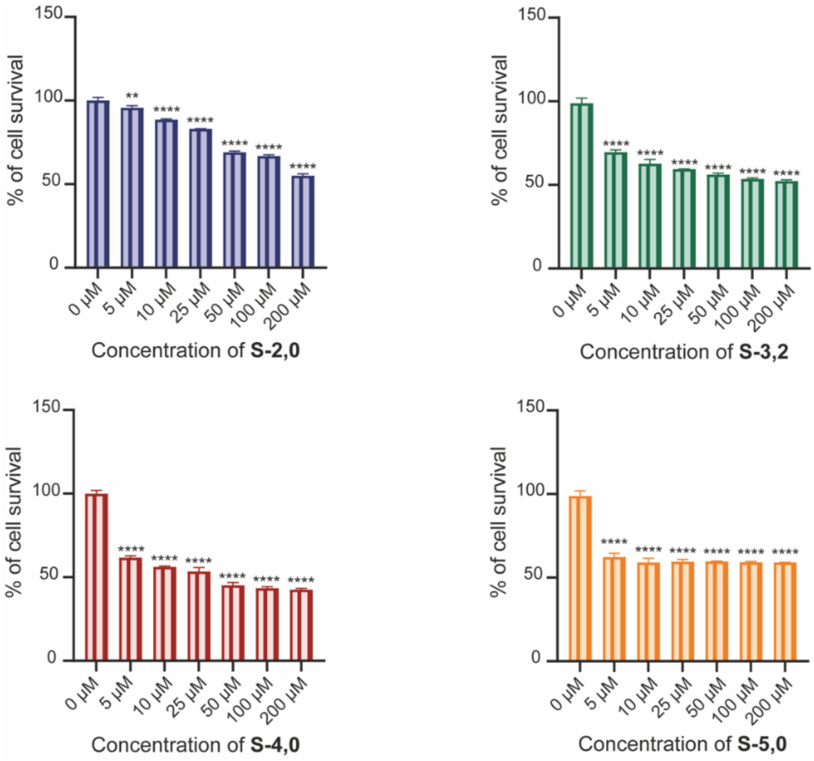


**Figure S10.** Percentage of survival of HeLa cells treated, for 48 h, with increasing concentrations (5–200 µM) of MTDLs containing a CAI scaffold of type **S**. Histograms show the mean ± SEM of three independent experiments. Differences in mean values between each treatment and the control group were determined by a one-way ANOVA test on GraphPad Prism 8.0.2 (**: *p*<0.01, ****: *p*<0.0001).

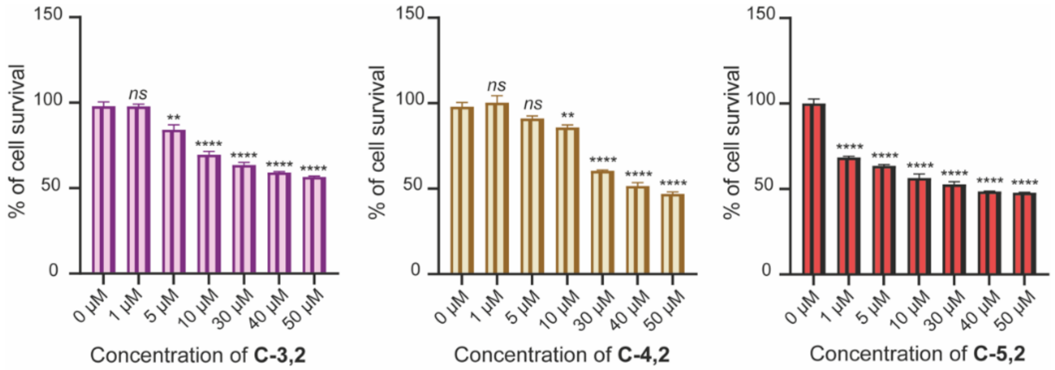


**Figure S11.** Percentage of survival of HeLa cells treated, for 48 h, with increasing concentrations (1–50 µM) of MTDLs containing a CAI scaffold of type **C**. Histograms show the mean ± SEM of three independent experiments. Differences in mean values between each treatment and the control group were determined by a one-way ANOVA test on GraphPad Prism 8.0.2 (*ns*: not significant, **: *p*<0.01, ****: *p*<0.0001).

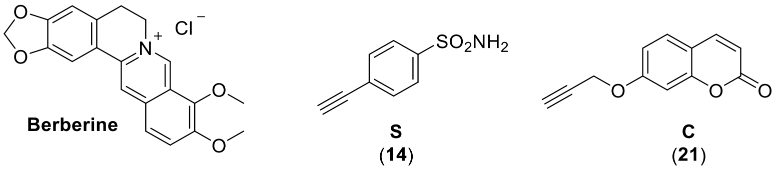


**Figure S12.** Chemical structure of the single-target-directed agents: berberine, **S**, and **C**.

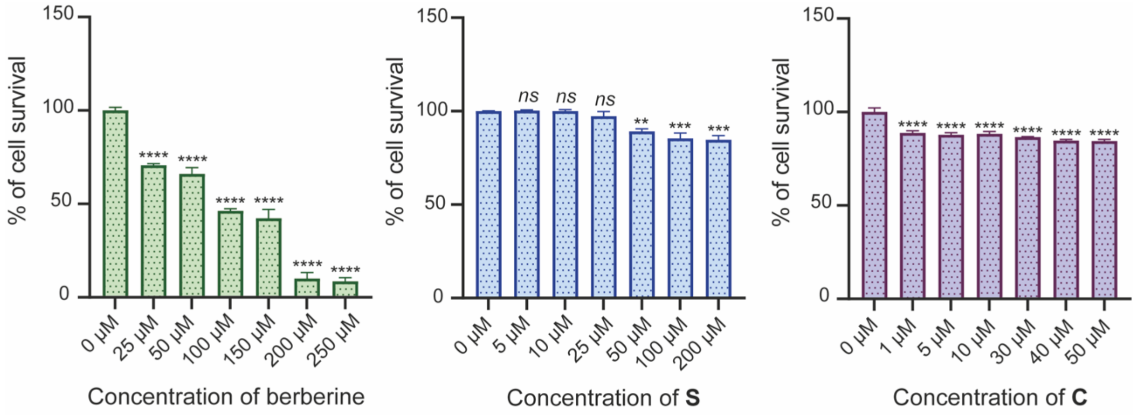


**Figure S13.** Percentage of survival of HeLa cells treated, for 48 h, with berberine (25–250 µM), **S** (5–200 µM) or **C** (1–50 µM). Histograms show the mean ± SEM of three independent experiments. Differences in mean values between each treatment and the control group were determined by a one-way ANOVA on GraphPad Prism 8.0.2 (*ns*: not significant, **: $p<0.01$, ***: $p<0.001$, ****: $p<0.0001$).

**Table S4.** Concentrations of the single-target-directed agents required to give 50% of cell viability reduction ($IC_{50}$ values), by means of the MTT assay, after 48 h of treatment.

| **Single-target-directed agent** | **$IC_{50}$ (µM)** | **95% confidence interval (µM)** |
|---|---|---|
| Berberine | 75.0 | 59.0–91.7 |
| **S** | > 200 | – |
| **C** | > 50 | – |

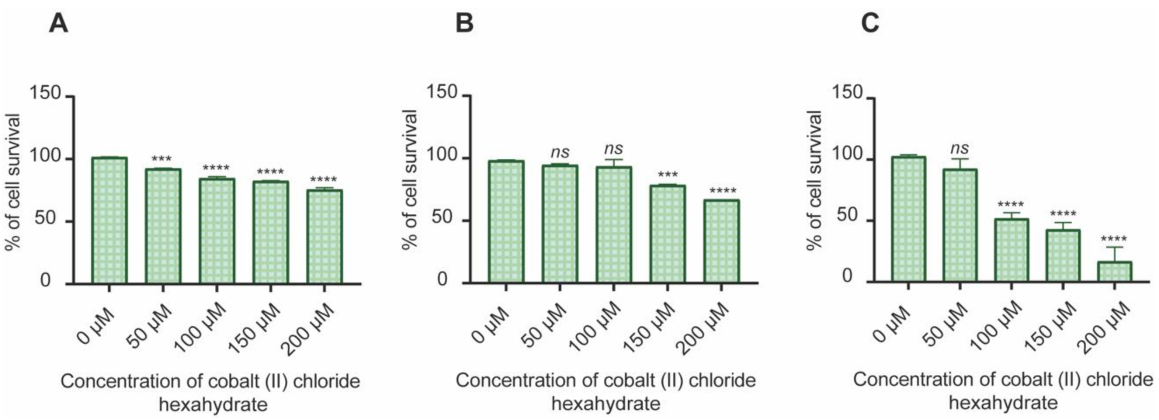


**Figure S14.** Percentage of survival of HeLa cells treated with increasing concentrations of cobalt (II) chloride hexahydrate (50–200 µM) for (A) 24 h, (B) 48 h, and (C) 72 h. Histograms show the mean ± SEM of three independent experiments. Differences in mean values between each treatment and the control group were determined by a one-way ANOVA on GraphPad Prism 8.0.2 (*ns*: not significant, ***: $p$<0.001, ****: $p$<0.0001).

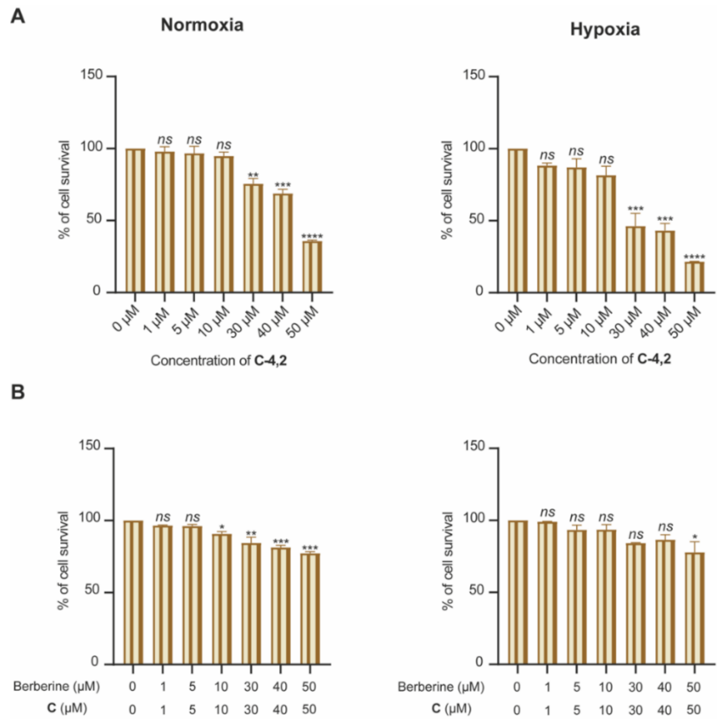


**Figure S15.** Percentage of survival of HeLa cells (A) treated with **C-4,2** (1–50 µM) and (B) co-treated with equimolar concentrations of berberine and **C** (1–50 µM), for 12 h, under normoxic and hypoxic conditions. Histograms show the mean ± SEM of three independent experiments. Differences in mean values between each treatment and the control group were determined by a one-way ANOVA test on GraphPad Prism 8.0.2 (*ns*: not significant, *: $p$<0.05, **: $p$<0.01, ***: $p$<0.001, ****: $p$<0.0001).

$^{1}$H-NMR **26**

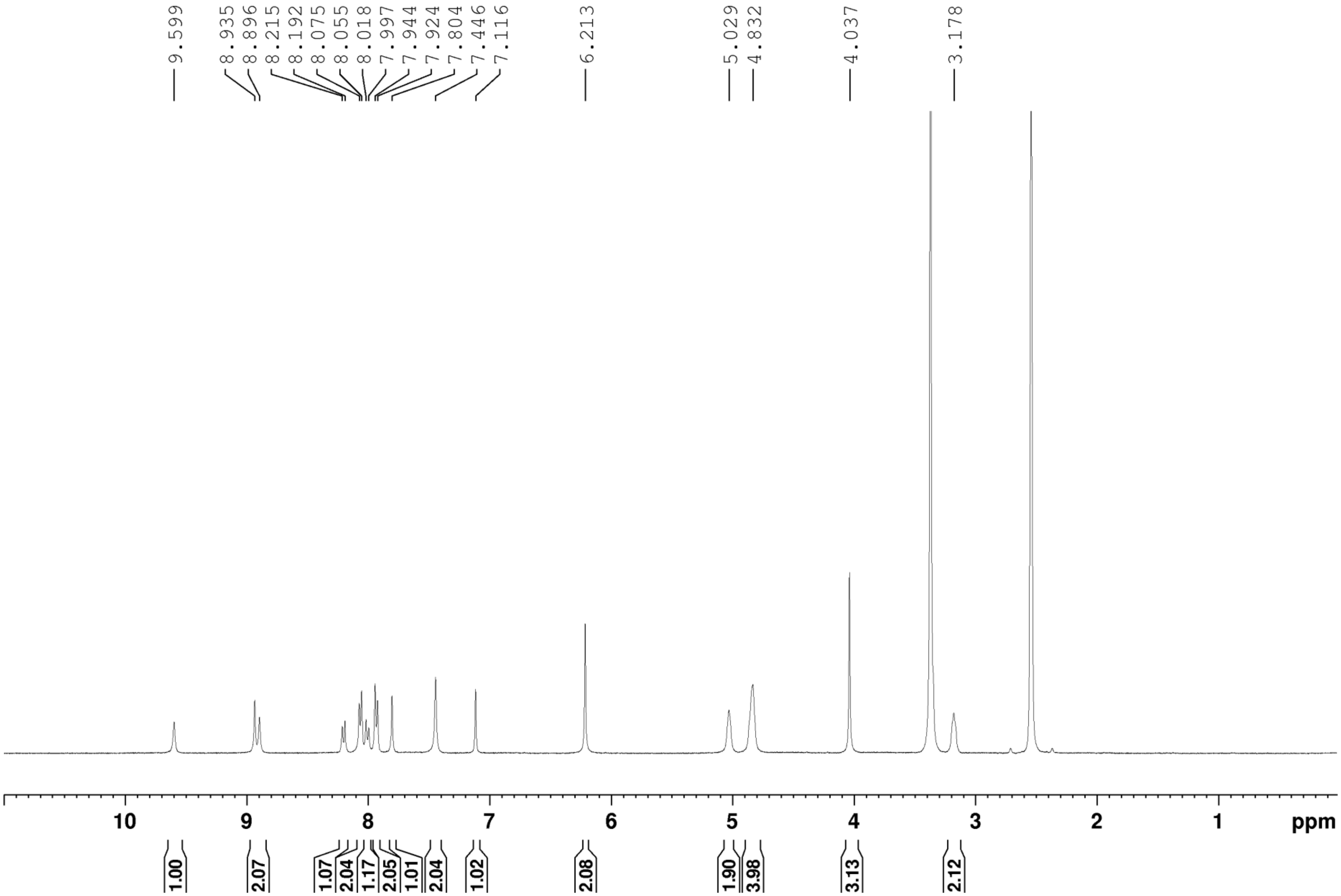

9.599
8.935
8.896
8.215
8.192
8.075
8.055
8.018
7.997
7.944
7.924
7.804
7.446
7.116
6.213
5.029
4.832
4.037
3.178
10
9
8
7
6
5
4
3
2
1
ppm
1.00
2.07
1.07
2.04
1.17
2.05
1.01
2.04
1.02
2.08
1.90
3.98
3.13
2.12

$^{13}$C-NMR **26**

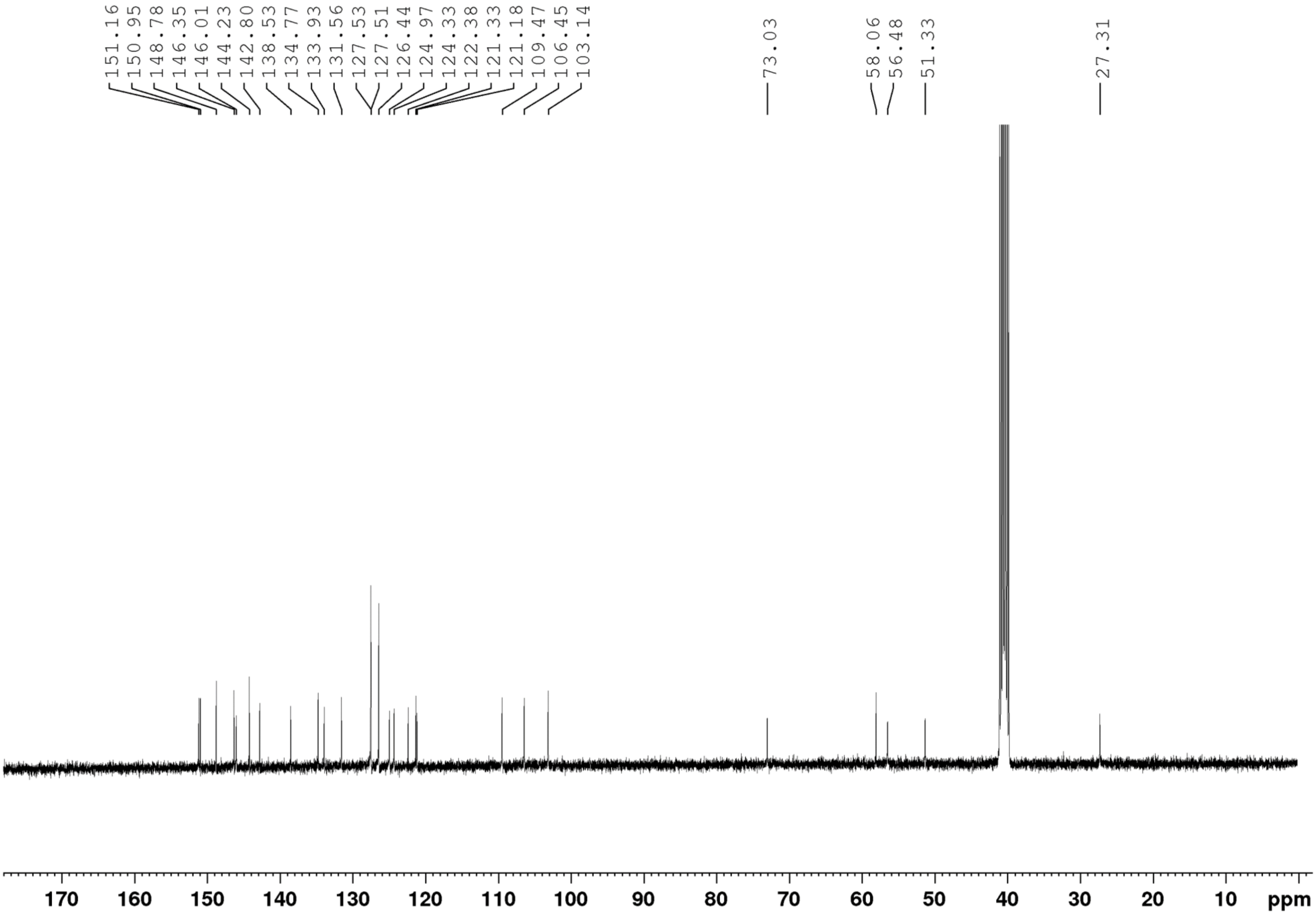
151.16
150.95
148.78
146.35
146.01
144.23
142.80
138.53
134.77
133.93
131.56
127.53
127.51
126.44
124.97
124.33
122.38
121.33
121.18
109.47
106.45
103.14
73.03
58.06
56.48
51.33
27.31
170
160
150
140
130
120
110
100
90
80
70
60
50
40
30
20
10
ppm

$^{1}$H-NMR **27**

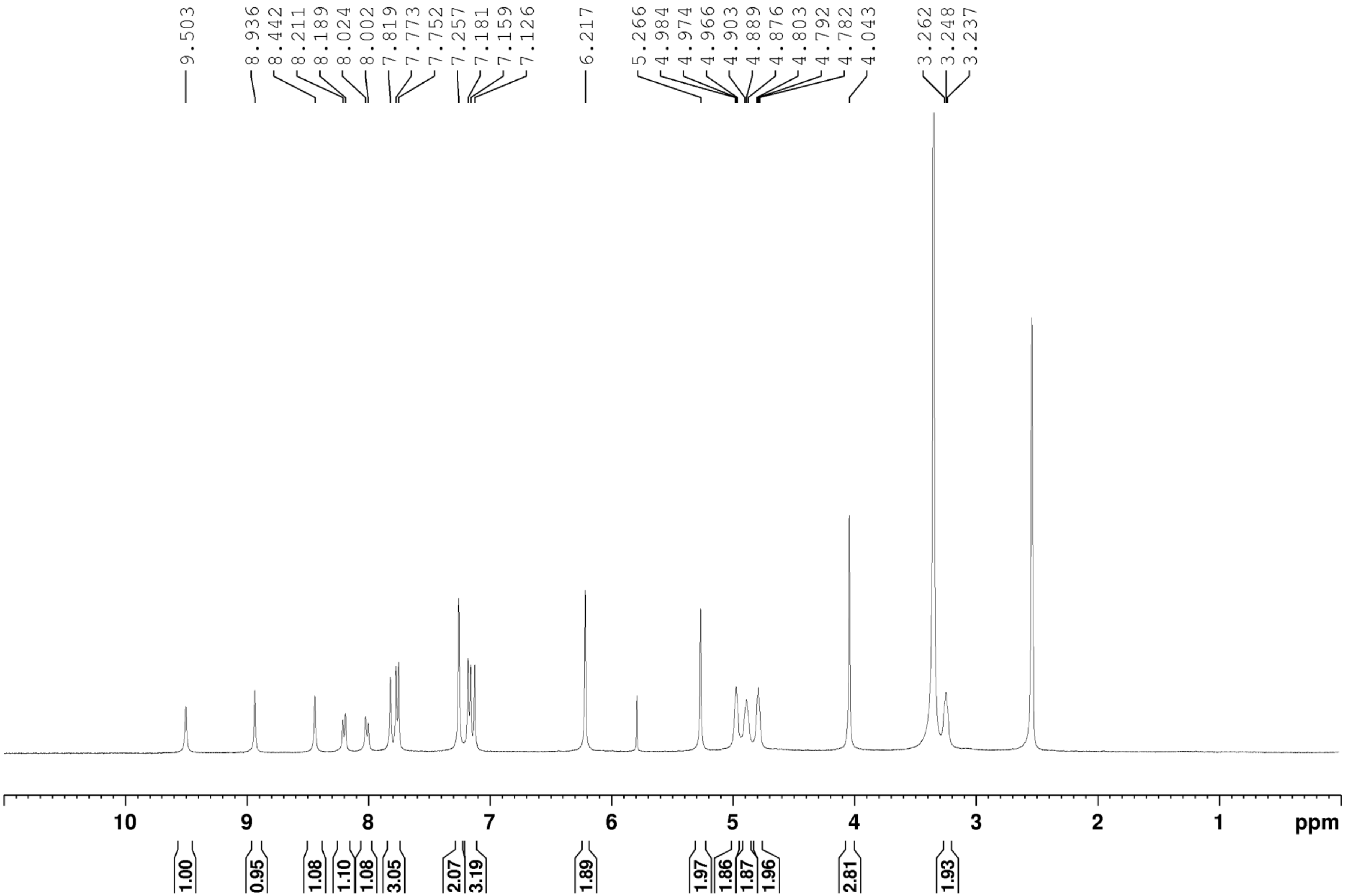
9.503
8.936
8.442
8.211
8.189
8.024
8.002
7.819
7.773
7.752
7.257
7.181
7.159
7.126
6.217
5.266
4.984
4.974
4.966
4.903
4.889
4.876
4.803
4.792
4.782
4.043
3.262
3.248
3.237
10
9
8
7
6
5
4
3
2
1
ppm
1.00
0.95
1.08
1.10
1.08
3.05
2.07
3.19
1.89
1.97
1.86
1.87
1.96
2.81
1.93

$^{13}$C-NMR **27**

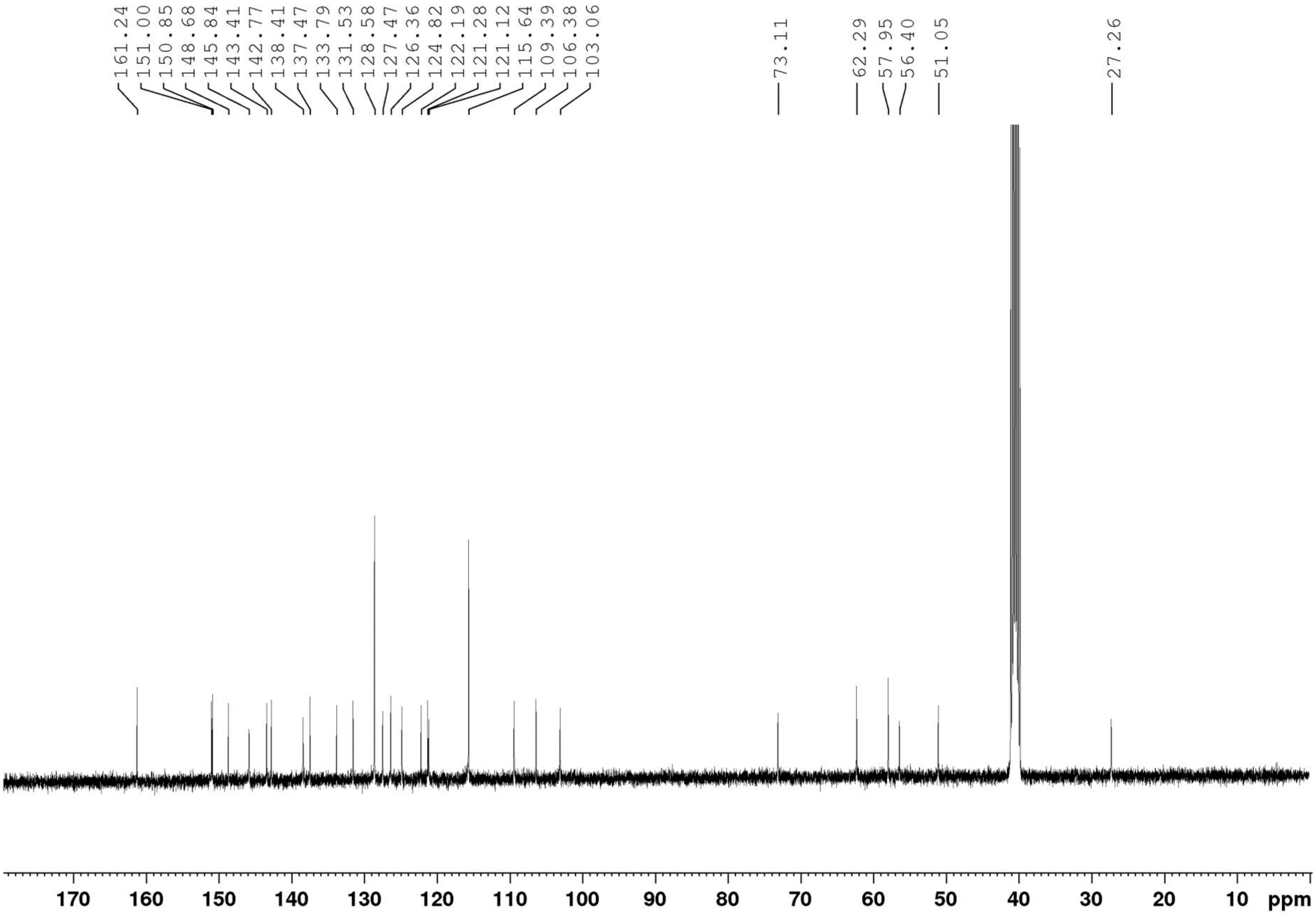

161.24
151.00
150.85
148.68
145.84
143.41
142.77
138.41
137.47
133.79
131.53
128.58
127.47
126.36
124.82
122.19
121.28
121.12
115.64
109.39
106.38
103.06
73.11
62.29
57.95
56.40
51.05
27.26
170
160
150
140
130
120
110
100
90
80
70
60
50
40
30
20
10
ppm

$^{1}$H-NMR **28**

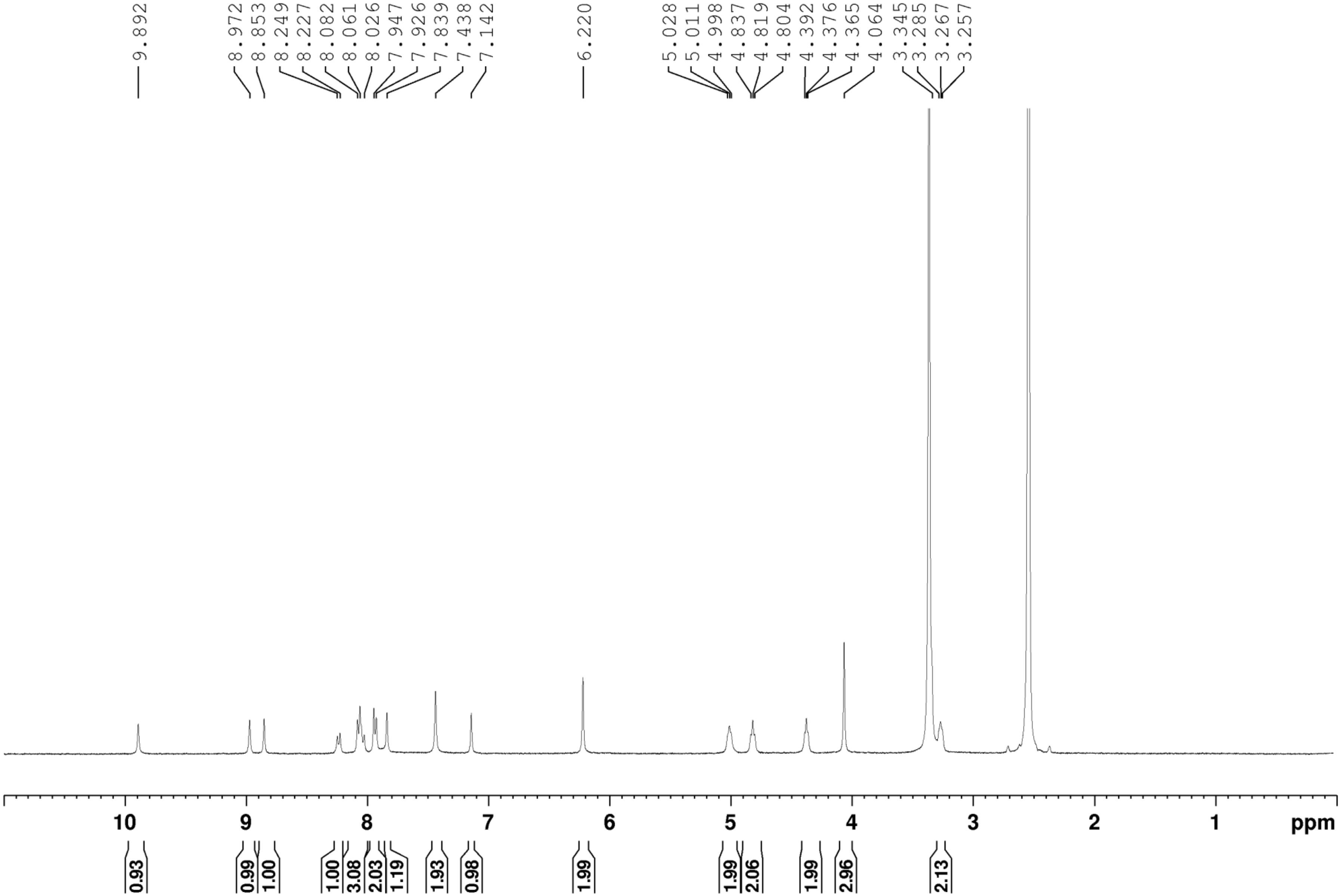

9.892
8.972
8.853
8.249
8.227
8.082
8.061
8.026
7.947
7.926
7.839
7.438
7.142
6.220
5.028
5.011
4.998
4.837
4.819
4.804
4.392
4.376
4.365
4.064
3.345
3.285
3.267
3.257
10
9
8
7
6
5
4
3
2
1
ppm
0.93
0.99
1.00
1.00
3.08
2.03
1.19
1.93
0.98
1.99
1.99
2.06
1.99
2.96
2.13

$^{13}$C-NMR **28**

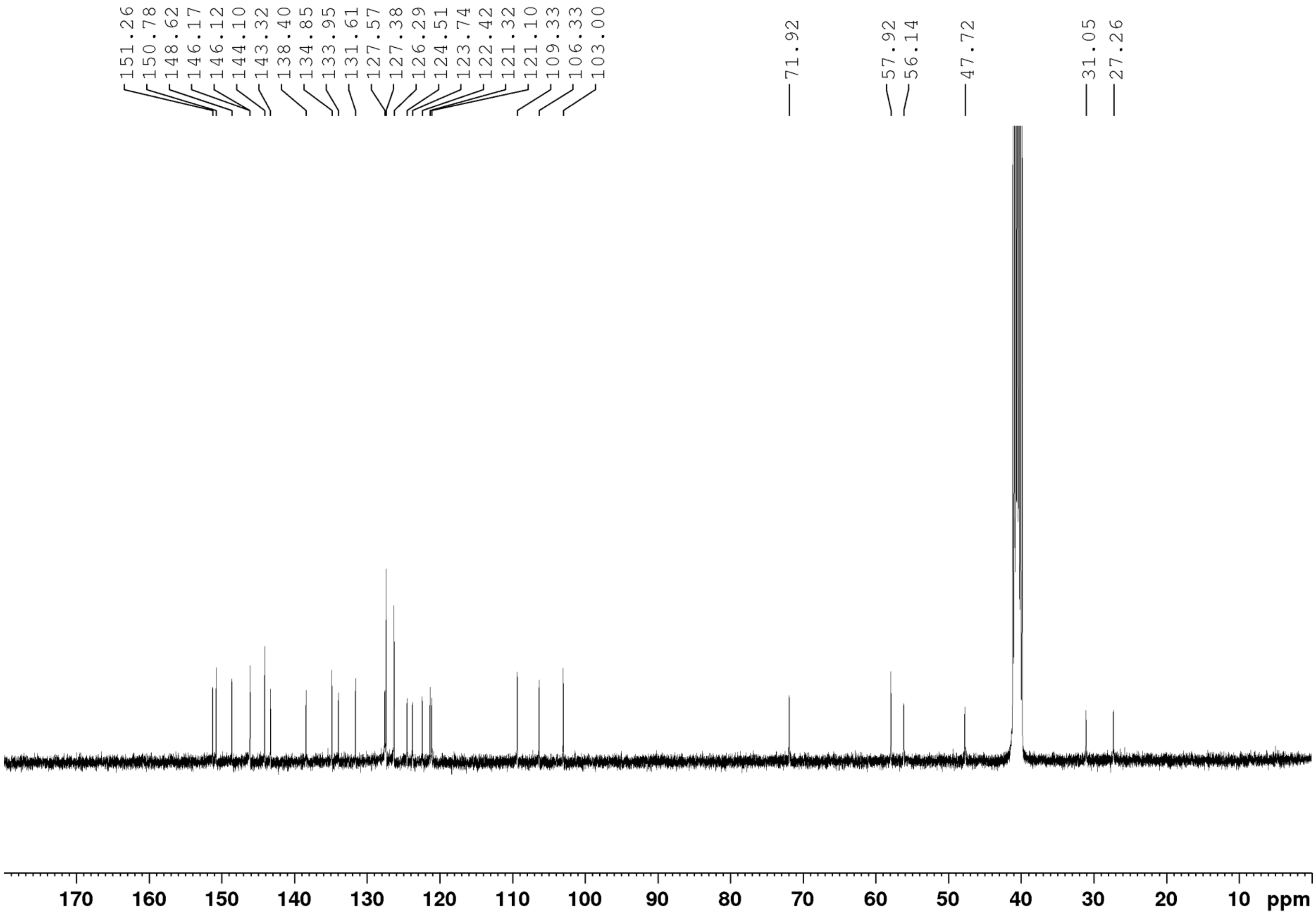

151.26
150.78
148.62
146.17
146.12
144.10
143.32
138.40
134.85
133.95
131.61
127.57
127.38
126.29
124.51
123.74
122.42
121.32
121.10
109.33
106.33
103.00
71.92
57.92
56.14
47.72
31.05
27.26
170
160
150
140
130
120
110
100
90
80
70
60
50
40
30
20
10
ppm

<sup></sup>

$^{1}$H-NMR **29**

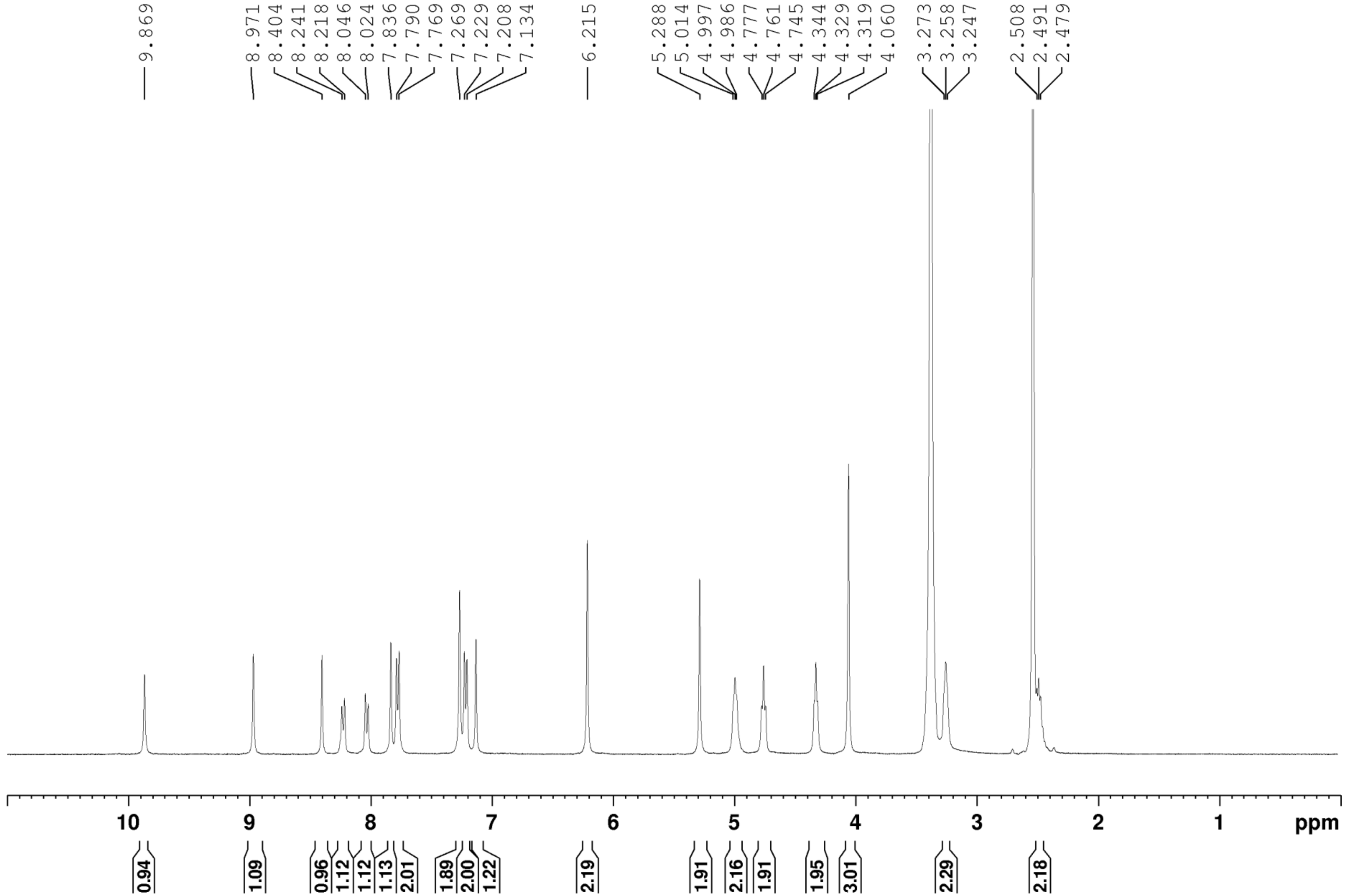

$^{13}$C-NMR **29**

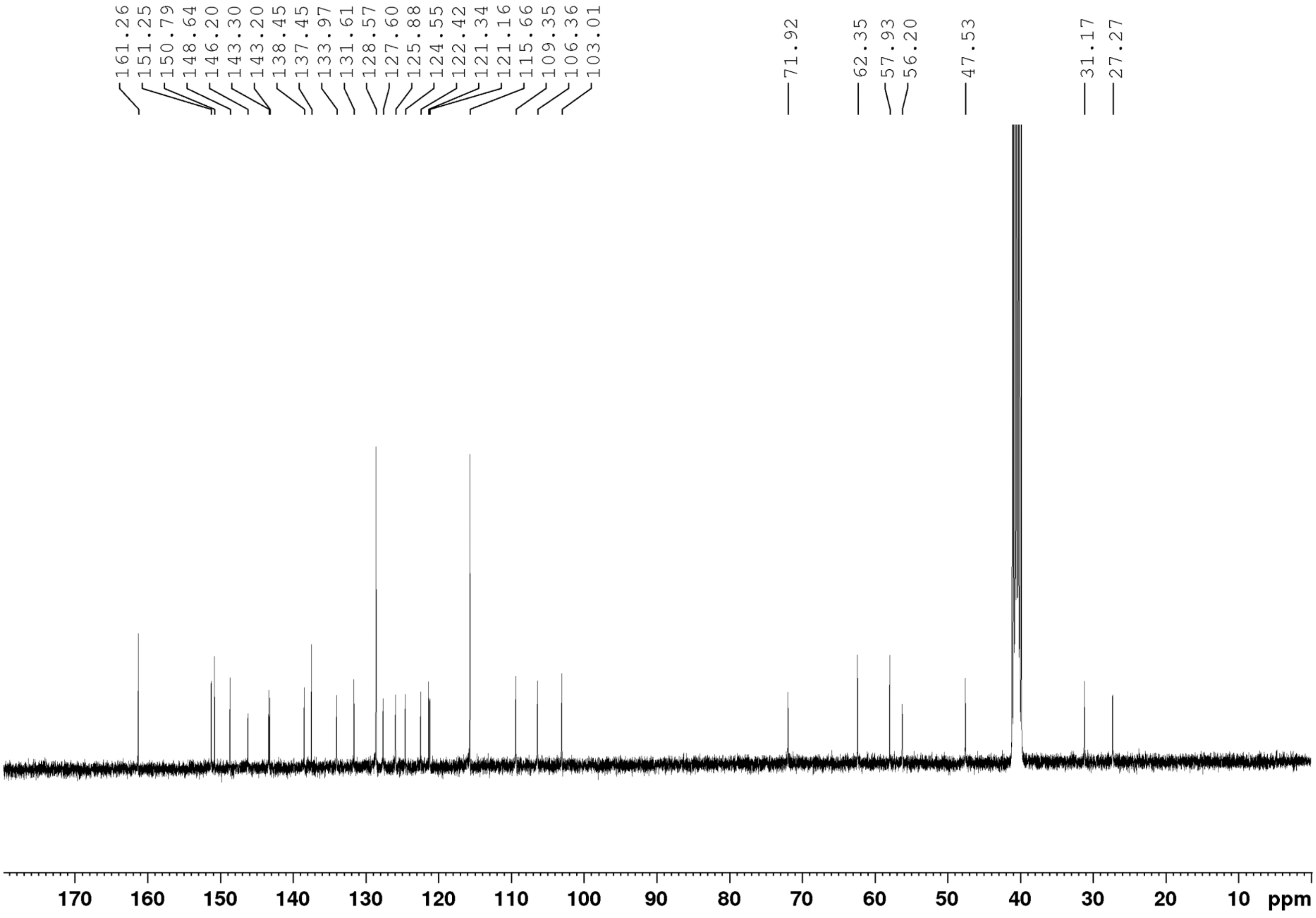
161.26
151.25
150.79
148.64
146.20
143.30
143.20
138.45
137.45
133.97
131.61
128.57
127.60
125.88
124.55
122.42
121.34
121.16
115.66
109.35
106.36
103.01
71.92
62.35
57.93
56.20
47.53
31.17
27.27
170 160 150 140 130 120 110 100 90 80 70 60 50 40 30 20 10 ppm

$^{1}$H-NMR **30**

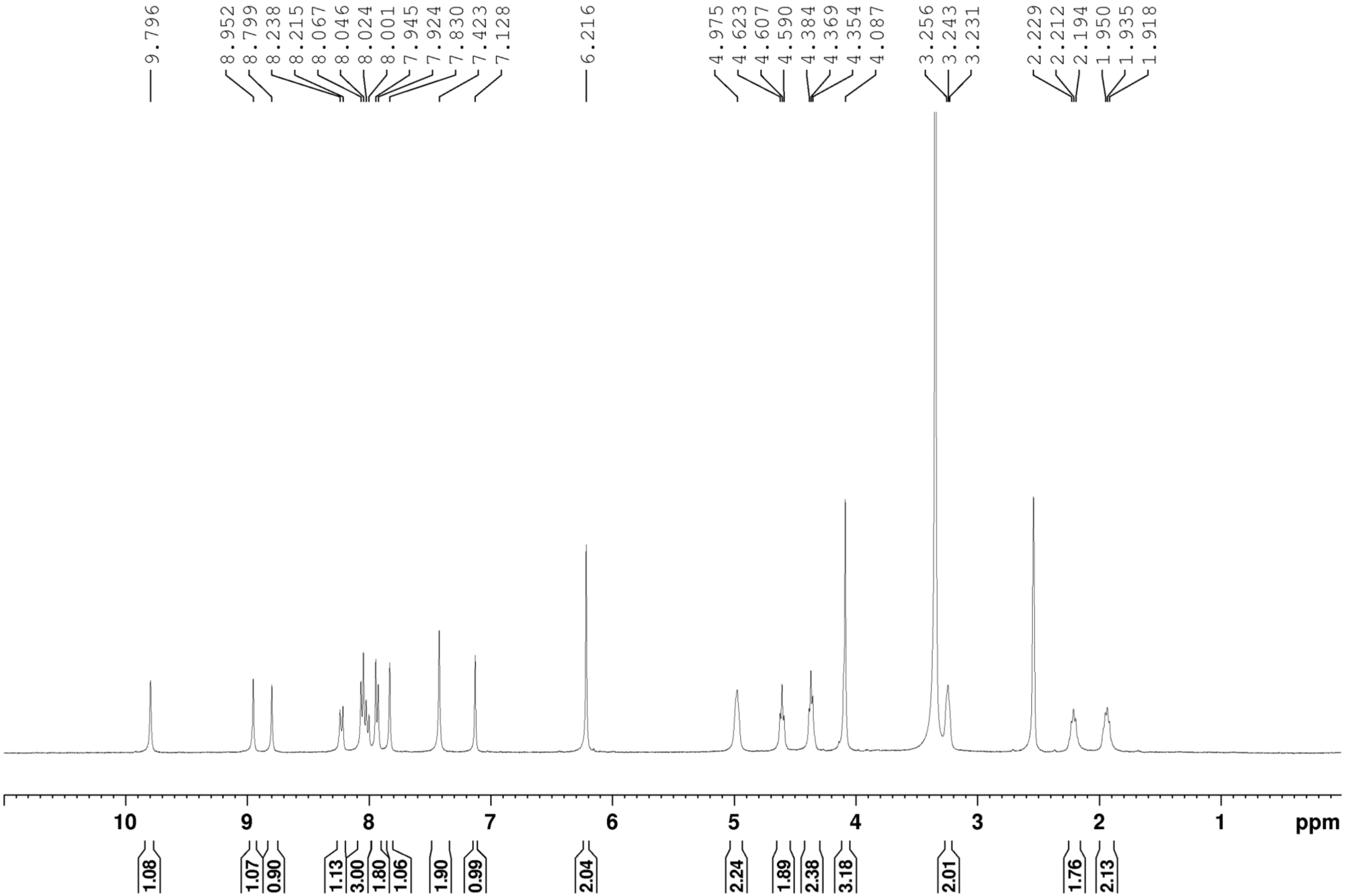

9.796
8.952
8.799
8.238
8.215
8.067
8.046
8.024
8.001
7.945
7.924
7.830
7.423
7.128
6.216
4.975
4.623
4.607
4.590
4.384
4.369
4.354
4.087
3.256
3.243
3.231
2.229
2.212
2.194
1.950
1.935
1.918
10
9
8
7
6
5
4
3
2
1
ppm
1.08
1.07
0.90
1.13
3.00
1.80
1.06
1.90
0.99
2.04
2.24
1.89
2.38
3.18
2.01
1.76
2.13

$^{13}$C-NMR **30**

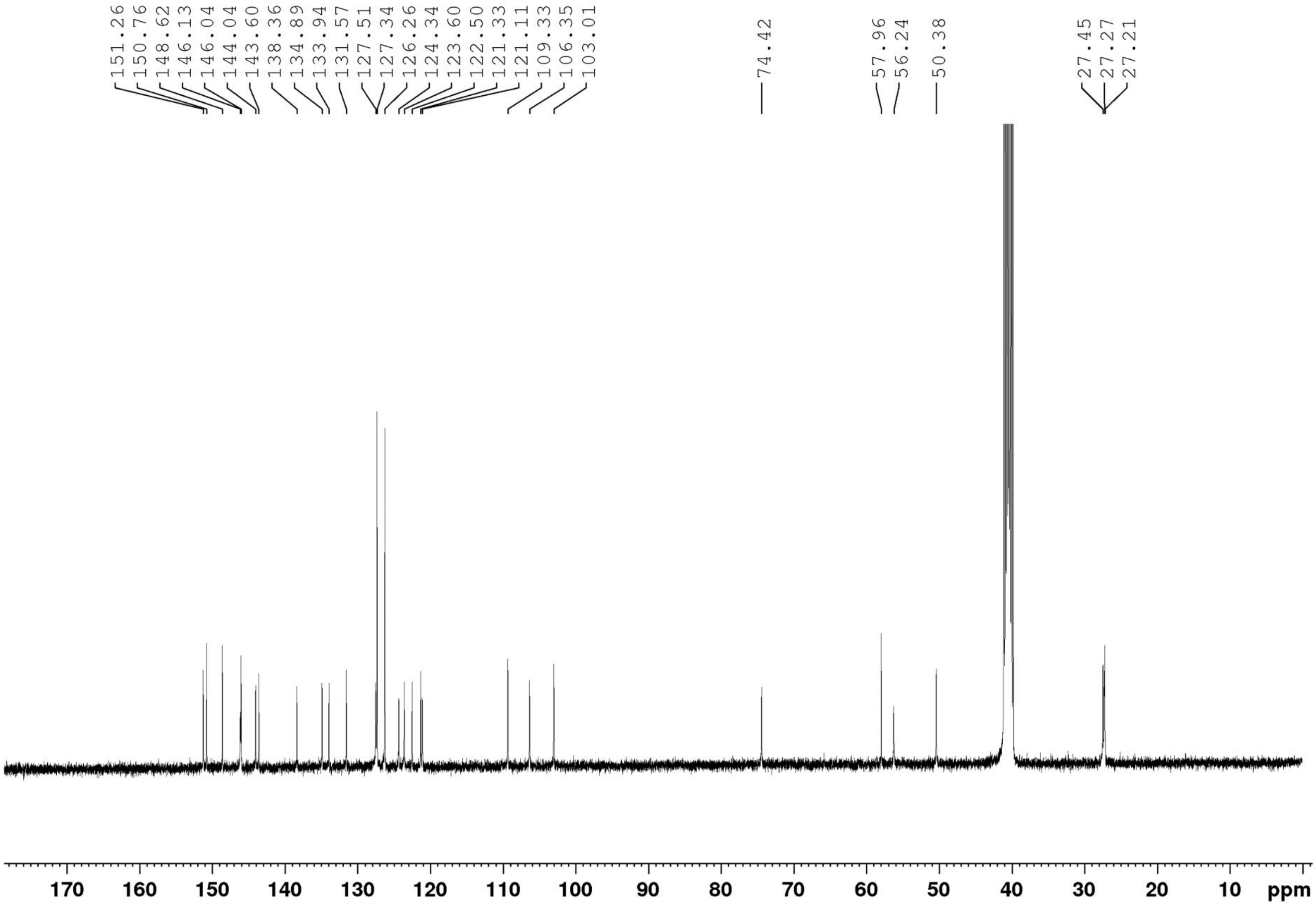

151.26
150.76
148.62
146.13
146.04
144.04
143.60
138.36
134.89
133.94
131.57
127.51
127.34
126.26
124.34
123.60
122.50
121.33
121.11
109.33
106.35
103.01
74.42
57.96
56.24
50.38
27.45
27.27
27.21
170
160
150
140
130
120
110
100
90
80
70
60
50
40
30
20
10
ppm

$^1$H-NMR **31**

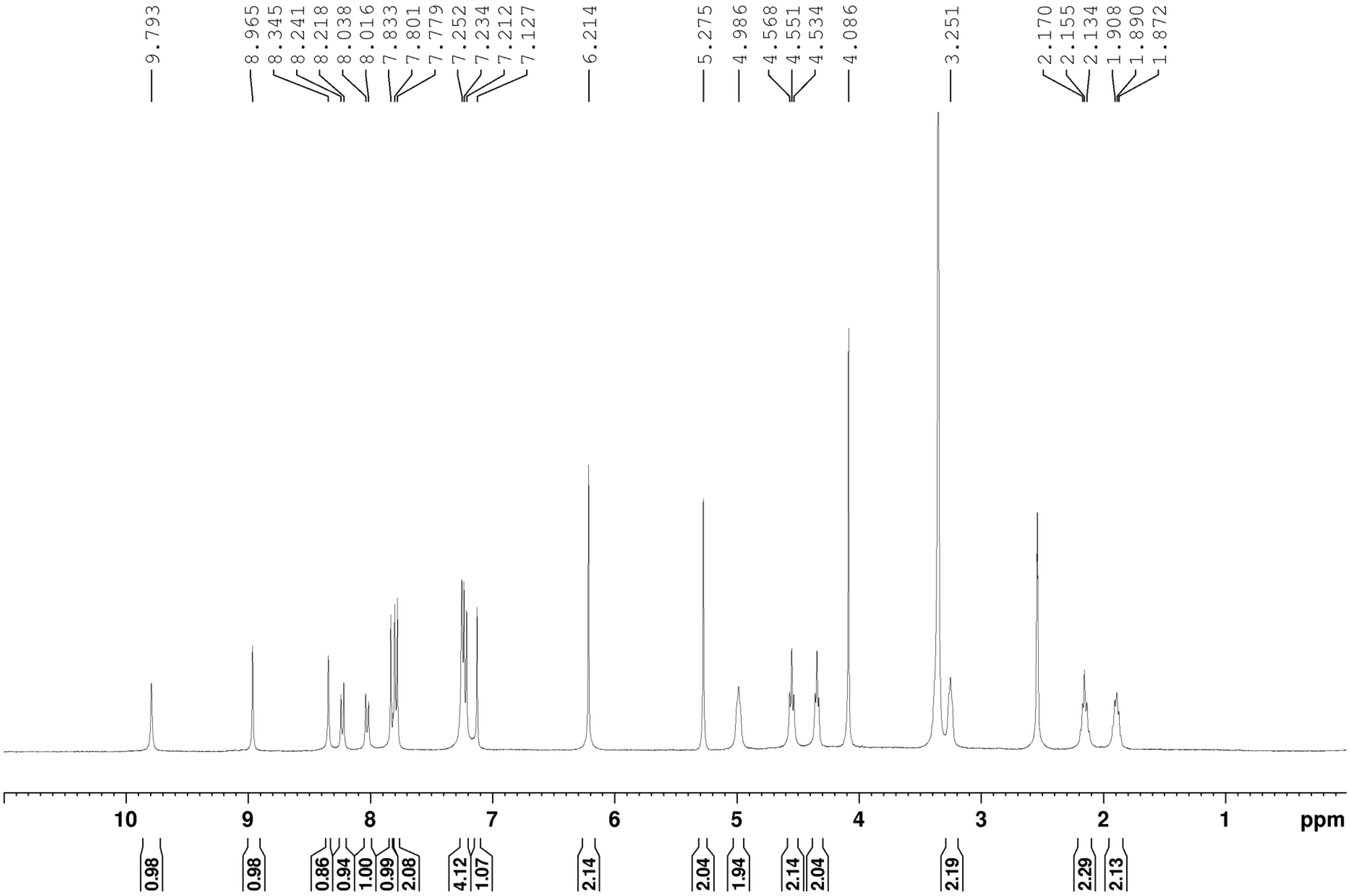

9.793
8.965
8.345
8.241
8.218
8.038
8.016
7.833
7.801
7.779
7.252
7.234
7.212
7.127
6.214
5.275
4.986
4.568
4.551
4.534
4.086
3.251
2.170
2.155
2.134
1.908
1.890
1.872
10
9
8
7
6
5
4
3
2
1
ppm
0.98
0.98
0.86
0.94
1.00
0.99
2.08
4.12
1.07
2.14
2.04
1.94
2.14
2.04
2.19
2.29
2.13

$^{13}$C-NMR **31**

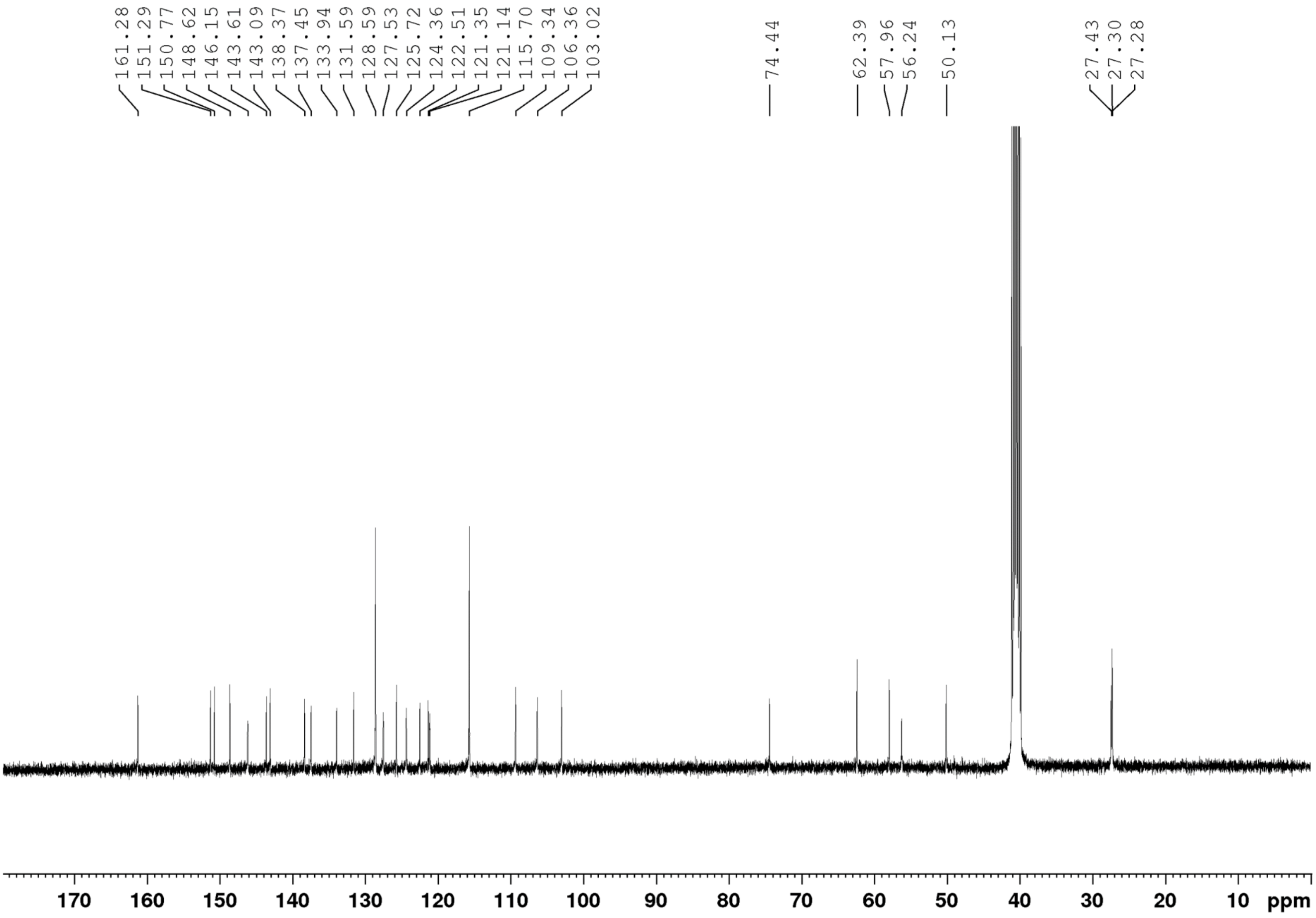
161.28
151.29
150.77
148.62
146.15
143.61
143.09
138.37
137.45
133.94
131.59
128.59
127.53
125.72
124.36
122.51
121.35
121.14
115.70
109.34
106.36
103.02
74.44
62.39
57.96
56.24
50.13
27.43
27.30
27.28
170
160
150
140
130
120
110
100
90
80
70
60
50
40
30
20
10
ppm

$^{1}$H-NMR **32**

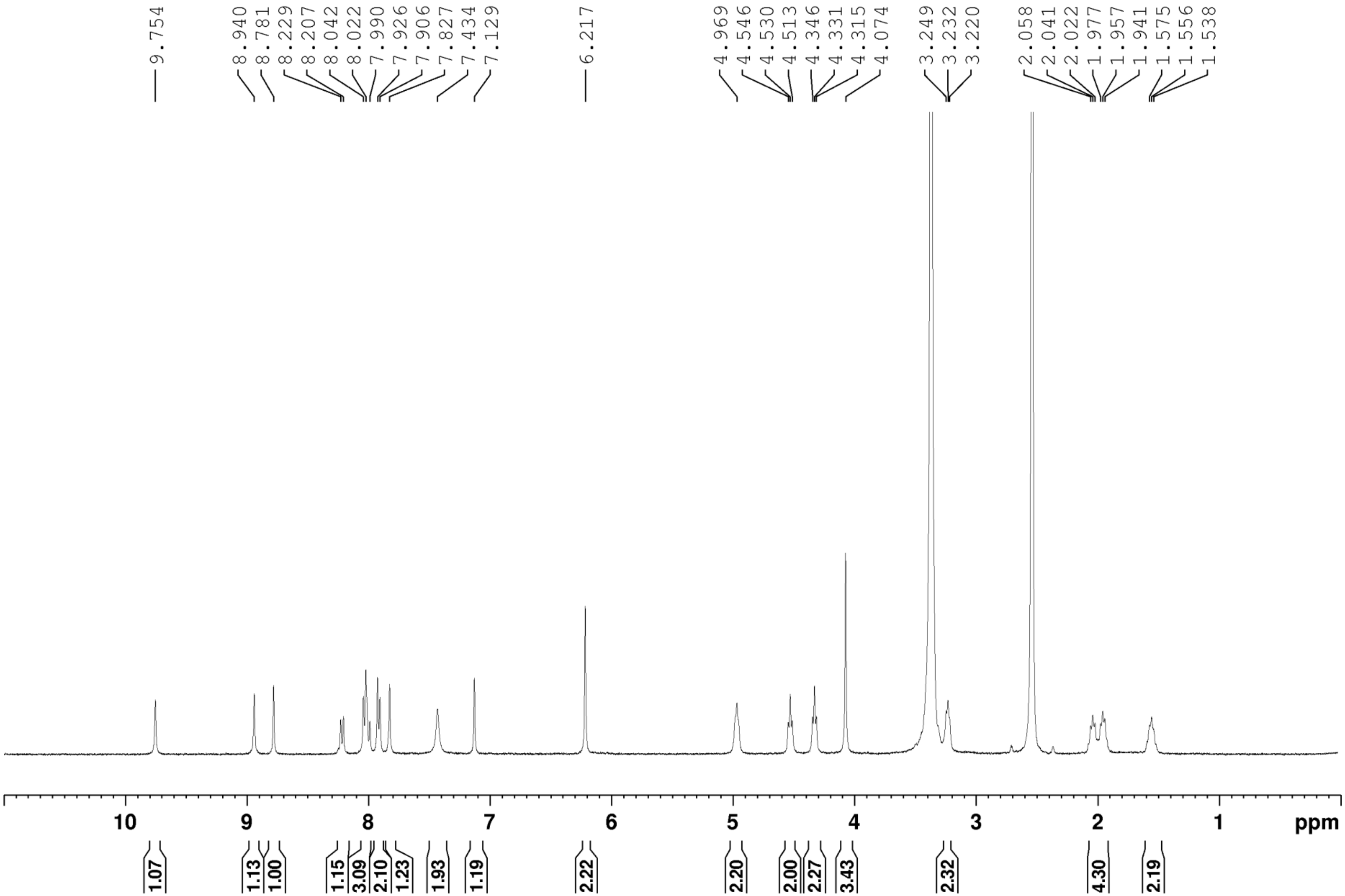
9.754
8.940
8.781
8.229
8.207
8.042
8.022
7.990
7.926
7.906
7.827
7.434
7.129
6.217
4.969
4.546
4.530
4.513
4.346
4.331
4.315
4.074
3.249
3.232
3.220
2.058
2.041
2.022
1.977
1.957
1.941
1.575
1.556
1.538
10
9
8
7
6
5
4
3
2
1
ppm
1.07
1.13
1.00
1.15
3.09
2.10
1.23
1.93
1.19
2.22
2.20
2.00
2.27
3.43
2.32
4.30
2.19

$^{13}$C-NMR **32**

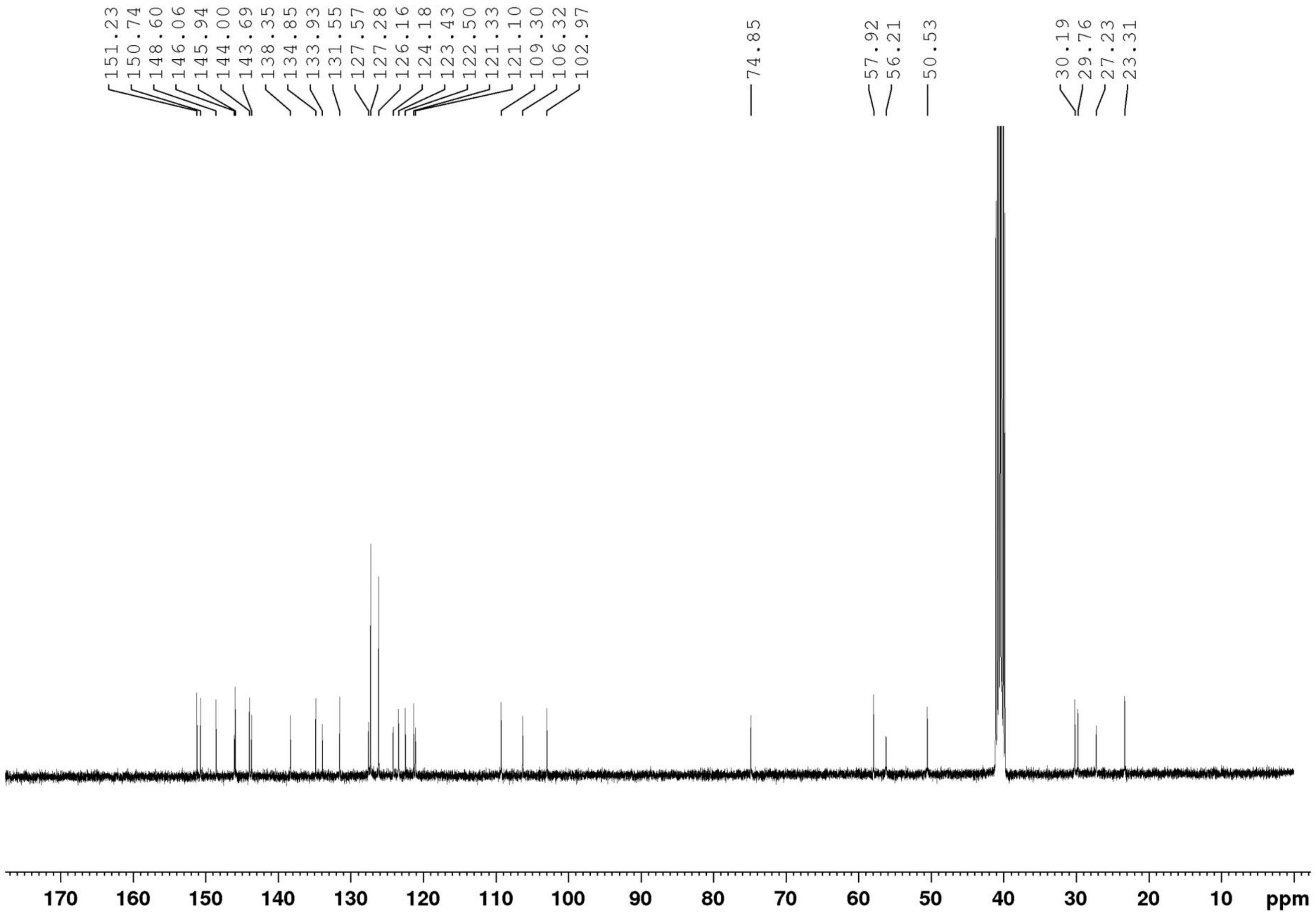
151.23
150.74
148.60
146.06
145.94
144.00
143.69
138.35
134.85
133.93
131.55
127.57
127.28
126.16
124.18
123.43
122.50
121.33
121.10
109.30
106.32
102.97
74.85
57.92
56.21
50.53
30.19
29.76
27.23
23.31
170 160 150 140 130 120 110 100 90 80 70 60 50 40 30 20 10 ppm

$^{1}$H-NMR **33**

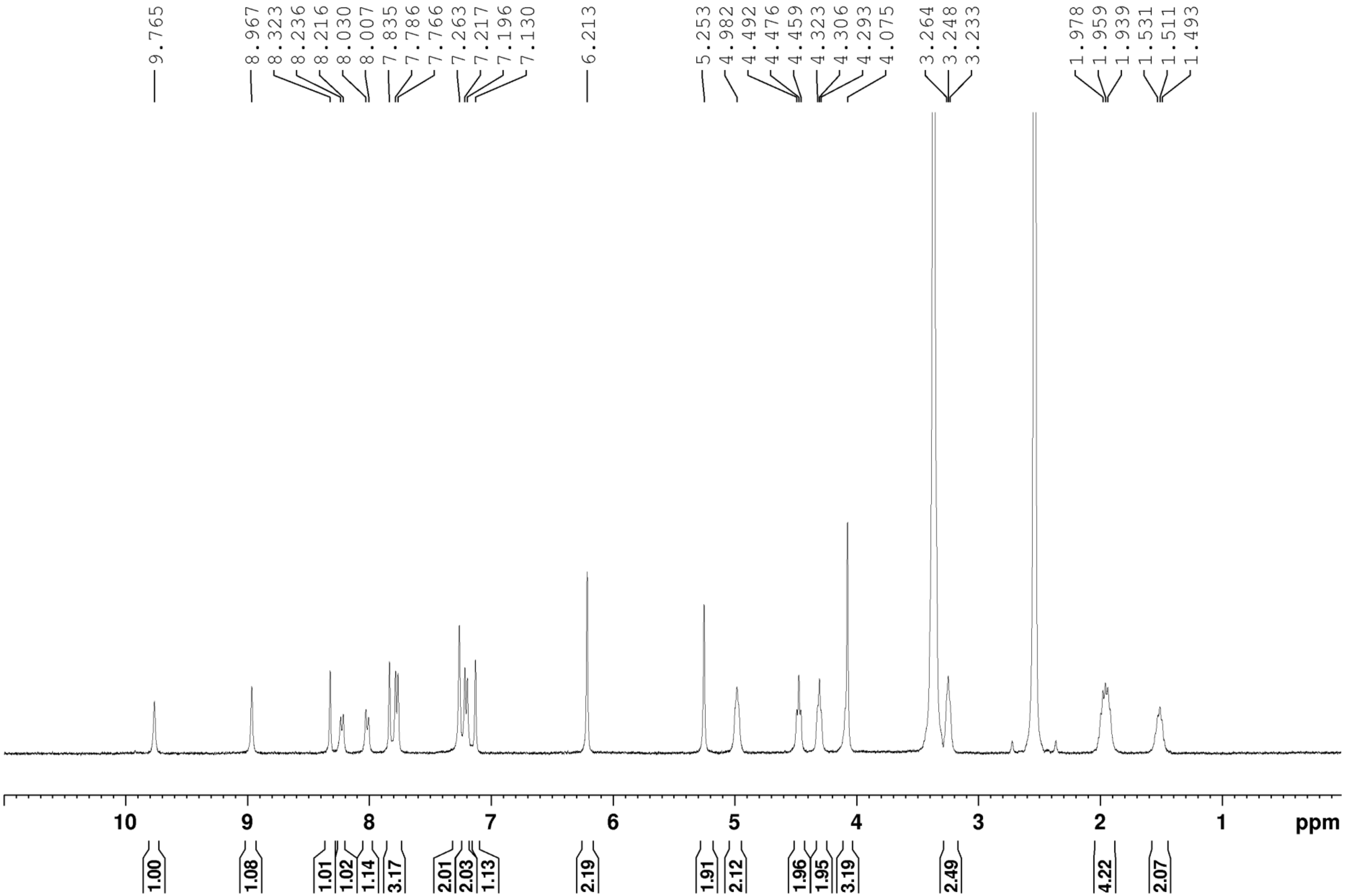
9.765
8.967
8.323
8.236
8.216
8.030
8.007
7.835
7.786
7.766
7.263
7.217
7.196
7.130
6.213
5.253
4.982
4.492
4.476
4.459
4.323
4.306
4.293
4.075
3.264
3.248
3.233
1.978
1.959
1.939
1.531
1.511
1.493
10
9
8
7
6
5
4
3
2
1
ppm
1.00
1.08
1.01
1.02
1.14
3.17
2.01
2.03
1.13
2.19
1.91
2.12
1.96
1.95
3.19
2.49
4.22
2.07

$^{13}$C-NMR **33**

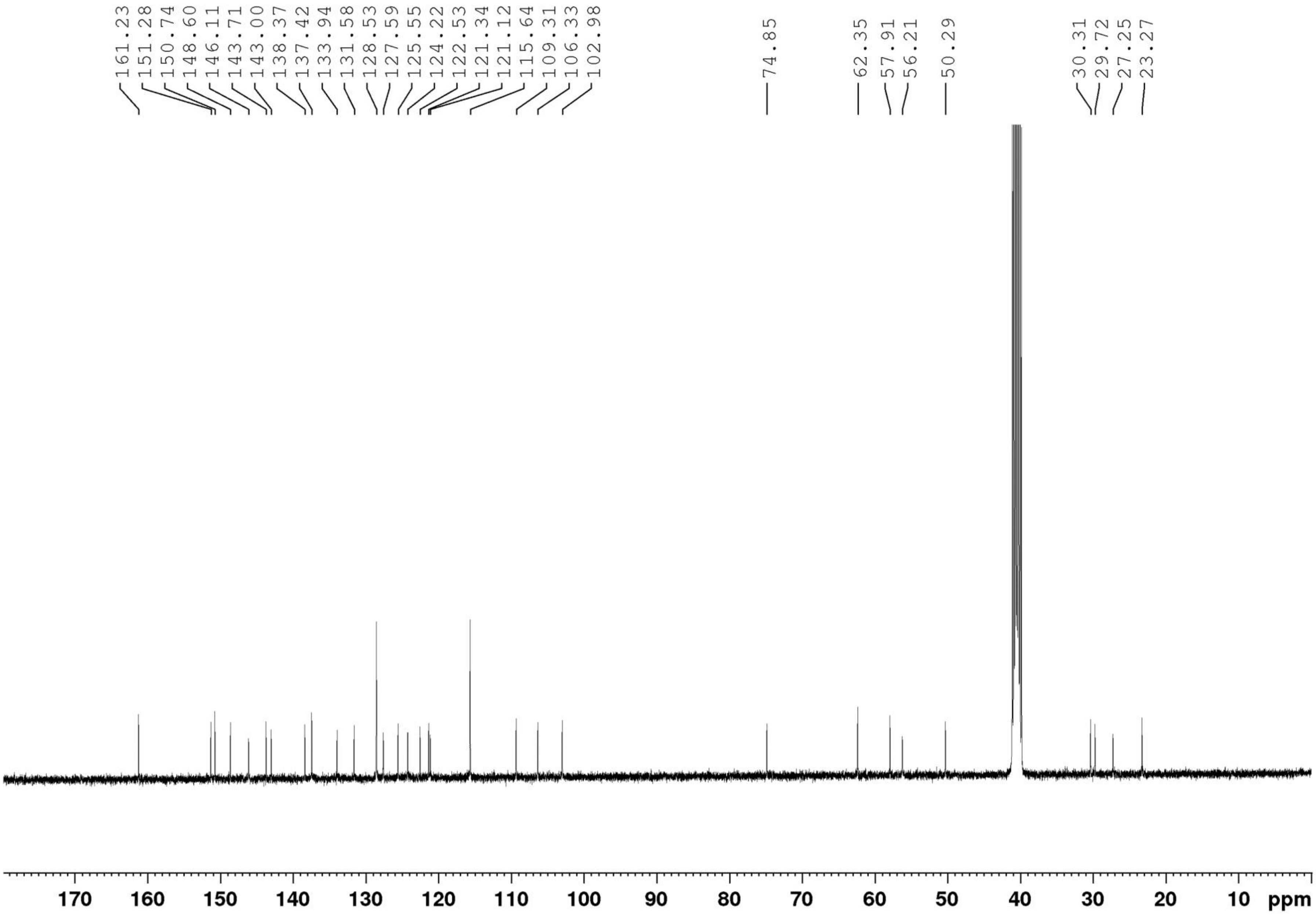
161.23
151.28
150.74
148.60
146.11
143.71
143.00
138.37
137.42
133.94
131.58
128.53
127.59
125.55
124.22
122.53
121.34
121.12
115.64
109.31
106.33
102.98
74.85
62.35
57.91
56.21
50.29
30.31
29.72
27.25
23.27
170
160
150
140
130
120
110
100
90
80
70
60
50
40
30
20
10
ppm

$^{1}$H-NMR **34**

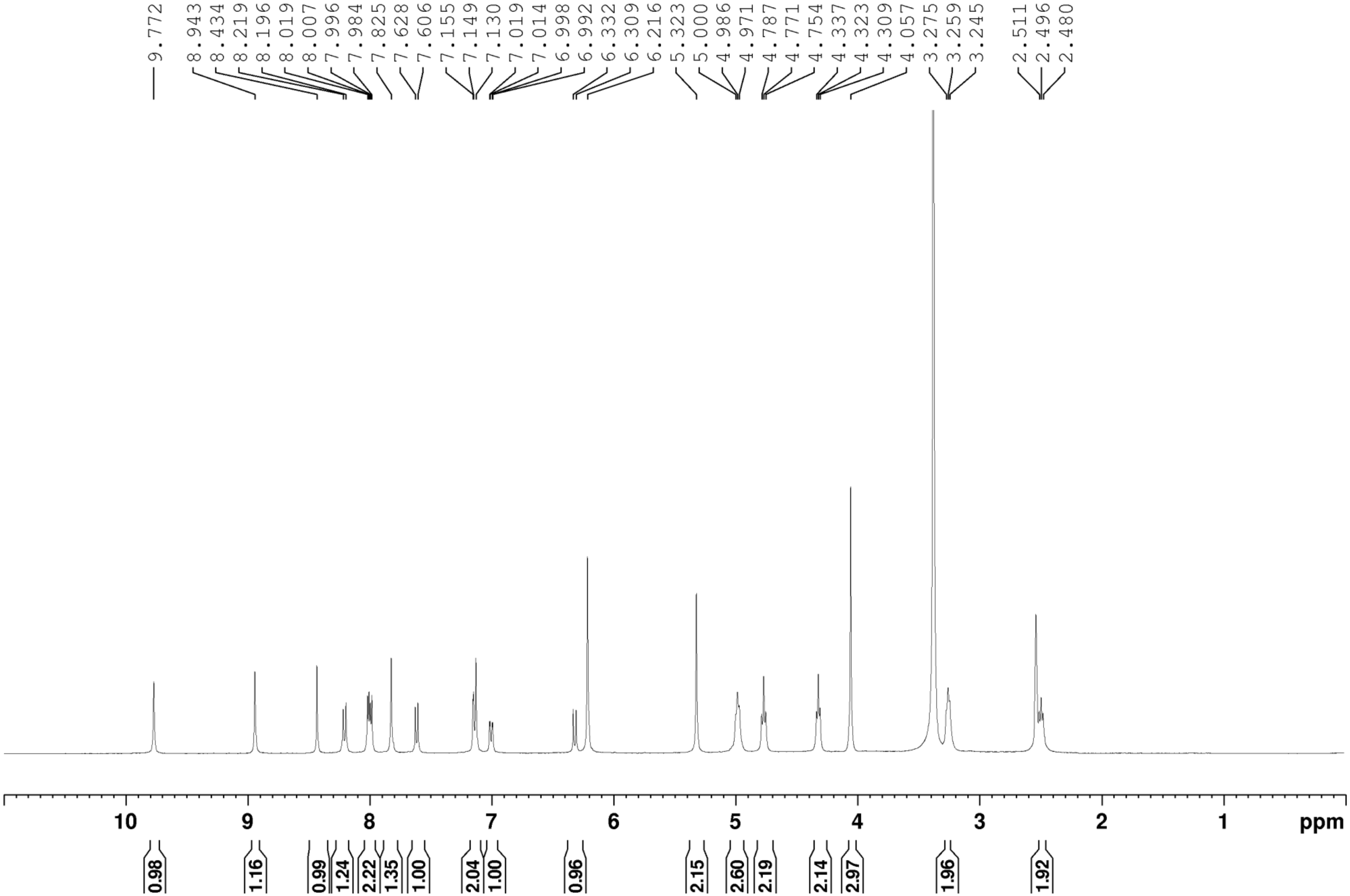
9.772
8.943
8.434
8.219
8.196
8.019
8.007
7.996
7.984
7.825
7.628
7.606
7.155
7.149
7.130
7.019
7.014
6.998
6.992
6.332
6.309
6.216
5.323
5.000
4.986
4.971
4.787
4.771
4.754
4.337
4.323
4.309
4.057
3.275
3.259
3.245
2.511
2.496
2.480
10
9
8
7
6
5
4
3
2
1
ppm
0.98
1.16
0.99
1.24
2.22
1.35
1.00
2.04
1.00
0.96
2.15
2.60
2.19
2.14
2.97
1.96
1.92

$^{13}$C-NMR **34**

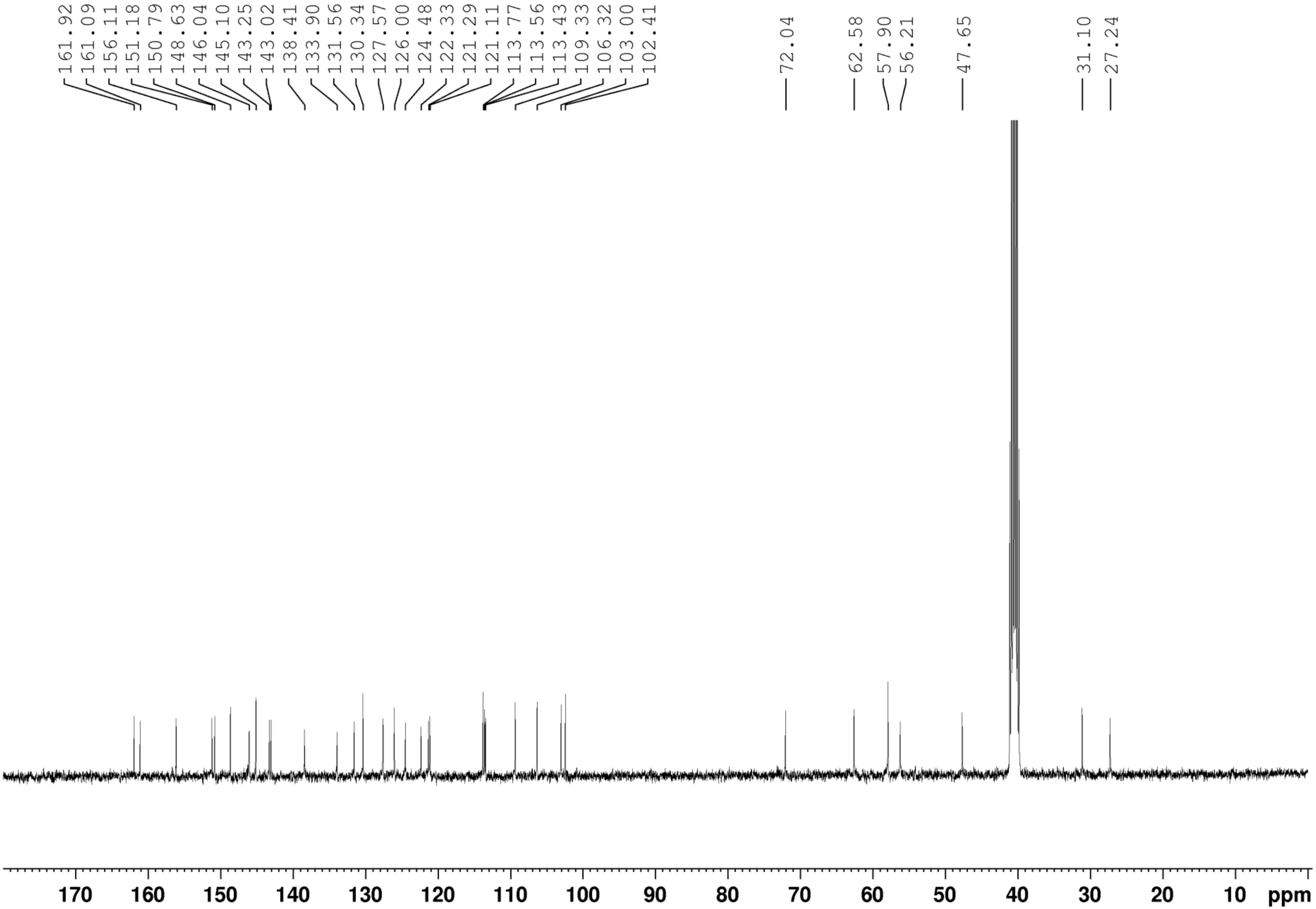
161.92
161.09
156.11
151.18
150.79
148.63
146.04
145.10
143.25
143.02
138.41
133.90
131.56
130.34
127.57
126.00
124.48
122.33
121.29
121.11
113.77
113.56
113.43
109.33
106.32
103.00
102.41
72.04
62.58
57.90
56.21
47.65
31.10
27.24
170
160
150
140
130
120
110
100
90
80
70
60
50
40
30
20
10
ppm

$^{1}$H-NMR **35**

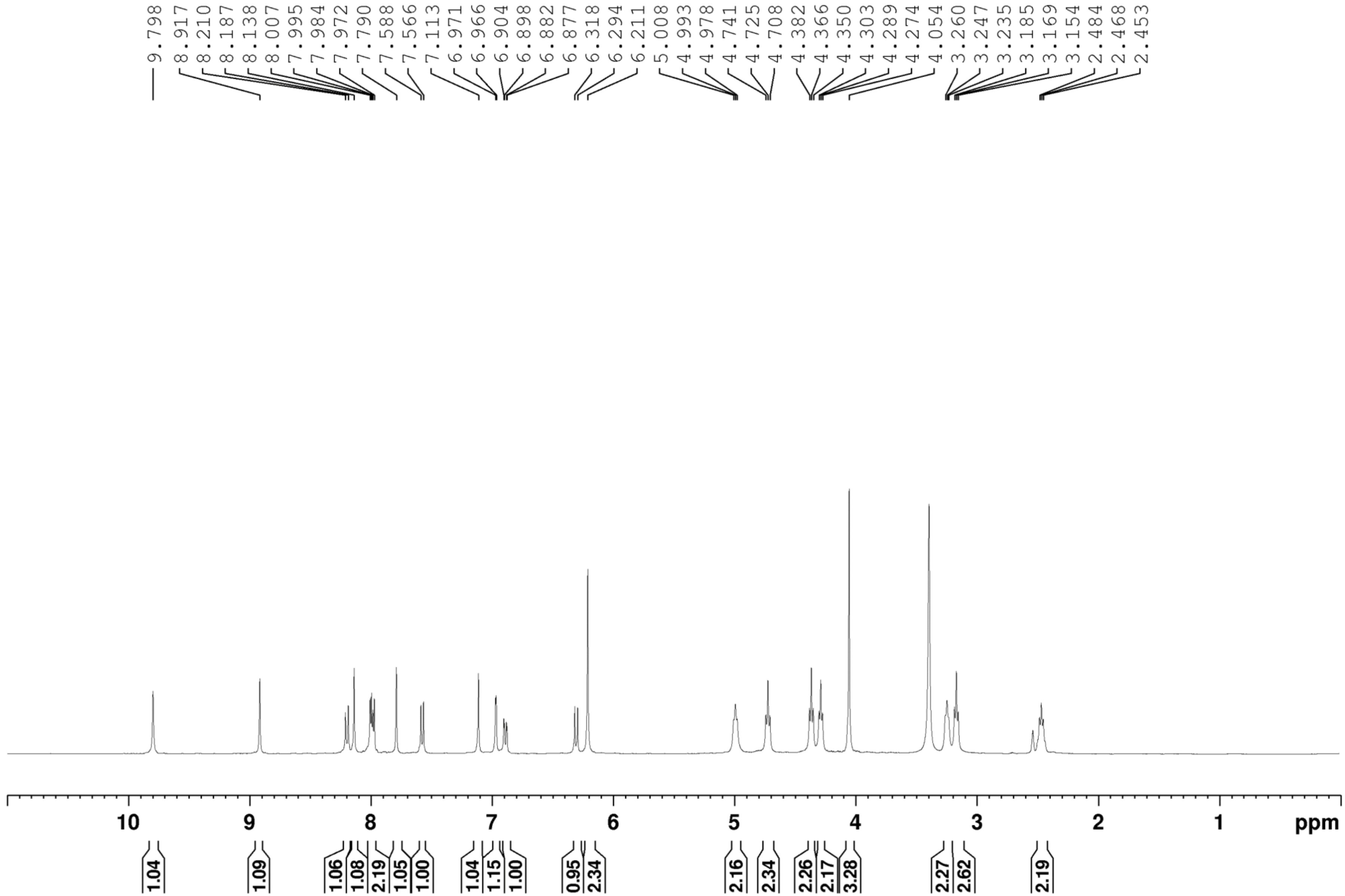

$^{13}C$-NMR **35**

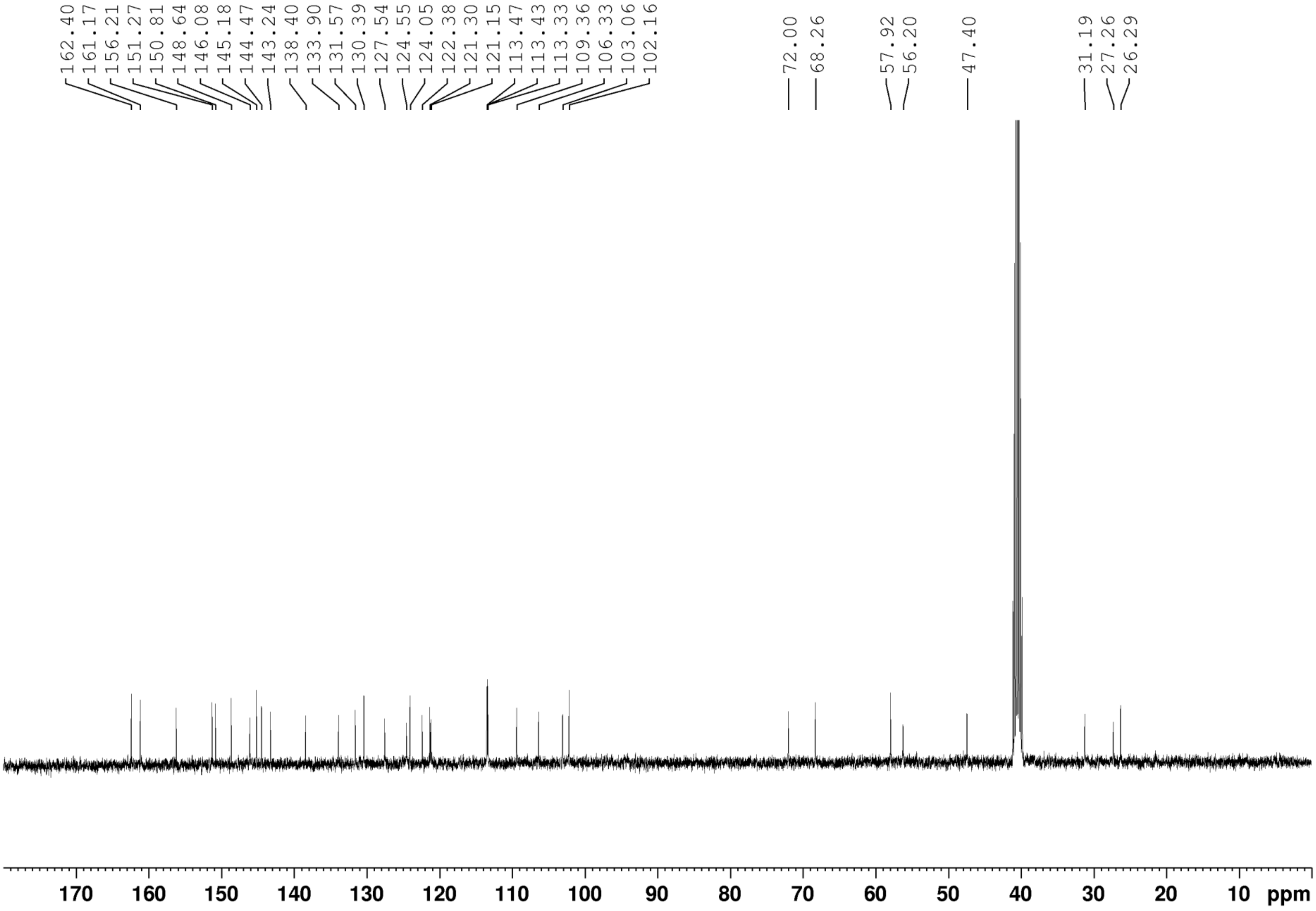
162.40
161.17
156.21
151.27
150.81
148.64
146.08
145.18
144.47
143.24
138.40
133.90
131.57
130.39
127.54
124.55
124.05
122.38
121.30
121.15
113.47
113.43
113.33
109.36
106.33
103.06
102.16
72.00
68.26
57.92
56.20
47.40
31.19
27.26
26.29
170
160
150
140
130
120
110
100
90
80
70
60
50
40
30
20
10
ppm

$^1$H-NMR **36**

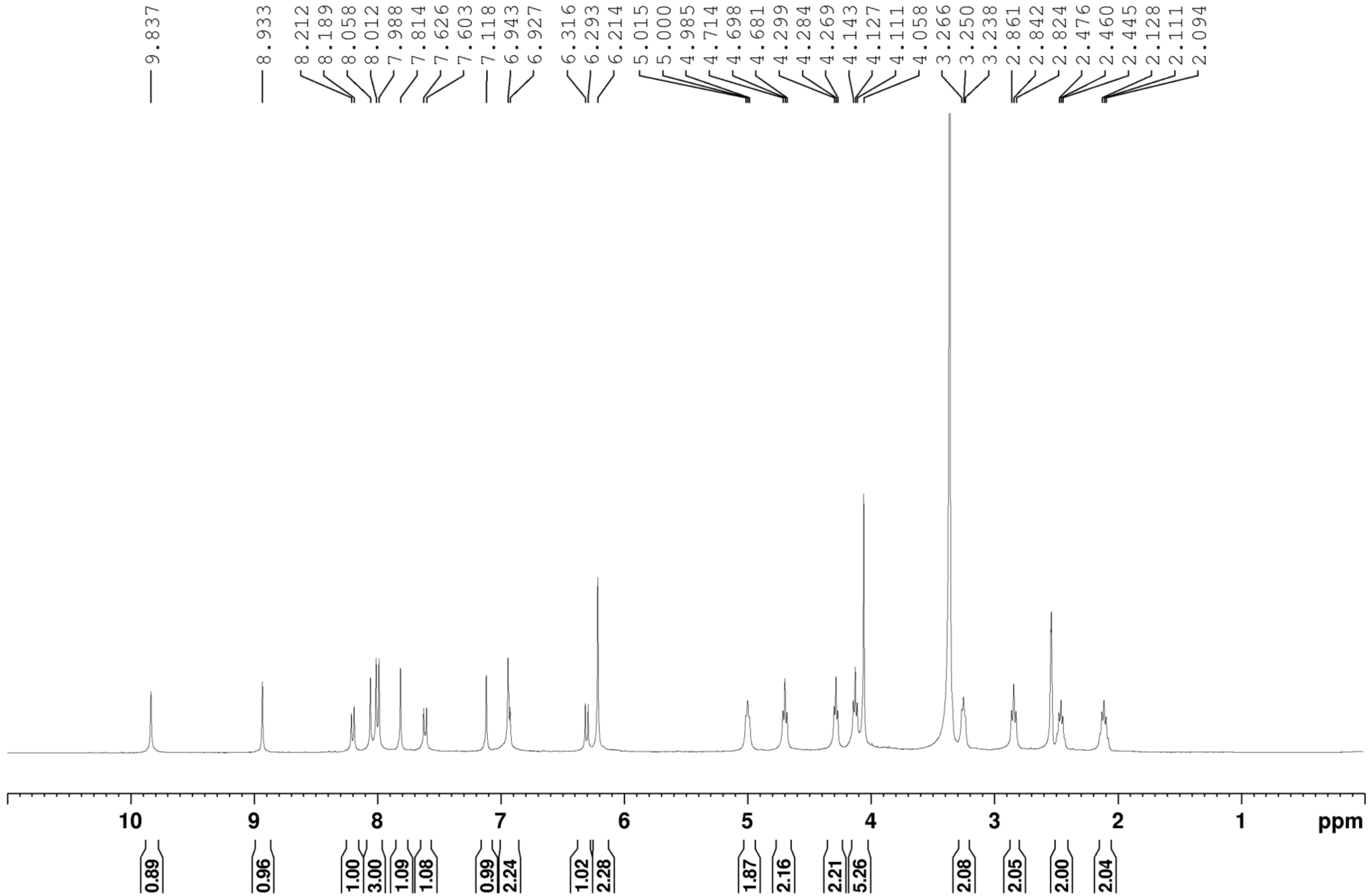
9.837
8.933
8.212
8.189
8.058
8.012
7.988
7.814
7.626
7.603
7.118
6.943
6.927
6.316
6.293
6.214
5.015
5.000
4.985
4.714
4.698
4.681
4.299
4.284
4.269
4.143
4.127
4.111
4.058
3.266
3.250
3.238
2.861
2.842
2.824
2.476
2.460
2.445
2.128
2.111
2.094
10
9
8
7
6
5
4
3
2
1
ppm
0.89
0.96
1.00
3.00
1.09
1.08
0.99
2.24
1.02
2.28
1.87
2.16
2.21
5.26
2.08
2.05
2.00
2.04

$^{13}$C-NMR **36**

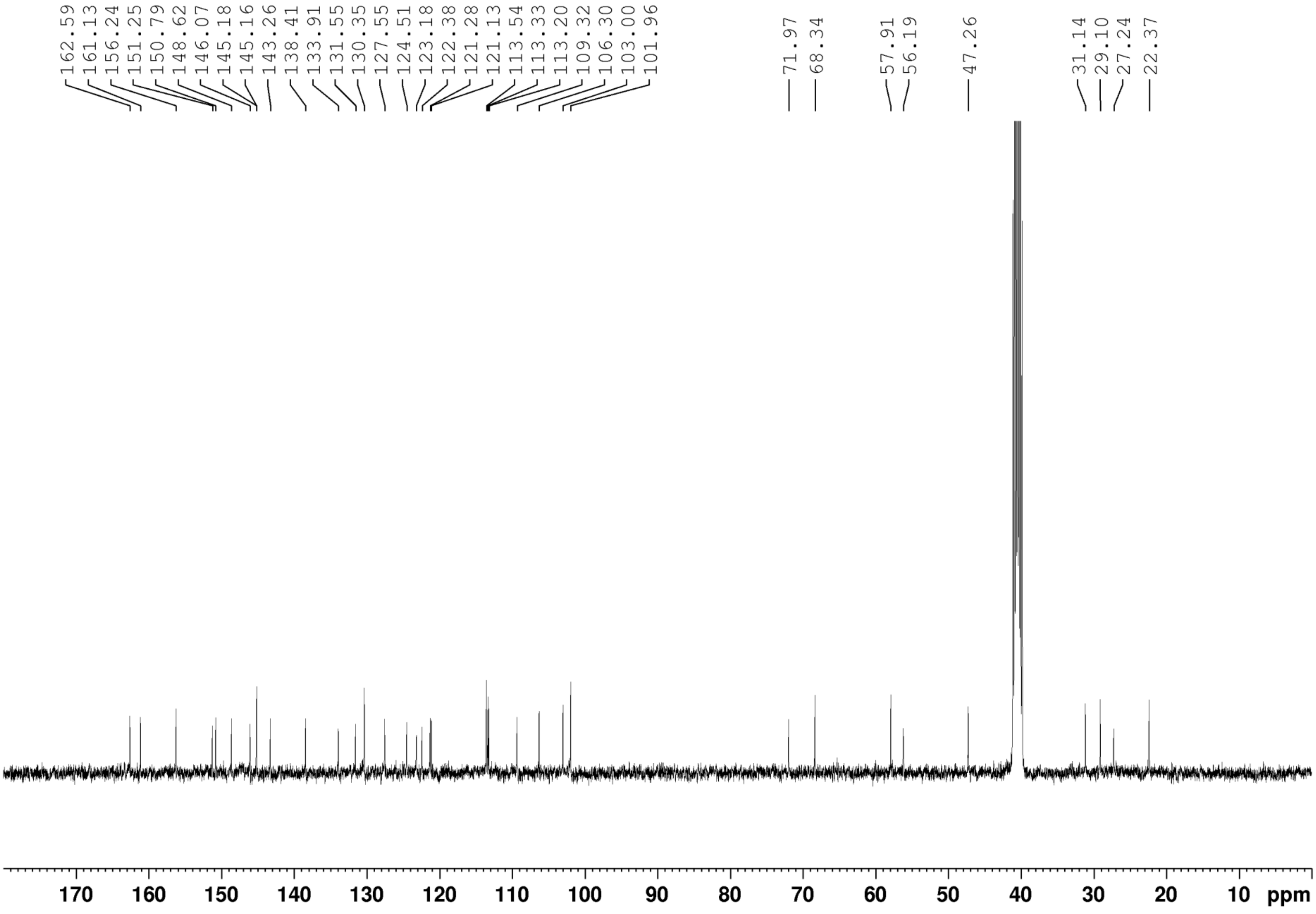
162.59
161.13
156.24
151.25
150.79
148.62
146.07
145.18
145.16
143.26
138.41
133.91
131.55
130.35
127.55
124.51
123.18
122.38
121.28
121.13
113.54
113.33
113.20
109.32
106.30
103.00
101.96
71.97
68.34
57.91
56.19
47.26
31.14
29.10
27.24
22.37
170
160
150
140
130
120
110
100
90
80
70
60
50
40
30
20
10
ppm

<sup></sup>$^{1}$H-NMR **37**

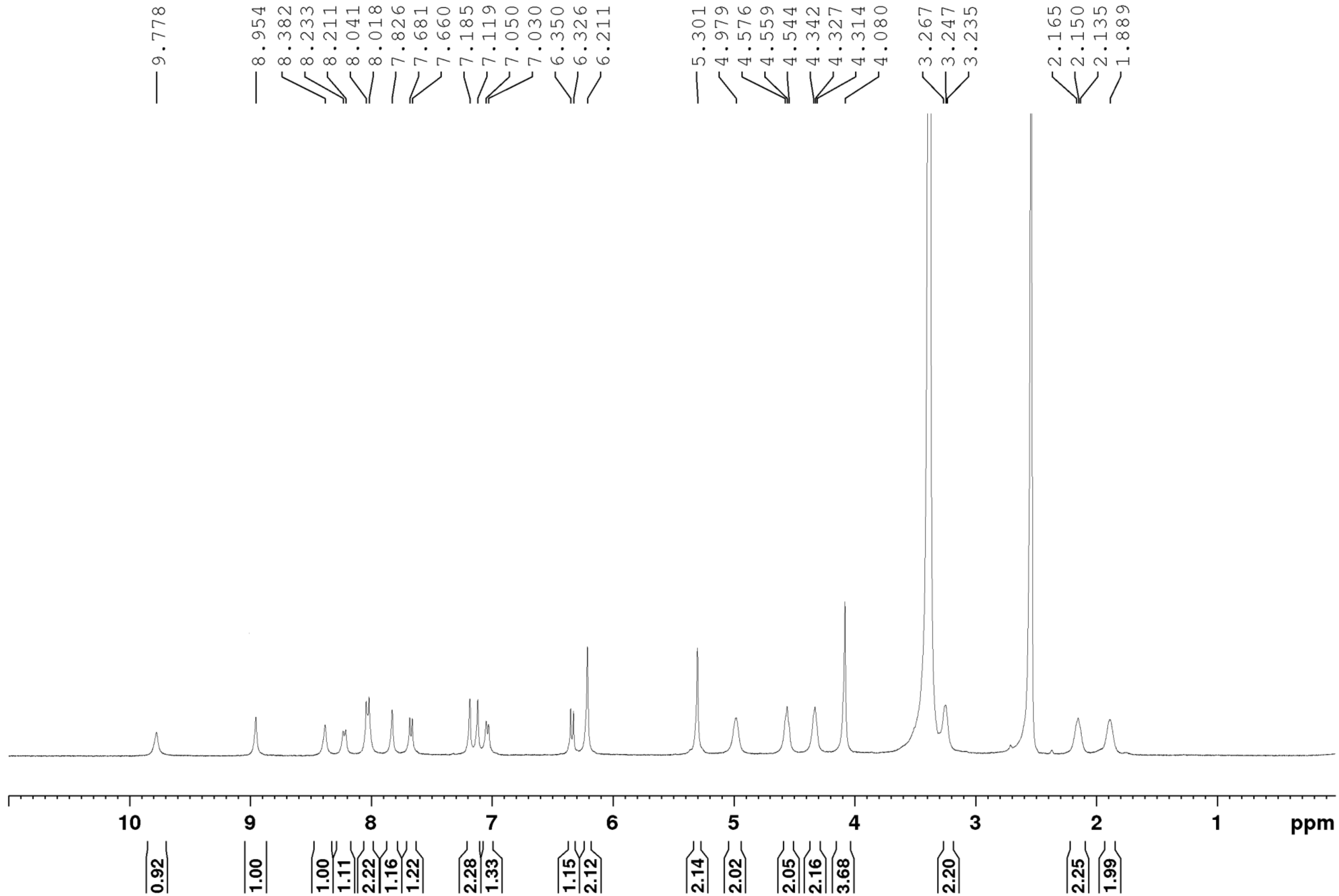

$^{13}C$-NMR **37**

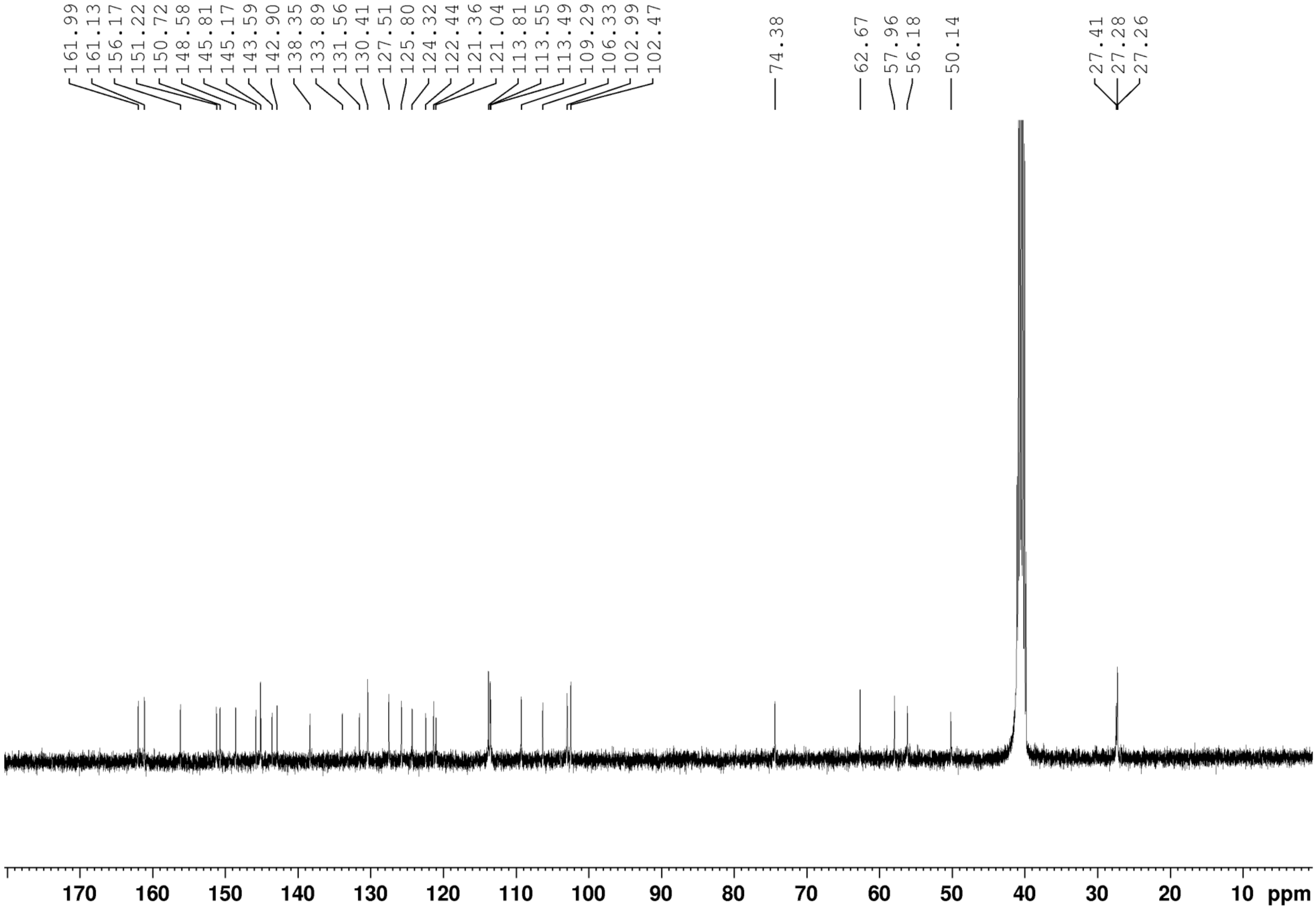
161.99
161.13
156.17
151.22
150.72
148.58
145.81
145.17
143.59
142.90
138.35
133.89
131.56
130.41
127.51
125.80
124.32
122.44
121.36
121.04
113.81
113.55
113.49
109.29
106.33
102.99
102.47
74.38
62.67
57.96
56.18
50.14
27.41
27.28
27.26
170
160
150
140
130
120
110
100
90
80
70
60
50
40
30
20
10
ppm

$^1$H-NMR **38**

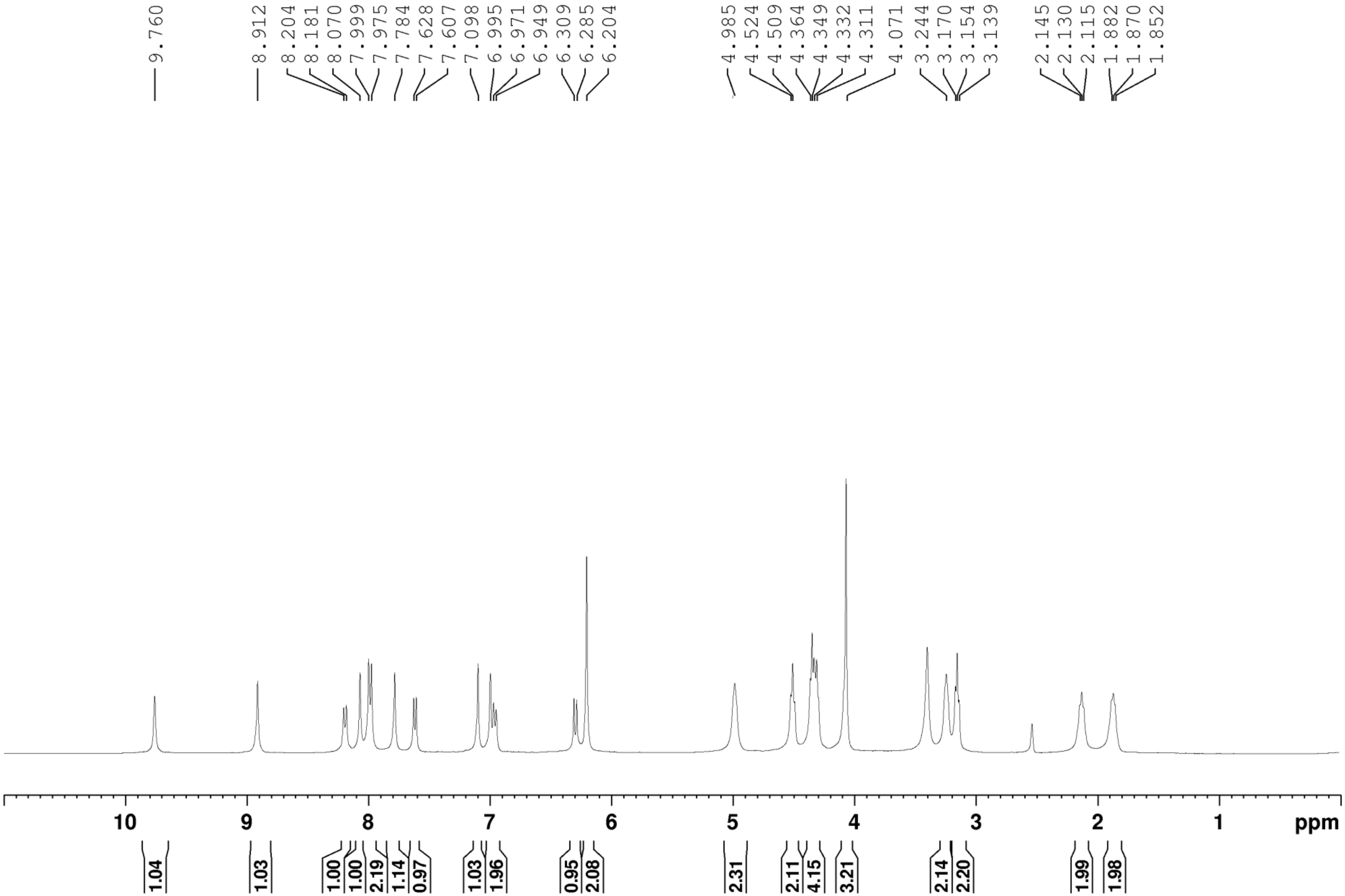
9.760
8.912
8.204
8.181
8.070
7.999
7.975
7.784
7.628
7.607
7.098
6.995
6.971
6.949
6.309
6.285
6.204
4.985
4.524
4.509
4.364
4.349
4.332
4.311
4.071
3.244
3.170
3.154
3.139
2.145
2.130
2.115
1.882
1.870
1.852
10
9
8
7
6
5
4
3
2
1
ppm
1.04
1.03
1.00
1.00
2.19
1.14
0.97
1.03
1.96
0.95
2.08
2.31
2.11
4.15
3.21
2.14
2.20
1.99
1.98

$^{13}$C-NMR **38**

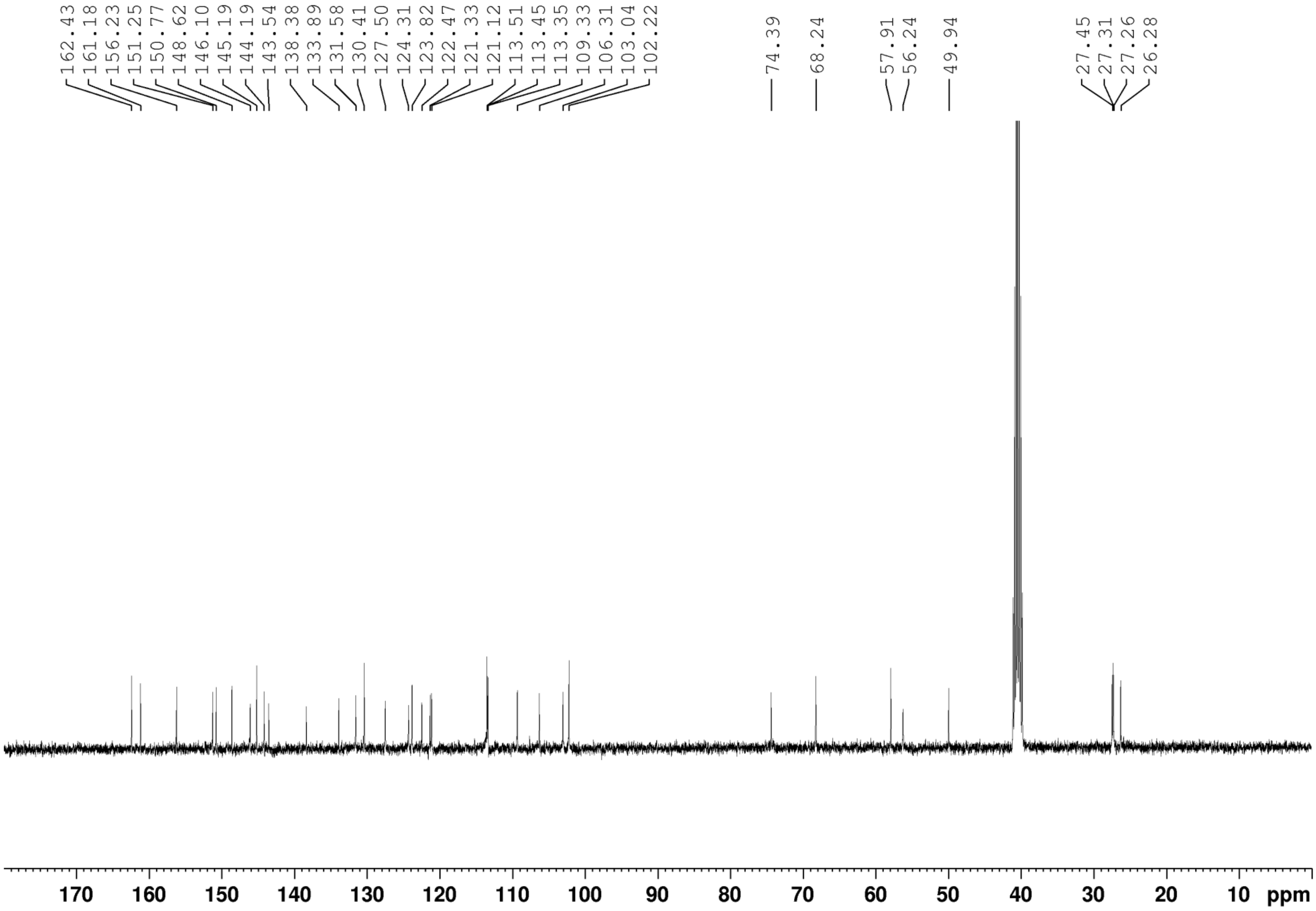
162.43
161.18
156.23
151.25
150.77
148.62
146.10
145.19
144.19
143.54
138.38
133.89
131.58
130.41
127.50
124.31
123.82
122.47
121.33
121.12
113.51
113.45
113.35
109.33
106.31
103.04
102.22
74.39
68.24
57.91
56.24
49.94
27.45
27.31
27.26
26.28
170
160
150
140
130
120
110
100
90
80
70
60
50
40
30
20
10
ppm

$^{1}$H-NMR **39**

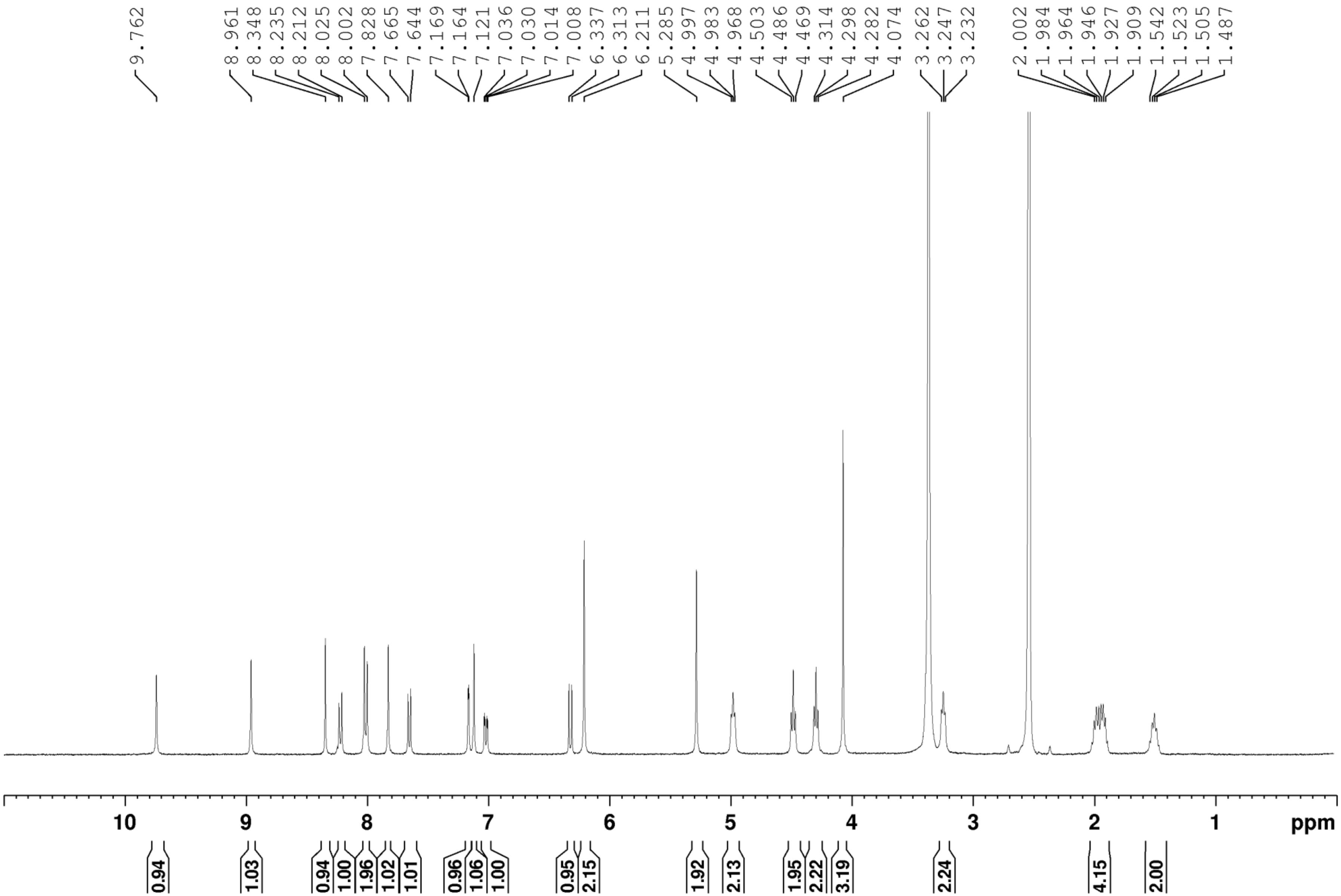
9.762
8.961
8.348
8.235
8.212
8.025
8.002
7.828
7.665
7.644
7.169
7.164
7.121
7.036
7.030
7.014
7.008
6.337
6.313
6.211
5.285
4.997
4.983
4.968
4.503
4.486
4.469
4.314
4.298
4.282
4.074
3.262
3.247
3.232
2.002
1.984
1.964
1.946
1.927
1.909
1.542
1.523
1.505
1.487
10 9 8 7 6 5 4 3 2 1 ppm
0.94
1.03
0.94
1.00
1.96
1.02
1.01
0.96
1.06
1.00
0.95
2.15
1.92
2.13
1.95
2.22
3.19
2.24
4.15
2.00

$^{13}$C-NMR **39**

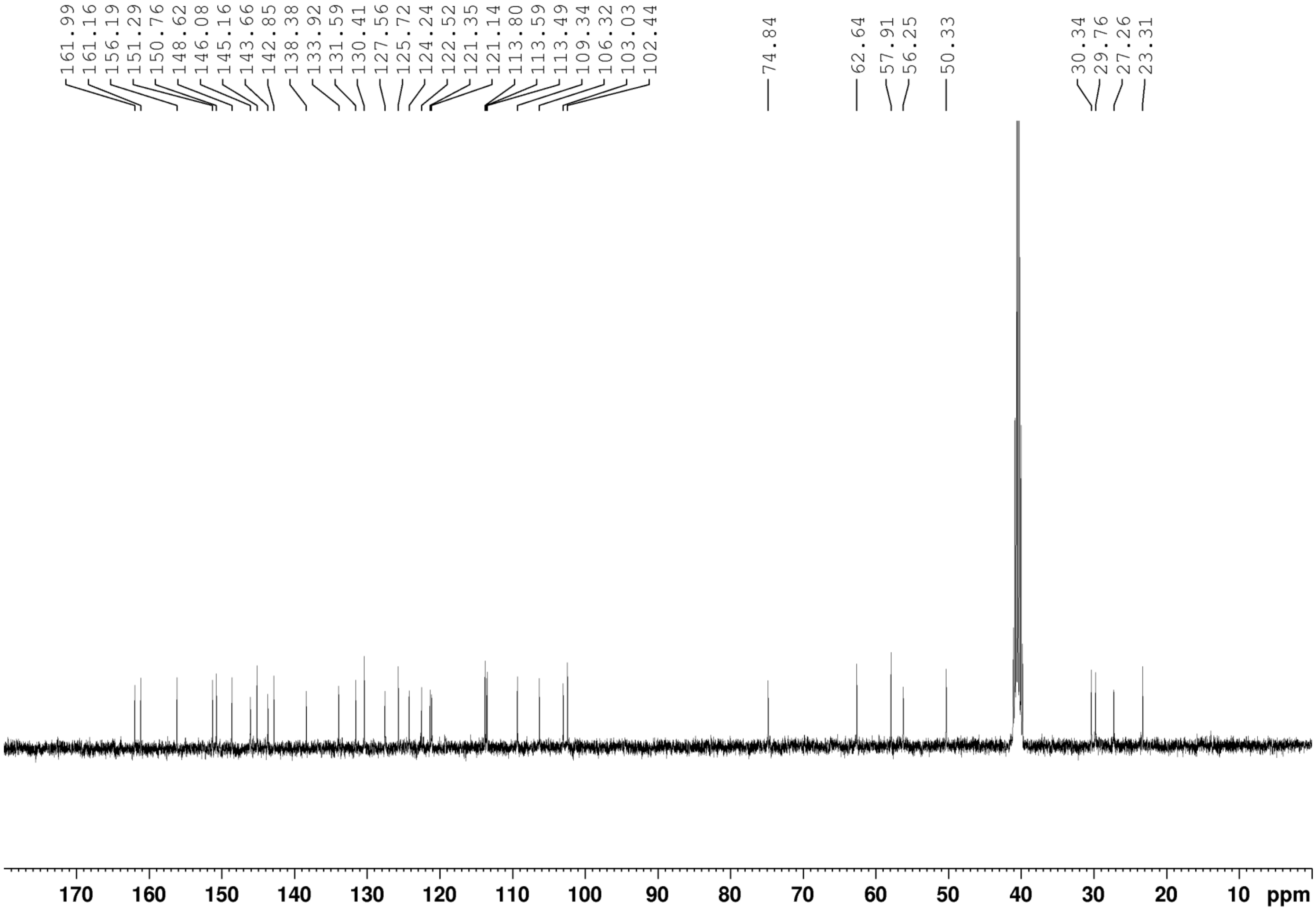

161.99
161.16
156.19
151.29
150.76
148.62
146.08
145.16
143.66
142.85
138.38
133.92
131.59
130.41
127.56
125.72
124.24
122.52
121.35
121.14
113.80
113.59
113.49
109.34
106.32
103.03
102.44
74.84
62.64
57.91
56.25
50.33
30.34
29.76
27.26
23.31
170
160
150
140
130
120
110
100
90
80
70
60
50
40
30
20
10
ppm

$^{1}$H-NMR **40**

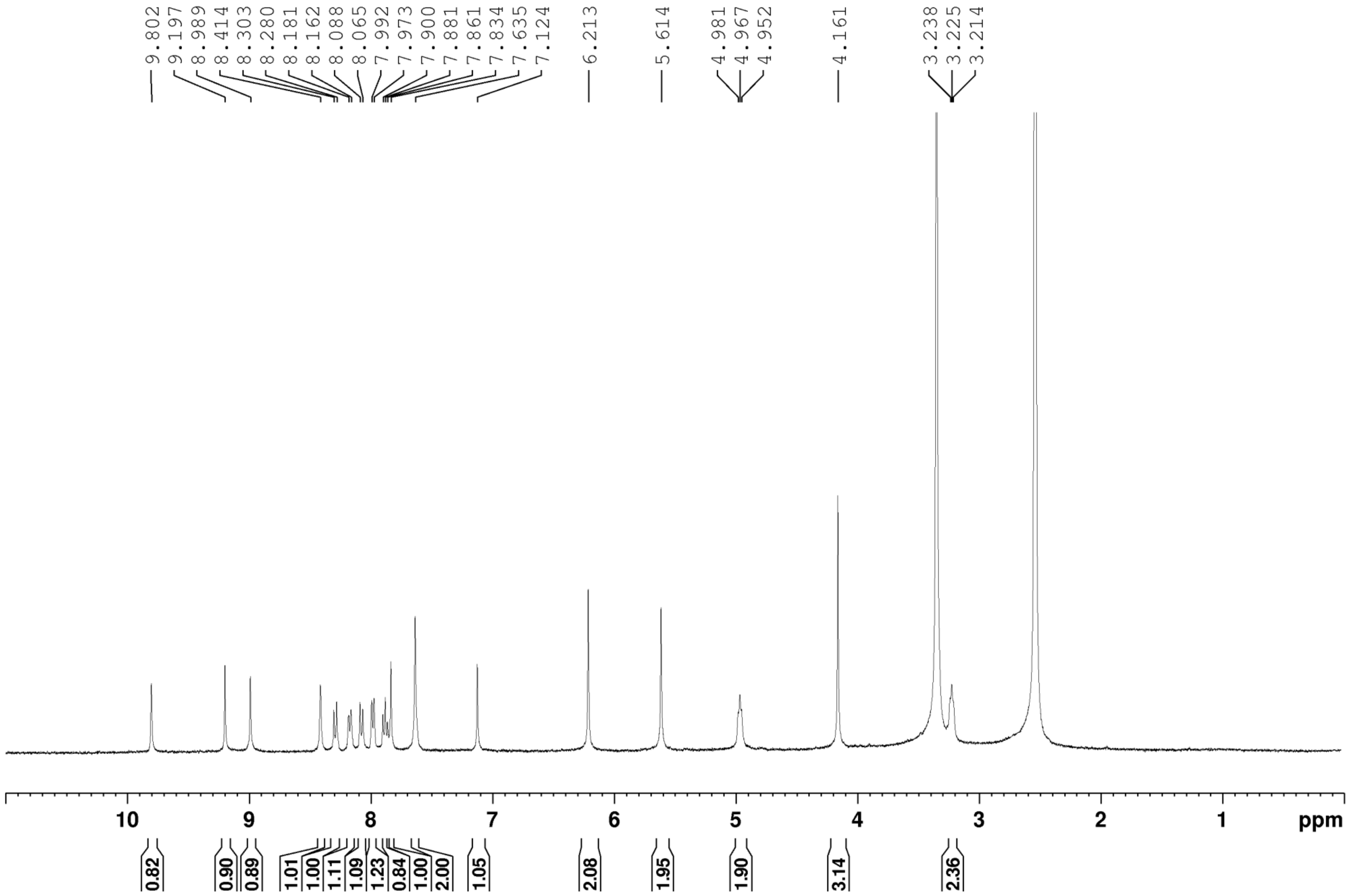
9.802
9.197
8.989
8.414
8.303
8.280
8.181
8.162
8.088
8.065
7.992
7.973
7.900
7.881
7.861
7.834
7.635
7.124
6.213
5.614
4.981
4.967
4.952
4.161
3.238
3.225
3.214
10
9
8
7
6
5
4
3
2
1
ppm
0.82
0.90
0.89
1.01
1.00
1.11
1.09
1.23
0.84
1.00
2.00
1.05
2.08
1.95
1.90
3.14
2.36

$^{13}$C-NMR **40**

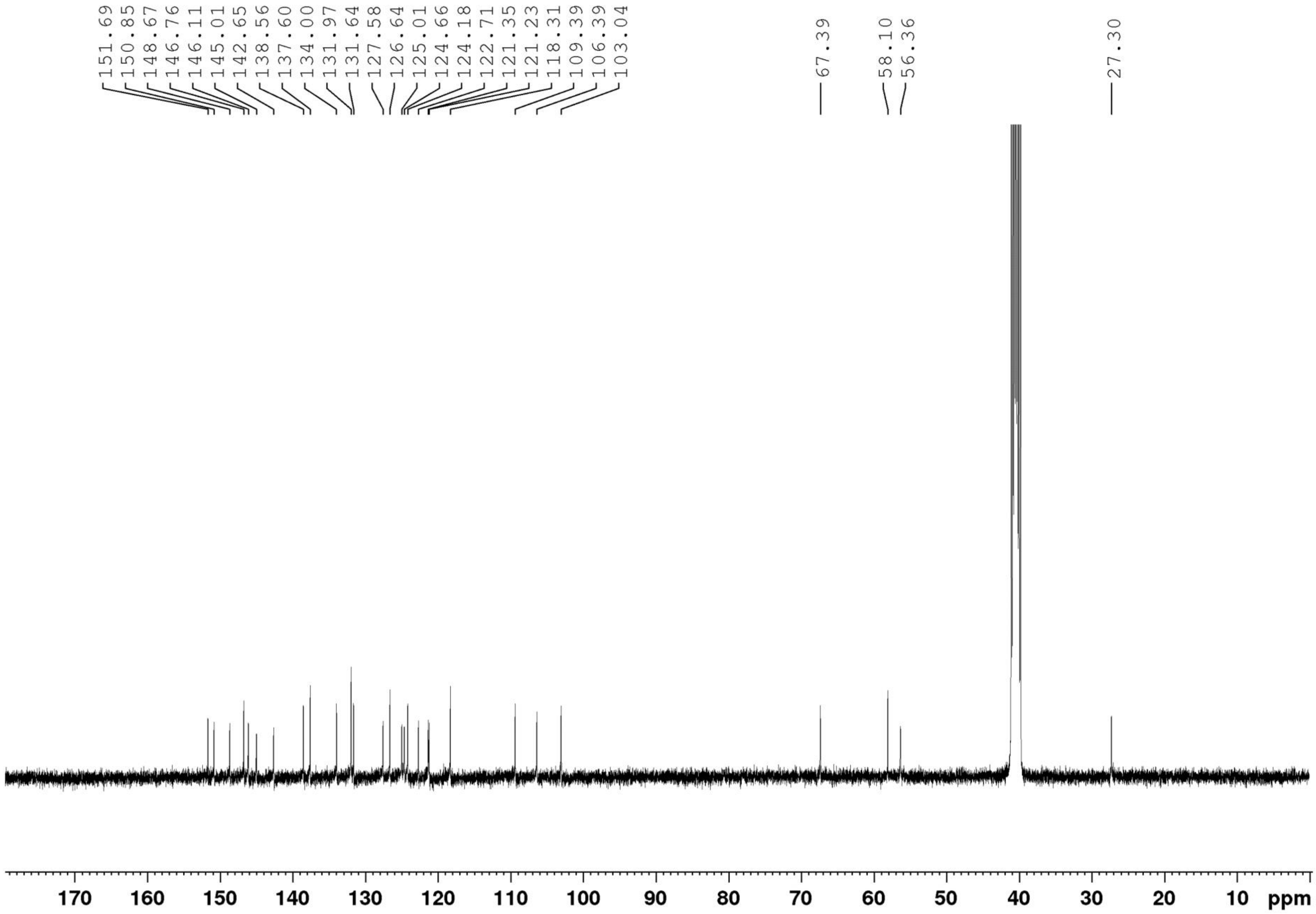
151.69
150.85
148.67
146.76
146.11
145.01
142.65
138.56
137.60
134.00
131.97
131.64
127.58
126.64
125.01
124.66
124.18
122.71
121.35
121.23
118.31
109.39
106.39
103.04
67.39
58.10
56.36
27.30
170
160
150
140
130
120
110
100
90
80
70
60
50
40
30
20
10
ppm

$^1$H-NMR **41**

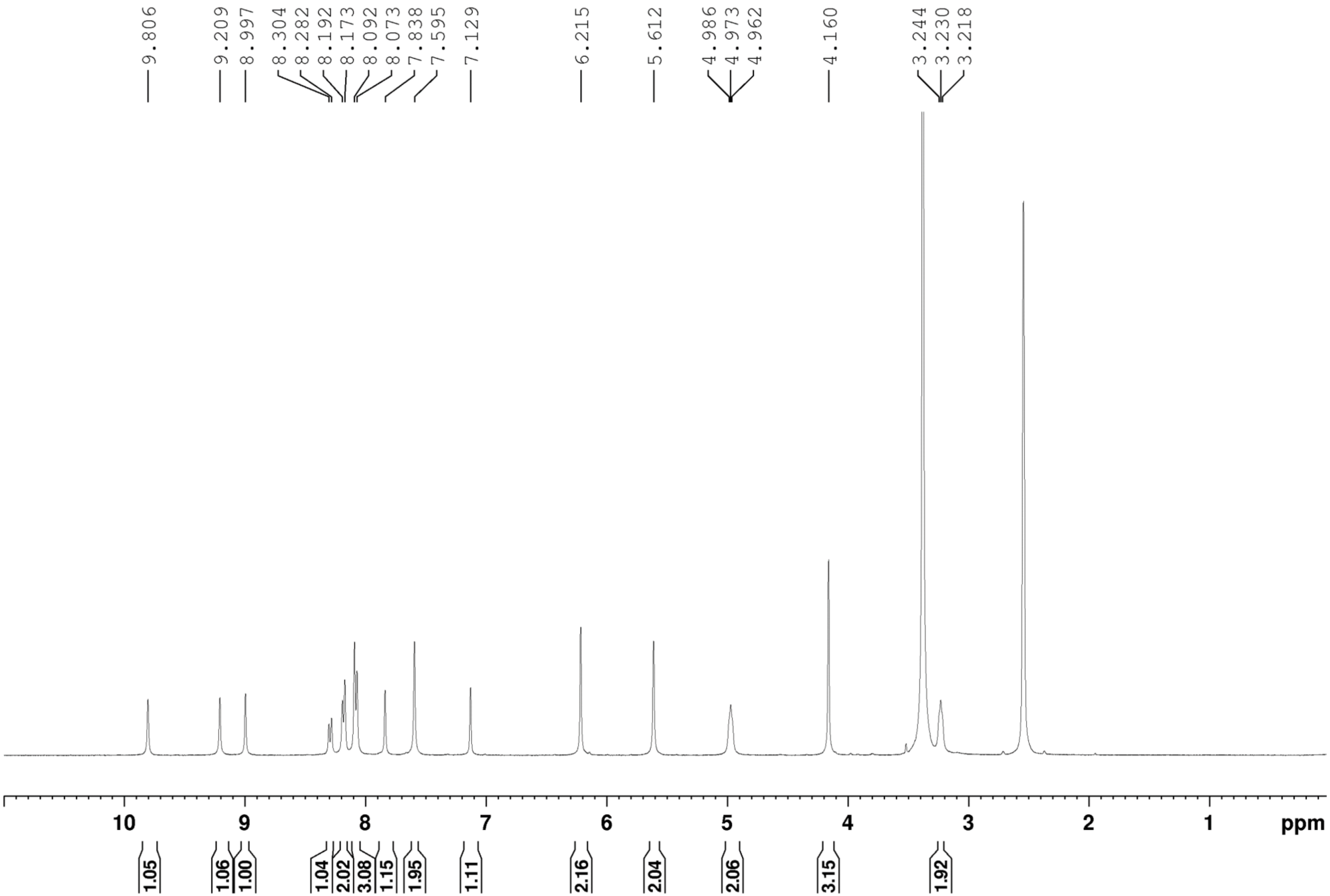

9.806
9.209
8.997
8.304
8.282
8.192
8.173
8.092
8.073
7.838
7.595
7.129
6.215
5.612
4.986
4.973
4.962
4.160
3.244
3.230
3.218
10
9
8
7
6
5
4
3
2
1
ppm
1.05
1.06
1.00
1.04
2.02
3.08
1.15
1.95
1.11
2.16
2.04
2.06
3.15
1.92

$^{13}$C-NMR **41**

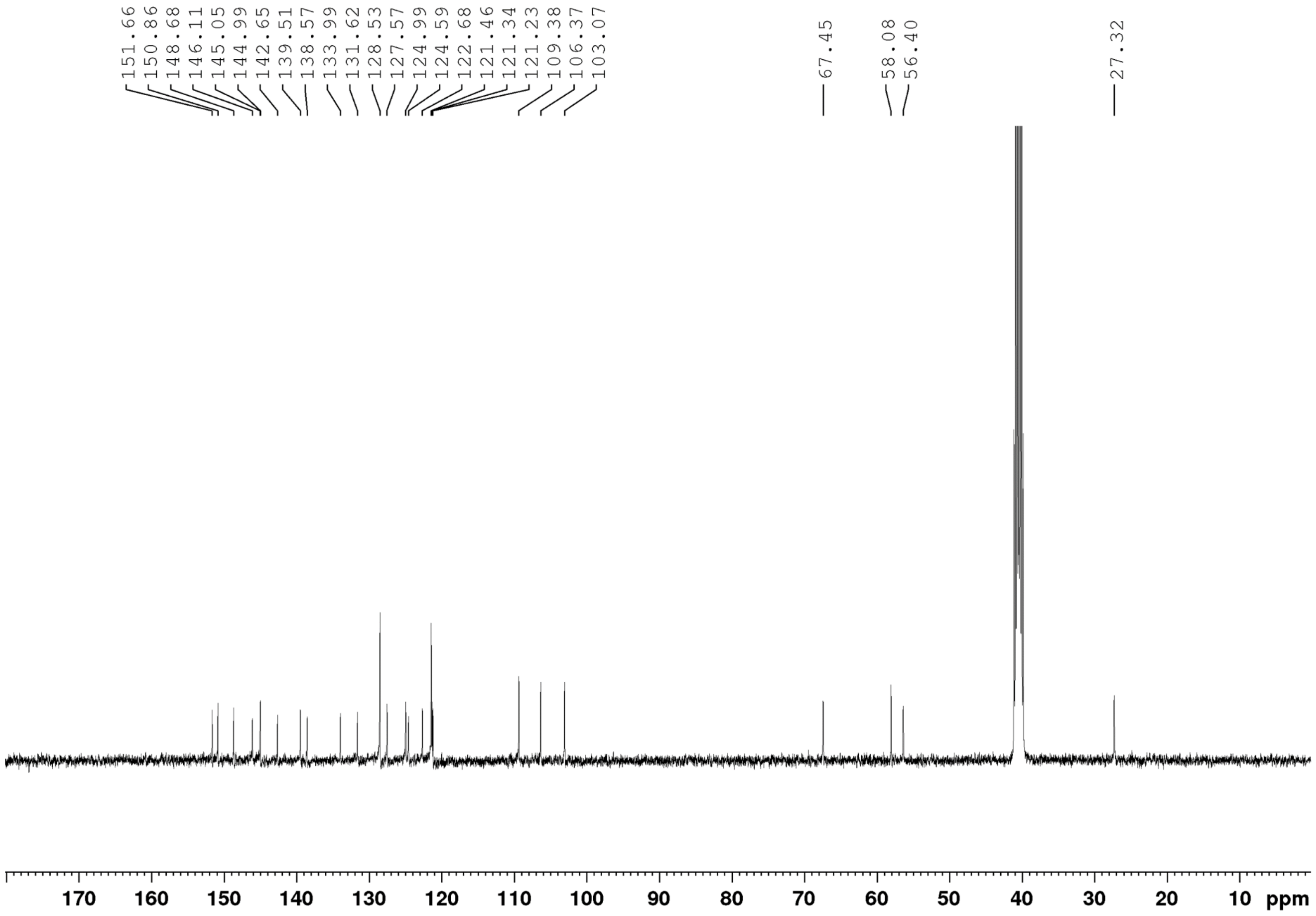
151.66
150.86
148.68
146.11
145.05
144.99
142.65
139.51
138.57
133.99
131.62
128.53
127.57
124.99
124.59
122.68
121.46
121.34
121.23
109.38
106.37
103.07
67.45
58.08
56.40
27.32
170 160 150 140 130 120 110 100 90 80 70 60 50 40 30 20 10 ppm

$^{1}$H-NMR **42**

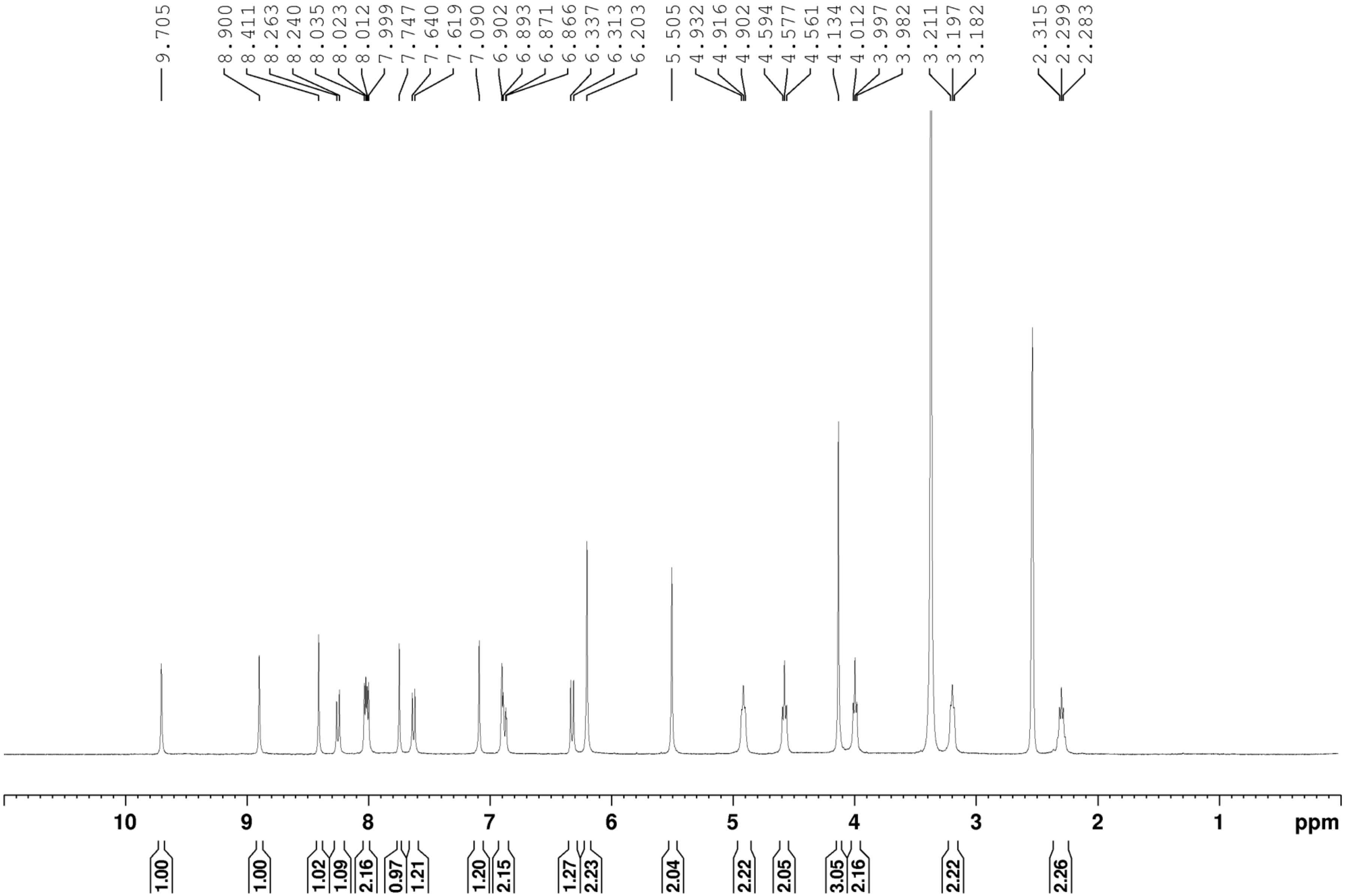
9.705
8.900
8.411
8.263
8.240
8.035
8.023
8.012
7.999
7.747
7.640
7.619
7.090
6.902
6.893
6.871
6.866
6.337
6.313
6.203
5.505
4.932
4.916
4.902
4.594
4.577
4.561
4.134
4.012
3.997
3.982
3.211
3.197
3.182
2.315
2.299
2.283
10
9
8
7
6
5
4
3
2
1
ppm
1.00
1.00
1.02
1.09
2.16
0.97
1.21
1.20
2.15
1.27
2.23
2.04
2.22
2.05
3.05
2.16
2.22
2.26

$^{13}$C-NMR **42**

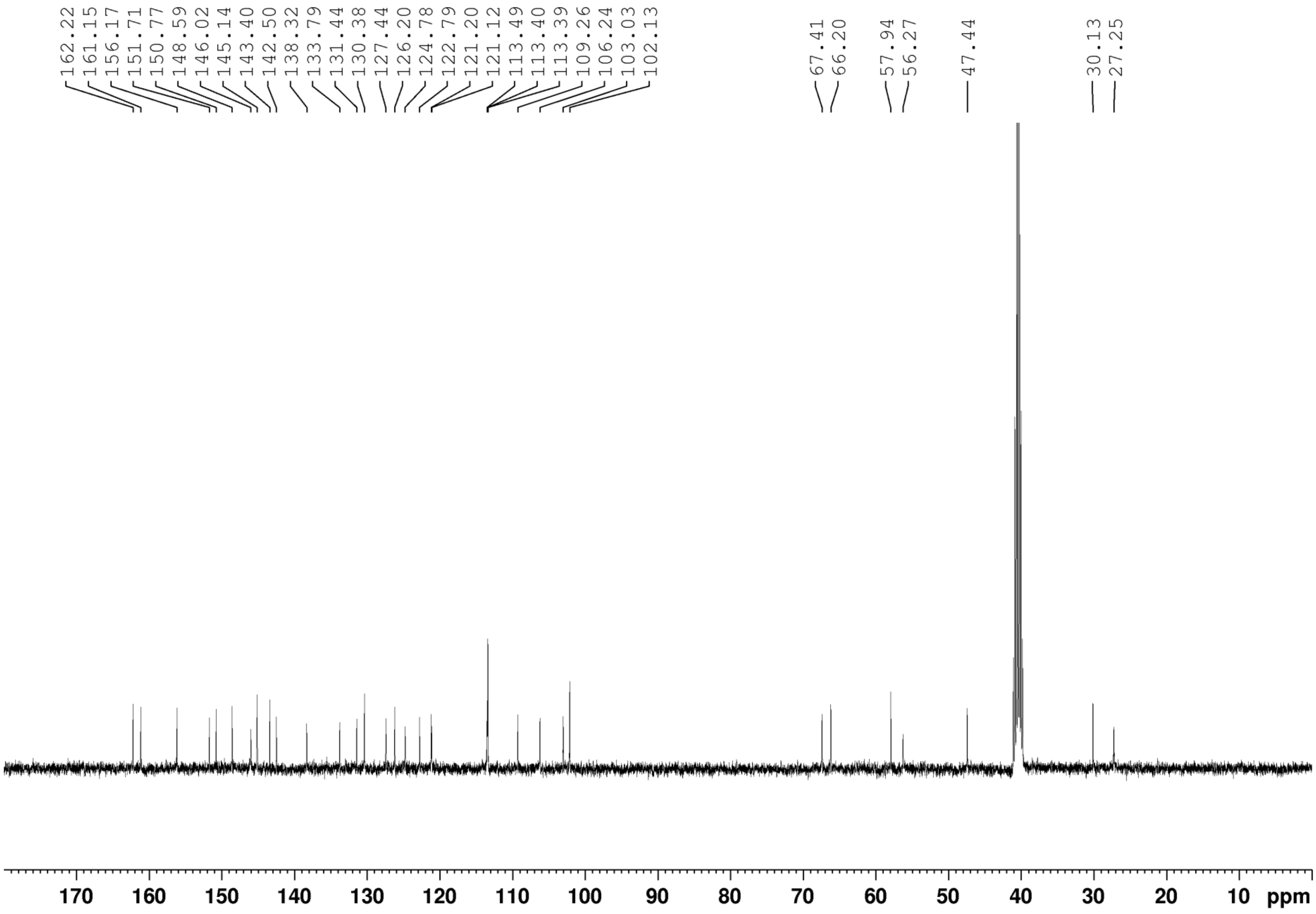
162.22
161.15
156.17
151.71
150.77
148.59
146.02
145.14
143.40
142.50
138.32
133.79
131.44
130.38
127.44
126.20
124.78
122.79
121.20
121.12
113.49
113.40
113.39
109.26
106.24
103.03
102.13
67.41
66.20
57.94
56.27
47.44
30.13
27.25
170
160
150
140
130
120
110
100
90
80
70
60
50
40
30
20
10
ppm

$^1$H-NMR **43**

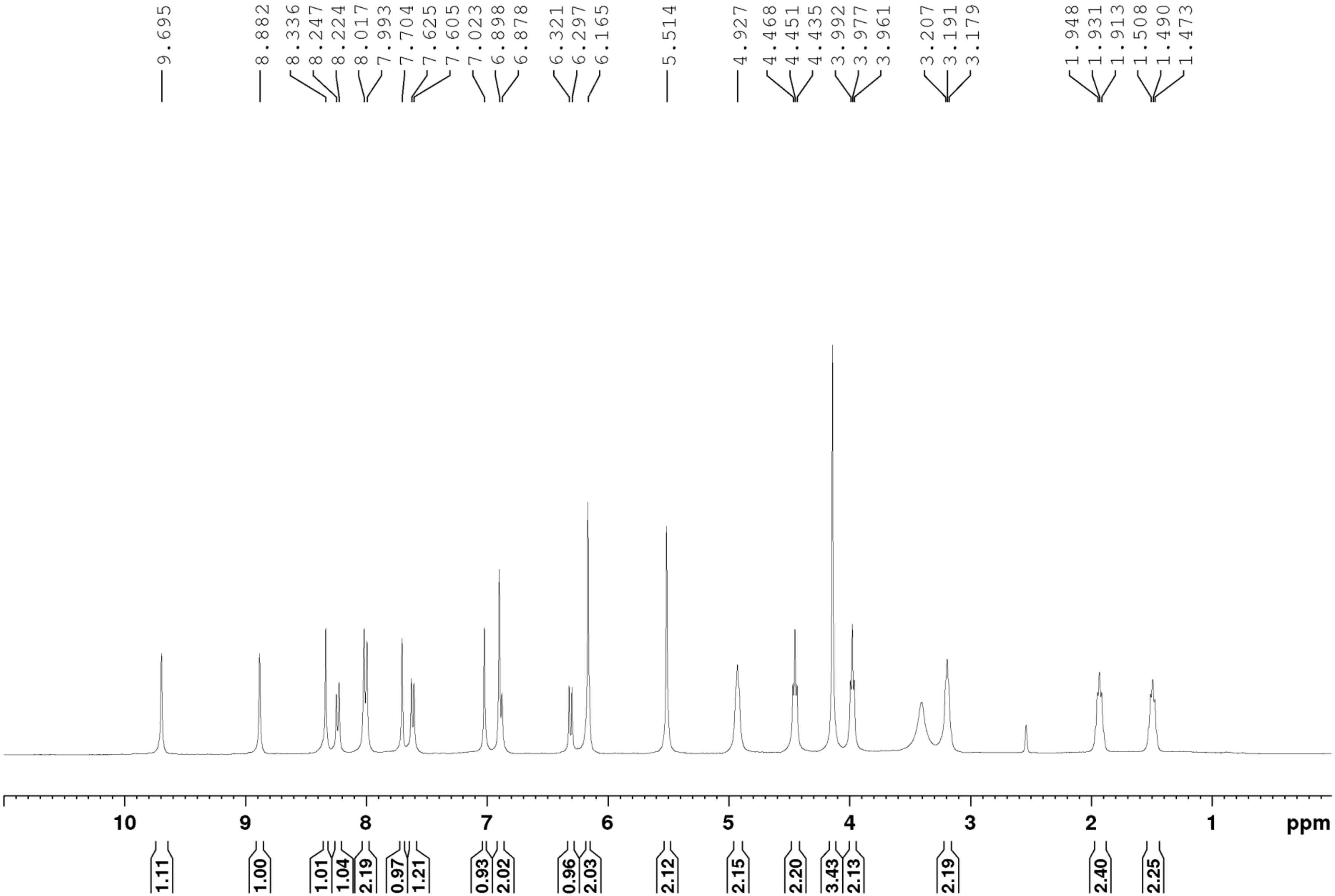
9.695
8.882
8.336
8.247
8.224
8.017
7.993
7.704
7.625
7.605
7.023
6.898
6.878
6.321
6.297
6.165
5.514
4.927
4.468
4.451
4.435
3.992
3.977
3.961
3.207
3.191
3.179
1.948
1.931
1.913
1.508
1.490
1.473
10
9
8
7
6
5
4
3
2
1
ppm
1.11
1.00
1.01
1.04
2.19
0.97
1.21
0.93
2.02
0.96
2.03
2.12
2.15
2.20
3.43
2.13
2.19
2.40
2.25

$^{13}$C-NMR **43**

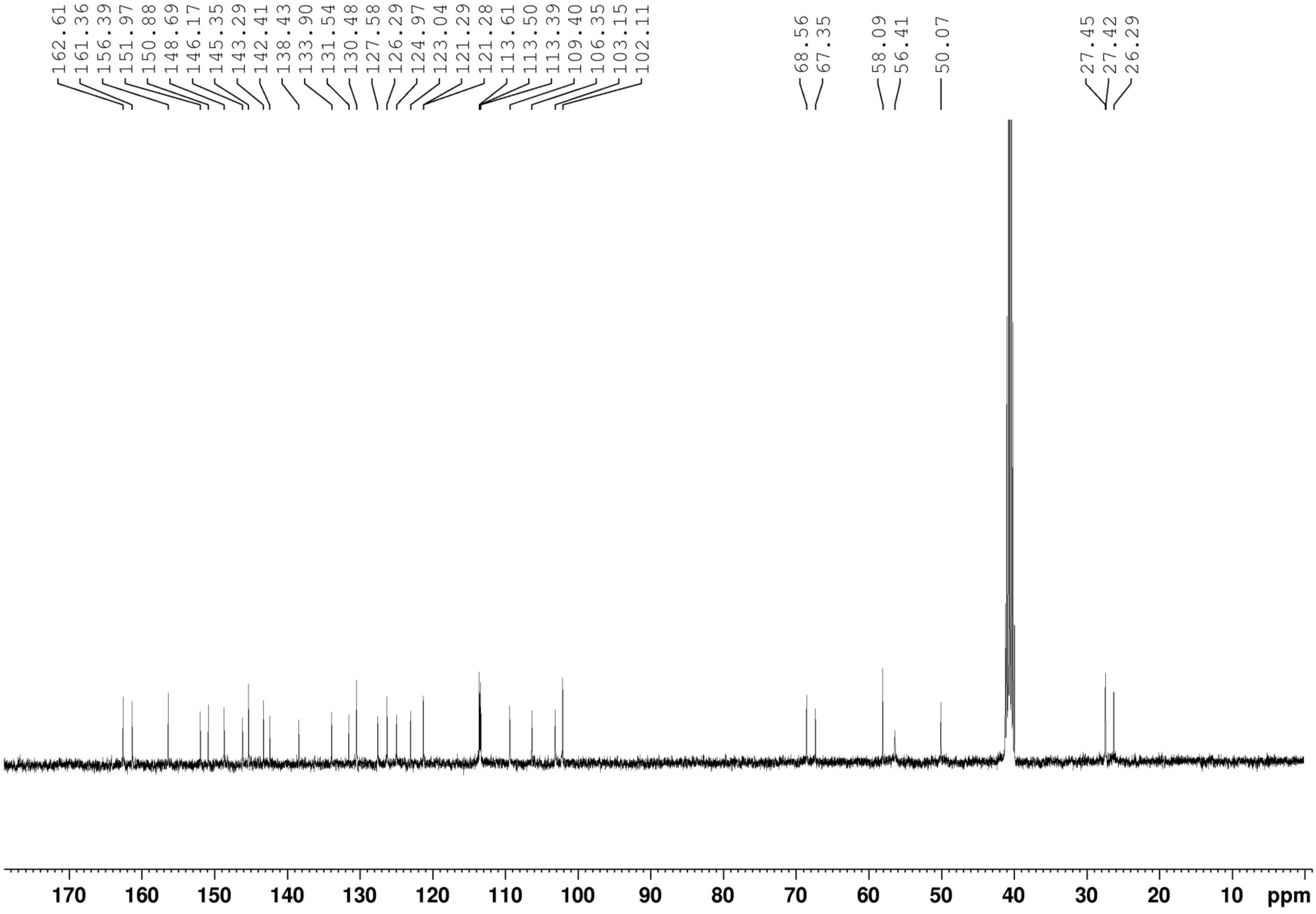

162.61
161.36
156.39
151.97
150.88
148.69
146.17
145.35
143.29
142.41
138.43
133.90
131.54
130.48
127.58
126.29
124.97
123.04
121.29
121.28
113.61
113.50
113.39
109.40
106.35
103.15
102.11
68.56
67.35
58.09
56.41
50.07
27.45
27.42
26.29
170
160
150
140
130
120
110
100
90
80
70
60
50
40
30
20
10
ppm

$^{1}$H-NMR **44**

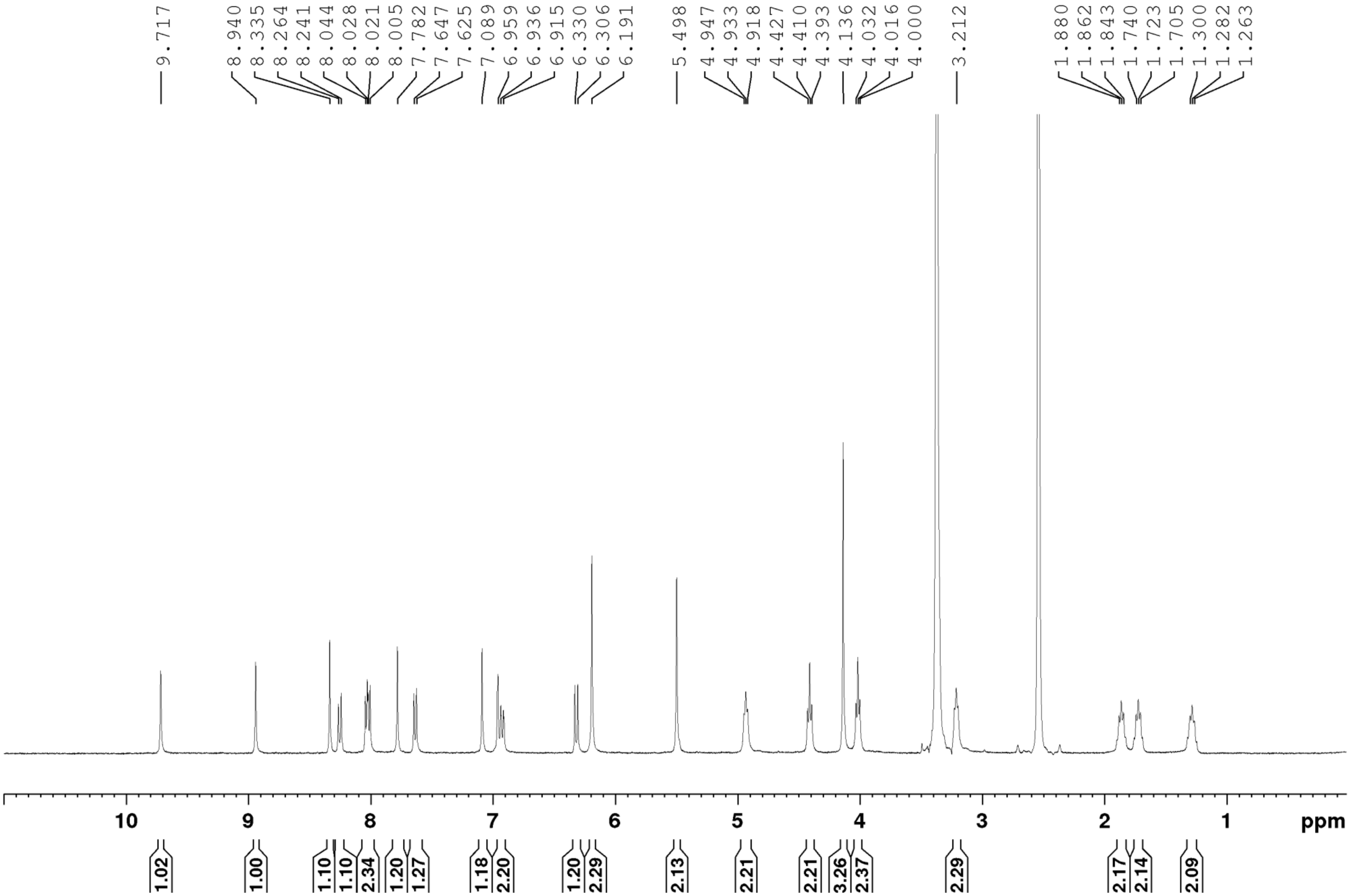
9.717
8.940
8.335
8.264
8.241
8.044
8.028
8.021
8.005
7.782
7.647
7.625
7.089
6.959
6.936
6.915
6.330
6.306
6.191
5.498
4.947
4.933
4.918
4.427
4.410
4.393
4.136
4.032
4.016
4.000
3.212
1.880
1.862
1.843
1.740
1.723
1.705
1.300
1.282
1.263
10
9
8
7
6
5
4
3
2
1
ppm
1.02
1.00
1.10
1.10
2.34
1.20
1.27
1.18
2.20
1.20
2.29
2.13
2.21
2.21
3.26
2.37
2.29
2.17
2.14
2.09

$^{13}$C-NMR **44**

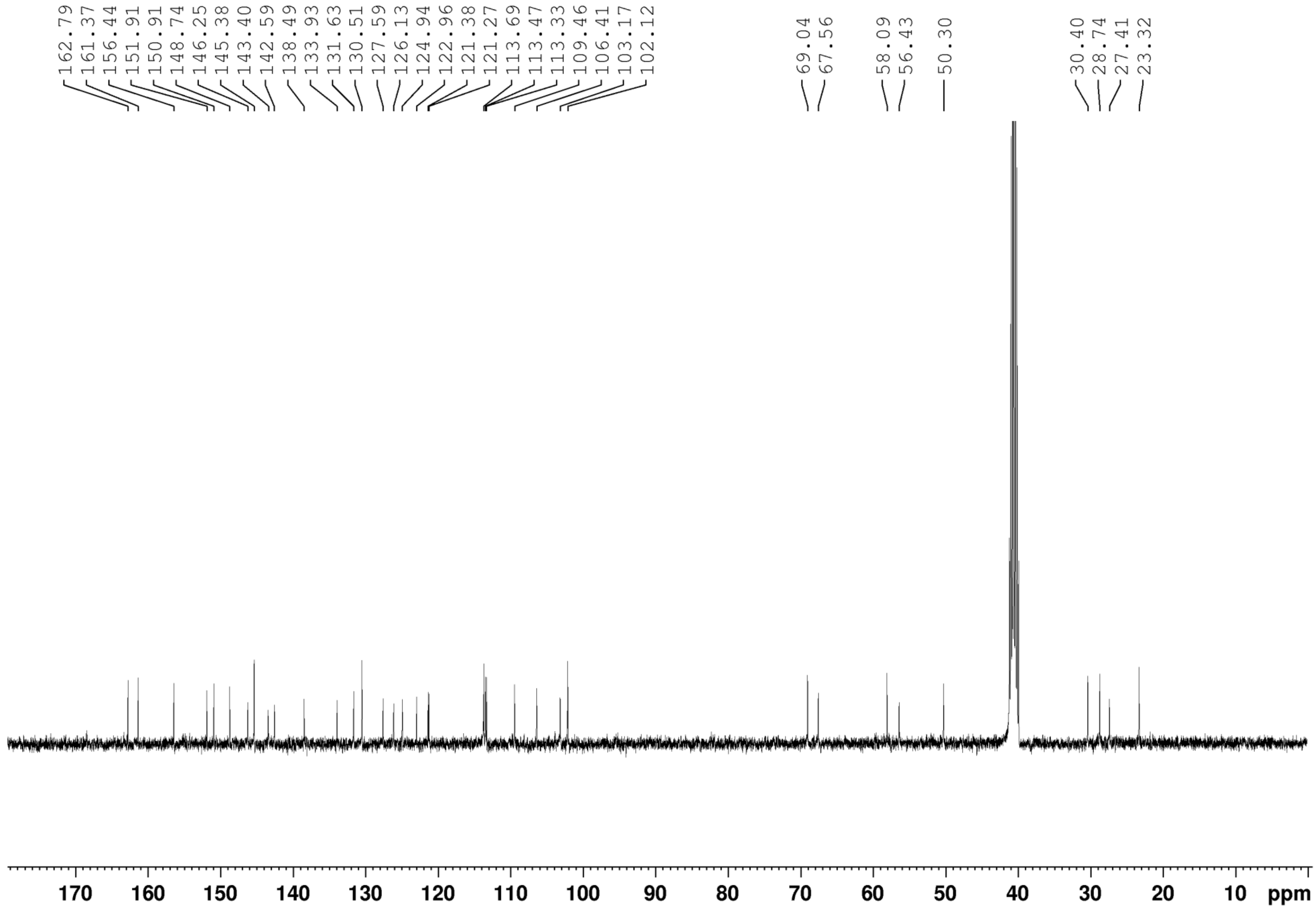

162.79
161.37
156.44
151.91
150.91
148.74
146.25
145.38
143.40
142.59
138.49
133.93
131.63
130.51
127.59
126.13
124.94
122.96
121.38
121.27
113.69
113.47
113.33
109.46
106.41
103.17
102.12
69.04
67.56
58.09
56.43
50.30
30.40
28.74
27.41
23.32
170
160
150
140
130
120
110
100
90
80
70
60
50
40
30
20
10
ppm

$^{1}$H-NMR **45**

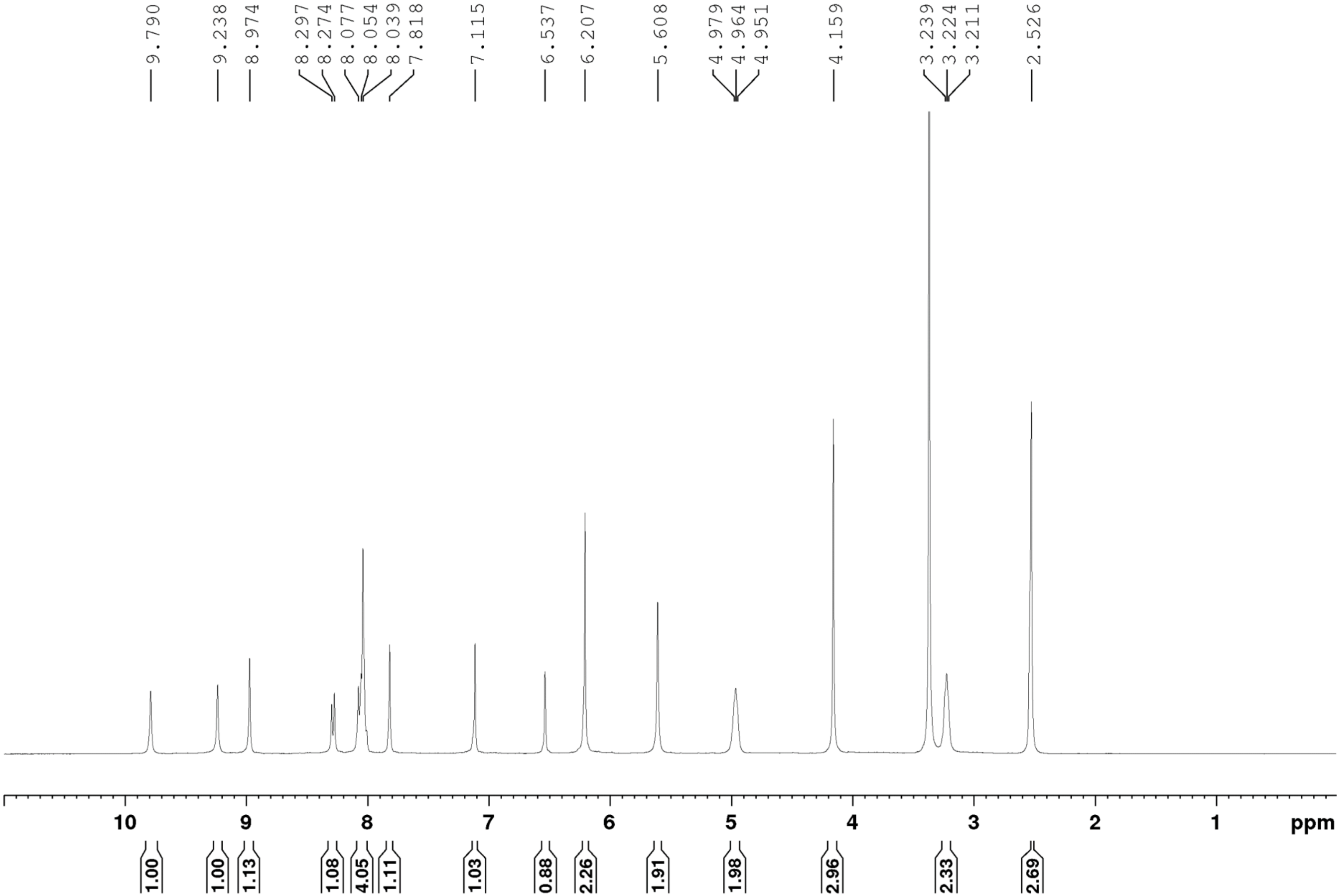
9.790
9.238
8.974
8.297
8.274
8.077
8.054
8.039
7.818
7.115
6.537
6.207
5.608
4.979
4.964
4.951
4.159
3.239
3.224
3.211
2.526
10
9
8
7
6
5
4
3
2
1
ppm
1.00
1.00
1.13
1.08
4.05
1.11
1.03
0.88
2.26
1.91
1.98
2.96
2.33
2.69

$^{13}$C-NMR **45**

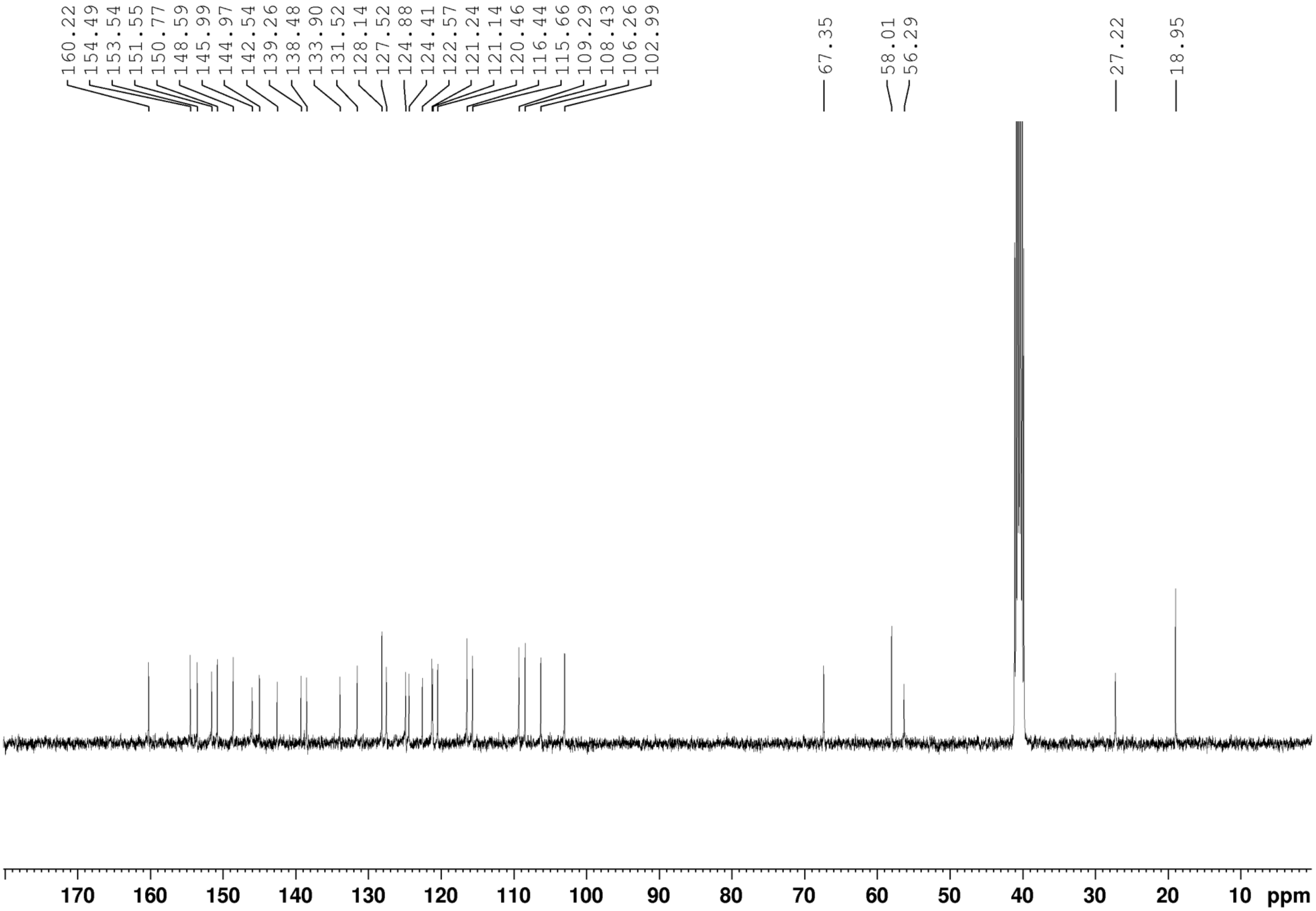
160.22
154.49
153.54
151.55
150.77
148.59
145.99
144.97
142.54
139.26
138.48
133.90
131.52
128.14
127.52
124.88
124.41
122.57
121.24
121.14
120.46
116.44
115.66
109.29
108.43
106.26
102.99
67.35
58.01
56.29
27.22
18.95
170
160
150
140
130
120
110
100
90
80
70
60
50
40
30
20
10
ppm

$^{1}$H-NMR **47**

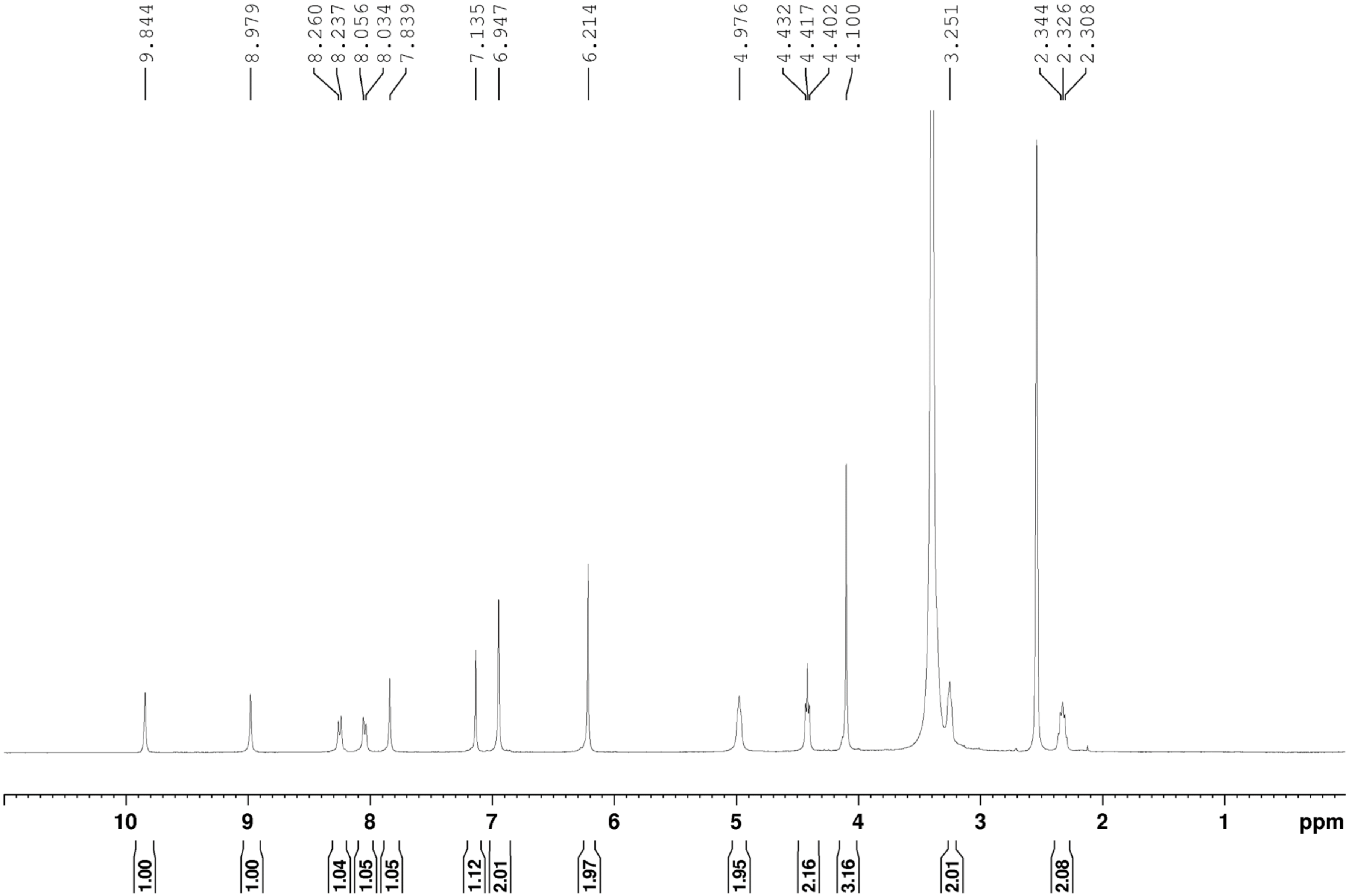
9.844
8.979
8.260
8.237
8.056
8.034
7.839
7.135
6.947
6.214
4.976
4.432
4.417
4.402
4.100
3.251
2.344
2.326
2.308
10
9
8
7
6
5
4
3
2
1
ppm
1.00
1.00
1.04
1.05
1.05
1.12
2.01
1.97
1.95
2.16
3.16
2.01
2.08

$^{13}$C-NMR **47**

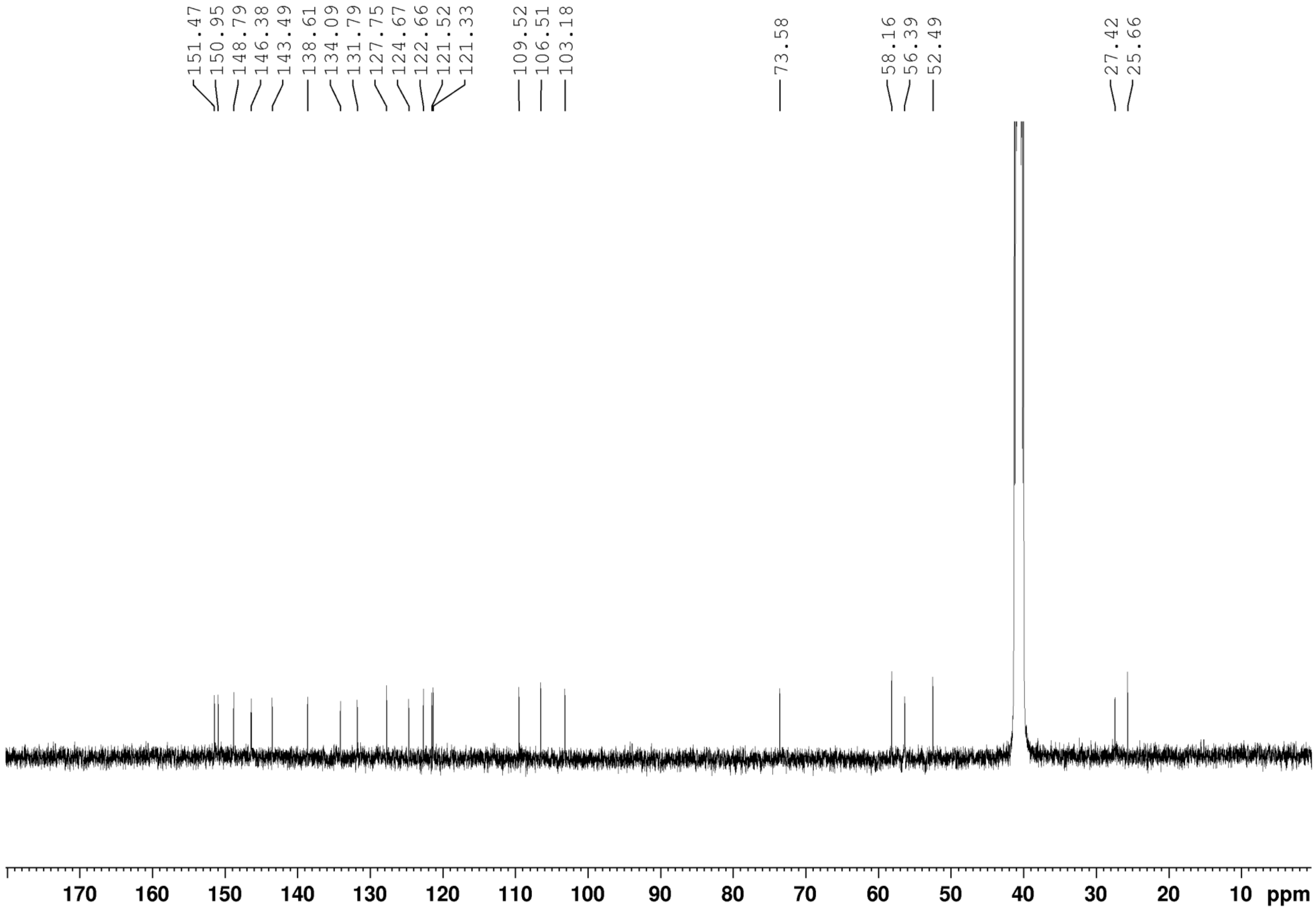
151.47
150.95
148.79
146.38
143.49
138.61
134.09
131.79
127.75
124.67
122.66
121.52
121.33
109.52
106.51
103.18
73.58
58.16
56.39
52.49
27.42
25.66
170 160 150 140 130 120 110 100 90 80 70 60 50 40 30 20 10 ppm

# HPLC traces

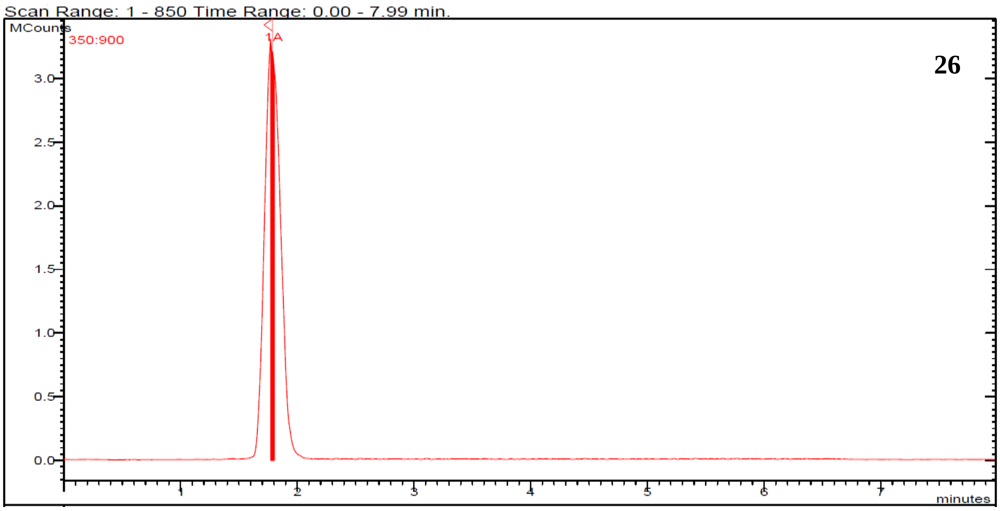
Scan Range: 1 - 850 Time Range: 0.00 - 7.99 min.
MCounts
350:900
1A
26
3.0
2.5
2.0
1.5
1.0
0.5
0.0
1
2
3
4
5
6
7
minutes

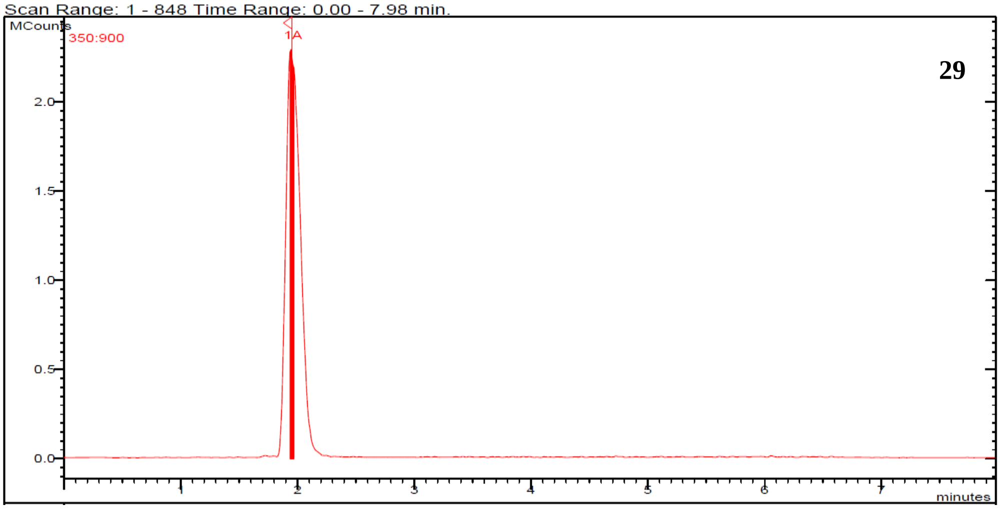
Scan Range: 1 - 848 Time Range: 0.00 - 7.98 min.
MCounts
350:900
1A
29
2.0
1.5
1.0
0.5
0.0
1
2
3
4
5
6
7
minutes

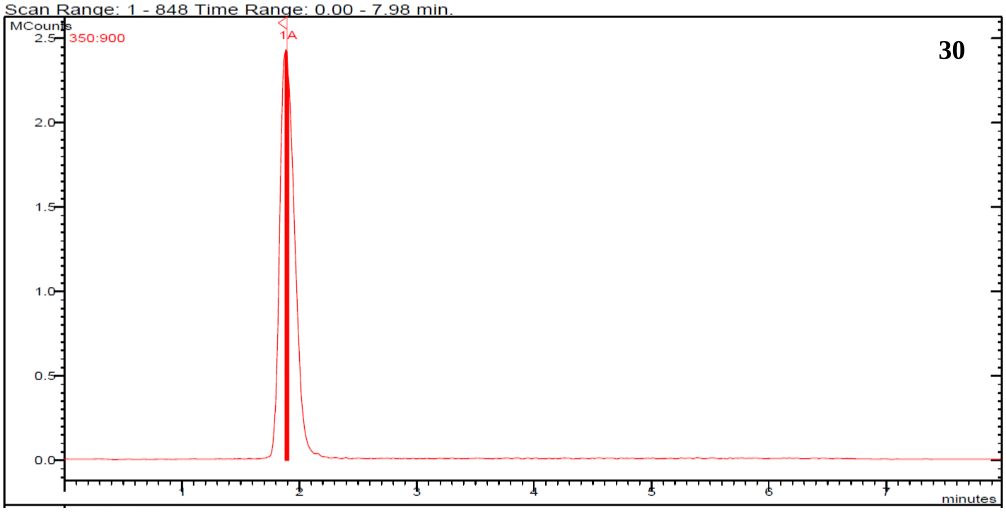
Scan Range: 1 - 848 Time Range: 0.00 - 7.98 min.
MCounts
2.5
350:900
1A
30
2.0
1.5
1.0
0.5
0.0
1
2
3
4
5
6
7
minutes

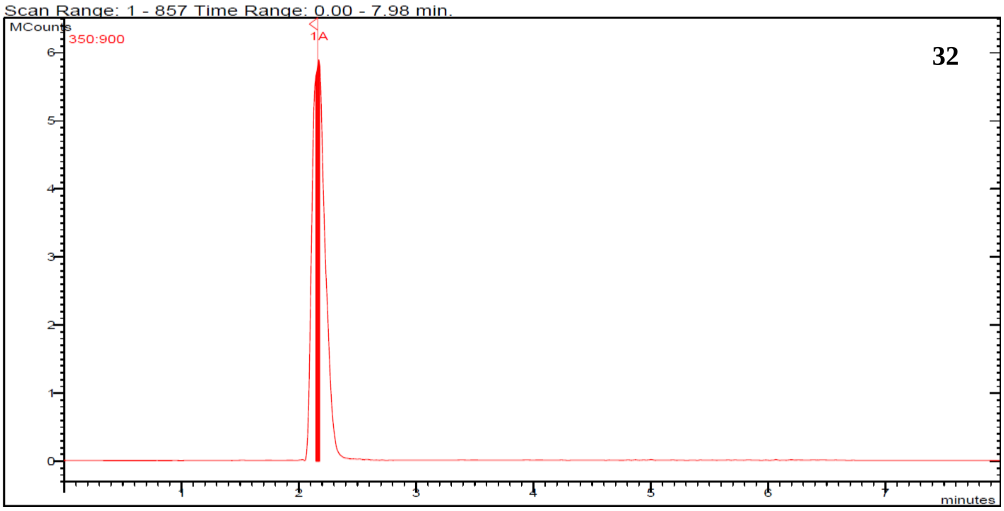
Scan Range: 1 - 857 Time Range: 0.00 - 7.98 min.
MCounts
350:900
1A
32
minutes

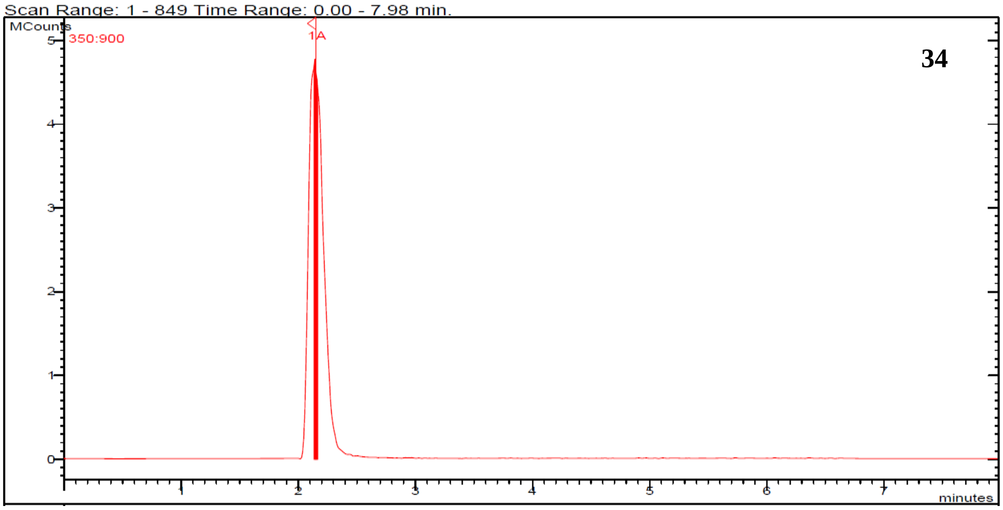
Scan Range: 1 - 849 Time Range: 0.00 - 7.98 min.
MCounts
350:900
1A
34
minutes

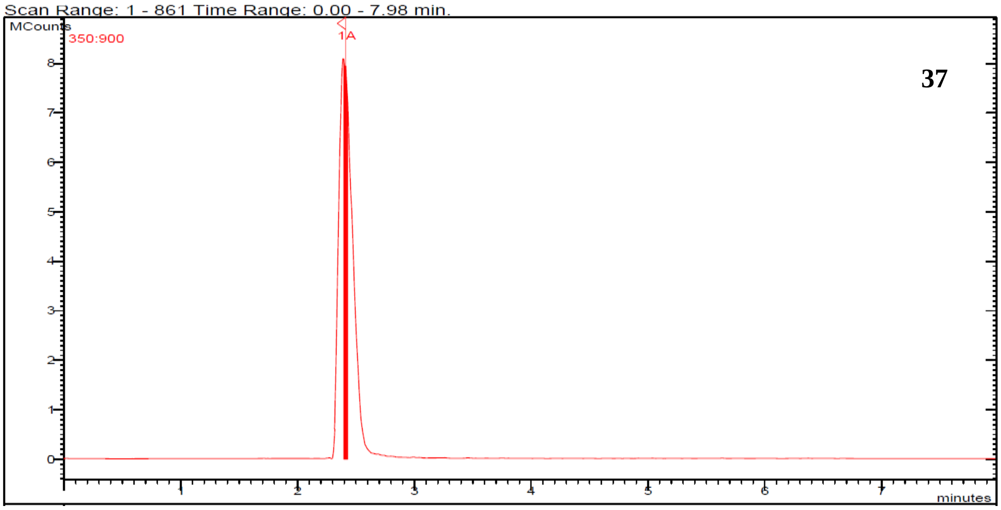
Scan Range: 1 - 861 Time Range: 0.00 - 7.98 min.
MCounts
350:900
1A
37
minutes

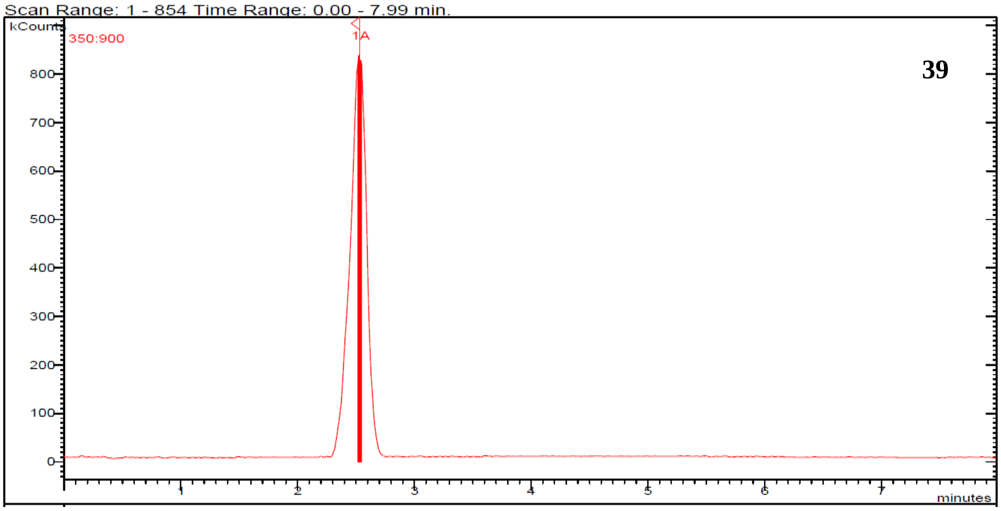

Scan Range: 1 - 854 Time Range: 0.00 - 7.99 min.
kCounts
350:900
1A
39
800
700
600
500
400
300
200
100
0
1
2
3
4
5
6
7
minutes
